# Mortality from gastrointestinal congenital anomalies at 264 hospitals in 74 low-income, middle-income, and high-income countries: a multicentre, international, prospective cohort study

**DOI:** 10.1016/S0140-6736(21)00767-4

**Published:** 2021-07-24

**Authors:** Global PaedSurg Research Collaboration, Naomi Jane Wright, Andrew J.M. Leather, Niyi Ade-Ajayi, Nick Sevdalis, Justine Davies, Dan Poenaru, Emmanuel Ameh, Adesoji Ademuyiwa, Kokila Lakhoo, Emily Rose Smith, Abdel Douiri, Maria Elstad, Marcus Sim, Cristiana Riboni, Bruno Martinez-Leo, Melika Akhbari, Stephen Tabiri, Ashrarur Mitul, Dayang Anita Abdul Aziz, Camila Fachin, Alliance Niyukuri, Muhammad Arshad, Fowzia Ibrahim, Natalie Moitt, Mohamed Fahmy Doheim, Hannah Thompson, Harmony Ubhi, Isabelle Williams, Sophia Hashim, Godfrey Sama Philipo, Laura Herrera, Aayenah Yunus, Dominique Vervoort, Samuel Parker, Yousra-Imane Benaskeur, Osaid H. Alser, Nana Adofo-Ansong, Ahmad Alhamid, Hosni khairy Salem, Mahmoud Saleh, Safa Abdal Elrais, Sadi Abukhalaf, Patricia Shinondo, Ibrahim Nour, Emrah Aydin, Agota Vaitkiene, Kelly Naranjo, Andile Maqhawe Dube, Sodumisa Ngwenya, Mina A. Yacoub, Henang Kwasau, Gabriella Hyman, Shrouk Mahmoud Elghazaly, Ibrahim Al-Slaibi, Intisar Hisham, Helena Franco, Hana Arbab, Lubna Samad, Aqil Soomro, Muhammad Amjad Chaudhry, Safina Karim, Muhammad Adnan Khan Khattak, Shireen Anne Nah, Doris Mae Dimatatac, Candy SC Choo, Niveshni Maistry, Ashrarur Rahman Mitul, Samiul Hasan, Sabbir Karim, Hina Yousuf, Taimur Qureshi, Ibrahim Rabi Nour, Raed Nael Al-Taher, Osama Abdul Kareem Sarhan, Luis Garcia-Aparicio, Jordi Prat, Eva Blazquez-Gomez, Xavier Tarrado, Martí Iriondo, Paolo Bragagnini, Segundo Rite, Lars Hagander, Emma Svensson, Sheila Owusu, Alhassan Abdul-Mumin, Dominic Bagbio, Vijay Anand Ismavel, Ann Miriam, Shajin T, Marlene Anaya Dominguez, Monica Ivanov, Andreea Madalina Serban, Miliard Derbew, Mahmoud Elfiky, Maricarmen Olivos Perez, Marcia Abrunhosa Matias, Alexis P Arnaud, Ahmed Negida, Sebastian King, Mohamad Rafi Fazli, Nadia Hamidi, Souhem Touabti, Rossana Francisco Chipalavela, Pablo Lobos, Brendan Jones, Damir Ljuhar, Georg Singer, Samiul Hasan, Annelien Cordonnier, Lorena Jáuregui, Zlatan Zvizdic, Janice Wong, Etienne St-Louis, Qiang Shu, Yang Lui, Catalina Correa, Lucie Pos, Elvyn Alcántara, Erick Féliz, Luis Enrique Zea-Salazar, Liza Ali, Matthieu Peycelon, Nzanzu Kipata Anatole, Cherno S. Jallow, Judith Lindert, Dhruv Ghosh, Cathline Freya Adhiwidjaja, Ahmad Khaleghnejad Tabari, Saran Lotfollahzadeh, Haidar Mohammad Mussein, Fabrizio Vatta, Noemi Pasqua, David Kihiko, Hetal Gohil, Ibrahim R. Nour, Muhammed Elhadi, Suad Ahmed Almada, Gilvydas Verkauskas, Toni Risteski, Alejandro Peñarrieta Daher, Oumaima Outani, James Hamill, Taiwo Lawal, Jack Mulu, Benjamin Yapo, Lily Saldaña, Beda Espineda, Krystian Toczewski, Eugene Tuyishime, Isaac Ndayishimiye, Enaam Raboe, Philip Hammond, Gregor Walker, Ivona Djordjevic, Milind Chitnis, Joonhyuk Son, Sanghoon Lee, Muaad Hussien, Sawazen Malik, Enas Musa Ismail, Ampaipan Boonthai, Nesrine Ben Hadj Dahman, Nigel Hall, Fabiola Ruth Castedo Camacho, Helena Sobrero, Marilyn Butler, Aliev Makhmud, Nathan Novotny, Ahmad G. Hammouri, Maisara Al-Rayyes, Bruce Bvulani, Qais Muraveji, Muhammad Yousuf Murzaie, Ajmal Sherzad, Sayed Aman Haidari, Abdul Baqi Monawar, Dr. Ahmad Zia Samadi, Jesh Thiessen, Ntakarutimana Venant, Sonia Inamuco Hospital, Niyonkuru Jérémie, Jean Claude Mbonicura, Butoyi Jean Marie Vianney, Amezene Tadesse, Samuel Negash, Charles A. Roberts, John N. Jabang, Abdoulie Bah, Kajali Camamra, Armandou Correa, Babucarr Sowe, A. Gai, Musa Jaiteh, Kwizera Jean Raymond, Jean Paul Mvukiyehe, Innocent Itangishaka, Emmanuel Kayibanda, Emery Manirambona, Joseph Lule, Ainhoa Costas-Chavarri, Ian Shyaka Gashugi, Albert Ndata, Georges Gasana, Yves Castar Nezerwa, Turatsinze Simeon, Jean De Dieu Muragijimana, Sakina Rashid, David Msuya, Joseph Elisante, Meghna Solanki, Emmanuel Manjira, Jay Lodhia, Mubashir Jusabani, Murad Tarmohamed, Sengua Koipapi, Touabti Souhem, Nabti Sara, Brahimi Sihem, Bouguermouh Dania, Iaiche Achour Toufik, Baghdadi Nour el islam Mounira, Alouani Habiba, Liliana Aragão, Victor Gonçalves, Marcelo Mauricio Lino Urquizo, Maria Florencia Varela, Pedro Mercado, Bonavia Horacio, Andrea Damiani, Carlos Mac, Daniel Putruele, Karen Liljesthrom, Marianela Bernaus, Cesar Jauri, Alejandrina Cripovich, Ezequiel Bianchin, Maria Gabriela Puig, Lorna Andreussi, Susana Iracelay, Dolores Marcos, Carina Herrera, Nelly Palacios, Romina Avile, Belen Serezo, Debora Montoya, Rodrigo Cepeda, Justo Vaquila, Sofficci Veronica, Liliana Pardo, Pelussi Valeria, Lapalma Julio, Aranda Diego Martin, Palazzi Lucio, Comba Gabriel, Depetrini Marianella, José Alfredo Calderón Arancibia, Enrique Huespe, Gabriela Natalia Losa, Elsa Arancibia Gutiérrez, Humberto Scherl, Daniel Emilio Gonzalez, Valentina Baistrocchi, Yanina Silva, Marcelo Galdeano, Pablo Medard, Ines Sueiras, Enrique Romero Manteola, Victor Hugo Defago, Carlos Mieres, Carlos Alberto, Fabio Cornelli, Marcelo Molina, Pablo Ravetta, Celeste Carolina Patiño Gonzalez, Maria Belen Dallegre, Maria Tatiana Szklarz, Marcos Federico Leyba, Nahuel Ignacio Rivarola, Maria Delia Charras, Adriana Morales, Paloma Caseb, Luzia Toselli, Carolina Millán, Maria del Carmen Junes, Oscar Di Siervi, Jose Gilardi, Soledad Simon, Carla Sofia Contreras, Nair Rojas, Lucia Beatriz Arnoletto, Otilia Eva Blain, Mauro Nicolas Bravo, Nancy Sanchez, Luciana Martina Herrera Pesara, Maria Eugenia Moreno, Carlos Ariel Sferco, Umama Huq, Tamanna Ferdousi, Abdullah Al-Mamun, Sadia Sultana, Refoyez Mahmud, Khalid Mahmud, Fatema Sayeed, Alexander Svirsky, Denisse Sempertegui, Amalia Negrete, Araceli Teran, Mariana Sadagurschi, Nusret Popovic, Kenan Karavdic, Emir Milisic, Asmir Jonuzi, Amira Mesic, Sabina Terzic, Nejra Dendusic, Elna Biber, Anesa Sehic, Nada Zvizdic, Emina Letic, Adna Saracevic, Ajla Hamidovic, Nejra Selak, Dzan Horozic, Lamija Hukic, Amila Muhic, Nedim Vanis, Emir Sokolovic, Adnan Sabic, Karin Becker, Elis Novochadlo Klüppel, André Iván Bradley dos Santos Dias, Miguel Angelo Agulham, Cristiano Bischoff, Stella Sabbatini, Rachel Fernandes de Souza, Ana Beatriz Souza Machado, Juliana Werneck Raposo, Maria Lucia da Silva Augusto, Bianca M.R. Martins, Mariana de Souza Santos Ferreira, Darli Fernandes de Oliveira, Carla Silva dos Santos, Fernanda Ribeiro de Fernández y Alcázar, Érika Alves Dutra da Silva, Mariana Furtado, Horácio Tamada, Marília Silva Ferreira dos Santos, Thayná Lopes de Almeida, Susy Oliveira de Andrade, Antonio Cipriano Gurgel do Amaral, Lais Sartori Giovanoni, Kamila de Deus Passos Leles, Eduardo Corrêa Costa, Leticia Feldens, Luciano Ferraz Schopf, José Carlos Soares de Fraga, Felipe Colombo de Holanda, Paola Maria Brolin Santis Isolan, Julia Loyola Ferreira, Carla Luisa Bruxel, Danielle Lopes Teixeira Ferdinando, Fabricio Zottis Barcelos, Natalia Baseggio, Nicole Knorr Brenner, Rafael Trindade Deyl, Carolina Dure, Iuri Nunes Kist, Rafael Bueno Mazzuca, Sarah Bueno Motter, Yna Ramos, Cristine Suzana Trein, Bianca Rezende Rosa, Murilo de Assis Silva, Flavio Augusto Menin, Isabela Cristina Semensato Carloni, Juliana Antinarelli Norberto da Silva, Adriano Luis Gomes, Mariana Girão Tauffer, Paulo César Bassan Gonçalves, Geraldo Magela Nogueira Marques, Eliane Moriya, Carla Labonia, Ana Lucia Carrasco, Karine Furtado Meyer, Luiz Farion-Aguiar, Fernando Amado, Amanda Antunes, Elisângela Silva, Leila Telles, Giovana Almeida, Aluísio Augusto Belmino Gadelha, Flavia de Azevedo Belesa, Acimar Gonçalves da Cunha, Jr, Beatriz Souza Barros, Josiane Bernartt Zanellato, Patricia Guimarães, Karina Ilheu da Silva, Bianca Ribas, Cristina Reuter, Francis Tanise Casado, Mila Torii Correa Leite, Daniela Testoni, Ruth Guinsburg, Simone de Campos Vieira Abib, Edson Khodor Cury, Suely Dornellas do Nascimento, Arthur Almeida Aguiar, Rodrigo Melo Gallindo, Carolina Gonçalves Borges, Yang Liu, Cai Duote, Jinhu Wang, Zhigang Gao, Liang Liang, Wenjuan Luo, Xiaoxia Zhao, Rui Chen, Peng Wang, Yijiang Han, Ting Huang, Hu Donglai, Guo Xiaodong, Chen Junjie, Libin Zhu, Guowei Wu, Xiaozhou Bao, Haijing Li, Junying Lv, Zhongrong Li, Feng Yong, Zhou Chong Gao, Qiang Bai, Weibing Tang, Hua Xie, Jethishka Motee, Jianming Zhu, Gang Wen, Weiwei Ruan, Shungen Li, Lulu Chen, Shungen Huang, Zhibao Lv, Jinjing Lu, Liuming Huang, Mengnan Yu, Wang Dajia, Yu Zuo Bai, Luis Carlos Rincon, Juliana Mancera, Edgar Alzate Gallego, Laura Torres-Canchala, Nathalia Silva Beltrán, Ghordana Osorio Fory, Daniela Castaño Avila, Angelica Maria Forero Ladino, Juanita Gomez, Martha Jaramillo, Otto Morales, Beatriz Sanchez, Nestor Julien Tinoco Guzmán, Sergio Castañeda Espinosa, Osbaldo Prieto Vargas, Lina Maria Pardo, Eliana Toral, Freud Cáceres Aucatoma, Daniel Hinostroza, Santiago Valencia, Vicente Salinas, Enrique Landivar Cino, Gabriela Yulissa Ponce Fajardo, Miguel Astudillo, Virginia Garcia, Guillermo Muñoz, Leonardo Verduga, Ivan Verduga, Ericka Murillo, Elena Bucaram, Marisol Guayelema, Monica Marmol, Janina Sanchez, Carolina Vergara, Adriana Mena, Junior Velaña, Karla Salazar, Sandra Lara, Elena Chiriboga, Julian Silva, Dalia Gad, Doaa Samy, Menan Ahmed Elsadek, Hanan Mahmoud Mohammed, Mohamed Abouheba, Karim Osamy Ali, Hayssam Rashwan, Omar Moustafa Fawzy, Tarek mohamed Kamel, Rawan Nemer, Mohamed Abada Hassan, Eyad Hassan Falah, Dina Sobhy Abdelhady, Mostafa Zain, Eman Abouzeid Abouzeid Ibrahim, Omar Ossama Elsiraffy, Ahmed Aboelela, Eman mohamed Farag, Ahmed Mohamed Oshiba, Omar Sameh Emam, Alaa Mobarak Attia, Moustafa A. Laymouna, Islam Abdelmonem Ghorab, Mansour Mkayed Mohammed, Nourhan Akram Soliman, Khaled Abd elrahman Ghaly, Kareem Sadek, Mohamed Elsherbiny, Amr Saleh, Hesham Sheir, Tamer Wafa, Mohamed Abd Elmenam, Sherif Abdelmaksoud, Ahmed Reda, Islam Mansour, Mohamed Elzohiri, Basma Waseem, Mohamed Elewaily, Mohammed El-Ghazaly, Ahmad Elhattab, Amr Shalaby, Adham Elsaied, Ahmad Adawy, Mirna Sadek, Mahmoud Abdelfattah Ahmed, Mohamed Omar Herdan, Gena Mohamed Hamed Elassall, Azhar Arabi Mohammed, Mohammed Hamada Takrouney, Tarek Mohamed Essa, Ahmed Mokhtar Mahmoud, Alshaimaa M. Saad, Mariam Albatoul Nageh Fouly, Mahmoud abdelshakour Ibrahim, Mohammad Nageh, Mahmoud M. Saad, Helmy Badr, Mohamed Fayez Fouda, Ahmed Hassan Nofal, Hisham Almohamady, Mohamed Ahmed Arafa, Mohamed Amad, Mohamed Awad Mansour, Jennifer O'Connor, Zachary O'Connor, Nzanzu Anatole, Elysé Nkunzimana, Solomon Machemedze, Lemfuka Dieudonné, William Appeadu-Mensah, Theophilus Teddy Kojo Anyomih, Priscilla Alhassan, Francis A. Abantanga, Vishal Michael, Roshine Mary Koshy, Ankit Raj, Vijay Kumar, Sundeep PT, P Santosh Prabhu, Armin Vosoughi, Ali Farooq Al-Mayoof, Muhamed Jassim Fadhle, Ali Egab Joda, Hayder Nadhim Obaid Algabri, Raed Nael Al-Taher, Sultan S. Abdelhamid, Hashem M. Al-Momani, Marzouq Amarin, Louay Y. Zaghlol, Nijmeh Nasser Alsaadi, Yasmeen Z. Qwaider, Hibah Qutishat, Ahmad Hasan Aliwisat, Esraa Arabiat, Isam Bsisu, Raghad M. Murshidi, Mohammad S. Jabaiti, Ziad A. Bataineh, Husam Aldean Abuhayyeh, Thekraiat M. Al Quran, Faris J. Abu Za'nouneh, Mohanad Mutasem Alebbini, Hamzah Abullah Qudah, Omar Ghazi Hussein, Amir M.I. Murad, Justin Z. Amarin, Haya H. Suradi, Sayel H. Alzraikat, Rand Y. Omari, Bashar M. Matour, Layana Al-Halbouni, Rajai O. Zurikat, Ahmad H. Yanis, Sara Al Hussein, Ali Shoubaki, Waleed H. Ghanem, Kuria David, Soita Wycliffe Chitiavi, Moraa Mose, Robert Mugo, James Ndungu, Timothy Mwai, Swaleh Shahbal, Janan Malik, Nirav Chauhan, Francisa Syovata, Kevin Ochieng, Polycarp Omendo Liyenzero, Syeda Ra'ana Hussain, Stanley Mugambi, Roseline Ochieng, Ebtesam Othman Abdulsalam Elkhazmi, Ala Khaled, Aya Albozidi, Manal Ben Enbaya, Mala Elgammudi, Enas Soula, Wegden ibrahim almabrouk Khalel, Yasmine Ali Elhajjaji, Nouriyah Ali Alwaggaa, Sumayyah Ghayth, Dafer abdulhakim .S. Zreeg, Sara Abobaker Tantush, Fatma Bibas, Tesneem Layas, Randa Alamen M Sharif, Wesal Omar F. Saied Aljadidi, Ahmed Tarek, Hazem Ahmed, Kamila Almabrouk Mohammed Essamilghi, Mabroka Alfoghi, Ma'aly A. Abuhlega, Saddam Arrmali, Fatima Mousa Abduljawad, Hasan Mustafa Alosta, Abdulsalam Abuajaila, Fakereldeen Abdelmutalib, Fatma Bashir, Inas Almengar, Mohammad hasan Annajjar, Abdelaziz Deyab, Fathi Elzowawi, Yousef Krayem, Weam Drah, Asma Meftah, Abobaker Mohammed, Lina Ali Arrmalli, Hajir Aljaboo, Abdallah Elayeb, Mohamed Altomi, Ahmed Altaweel, Mohamed Tumi, Hana Milad Bazozi, Aisha Shaklawoon, Mohammed Meftah Alglaib, Abdullahn Abdousalam Elkaloush, Sara Trainba, Hisham Swessi, Ali Alnaeri, Aya Essam Shnishah, Hamassat Mustufa, Sondas Ali Gargum, Sara Ali Tarniba, Hawa Ahmed Shalluf, Hajer Ali Shokri, Taher L. Sarkaz, Osama Tababa, Ahmed Elhadi, Vesna Cvetanovska Naunova, Laze Jovcheski, Marjan Kamilovski, Aleksandra Gavrilovska-Brzanov, Zarina Abdul Latiff, Siti Farhan Moh Pauzi, Marjmin Osman, Felicia Lim, Ainal Huda Abu Bakar, Azrina SK Zaman, Shareena Ishak, Rufinah Teo, Dr. Tammy Teoh Han Qi, Mohd Yusran Bin Othman, Dato' Dr Zakaria bin Zahari, Zulfitri bin Md Hassan, Cheah Hui Shan, Abhirrami Lechmiannandan, Hafatin Fairos bt Tamaddun, Mohd Fitri Shukri bin Mohamed Adanan, Mohd Yusof bin Abdullah, Wang Junyi, Mohd. Tarmizi Mohd Nor, Wan Ruzaimie Noor, Mohd Razin bin Hassan, Noor Fa'izatul Rahil Ambok Dalek, Hidayah Hayati binti Hashim, Ahmad Zulhisyam bin Zarwawi, V Muthualhagi M Vellusamy, Quah Soong Yuen, Hemasutha a/p Kannessan, Najua binti Ramli, Ahmad Shafiee bin Bujarimin, Jessmine Anntinea, Anthony Dass, Hazlina Mohd. Khalid, Nur Atiqah binti Mohd Hanifah, Keily Wong Yue Jyun, Rahilah binti Abd Razak, Nur Atifah binti Mohd Naim, Siti Nur Aien binti Hamid Hamzah, Cristian R. Zalles Vidal, Eduardo Bracho Blanchet, Roberto Dávila Perez, Emilio Fernandez Portilla, Raúl Villegas Silva, Daniel Ibarra, Antonio Calderon Moore, Cesar Carrasco-Ortega, Monica Noguez Castillo, Dorihela Herappe Mellado, Guillermo Yanowsky Reyes, Luis Fernando Gonzalez Cortez, Rafael Santana Ortiz, Jamie Orozco Perez, Jorge Román Corona C.Rivera, Juan Jose Cardenas Ruiz Velasco, Moises Quiles Corona, Christian Peña Padilla, Lucina Bobadilla Morales, Alfredo Corona Rivera, Izabel Maryalexandra Rios Flores, Cristian Irela Aranda Sánchez, Gabriela Ambriz-González, Nestor Martínez Hernández Magro, Francisco Javier León Frutos, José de Jesús Cárdenas Barón, Alejandro González Ojeda, Jessica Yarza Fernández, Juan Domingo Porras, Pastor Aguirre-Lopez, Vicente Sánchez Paredes, Arturo Montalvo Marin, Jose Manuel Diaz Gomez, Lorenzo Juvencio Caamal, David Bulnes Mendizabal, Pablo Sanchez Valladares, Humberto Garcia Martinez, Opeoluwa Adesanya, Moses Olanrewaju, Rilwan Adegboyega, Nurudeen Abdulraheem, Anuoluwapo Aremo, Florence Dedeke, Anyanwu Lofty-John Chukwuemeka, Mohammad Aminu Mohammad, Abdullahi Lawalbarau, Nwokoro Collins, Ogundele Ibukunolu, Amo Shonubi, Oluwaseun Ladipo-Ajayi, Olumide Abiodun Elebute, Justina Seyi-Olajide, Felix Alakaloko, George Ihediwa, Kayode Olayade, Christopher Bode, Olakayode Ogundoyin, Dare I. Olulana, Ifeanyichukwu Kelvin Egbuchulem, Felix O. Kumolalo, Ikechukwu Ulasi, Uchechukwu Obiora Ezomike, Sebastian Okwuchukwu Ekenze, Elochukwu Perpetua Nwankwo, Emmanuel Ifeanyi Nwangwu, Isaac Chukwu, Christopher Chim Amah, Nene Elsie Obianyo, Omolara Williams, Roland Iheanyichukwu Osuoji, Omolara Moronkeji Faboya, Olalekan Temitope Ajai, Moruf Adekunle Abdulsalam, Titiloye Hannah Agboola, Bolarinwa Bolanle Temilade, Maryrose Osazuwa, Morayo Monsurat Salawu, Eze Chukwuemeka Ejinkeonye, Mariya Mukhtar Yola, Amsa B. Mairami, Adekunle T. Otuneye, Matthias Igoche, Adebayo Gbenga Tanimola, Emmanuel Akinlabi Ajao, Efeturi Agelebe, Samson Olori, Philip Mari Mshelbwala, Olabisi Osagie, Adewale Oyinloye, Auwal M Abubakar, Lateef Oyebanji, Ibrahim Shehu, Cyril Cletus, Ahmed Bamanga, Faruk Suleiman, Sani Adamu, David C.Nwosu, Yahya S.Alkali, Iliya Jalo, Aliu Rasaki, Yusuf T.Sambo, Kalakwa A.Mohammed, Abubakar M.Ballah, Victor Modekwe, Okechukwu Hyginus Ekwunife, Ugochukwu S Ezidiegwu, Andrew N Osuigwe, Jideofor O Ugwu, Chuka A Ugwunne, Nadeem Akhter, Mudassir Fayaz Gondal, Rafee Raza, Ali Raza Chaudary, Hassan Ali, Muhammad Umar Nisar, Muhammad Umer Jamal, Ghuri Shankar Pandit, Uzma Mumtaz, Muhammad Bin Amjad, Nabila Talat, Wajeeh ur Rehman, Muhammad Saleem, Muhammad Bilal Mirza, Imran Hashim, Naveed Haider, Soban Hameed, Ayesha Saleem, Sohail Dogar, Muhammad Sharif, Muhammad Kashif Bashir, Fatima Naumeri, Zarqa Rani, Muath A.M. Baniowda, Basheer Ba'baa', Majd Yousef Mohammed Hassan, Ammar Darwish, Abrar Shaheen Sehwiel, Mohammed Shehada, Abrar ghassan Balousha, Yara Ajrami, Ainaa Ata Mohammad Alzamari, Bashar Yaghi, Hasan Subhi Hasan Abu Al-saleem, Mervat Sufian Abu Farha, Mohammad Omar Mohammad Abdelhafez, Firas Anaya, Asef belal Qadomi, Abd Al-Naser Bany Odi, Muath Abdelrahem Fuad Assi, Fadwa Sharabati, Ahmad Abueideh, Doha mustafa saleh Beshtawi, Hasan Arafat, Lara Zahi Adel Khatatba, Safa' Jamal Abatli, Hiba Al-Tammam, Dania Jaber, Yara Imad Omar Kayed, Ali Abdelhay Abumunshar, Rami Anwar Misk, Asmahan Mohammad Suliman Alzeer, Mutassem Sharabati, Ihsan Ghazzawi, Osama Majed Darras, Mahmoud M.Qabaja, Ma'alem sameer Hajajreh, Yasmeen Ahmad Samarah, Dua Hasan Yaghi, Moradallah Asad Fahmi Qunaibi, Abdelrazzaq Abu Mayaleh, Sharehan Joubeh, Annan Ebeido, Samer Adawi, Ihda Adawi, Mohammad Omar Ibrahim Alqor, Ahmad Samih Arar, Hadeel Awad, Fawzi Abu-Nejmah, Osaid Shaher Shabana, Firas Alqarajeh, Tareq Z. Alzughayyar, Jomana Madieh, Mahmoud Fuad Sbaih, Raghad mohammad abdu Alkareem, Raghad abdullateef Lahlooh, Yasmeen Adly Halabi, Wisam Baker, Tasneem Fathi Hasan Almusleh, Abdulraheem Adnan Abdulraheem Tahyneh, Yazid yousef mahmoud Atatri, Najlaa Abu Jamie, Nasrallah Ashraf Al Massry, Walaa Lubbad, Ayoub A.Nemer, Mohammed Alser, Aya Azmi Shehda Salha, Khaled Alnahhal, Aya Mahmoud Elmzyyen, Amir Talat Sheda Ghabayen, Abdulwhhab Ayman Abu Alamrain, Samar H. Al-Shwaikh, Omar Adly Elshaer, Nureddin Shaheen, Jehad Fares, Hisham Dalloul, Anas Qawwash, Mustafa abu Jayyab, Dina Ayman Ashour, Ahmad Ashraf Shaheen, Samy Rafat Ramadan Naim, Eman Abu Shiha, Nagham Mohammed Al Dammagh, Walaa Almadhoun, Ashraf Ayman Al-Salhi, Abdalkarim Yhya Hammato, Jamal Mohammed Salim, Doaa Khalil Hasanain, Soha Marwan Salem Alwadia, Ismail Nassar, Hala M. Al-Attar, Haya Abdulnasser Ali Alshaikhkhalil, Yasmin Mohammed Khalil Abu Jamie, Yara shareef Ashour, Sharif S. Alijla, Mohamed Anwer El Tallaa, Adham Ashraf Abuattaya, Bisan D.M. Wishah, MOHAMMED A.M. ALDIRAWI, Ahmed S Darwish, Sulaiman T. Alzerei, Nidal Wishah, Sharif Alijla, Isidora Garcia, Marlene Diaz Echegaray, Veronica Raquel Cañapataña Sahuanay, Fernando Trigoso Mori, Jackelyne Alvarado Zelada, Juan Jose Salinas Barreto, Porfirio Rivera Altamirano, Cesar Torres Miranda, Rocio Anicama Elias, Julio Rivera Alvarez, Juan Pedro Vasquez Matos, Fernando Ayque Rosas, Jesmarina Ledesma Peraza, Andrea Gutarra Palomino, Stephany Vega Centen, Victor Casquero, María Rosa Ortiz Argomedo, Francisco Lapouble, Genaro Llap Unchón, Florangel Patricia Delgado Malaga, Luis Ortega Sotelo, Segundo Gamboa Kcomt, Araceli Villalba Villalba, Nancy Rossana Mendoza Leon, Loreley Raquel Cardenas Alva, Maria Susana Loo Neyra, Cathy Lee Alanguia Chipana, Cintya Maria de Jesus Torres Picón, Natalia Huaytalla Quiroz, Danny Dominguez, Carlos Segura Calle, Jenny Arauco, Luis Ormeño Calderón, Ximena Ghilardi Silva, Miriam Daniela Fernandez Wilson, Joan Elizabeth Gutierrez Maldonado, Cesar Diaz Leon, Waldo Berrocal Anaya, Patricia Chavez Galvez, Prince Pamela Aguilar Gargurevich, Flor de Maria Diaz Castañeda, Carmen Guisse, Erika Ramos Paredes, Jose Luis Apaza Leon, Faye Aguilar Aguilar, Raul Ramirez De La Cruz, Lenny Flores Carbajal, Carlos Mendoza Chiroque, Gladys Johana Sulca Cruzado, Natalia Tovar Gutierrez, Jennifer Sotelo Sanchez, Carolina Paz Soldan, Karina Hernández Córdova, Edgar Fernando Delgado Quinteros, Luz Mery Brito Quevedo, Juan Jose Mendoza Oviedo, Angel Samanez Obeso, Patricia Paredes Espinoza, Johann de Guzman, Raisa Yu, Vlad Cosoreanu, Sebastian Ionescu, Aurel Mironescu, Lucian Vida, Adrian Papa, Roxana Verdeata, Bogdan Gavrila, Liviu Muntean, Marija Lukac, Miona Stojanovic, Djordje Toplicic, Milan Slavkovic, Andjelka Slavkovi, Dragoljub Zivanovic, Ana Kostic, Maja Raicevic, Delphine Nkuliza, Daniel Sidler, Corné de Vos, Elmarie vd Merwe, David Tasker, Omar Khamag, Cecilia Rengura, Thozama Siyotula, Uzair Jooma, Dirk von Delft, Marion Arnold, Hansraj Mangray, Shamaman Harilal, Sanele Madziba, Naveen Wijekoon, Tharanga Gamage, Benedict Paul Bright, Alaa Abdulrahman, Ola Ahmed Abdulmjeed Mohammed, Mohammed Salah, Ahmad Elian Abu Ajwa, Mohammed Morjan, Mohammad Mohannad Batal, Vivian Faks, Mohamad Bassel Mouti, Ahmadfateh Assi, Ahmad Al-Mouakeh, Ahmad Sankari Tarabishi, Ziad Aljarad, Aos Alhamid, Jiraporn Khorana, Wannisa Poocharoen, Sirima Liukitithara, Anan Sriniworn, Wasun Nuntasunti, Monawat Ngerncham, Ratiyaporn Phannua, Kanokrat Thaiwatcharamas, Patchareeporn Tanming, Lassaad Sahnoun, Nahla Kchiche, Roua Abdelmoumen, Egemen Eroğlu, Mehmet Ali Ozen, Hatice Sonay Yalçın Cömert, Mustafa İmamoğlu, Haluk Sarıhan, Şebnem Kader, Mehmet Mutlu, Yakup Aslan, Ahmet Beşir, Şükran Geze, Bahanur Çekiç, Ali Yalcinkaya, Kaan Sönmez, Ramazan Karabulut, Zafer Türkyılmaz, Kıvanç Şeref, Merve Altın, Merve Aykut, M.Eren Akan, Melisa Erdem, Ebru Ergenekon, Canan Türkyılmaz, Elif Keleş, Ali Canözer, Aslı Öztürk Yeniay, Elif Eren, İlknur Banlı Cesur, Zerrin Özçelik, Gökmen Kurt, Mustafa Kurthan Mert, Hatice Kaya, Müge Çelik, Suleyman Cuneyt Karakus, Nazile Erturk, Alev Suzen, Nilay Hakan, Fatih Akova, Mehmet Pasaoglu, Shukurali Eshkabilov, Rustam Z. Yuldashev, Dekhkonboev Avazjon Abdunomonovich, Aliev Makhmudjan Muslimovich, Azad Patel, Chisengo Kapihya, Nicholas Ensar, Ramesh M Nataraja, Mithila Sivasubramaniam, Matthew Jones, Warwick Teague, Sharman Tan Tanny, Gordon Thomas, Kiera Roberts, Soundappan Sannappa Venkatraman, Holger Till, Manon Pigeolet, Martine Dassonville, Anas Shikha, Win Sabai Phyu Win, Zahidah Adlynee Haji Ahmad, Léamarie Meloche-Dumas, Louise Caouette-Laberge, Dickens St-Vil, Ann Aspirot, Nelson Piché, Shahrzad Joharifard, Nadia Safa, Jean-Martin Laberge, Sherif Emil, Pramod Puligandla, Kenneth Shaw, Hussein Wissanji, Eileen Duggan, Elena Guadagno, Maria Consuelo Puentes, Paola Osses Leal, Carolina Mendez Benavente, Michal Rygl, Barbora Trojanová, Klára Berková, Tereza Racková, Ladislav Planka, Jan Škvařil, Radek Štichhauer, Shahad Sabti, Alex Macdonald, Nordeen Bouhadiba, Dorothy Kufeji, Caroline Pardy, Simon Mccluney, Alireza Keshtgar, Rebecca Roberts, Hannah Rhodes, Kate Burns, Robin Garrett-Cox, Kat Ford, Hannah Cornwall, Krithi Ravi, Felicity Arthur, Paul Losty, Tony Lander, Ingo Jester, Suren Arul, Oliver Gee, Giampiero Soccorso, Michael Singh, Max Pachl, Benjamin Martin, Afnan Alzubair, Arun Kelay, Jonathan Sutcliffe, Thomas Middleton, Amy Hughes Thomas, Merina Kurian, Fraser Cameron, Jayaram Sivaraj, Mark C Thomas, Dean Rex, Ceri Jones, Kate Bradshaw, Arnaud Bonnard, Xavier Delforge, Camille Duchesne, Caroline Le Gall, Coralie Defert, Samia Laraqui Hossini, Florent Guerin, Géraldine Hery, Virginie Fouquet-Languillat, Jules Kohaut, Aline Broch, Thomas Blanc, Luke Harper, Thomas Delefortrie, Quentin Ballouhey, Laurent Fourcade, Céline Grosos, Benoit Parmentier, Guillaume Levard, Maria Giovanna Grella, Mariette Renaux Petel, Lucie Grynberg, Olivier Abbo, Sofia Mouttalib, Mélodie Juricic, Aurelien Scalabre, Elodie Haraux, Anke Rissmann, Hardy Krause, Peter Goebel, Ludwig Patzer, Udo Rolle, Andrea Schmedding, Alexandra Antunez-Mora, Bernd Tillig, Sylvester von Bismarck, Patricia Reis Barbosa, Christian Knorr, Domitille Stark, Marco Brunero, Luigi Avolio, Francesco Manni, Matilde Molinelli, Marinella Guazzotti, Alessandro Raffaele, Piero Giovanni Romano, Silvia Cavaiuolo, Gian Battista Parigi, Laszlo Juhasz, Anna Rieth, Arunas Strumila, Rūta Dagilytė, Arunas Liubsys, Pranas Gurskas, Dalius Malcius, Agne Mikneviciute, Asta Vinskaite, Vidmantas Barauskas, Liam Vierboom, Timothy Hall, Spencer Beasley, Lucy Goddard, Mark Stringer, Naveen Weeratunga, Stephen Adams, Jitoko Cama, Marilyn Wong, Sridharan Jayaratnam, Askar Kukkady, Udaya Samarakkody, Sylwester Gerus, Dariusz Patkowski, Agnieszka Wolny, Tomasz Koszutski, Szymon Tobor, Marta Osowicka, Piotr Czauderna, Dariusz Wyrzykowski, Hanna Garnier, Stefan Anzelewicz, Osowicka Marta, Agata Knurowska, Alicja Weiszewsk, Andrzej Grabowski, Wojciech Korlacki, Michal Pasierbek, Przemyslaw Wolak, Aneta Piotrowska, Anna Roszkiewicz, Piotr Kalicińsk, Agata Trypens, Grzegorz Kowalewsk, David Sigalet, Amer Alsaied, Mansour Ali, Ameen Alsaggaf, Alaa Ghallab, Yazeed Owiwi, Ali Zeinelabdeen, Mohamed Fayez, Ahmed Atta, Mazen Zidan, Asaad saleh Radwan, Hanin Shalaby, Reem Abdelbaqi, Khalid Alattas, Yar Kano, Omar Sindi, Abdullah Alshehri, Tariq Altokhais, Fahad Alturki, Mohammad Almosaibli, Dasha Krisanova, Wisam Abbas, Hee-Beom Yang, Hyun-Young Kim, Joong Kee Youn, Jae Hee Chung, Seok Hyeon Cho, In ji Hwang, Ju yeon Lee, Eung song Song, Jenny Arboleda, Mercedes Ruiz de Temiño Bravo, Alexander Siles Hinojosa, Miriam García, Isabel Casal Beloy, Detlef Oliu San Miguel, Maria Elena Molina Vazquez, Verónica Alonso, Alberto Sanchez, Oscar Gomez, Isabel Carrillo, Tomas Wester, Carmen Mesas Burgos, Lars Hagander, Martin Salö, Erik Omling, Niclas Rudolfson, Christina Granéli, Helena Arnadóttir, Emma Grottling, Kate Abrahamsson, Vladimir Gatzinsky, Michaela Dellenmark Blom, Daniel Borbonet, Paul Puglia, Vinicio Jimenez Morejon, Gaston Acuna, Mario Moraes, Jonathan Chan, Pavan Brahmamdam, Alan Tom, Karen Sherer, Brandy Gonzales, Aaron Cunningham, Sanjay Krishnaswami, Reto Baertschiger, Mary Leech, Regan Williams, Lauren Camp, Ankush Gosain, Maria Mora, Bailey D. Lyttle, Jeremy Chang, Lydia McColl Makepeace, Kathryn L Fowler, Sara Mansfield, Erica Hodgman, Chukwubinyelum Amaechi, Alana Beres, Mark N. Pernik, Luke J. Dosselman, Murad Almasri, Sunil Jain, Varun Modi, Marianelly Fernandez Ferrer, John Coon, Joann Gonzalez, Medhavi Honhar, Nensi Ruzgar, Griffin Coghill, Sarah Ullrich, Maija Cheung, Katrine Løfberg, Jodie Greenberg, Kate Davenport, Samir Gadepalli, Sarah Fox, Stephanie Johnson, Mercedes Pilkington, April Hamilton, Nicole Lin, Juan Sola, Yang Yao, Jenna Kylene Davis, Monica Langer, Jonathan Vacek, Fizan Abdullah, Julie Khlevner, William Middlesworth, Marc Levitt, Hira Ahmad, Sabina M Siddiqui, Alex Bowder, Terry Derks, Afua Amoabin Amoabin, Brooke Pinar, Frank Owusu-Sekyere, Benmanseur Saousen, Rasika Naidoo, Azra Karamustafic, Danielle Paula de Oliveira, Sarah Bueno Motter, Jerhy Andrade, Antonín Šafus, Jason Langley, Alexandra Wilke, Corazone Deya, Habib Mansour Murtadi, Mindaugas Berzanskis, Nwachukwu Calistus, Olalekan S. Ajiboye, Michael Felix, Osagie O Olabisi, Seçil Erçin, Teymursha Muradi, Stephen S. Burks, Sergio Lerma, Jillian Jacobson, Calin Calancea, Rafael Valerio-Vazquez, Guigui Sikwete, Owusu Sekyere, Akhona Mbonisweni, Shahnoor Syed, Cho Seok Hyeon, Fatemeh Pajouhandeh, Sheba Mary Pognaa Kunfah

## Abstract

**Background:**

Congenital anomalies are the fifth leading cause of mortality in children younger than 5 years globally. Many gastrointestinal congenital anomalies are fatal without timely access to neonatal surgical care, but few studies have been done on these conditions in low-income and middle-income countries (LMICs). We compared outcomes of the seven most common gastrointestinal congenital anomalies in low-income, middle-income, and high-income countries globally, and identified factors associated with mortality.

**Methods:**

We did a multicentre, international prospective cohort study of patients younger than 16 years, presenting to hospital for the first time with oesophageal atresia, congenital diaphragmatic hernia, intestinal atresia, gastroschisis, exomphalos, anorectal malformation, and Hirschsprung's disease. Recruitment was of consecutive patients for a minimum of 1 month between October, 2018, and April, 2019. We collected data on patient demographics, clinical status, interventions, and outcomes using the REDCap platform. Patients were followed up for 30 days after primary intervention, or 30 days after admission if they did not receive an intervention. The primary outcome was all-cause, in-hospital mortality for all conditions combined and each condition individually, stratified by country income status. We did a complete case analysis.

**Findings:**

We included 3849 patients with 3975 study conditions (560 with oesophageal atresia, 448 with congenital diaphragmatic hernia, 681 with intestinal atresia, 453 with gastroschisis, 325 with exomphalos, 991 with anorectal malformation, and 517 with Hirschsprung's disease) from 264 hospitals (89 in high-income countries, 166 in middle-income countries, and nine in low-income countries) in 74 countries. Of the 3849 patients, 2231 (58·0%) were male. Median gestational age at birth was 38 weeks (IQR 36–39) and median bodyweight at presentation was 2·8 kg (2·3–3·3). Mortality among all patients was 37 (39·8%) of 93 in low-income countries, 583 (20·4%) of 2860 in middle-income countries, and 50 (5·6%) of 896 in high-income countries (p<0·0001 between all country income groups). Gastroschisis had the greatest difference in mortality between country income strata (nine [90·0%] of ten in low-income countries, 97 [31·9%] of 304 in middle-income countries, and two [1·4%] of 139 in high-income countries; p≤0·0001 between all country income groups). Factors significantly associated with higher mortality for all patients combined included country income status (low-income *vs* high-income countries, risk ratio 2·78 [95% CI 1·88–4·11], p<0·0001; middle-income *vs* high-income countries, 2·11 [1·59–2·79], p<0·0001), sepsis at presentation (1·20 [1·04–1·40], p=0·016), higher American Society of Anesthesiologists (ASA) score at primary intervention (ASA 4–5 *vs* ASA 1–2, 1·82 [1·40–2·35], p<0·0001; ASA 3 *vs* ASA 1–2, 1·58, [1·30–1·92], p<0·0001]), surgical safety checklist not used (1·39 [1·02–1·90], p=0·035), and ventilation or parenteral nutrition unavailable when needed (ventilation 1·96, [1·41–2·71], p=0·0001; parenteral nutrition 1·35, [1·05–1·74], p=0·018). Administration of parenteral nutrition (0·61, [0·47–0·79], p=0·0002) and use of a peripherally inserted central catheter (0·65 [0·50–0·86], p=0·0024) or percutaneous central line (0·69 [0·48–1·00], p=0·049) were associated with lower mortality.

**Interpretation:**

Unacceptable differences in mortality exist for gastrointestinal congenital anomalies between low-income, middle-income, and high-income countries. Improving access to quality neonatal surgical care in LMICs will be vital to achieve Sustainable Development Goal 3.2 of ending preventable deaths in neonates and children younger than 5 years by 2030.

**Funding:**

Wellcome Trust.

## Introduction

In the past 30 years, major strides have been made in reducing childhood mortality globally, with a decrease in deaths in children younger than 5 years from 12·6 million in 1990 to 5·2 million in 2019.^1^, ^2^ However, neonatal mortality has fallen at a slower rate, from 4·7 million deaths in 1990 to 2·4 million in 2019.^1^, ^2^ Consequently, the proportion of deaths in children younger than 5 years occurring in the neonatal period has risen from 37% in 1990 to 46% in 2019.^2^ As the number of deaths from infectious diseases has decreased, the proportion of deaths attributed to congenital anomalies (birth defects) has concurrently increased, accounting for an estimated 303 000 neonatal deaths and half a million deaths in children younger than 5 years annually.^3^, ^4^, ^5^ Congenital anomalies are now the fifth leading cause of mortality in children younger than 5 years and the 11th leading cause of years of life lost for the global population.^6^, ^7^

**Research in context**

**Evidence before this study**

We searched PubMed, Embase, and the Cochrane Central Register of Controlled Trials for observational or randomised studies published in English from Jan 1, 2000, to Oct 10, 2020. Three search strings were used: the seven gastrointestinal congenital anomalies included in our study; all-cause in-hospital or 30-day postoperative mortality; and patients aged under 16 years. Studies were limited to those of primary surgical intervention and cohorts of more than 100 patients. We found no previous studies that have prospectively compared outcomes from gastrointestinal congenital anomalies between low-income, middle-income, and high-income countries globally. Research on the individual conditions was mainly from high-income countries (79 studies), with a smaller number of studies from middle-income countries (14 studies), and one from a low-income country. Due to the heterogeneity of the studies, an accurate comparison of outcomes between income strata was not possible. Information regarding leading causes of death or factors associated with mortality for these conditions in low-income and middle-income countries (LMICs) is scarce.

**Added value of this study**

This study provides validated, prospectively collected data on patients with gastrointestinal congenital anomalies in 74 low-income, middle-income, and high-income countries across the globe. The results highlight large disparities in mortality between income settings. Moreover, the high mortality rates identified for these conditions in LMICs far exceed surgical mortality rates among older children and adults reported in previous international surgical outcomes studies. The large study cohort has enabled robust multivariable analysis and identification of numerous factors substantially and significantly associated with mortality. These results, along with the detailed data on patient management in each setting, provide a foundation from which interventions, guidelines, and policies can be established with the aim of reducing the vast inequities in care provision and outcomes that currently exist.

**Implications of all the available evidence**

Sustainable Development Goal 3.2, which is to end preventable deaths of neonates and children under 5 years of age by 2030, is unachievable without an urgent focus on improving access to quality neonatal surgical care in LMICs. Indicators of clinical deterioration before surgical intervention were significantly associated with higher mortality for all conditions. Birth at a paediatric surgery centre (enabled by antenatal diagnosis) can help to prevent this and reduce mortality, as shown in patients with gastroschisis and congenital diaphragmatic hernia. However, most patients present from district hospitals, highlighting the importance of improved diagnosis, resuscitation, and timely transfer at this level. At paediatric surgery centres, improved provision of basic neonatal intensive care facilities, including ventilation, parenteral nutrition, and central intravenous access, for neonates could reduce mortality further. These interventions would also benefit sick neonates more broadly, and therefore would help to further reduce global neonatal mortality.

Congenital anomalies are defined by WHO as structural or functional anomalies that occur during intrauterine life.^4^ They affect 3–6% of global live births.^4^ Low-income and middle-income countries (LMICs) have the highest prevalence due to greater maternal exposure to micronutrient deficiencies, teratogens, and intrauterine infections, and lower termination rates resulting from limited antenatal diagnosis.^4^, ^8^ LMICs are estimated to account for more than 95% of congenital anomaly deaths, two-thirds of which could be prevented through surgical care. However, these estimates are based on sparse data.^5^

Data on congenital anomaly outcomes and associated factors in LMICs are limited due to a lack of congenital anomaly registries, research, and inclusion of these conditions within national health surveys.^9^, ^10^ Through international charitable organisations, data have been collected on some congenital anomalies, including cleft lip and palate, club foot, neural tube defects, and congenital heart disease.^11^, ^12^, ^13^, ^14^ However, gastrointestinal congenital anomalies, which are also very prevalent, have received little attention. These anomalies, which are often fatal without access to emergency neonatal surgical care, could contribute to a large proportion of the preventable congenital anomaly deaths in LMICs.

Sustainable Development Goal 3.2 aims to “end preventable deaths of newborns and children under 5 years of age”.^15^ Therefore, preventable deaths from gastrointestinal congenital anomalies need to be identified and quantified globally, and insight must be gained into how to improve survival. The aim of this study was to prospectively compare the outcomes of the seven most common gastrointestinal congenital anomalies in low-income, middle-income, and high-income countries, and to identify factors associated with mortality.

## Methods

### Study design and participants

We did a global, multicentre, international, prospective cohort study of patients presenting to hospital for surgical care with seven gastrointestinal congenital anomalies (oesophageal atresia, congenital diaphragmatic hernia, intestinal atresia, gastroschisis, exomphalos [also known as omphalocele], anorectal malformation, and Hirschsprung's disease). STROBE guidelines were followed for this study.

Data were collected by patients' health-care providers, including a consultant or senior physician with overall clinical responsibility, who also oversaw patient recruitment, data completeness, and accuracy. We aimed to recruit as many participating hospitals as possible from across the world. Local investigators were invited to participate through international conference presentations, professional organisations, social media, and via a network of national and regional study leads. Participation was voluntary; no payment was made for data collection. Hospital teams chose one calendar month (commencing on the first day of the month) or multiple 1-month study periods (depending on local capacity), between October, 2018, and April, 2019, inclusive, to recruit consecutive patients to the study (by date of presentation).

Patients included any child younger than 16 years presenting acutely, for the first time, with one or more of the study conditions, and who received primary surgical intervention, conservative treatment, or palliative care. Patients were excluded if they had previously had surgery for their condition, were returning with a postoperative complication, were presenting electively, or were being transferred elsewhere for surgical intervention.

This study was classified as a clinical audit, with written confirmation from King's College London Ethics Committee that it, therefore, does not require ethical approval. All participating centres gained local study approval to participate according to their institutional ethical regulations. Consent forms were completed by all patients in hospitals requiring them. Data transfer agreements were legally signed between institutions where required.

### Procedures

The study protocol, data collection forms, and all supporting documentation were produced in 12 languages.^16^ Anonymous, de-identified data were collected using the secure online platform REDCap.^17^ A pilot study to optimise data collection procedures was done in 16 hospitals (in 13 countries). Variables were chosen based on published core outcome sets and commonly collected outcomes in systematic reviews from high-income countries, as well as important variables identified in LMIC literature.^18^

Generic variables collected for all patients included: demographics, antenatal care (maternal ultrasound) and diagnosis, delivery type (vaginal or caesarean section), transportation (ambulance, patient's own, or born at study hospital), referral site if applicable (district hospital, community clinic, home, or other), clinical condition on arrival (sepsis, hypovolaemia, or hypothermia), resuscitation on arrival (antibiotics, intravenous fluid, or warming), clinical condition at surgery (American Society of Anesthesiologists [ASA] score), intraoperative care (surgical safety checklist used, anaesthetist and surgeon grade or position, and anaesthetic administered), perioperative care (ventilation, intravenous access, parenteral nutrition, blood transfusion, and antibiotics), and outcomes (detailed later). Condition-specific variables included: condition type or classification, surgical intervention, and complications. Patients were followed up until 30 days after the primary intervention, or 30 days after admission in patients who did not receive an intervention. The presence and type of follow-up was collected for patients discharged before 30 days.

Clear definitions are provided for all variables in the published protocol.^18^ Internationally utilised and validated definitions were used where available. Cause of death was decided by the clinical team using 16 predetermined categories and one free-text category. From the free-text responses, one additional category was added (syndrome incompatible with life). Participating country name was collected, and World Bank 2018 country income status classification was used to categorise countries as low-income, middle-income, or high-income.^19^

Data validation was done in 10% of randomly selected participating hospitals with use of an independent validating local investigator, who retrospectively collected a selection of the data again for a 1-month study period. The validation data collected included the number of eligible patients, generic variables (month of presentation, study condition, sex, unplanned interventions, and survival to discharge), and condition-specific variables (condition type and surgical intervention). All local investigators at validation hospitals completed a data accuracy questionnaire to help identify potential errors and aid data interpretation.

### Outcomes

The primary outcome was all-cause, in-hospital mortality for all conditions combined and each condition individually, stratified by country income (low-income, middle-income, or high-income). Patients were categorised as alive if they were either discharged alive or were still in hospital 30 days after primary intervention or 30 days after admission for patients who did not receive an intervention. Patients were categorised as dead if they died in hospital within 30 days of the primary intervention or 30 days after admission for patients who did not receive an intervention.

Secondary outcomes were the presence of one or more of surgical site infection, wound dehiscence, or a need for unplanned reintervention, within 30 days of surgery, and 30-day post-primary intervention mortality. Length of hospital stay was recorded for all patients (including admission and discharge day, up to a 30-day maximum). Cause of death was an exploratory outcome.

The study aimed to test our hypothesis that there is a significant difference in mortality from the seven most common gastrointestinal congenital anomalies between low-income, middle-income, and high-income countries globally.

### Statistical analysis

A sample size calculation was done using Bonferroni correction for multiple testing, assuming 80% power and an overall type 1 error of 5% (appendix p 13). To determine a significant difference in mortality between high-income countries and LMICs, the minimum sample size per country income group was estimated to be 21 for oesophageal atresia, 63 for congenital diaphragmatic hernia, 24 for intestinal atresia, 15 for gastroschisis, 115 for exomphalos, 85 for anorectal malformation, and 79 for Hirschsprung's disease (804 patients in total). A comparison of mortality between high-income countries, middle-income countries, and low-income countries was planned if a sufficient cohort was collected.

We did a complete case analysis. Duplicate entries were identified and excluded. Patients missing the study condition or primary outcome were excluded. If more than 20% of patients were missing the primary outcome in any given month at a participating hospital, all patients in that month were excluded. Data are presented as means with SDs if normally distributed and medians with IQRs if skewed; count data are presented as numbers and percentages. Data are summarised for all patients and by country income status. We calculated differences in patient demographics, care received, and primary and secondary outcomes, between country income strata using χ^2^ analysis or Fisher's exact test if a group had less than five patients. p<0·05 was deemed statistically significant. Mortality is presented by country income status for all patients and for each condition separately, with 95% CIs (calculated using the Wald CI for a proportion formula when n>5 or exact binomial confidence intervals when n≤5).

Continuous variables were used as collected (ie, they were not categorised). Categorical variables were collapsed to include at least 15 patients per group if clinically and statistically appropriate (appendix pp 47–62). We combined hypovolaemia, hypothermia, or both on admission into one variable due to collinearity.

Three multilevel, multivariable models were used to identify factors associated with mortality in all study patients (including income status as a covariable), and in LMIC and high-income country settings separately. All models excluded duration of hospital stay due to missing data (n=308) and variable subgroups (time to primary intervention; and time to first and full enteral feeding, and antibiotic duration, following primary intervention). The models containing all patients and those from LMICs included all other generic variables. Three additional variables from the high-income countries model were excluded due to low or no patients in a group: anaesthetic type, surgeon grade or position, and wound dehiscence (appendix pp 47–48). All variables included within the models had a maximum of 0·2% missing data (appendix pp 47–62). In the multivariable models, patients with missing data for one or more entries were excluded. Through the use of dummy variables that indicate when a data point is missing, we tested and concluded that the small amount of missing data did not affect the multivariable outcomes. There were no significant differences in the mortality between the patients included in the models and the small groups that were excluded due to missing data. Similarly, there were no significant differences in the proportion of patients from high-income, middle-income, and low-income countries in the patients included in the models and the small groups excluded due to missing data. Therefore, missing data imputation was not done.

All models were adjusted for hospital-level clustering and included potential confounders (gestational age at birth, bodyweight and age at presentation, presence of additional anomalies, and ASA score at primary intervention) and effect modifiers (receipt of ventilation, central intravenous access, and parenteral nutrition). Patients who had no surgical intervention, and therefore had no data on ASA score, anaesthetic, anaesthetist, surgeon, surgical safety checklist, or secondary outcome complications were included in the models (categorised as not applicable within each variable) to avoid bias, because these patients were either palliated or well enough to be managed without emergency intervention. We used penalised Lasso regression to determine the risk ratio (RR; 95% CI, p value) of mortality for each variable within the models. This method was chosen over the originally planned logistic regression with backwards stepwise elimination to enable more variables to be included in the models, with greater robustness. Our large cohort size made this technique feasible.

Exploratory penalised Lasso regression analyses were done for each condition separately, with income status as a covariable, adjustments for hospital-level clustering, and with the aforementioned confounders and effect modifiers included. Models included both generic and condition-specific variables. Variables excluded due to no or low counts are detailed in the appendix (pp 49–62). All multivariable results are presented as forest plots.

We compared the validation data with the original study data collected using a weighted κ statistic to determine level of agreement; observed agreement was also reported. We analysed the data using STATA 15.

The study protocol was registered with ClinicalTrials.gov, NCT03666767, and was published previously.^18^

### Role of the funding source

The funder had no role in the study design, data collection, data analysis, data interpretation, or writing of the report.

## Results

**Figure 1:**
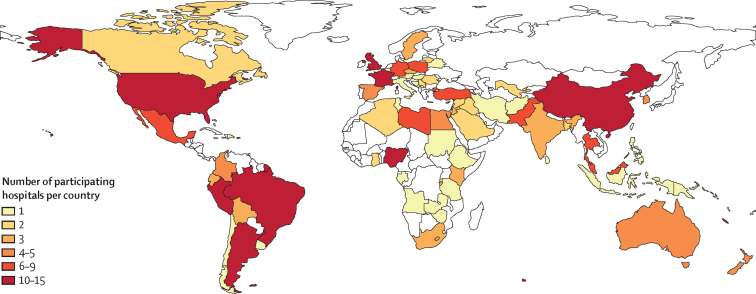
Global distribution of participating hospitals

**Figure 2:**
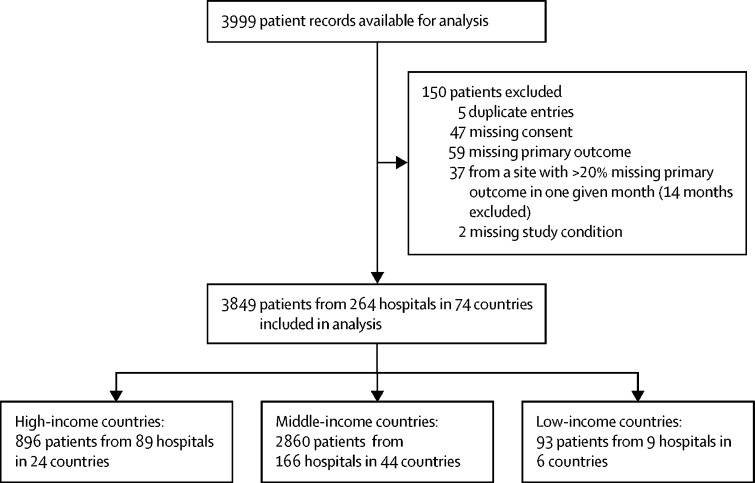
Flow diagram of patient inclusion in the study

We included 3849 patients with 3975 study conditions from 264 hospitals in 74 countries (Figure 1, Figure 2) over 962 1-month study periods (median 3 months per hospital [IQR 2–5]). Of the 3849 patients, 2231 (58·0%) were male (table 1). Median gestational age at birth was 38 weeks (36–39) and median bodyweight at presentation was 2·8 kg (2·3–3·3); both characteristics were similar across income groups. Similar proportions of patients presented with oesophageal atresia, intestinal atresia, exomphalos, and Hirschsprung's disease across all income settings, but significantly fewer patients presented with congenital diaphragmatic hernia and gastroschisis in LMICs compared with high-income countries, and significantly more presented with anorectal malformation. Fewer patients in low-income countries (n=24, 25·8%) had an additional anomaly diagnosed compared with middle-income countries (n=1306, 45·7%) and high-income countries (n=448, 50·0%).

**Table 1:** Patient characteristics

		**Total (n=3849)**	**High-income countries (n=896)**	**Middle-income countries (n=2860)**	**Low-income countries (n=93)**	p value*
Sex						
	Male	2231 (58·0%)	528 (58·9%)	1655 (57·9%)	48 (51·6%)	0·39
	Female	1596 (41·5%)	367 (41·0%)	1185 (41·4%)	44 (47·3%)	..
	Ambiguous genitalia	21 (0·5%)	1 (0·1%)	19 (0·7%)	1 (1·1%)	..
	Unknown	1 (<0·1%)	0	1 (<0·1%)	0	..
Gestational age at birth, weeks		38 (36–39)	38 (36–39)	38 (36–39)	37 (36–39)	0·76
Bodyweight at presentation, kg		2·8 (2·3–3·3)	2·9 (2·4–3·4)	2·8 (2·3–3·3)	2·8 (2·2–3·5)	0·13
Study condition						
	Oesophageal atresia	560 (14·5%)	141 (15·7%)	412 (14·4%)	7 (7·5%)	0·093
	Congenital diaphragmatic hernia	448 (11·6%)	148 (16·5%)	299 (10·5%)	1 (1·1%)	<0·0001
	Intestinal atresia	681 (17·7%)	152 (17·0%)	509 (17·8%)	20 (21·5%)	0·53
	Gastroschisis	453 (11·8%)	139 (15·5%)	304 (10·6%)	10 (10·8%)	0·0004
	Exomphalos	325 (8·4%)	70 (7·8%)	241 (8·4%)	14 (15·1%)	0·057
	Anorectal malformation	991 (25·7%)	178 (19·9%)	788 (27·6%)	25 (26·9%)	0·0003
	Hirschsprung's disease	517 (13·4%)	107 (11·9%)	393 (13·7%)	17 (18·3%)	0·15
Additional anomaly or study condition diagnosed		1778 (46·2%)	448 (50·0%)	1306 (45·7%)	24 (25·8%)	<0·0001
Age at presentation, h		22 (1–85)	3 (0–28)	24 (3–96)	72 (16–192)	0·0001
Distance from patient's home to study hospital, km		25 (2–100)	11 (0–64)	30 (5–110)	55 (10–133)	<0·0001
Sepsis status on arrival to study centre						
	Yes	660 (17·1%)	38 (4·2%)	598 (20·9%)	24 (25·8%)	<0·0001
	Missing	3 (0·1%)	1 (0·1%)	2 (0·1%)	0 (0·0%)	..
Hypovolaemia status on arrival to study centre						
	Yes	564 (14·7%)	75 (8·4%)	478 (16·7%)	11 (11·8%)	<0·0001
	Missing	4 (0·1%)	1 (0·1%)	2 (0·1%)	1 (1·1%)	..
Hypothermia status on arrival to study centre						
	Yes	403 (10·5%)	32 (3·6%)	358 (12·5%)	13 (14·0%)	<0·0001
	Missing	6 (0·2%)	1 (0·1%)	4 (0·1%)	1 (1·1%)	..
ASA score at time of primary intervention						
	1 (healthy person)	678 (17·6%)	115 (12·8%)	534 (18·7%)	29 (31·2%)	<0·0001
	2 (mild systemic disease)	1195 (31·0%)	260 (29·0%)	914 (32·0%)	21 (22·6%)	..
	3 (severe systemic disease)	1046 (27·2%)	316 (35·3%)	717 (25·1%)	13 (14·0%)	..
	4 (severe systemic disease that is a constant threat to life	375 (9·7%)	122 (13·6%)	249 (8·7%)	4 (4·3%)	..
	5 (moribund patient who is not expected to survive without the operation)	151 (3·9%)	15 (1·7%)	136 (4·8%)	0	..
	Not applicable (no surgical intervention)†	395 (10·3%)	62 (6·9%)	307 (10·7%)	26 (28·0%)	..
	Missing	9 (0·2%)	6 (0·7%)	3 (0·1%)	0	..

Data are n (%) or median (IQR). ASA=American Society of Anesthesiologists.

* p values represent univariable testing between country income strata.

† These patients were either palliated, managed conservatively, or discharged without intervention with planned future intervention (appendix pp 16–44).

Median age at presentation was 72 h (IQR 16–192) in low-income countries, 24 h (3–96) in middle-income countries, and 3 h (0–28) in high-income countries. Neonates accounted for 90% (n=3464) of the study participants at presentation; the other 10% ranged from 29 days to 15·8 years of age. Patients travelled further from home to the study hospital in low-income countries (median distance of 55 km [10–133]) compared with middle-income countries (30 km [5–110]) and high-income countries (11 km [0–64]). Higher proportions of patients presented with sepsis, hypovolaemia, and hypothermia in low-income countries and middle-income countries compared with high-income countries. A higher proportion of patients did not receive a surgical intervention in low-income countries (n=26, 28·0%) compared with middle-income countries (n=307, 10·7%) and high-income countries (n=62, 6·9%); consequently, these patients did not have an ASA score. Among patients who received an intervention, an ASA score of 1 was most prevalent in low-income countries, an ASA score of 2 was most prevalent in middle-income countries, and an ASA score of 3 was most prevalent in high-income countries.

Nine (9·7%) of 93 patients had their condition diagnosed or a problem identified antenatally in low-income countries compared with 823 (28·8%) of 2860 in middle-income countries and 506 (56·5%) of 896 in high-income countries (table 2). In low-income countries, most patients (n=75, 80·7%) were born via vaginal delivery and few (n=15, 16·1%) via caesarean section. By contrast, 1421 (49·7%) patients in middle-income countries and 411 (45·8%) in high-income countries were born via caesarean section. Only two (2·2%) patients from low-income countries were born at the paediatric surgery centre, compared with 618 (21·6%) in middle-income countries and 391 (43·6%) in high-income countries. In all settings, the majority of outborn patients (born outside the paediatric surgery centre) presented from district hospitals. In low-income countries, 41 (45·1%) patients travelled to the study centre using non-hospital transport, compared with 1041 (46·4%) in middle-income countries and 74 (14·7%) in high-income countries.

**Table 2:** Care received by patients

			**Total (n=3849)**	**High-income countries (n=896)**	**Middle-income countries (n=2860)**	**Low-income countries (n=93)**	p value*
**Antenatal care, delivery, transportation to the paediatric surgery centre, and referral site**							
Antenatal ultrasound							
	Yes, study condition diagnosed		881 (22·9%)	368 (41·1%)	512 (17·9%)	1 (1·1%)	<0·0001
	Yes, problem identified but study condition not diagnosed		457 (11·9%)	138 (15·4%)	311 (10·9%)	8 (8·6%)	..
	Yes, no problem identified		1945 (50·5%)	343 (38·3%)	1551 (54·2%)	51 (54·8%)	..
	No		558 (14·5%)	44 (4·9%)	482 (16·9%)	32 (34·4%)	..
	Missing		8 (0·2%)	3 (0·3%)	4 (0·1%)	1 (1·1%)	..
Gestational age of study condition diagnosis if antenatal, weeks			25 (20–31)	21 (16–27)	28 (21–32)	..	0·0002
Type of delivery							
	Vaginal (spontaneous)		1767 (45·9%)	373 (41·6%)	1324 (46·3%)	70 (75·3%)	<0·0001
	Vaginal (induced)		194 (5·0%)	97 (10·8%)	92 (3·2%)	5 (5·4%)	..
	Caesarean section (elective)		1022 (26·6%)	185 (20·6%)	830 (29·0%)	7 (7·5%)	..
	Caesarean section (urgent or non-elective)		825 (21·4%)	226 (25·2%)	591 (20·7%)	8 (8·6%)	..
	Unknown		37 (1·0%)	14 (1·6%)	21 (0·7%)	2 (2·2%)	..
	Missing		4 (0·1%)	1 (0·1%)	2 (0·1%)	1 (1·1%)	..
Born at the study hospital							
	Yes		1011 (26·3%)	391 (43·6%)	618 (21·6%)	2 (2·2%)	<0·0001
	Missing		5 (0·1%)	1 (0·1%)	4 (0·1%)	0 (0·0%)	..
Mode of transport to hospital if born elsewhere							
	Ambulance or other transport provided by the health service		1677 (59·1%)	430 (85·1%)	1197 (53·4%)	50 (54·9%)	<0·0001
	Patient's own transport		1156 (40·7%)	74 (14·7%)	1041 (46·4%)	41 (45·1%)	..
	Missing		5 (0·2%)	1 (0·2%)	4 (0·2%)	0	..
Location from which patient presented if born elsewhere							
	District hospital		1835 (64·7%)	401 (79·4%)	1377 (61·4%)	57 (62·6%)	<0·0001
	Home		504 (17·8%)	51 (10·1%)	445 (19·8%)	8 (8·8%)	..
	Community clinic or general practice		446 (15·7%)	44 (8·7%)	379 (16·9%)	23 (25·3%)	..
	From another country		7 (0·2%)	3 (0·6%)	4 (0·2%)	0	..
	From a different specialty within the hospital		5 (0·2%)	4 (0·8%)	0	1 (1·1%)	..
	Unknown		33 (1·2%)	1 (0·2%)	30 (1·3%)	2 (2·2%)	..
	Missing		8 (0·3%)	1 (0·2%)	7 (0·3%)	0	..
**Care at the paediatric surgery centre**							
Resuscitation on arrival							
	Administration of appropriate antibiotics if septic						
		n	660	38	598	24	..
		Yes, within 1 h of arrival	500 (75·8%)	31 (81·6%)	454 (75·9%)	15 (62·5%)	0·42
		Yes, within the first day of arrival	150 (22·7%)	7 (18·4%)	135 (22·6%)	8 (33·3%)	..
		No	10 (1·5%)	0	9 (1·5%)	1 (4·2%)	..
	Administration of intravenous fluid if hypovolaemic						
		n	564	75	478	11	..
		Yes, within 1 h of arrival	440 (78·0%)	40 (53·3%)	394 (82·4%)	6 (54·5%)	<0·0001
		Yes, within the first day of arrival	104 (18·4%)	24 (32·0%)	76 (15·9%)	4 (36·4%)	..
		No	19 (3·4%)	10 (13·3%)	8 (1·7%)	1 (9·1%)	..
		Missing	1 (0·2%)	1 (1·3%)	0	0	..
	Quantity of intravenous fluid given if hypovolaemic						
		n	564	75	478	11	..
		10–20 mL/kg	408 (72·3%)	36 (48·0%)	363 (75·9%)	9 (81·8%)	<0·0001
		>20 mL/kg	135 (23·9%)	28 (37·3%)	106 (22·2%)	1 (9·1%)	..
		Missing	21 (3·7%)	11 (14·7%)	9 (1·9%)	1 (9·1%)	..
	Warming of patient to within normal range on arrival if hypothermic						
		n	403	32	358	13	..
		Yes	371 (92·1%)	28 (87·5%)	330 (92·2%)	13 (100·0%)	0·35
Primary intervention							
	Time from arrival at study hospital to primary intervention, h		24 (7–66)	22 (5–48)	24 (8–72)	34 (10–96)	0·0001
	Type of anaesthesia used for primary intervention						
		General anaesthesia with endotracheal tube or laryngeal airway	3154 (81·9%)	772 (86·2%)	2327 (81·3%)	55 (59·1%)	<0·0001
		Intervention without anaesthesia and with or without analgesia	248 (6·4%)	67 (7·5%)	178 (6·2%)	3 (3·2%)	..
		Local anaesthesia only	25 (0·6%)	1 (0·1%)	24 (0·8%)	0	..
		Spinal or caudal anaesthesia	19 (0·5%)	0	19 (0·7%)	0	..
		Ketamine anaesthesia	9 (0·2%)	1 (0·1%)	5 (0·2%)	3 (3·2%)	..
		Not applicable (no surgery or primary intervention)	392 (10·2%)	55 (6·1%)	305 (10·7%)	32 (34·4%)	..
		Missing	2 (0·1%)	0	2 (0·1%)	0	..
	Person delivering anaesthetic for primary intervention						
		Anaesthetic doctor	3115 (80·9%)	741 (82·7%)	2336 (81·7%)	38 (40·9%)	<0·0001
		Medical officer, surgeon, or other health-care professional	86 (2·3%)	42 (4·7%)	41 (1·5%)	3 (3·2%)	..
		Anaesthetic nurse	35 (0·9%)	1 (0·1%)	17 (0·6%)	17 (18·3%)	..
		No anaesthetic	610 (15·8%)	112 (12·5%)	463 (16·2%)	35 (37·6%)	..
		Missing	3 (0·1%)	0	3 (0·1%)	0	..
	Person delivering primary intervention						
		Paediatric surgeon (or junior with paediatric surgeon assisting or in the room)	3345 (86·9%)	825 (92·1%)	2474 (86·5%)	46 (49·5%)	<0·0001
		Junior doctor or other (without a paediatric or general surgeon assisting or in the room)	59 (1·5%)	7 (0·8%)	49 (1·7%)	3 (3·2%)	..
		Trainee surgeon (without a paediatric or general surgeon assisting or in the room)	49 (1·3%)	7 (0·8%)	36 (1·3%)	6 (6·5%)	..
		General surgeon (or junior with general surgeon assisting or in the room)	32 (0·8%)	7 (0·8%)	18 (0·6%)	7 (7·5%)	..
		Not applicable (no surgery or primary intervention)	361 (9·4%)	49 (5·5%)	281 (9·8%)	31 (33·3%)	..
		Missing	3 (0·1%)	1 (0·1%)	2 (0·1%)	0	..
	Surgical safety checklist used at the time of primary intervention						
		Yes	2569 (66·7%)	747 (83·4%)	1791 (62·6%)	31 (33·3%)	<0·0001
		No	693 (18·0%)	39 (4·4%)	626 (21·9%)	28 (30·1%)	..
		Not applicable (no surgical intervention)	584 (15·1%)	109 (12·1%)	441 (15·4%)	34 (36·5%)	..
		Missing	3 (0·1%)	1 (0·1%)	2 (0·1%)	0	..
Perioperative care							
	Patient received central venous access						
		Yes, peripherally inserted central catheter	1120 (29·1%)	436 (48·7%)	678 (23·7%)	6 (6·5%)	<0·0001
		Yes, percutaneously inserted central line	415 (10·8%)	187 (20·9%)	228 (8·0%)	0	<0·0001
		Yes, umbilical catheter	402 (10·4%)	153 (17·1%)	249 (8·7%)	0	<0·0001
		Yes, surgically placed central line (open insertion)	254 (6·6%)	27 (3·0%)	227 (7·9%)	0	<0·0001
		No	1910 (49·6%)	226 (25·2%)	1597 (55·8%)	87 (93·5%)	<0·0001
Total duration of antibiotics after primary intervention, days			7 (3–11)	3 (1–7)	7 (3–13)	3 (0–7)	0·0001
Blood transfusion							
	Not required		2448 (63·6%)	671 (74·9%)	1708 (59·7%)	69 (74·2%)	<0·0001
	Yes		1348 (35·0%)	213 (23·8%)	1114 (38·9%)	21 (22·6%)	..
	Required but not available		47 (1·2%)	9 (1·0%)	35 (1·2%)	3 (3·2%)	..
	Missing		6 (0·1%)	3 (0·3%)	3 (0·1%)	0	..
	Ventilation						
		No	1755 (45·6%)	258 (28·8%)	1422 (49·7%)	75 (80·6%)	<0·0001
		Yes	2008 (52·2%)	637 (71·1%)	1363 (47·7%)	8 (8·6%)	..
		Required but not available	85 (2·2%)	1 (0·1%)	74 (2·6%)	10 (10·8%)	..
		Missing	1 (<0·1%)	0	1 (<0·1%)	0	..
	Duration of ventilation if given, days		4 (2–8)	4 (2–9)	4 (2–8)	2 (1–3)	0·0025
	Time to first enteral feed post-primary intervention, days		4 (2–8)	4 (2–8)	4 (2–8)	1 (1–3)	<0·0001
	Time to full enteral feeds post-primary intervention, days		8 (4–16)	11 (6–22)	7 (3–15)	3 (2–7)	<0·0001
	Parenteral nutrition						
		No	1476 (38·3%)	212 (23·7%)	1196 (41·8%)	68 (73·1%)	<0·0001
		Yes	2102 (54·6%)	683 (76·2%)	1416 (49·5%)	3 (3·2%)	..
		Yes, but less was available than required	143 (3·7%)	0	143 (5·0%)	0	..
		Required but not available	125 (3·2%)	0	103 (3·6%)	22 (23·7%)	..
		Missing	3 (0·1%)	1 (0·1%)	2 (0·1%)	0	..
	Duration of parenteral nutrition if received, days		11 (6–20)	14 (8–24)	10 (5–18)	30 (10–30)	0·0001

Data are n (%), median (IQR), or n.

* p values represent univariable testing between country income strata.

Some septic and hypovolaemic patients did not receive intravenous antibiotics (nine [37·5%] of 24 in low-income countries; 144 [24·1%] of 598 in middle-income countries; seven [18·4%] of 38 in high-income countries) or intravenous fluids (five [45·5%] of 11 in low-income countries; 84 [17·6%] of 478 in middle-income countries; 34 [45·3%] of 75 in high-income countries) within 1 h of presentation, and some hypothermic patients were not warmed (28 [7·8%] in middle-income countries; four [12·5%] in high-income countries). Only 55 (59·1%) of 93 patients in low-income countries received a general anaesthetic (of which 32 [34·4%] did not receive a general anaesthetic because they did not have surgery), compared with 2327 (81·3%) of 2860 in middle-income countries and 772 (86·2%) of 896 in high-income countries. Anaesthesia was more frequently provided by a nurse in low-income countries (n=17, 18·3%) than in middle-income countries (n=17, 0·6%) and high-income countries (n=1, 0·1%), and surgery was more frequently performed by a general surgeon or unsupervised trainee (low-income countries, n=13 [14·0%]; middle-income countries, n=54 [1·9%]; high-income countries, n=14 [1·6%]). A surgical safety checklist was used less frequently in low-income countries (n=31, 33·3%) than in middle-income countries (n=1791, 62·6%) and high-income countries (n=747, 83·4%).

In low-income countries, only eight (8·6%) patients received ventilation, three (3·2%) received parenteral nutrition, and six (6·5%) had central intravenous access, compared with much higher proportions in middle-income countries (1363 [47·7%] received ventilation, 1416 [49·5%] received parenteral nutrition, and 1263 [44·2%] had central venous access) and high-income countries (637 [71·1%], 683 [76·2%], and 670 [74·8%]).

Condition-specific patient characteristics, antenatal care, perioperative care, surgical intervention, and outcomes are detailed in the appendix (pp 16–44). In high-income countries, where 849 (94·8%) of 896 women received an antenatal ultrasound, antenatal detection rates (problem identified with or without diagnosis of condition) were: 134 (96·4%) of 139 for gastroschisis, 65 (92·9%) of 70 for exomphalos, 108 (71·1%) of 152 for intestinal atresia, 96 (64·9%) of 148 for congenital diaphragmatic hernia, 72 (51·1%) of 141 for oesophageal atresia, 49 (27·5%) of 178 for anorectal malformation, and 12 (11·2%) of 107 for Hirschsprung's disease.

The proportions of patients followed up to 30 days post primary intervention to assess survival status and presence of complications are described in the appendix (p 45). Of 3849 study patients, 418 (10·9%) were still in hospital at 30 days post intervention. Of the 2761 (71·7%) patients discharged home before 30 days, 2495 (90·4%) were followed up to 30 days.

**Figure 3:**
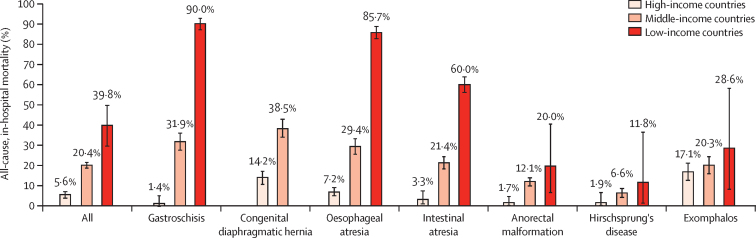
All-cause, in-hospital mortality Data are shown as percentages (95% CIs). Numbers of patients are shown in the appendix (p 46). Only one patient with congenital diaphragmatic hernia presented in a low-income country during the study period.

Overall all-cause, in-hospital mortality was 37 (39·8%) of 93 in low-income countries, 583 (20·4%) of 2860 in middle-income countries, and 50 (5·6%) of 896 in high-income countries, (p<0·0001 between all country income groups; figure 3, appendix p 46). For each condition considered individually, gastroschisis, oesophageal atresia, and intestinal atresia also showed a significant difference between all income groups; congenital diaphragmatic hernia showed a significant difference between high-income countries and middle-income countries (there were too few patients from low-income countries to make a comparison); anorectal malformation had a significant difference between high-income countries and low-income countries, and high-income countries and middle-income countries, but not between middle-income countries and low-income countries; Hirschsprung's disease and exomphalos showed no significant difference between country income groups (appendix p 46). Gastroschisis had the greatest difference in mortality (nine [90·0%] of ten in low-income countries, 97 [31·9%] of 304 in middle-income countries, two [1·4%] of 139 in high-income countries; p≤0·0001 between all country income groups), followed by congenital diaphragmatic hernia, oesophageal atresia, and intestinal atresia (figure 3, appendix p 46). Neonates accounted for 658 (98·2%) of 670 deaths. Of note, all of the patients who did not receive an intervention had either been discharged alive or died within 30 days of admission.

**Figure 4:**
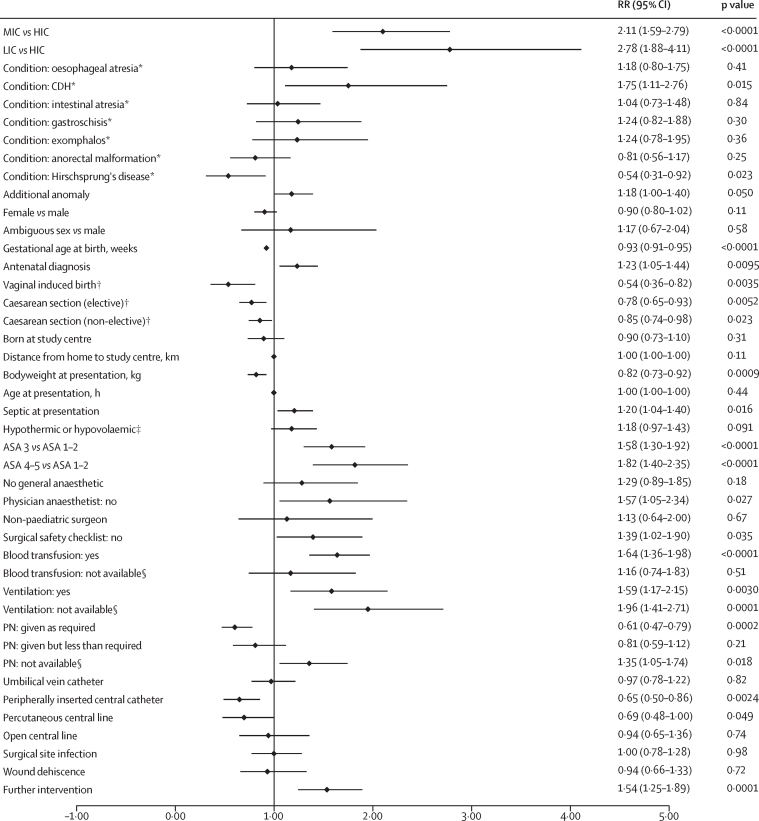
Multivariable analysis of factors affecting mortality (all patients and income settings) Of 3849 study patients, 3735 were included within this multivariable model (n=114 excluded due to missing data). Additional anomaly includes additional study condition(s) if present. Further intervention refers to the need for unplanned re-intervention within 30 days of surgery. ASA=American Society of Anesthesiologists score at primary intervention. CDH=congenital diaphragmatic hernia. HIC=high-income country. LIC=low-income country. MIC=middle-income country. PN=parenteral nutrition. RR=risk ratio. *Versus not having the specified condition. †Versus spontaneous vaginal delivery. ‡At presentation. §When required.

On multivariable analysis of all study patients, country income status was associated with the highest risk of mortality (low-income *vs* high-income country, RR 2·78 [95% CI 1·88–4·11]; middle-income *vs* high-income country, 2·11 [1·59–2·79]; figure 4). Congenital diaphragmatic hernia had the highest risk of mortality and Hirschsprung's disease had the lowest.

Antenatal diagnosis and presence of an additional anomaly were associated with higher mortality; higher gestational age and bodyweight, and delivery via induced vaginal birth or caesarean section were associated with lower mortality. For outborn patients, sepsis at presentation was associated with a higher mortality. At the time of primary intervention, mortality was higher for patients with a higher ASA score, with no physician anaesthetist present, and with a surgical safety checklist not used. In the perioperative period, not having ventilation or parenteral nutrition when required, needing or receiving ventilation or a blood transfusion, and undergoing a further unplanned intervention were associated with higher mortality. Receiving parenteral nutrition, a peripherally inserted central catheter, or a percutaneous central line were associated with lower mortality.

The multivariable analysis results of patients in LMICs were similar to those for all patients, except that gastroschisis was also significantly associated with higher mortality alongside congenital diaphragmatic hernia, and Hirschsprung's disease was no longer significantly lower (appendix p 63). In the high-income country multivariable model, no individual condition had a significantly higher or lower risk of mortality compared with the study patients without that condition (appendix p 64). Delivery type, sepsis at presentation, ASA score, use of a surgical safety checklist, ventilation, parenteral nutrition, and central intravenous access were not significantly associated with mortality. By contrast, hypothermia and hypovolaemia at presentation were associated with higher mortality.

On exploratory analysis of mortality by study condition, exomphalos was the only condition for which delivery method affected mortality risk (elective caesarean section *vs* spontaneous vaginal delivery, RR 0·25 [95% CI 0·12–0·54]; appendix pp 65–71). For congenital diaphragmatic hernia (0·63 [0·43–0·93]) and gastroschisis (0·58 [0·35–0·95]), birth at the study centre was associated with lower mortality compared with outborn patients.

30-day post-intervention mortality was similar to all-cause in-hospital mortality, with the exception that an additional 11 patients died after discharge before 30 days in middle-income countries (appendix p 72). In patients who had surgery, surgical site infection rates did not differ across income settings, whereas wound dehiscence and further unplanned intervention differed statistically, although not substantially. Median hospital stay among survivors was lowest in low-income countries (9 days [IQR 5–18]), followed by middle-income countries (14 days [8–23]) and high-income countries (20 days [12–30]). Time to death among non-survivors was similar across settings (low-income countries, 6 days [3–12]; middle-income countries, 6 days [2–13]; high-income countries, 9 days [3–15]).

Overall, the leading causes of death were sepsis (n=235, 35·1%) and respiratory failure (n=189, 28·2%; appendix p 73). Proportionally, sepsis caused more deaths in LMICs than in high-income countries.

Median observed agreement between the study and validation data was 100% (IQR 88–100; κ statistic 0·96 [IQR 0·57–1·00]; appendix pp 74–75). Variables deemed potentially inaccurate were gestational age at birth, distance from home to study centre, and time from birth to presentation (appendix pp 76–79). Validators identified eight patients missed from study inclusion (appendix p 80).

## Discussion

This international, prospective, cohort study has provided information on outcomes for almost 4000 patients with gastrointestinal congenital anomalies in 74 countries across the world. The study highlights substantial differences in mortality between low-income, middle-income, and high-income countries. The chance of dying from a gastrointestinal congenital anomaly if born in a low-income country is two in five, compared with one in five in a middle-income country and one in 20 in a high-income country. Neonates born with gastroschisis have the greatest mortality difference, with mortality of 90% in low-income countries and 32% in middle-income countries, compared with 1% in high-income countries. Thus, conditions associated with a normal lifespan for most individuals in high-income countries are frequently fatal within days of life for neonates born with the same conditions in LMICs. Tackling these inequities has the potential to reduce global neonatal mortality and is essential if preventable deaths in neonates are to be ended by 2030.^15^

Gastrointestinal congenital anomalies require surgical care, and our findings are consistent with previous studies that have shown far better surgical outcomes in high-income countries than in low-income or middle-income countries.^20^, ^21^, ^22^ However, the notably high surgical mortality rates amongst neonates in our study far exceed those reported in LMICs for older children and adults requiring surgery (between 1% and 4%, depending on the study).^20^, ^21^, ^22^ The inequities that we have found highlight neonatal surgical care as a global health priority. Our findings fit with knowledge that surgery has been neglected in the global health field; indeed, a focus in LMICs on paediatric surgery, particularly neonatal surgery, has been almost non-existent.^8^

To our knowledge, this is the first comprehensive global outcomes study of gastrointestinal congenital anomalies. It confirms previous findings from smaller, mostly single-centre, retrospective studies. A systematic review of neonatal surgery in sub-Saharan Africa reported greater than 50% mortality for emergency gastrointestinal surgery compared with 3% mortality for spina bifida and cleft lip and palate surgery.^9^ A hospital in northern Ghana reported that 96% of neonatal surgical deaths were from congenital anomalies and two-thirds of such deaths involved gastrointestinal anomalies.^23^

Our results highlight that many patients in LMICs do not receive components of neonatal surgical care that are considered essential in high-income settings. These include antenatal diagnosis, birth at a paediatric surgery centre, effective resuscitation, timely ambulance transfer for patients born in or referred to district hospitals, use of a surgical safety checklist, a physician anaesthetist at primary intervention, and basic neonatal intensive care unit resources such as ventilation, central intravenous access, and parenteral nutrition. Our large study cohort, across all income settings, enabled us to calculate the risk of mortality associated with receipt of, or lack of access to, these resources.

Our finding that antenatal diagnosis is associated with higher mortality is potentially misleading, simply reflecting easier antenatal detection of more severe cases.^24^ Indeed, on exploratory multivariable analyses, lower mortality was associated with birth at the study hospital for gastroschisis and congenital diaphragmatic hernia, and caesarean section for exomphalos, both enabled by antenatal diagnosis. Antenatal diagnosis enables delivery at a paediatric surgery centre, avoiding clinical deterioration before arrival and presentation in a poor clinical condition. Multiple indicators of poor clinical condition were significantly associated with higher mortality on multivariable analysis in LMICs: sepsis at presentation, higher ASA score at primary intervention, and a need for blood transfusion and ventilation. Although proportionally more patients who had an operation had better ASA scores in low-income countries and middle-income countries compared with high-income countries, this finding could reflect that the most sick patients in LMICs do not receive surgical intervention (and therefore an ASA score) and are palliated; competing priorities for limited resources and cost of surgery, which often requires out-of-pocket expenses for families in LMICs, might contribute to such decision making.^25^, ^26^ Our study highlights that fewer women underwent antenatal ultrasound scanning in LMICs, and even when they did, the anomalies were less frequently detected than in high-income countries, highlighting the need for both increased access to and improved quality of antenatal ultrasound. A randomised controlled trial in five LMICs showed that increased antenatal ultrasound scanning is possible (95% of women in the intervention group *vs* 43% in the control group) and that 9·3% of scanned women were referred for an ultrasound-diagnosed condition (maternal and fetal).^27^ However, the study authors found that increased antenatal diagnosis rates alone do not translate into increased hospital delivery or neonatal survival, emphasising the need for a systems approach targeting barriers to delivery at a paediatric surgery centre.^28^

Our study highlights that most patients with gastrointestinal congenital anomalies in LMICs are not born at the paediatric surgery centre; most are referred from district hospitals. Even in high-income countries, where 95% of the women received an antenatal ultrasound, not all anomalies were detected. Therefore, upskilling staff at district hospitals to deal with births at, or referrals to, these facilities is vital to prevent clinical deterioration before surgical intervention. Such an initiative in India showed successful knowledge and skills transfer by multidisciplinary paediatric surgical teams to district hospitals.^29^ Unfortunately, the 2017 WHO recommendations on newborn health include a section on management of “other severe conditions”, but do not mention congenital anomalies.^30^ Therefore, upgrading this document will be an important step for knowledge dissemination. Similarly, management of neonates with congenital anomalies should be incorporated within national WHO Every Newborn Action Plans, with a particular focus on the prevention of sepsis, hypothermia and hypovolaemia.^31^ Our study also showed that patients in LMICs travel further to hospitals and present later, frequently without hospital transport. Although they were not independently significantly associated with mortality, these factors probably also affect the clinical condition of patients on arrival, highlighting the need for improved access to timely and effective inter-hospital transportation.

At paediatric surgery centres, we identified a number of factors that were independently associated with mortality in the preoperative, intraoperative, and perioperative periods. Poorer clinical condition was associated with higher mortality, which could potentially be addressed through improved resuscitation on arrival. Our results show that not all septic and hypovolaemic patients received intravenous antibiotics and fluids within an hour of arrival, and some hypothermic patients were not warmed. The absence of a physician anaesthetist at the primary intervention and not using a surgical safety checklist were associated with a higher mortality. To address the absence of physician anaesthetists, the charity KidsOR has recently pledged funds to train paediatric anaesthetists alongside paediatric surgeons across Africa. Efforts are required to broaden the use of surgical safety checklists in LMICs; the use of implementation science techniques may help to improve this.^32^ In the perioperative period, non-availability of ventilation and parenteral nutrition when required was significantly associated with high mortality in LMICs, whereas receipt of parenteral nutrition and peripheral or percutaneous central intravenous access were associated with lower mortality.

Basic neonatal intensive care facilities have been omitted from previous global neonatal care recommendations because they are deemed expensive.^1^ However, these resources are essential, not only for surgical neonates, but also for many low-birthweight and sick neonates due to other causes, and they should be included in long-term strategies for LMICs. Such interventions lend themselves to innovative solutions, as seen with the rapid development of low-technology, cost-effective ventilation methods during the COVID-19 pandemic.^33^ The need for intensive care resources can also be reduced through context-optimised surgical techniques, such as cotside bowel reduction and sutureless closure of gastroschisis using a preformed silo, which reduces the need for ventilation.^34^ These techniques are currently being trialled in a multicentre, multinational interventional study in sub-Saharan Africa, alongside locally sourced, affordable, peripherally administered, partial parenteral nutrition, which could benefit neonatal outcomes more broadly.^35^

This study has several limitations. For feasibility, the study focused on a selection of common, high-mortality, gastrointestinal congenital anomalies rather than the full complement of anomalies. Despite the study being intentionally designed to minimise reporting burden for high-volume, low-resource centres, the proportion of patients included from low-income countries (2%) was lower than in the global population (9%).^36^ However, the proportion of study patients from middle-income countries (74%) reflects the global middle-income country population (75%).^36^ Although the number of patients included from low-income countries was relatively low, the mortality rates that we found reflect what has previously been reported in the scarce data available from these regions. For example, two of the largest single-centre observational studies on gastroschisis in low-income countries reported a mortality of 90% (136 of 151) in Uganda and 84% (80 of 95) in Zimbabwe.^37^, ^38^ The Gastroschisis Interventional Study across seven tertiary paediatric surgery centres in Ghana, Zambia, Malawi, and Tanzania (low-income countries and lower-middle income countries) reported an overall baseline mortality of 95%.^35^ Mortality rates for the other study conditions are also similar to those reported from Uganda.^38^

Despite the higher mortality rates in LMICs compared with high-income countries, the reported mortality could be an underestimation for several reasons. Data collection was done at paediatric surgery centres; some patients might have died without reaching such care in LMICs.^39^ This is evidenced by the missing patients with congenital diaphragmatic hernia, particularly within the low-income country cohort, and the under-representation of gastroschisis within the LMIC cohorts; such under-reporting also occurred in high-income countries in the 1970s.^40^ Cases with more advanced disease severity (eg, severe congenital diaphragmatic hernia) or multiple anomalies (eg, coexisting cardiac anomaly) might be more likely to die before presentation in LMICs (or not get referred). This situation would account in part for the higher proportion of patients with an ASA score of 1 in low-income countries and of 2 in middle-income countries compared with high-income countries, and also the lower proportion of patients with associated anomalies in low-income countries. However, the lower proportion of associated anomalies might also result from underdiagnosis due to lower diagnostic expertise and resources in low-income countries. If more than 20% of patients were missing the primary outcome in any given month at a participating hospital, all patients in that month were excluded. Although we used this strategy to optimise the accuracy of mortality estimates, it could have inadvertently introduced bias if poorer data collection is associated with poorer outcomes. However, no participating hospitals were excluded as a result, and only 14 months of data (37 patients) were excluded compared with 962 months of data (3849 patients) included in the study. Therefore, the effect is likely to be small. 30-day post-intervention follow-up was missing in 37·5% of patients in low-income countries, 9·0% of patients in middle-income countries, and 7·5% of patients in high-income countries; therefore, some post-discharge deaths and complications were potentially missed.

There are some additional factors to consider when interpreting the study data. Although we identified multiple factors associated with mortality through robust multivariable analyses, our findings regarding the causes of death are less robust. Cause of death was determined via clinical diagnosis of the treating physician, which is commonly multifactorial and difficult to confirm with certainty. However, our findings are consistent with the *Lancet* Newborn Series, which also reported sepsis to be the leading global cause of death in neonates more broadly.^1^ ASA scoring could have inter-rater variability in different regions of the world. Our multivariable model of patients with exomphalos included both minor and major variants; elective caesarean section is commonly confined to major variants. In low-income countries, most cases of Hirschsprung's disease were diagnosed clinically without biopsy confirmation; a lack of diagnostic facilities could result in missed patients and also inclusion of patients without the condition. Centralisation of care within and between paediatric surgery centres and multidisciplinary team care has played a key role in optimising outcomes in high-income countries, but it has not been captured within this study. In high-income countries and some middle-income countries where antenatal detection is higher, some fetuses with more severe or multiple anomalies might have been terminated, contributing to the lower mortality. However, this situation is not reflected in the data because high-income countries had the highest proportion of patients with additional anomalies, followed by middle-income countries. For feasibility and to focus on neonatal mortality, the follow-up period was limited to 30 days; longer-term follow-up is required to determine disability and quality of life.

This study provides evidence that Sustainable Development Goal 3.2 to end preventable deaths in neonates and children younger than 5 years by 2030 is unachievable without urgent action to improve neonatal surgical care in LMICs.^15^ The comprehensive study design and large cohort enabled identification of factors associated with mortality that can be addressed through improvements in antenatal and district-level care, and care at paediatric surgery centres. Strong collaboration between obstetric, neonatal, surgical, anaesthetic, and nursing teams is required. The Global Initiative for Children's Surgery provides such a platform, and the newly formed Congenital Anomalies Working Group focuses on bringing these teams together for collective action.^41^, ^42^ This study provides the necessary data to inform interventions, guidelines, and policies in the field, and to advocate for the inclusion of neonatal surgical care within national surgical, obstetric, and anaesthetic plans being developed in LMICs globally.^43^ Improving access to quality neonatal surgical care in LMICs holds the potential to reaccelerate global neonatal mortality reduction.

## Data sharing

Following publication of the study results, the full, anonymous, de-identified patient dataset will be made publicly available via the Centre for Open Science website.

## Declaration of interests

NS is the director of the London Safety and Training Solutions, which offers training in patient safety, implementation solutions, and human factors to health-care organisations. All other authors declare no competing interests.
