# Supplementary material for: Mortality from gastrointestinal congenital anomalies at 264 hospitals in 74 low-income, middle-income, and high-income countries: a multicentre, international, prospective cohort study
Source: Lancet. 2021 Jul 24;398(10297):325–39. doi: 10.1016/S0140-6736(21)00767-4 (PMC8314066; doi:10.1016/S0140-6736(21)00767-4)
Supplement: Supplementary appendix [file mmc1.pdf]

THE LANCET

Supplementary appendix

This appendix formed part of the original submission and has been peer reviewed. We post it as supplied by the authors.

Supplement to: Global PaedSurg Research Collaboration. Mortality from gastrointestinal congenital anomalies at 264 hospitals in 74 low-income, middle-income, and high-income countries: a multicentre, international, prospective cohort study. *Lancet* 2021; published online July 13. http://dx.doi.org/10.1016/S0140-6736(21)00767-4.

Supplementary appendix

Supplement to:

Mortality from Gastrointestinal Congenital Anomalies at 264 Hospitals in 74 Low-, Middle- and High-Income Countries: A Multicentre, International, Prospective Cohort Study

Mortality from Gastrointestinal Congenital Anomalies at 264 Hospitals in 74 Low-, Middle- and High-Income Countries: A Multicentre, International, Prospective Cohort Study

Global PaedSurg Research Collaboration

Appendix Supplement 1: Authorship (all co-authors PubMed citable)

Principal Investigator: Naomi Jane Wright (King's College London, UK).

Steering Committee: Adesoji Ademuyiwa (Lagos University Teaching Hospital, Nigeria), Emmanuel Ameh (National Hospital, Abuja, Nigeria), Justine Davies (University of Birmingham, UK), Kokila Lakhoo (University of Oxford and Oxford University Hospitals, UK), Dan Poenaru (McGill University, Montreal, Canada), Emily Rose Smith (Baylor University, Texas, USA), Niyi Ade-Ajayi (King's College Hospital, UK), Nick Sevdalis (King's College London, UK), Andrew JM Leather (King's College London, UK).

Writing Committee: Naomi Jane Wright* (King's College London, UK), Adesoji Ademuyiwa (Lagos University Teaching Hospital, Nigeria), Melika Akhbari (King's College London, UK), Emmanuel Ameh (National Hospital, Abuja, Nigeria), Muhammad Arshad (Liaquat National Hospital, Aga Khan University Hospital, Pakistan), Dayang Anita Abdul Aziz (UKM Medical Centre, Malaysia), Abdel Douiri (King's College London, UK), Maria Elstad (King's College London, UK), Camila Girardi Fachin (Federal University of Parana, Brazil), Kokila Lakhoo (University of Oxford and Oxford University Hospitals, UK), Bruno Martinez-Leo (Moctezuma Children's Hospital, Mexico), Ashrarur Rahman Mitul (Dhaka Shishu (Children) Hospital, Bangladesh), Alliance Niyukuri (Kibuye Hope Hospital, Burundi), Cristiana Riboni (University of Pavia, Italy), Marcus Sim (Stepping Hill Hospital, UK), Emily Rose Smith (Baylor University, Texas, USA), Stephen Tabiri (Tamale Teaching Hospital, Ghana), Dan Poenaru (McGill University, Montreal, Canada), Justine Davies (University of Birmingham, UK), Nick Sevdalis† (King's College London, UK), Niyi Ade-Ajayi† (King's College Hospital, UK), Andrew JM Leather† (King's College London, UK).
*First author and study guarantor. †Joint last authors contributed equally.

Statistical Analysis: Abdel Douiri* (King's College London, UK), Mohamed Fahmy Doheim (Alexandria University Hospital, Egypt), Maria Elstad* (King's College London, UK), Fowzia Ibrahim (King's College London, UK), Natalie Moitt (King's College London, UK), Naomi Jane Wright* (King's College London, UK).
*Lead statisticians.

Lead Organisers: Sadi Abukhalaf (Al Quds University, Palestine), Nana Adofo-Ansong (Mahikeng Provincial Hospital, South Africa), Melika Akhbari (King's College London, UK), Ahmad Alhamid (University of Aleppo, Syria), Osaid H. Alser (University of Oxford, UK), Ibrahim Al-Slaibi (West Bank and Gaza, Palestine), Emrah Aydin (Koc University, Turkey), Yousra-Imane Benaskeur (Universite de Montreal, Canada), Andile Maqhawe Dude (National University of Science and Technology, Mpilo Hospital, Zimbabwe), Shrouk M. Elghazaly (Assiut University, Egypt), Safa abdal Elrais (University of Tripoli, Libya), Helena Franco (Bond University, Australia), Sophia Hashim (University College London, UK), Laura Herrera (Geisel School of Medicine, Dartmouth, USA), Intisar Hisham (Kenyatta National Hospital, Kenya), Gabriella Hyman (University of Witwatersrand, South Africa), Henang Kwasau (College of Medicine and Allied Health Sciences, University of Sierra Leone), Yang Liu (Children's Hospital, Zhejiang University School of Medicine, China), Bruno Martinez-Leo (Moctezuma Children's Hospital, Mexico), Kelly Naranjo (Columbia University Medical Centre, USA), Sodumisa Ngwenya (National University of Science and Technology, Mpilo Hospital, Zimbabwe), Ibrahim Nour (Jordan University Hospital, Jordan), Samuel Parker (Imperial College London, UK), Cristiana Riboni (University of Pavia, Italy), Mahmoud Saleh (University of Gezira, Sudan), Hosni Khairy Salem (Cairo University, Egypt), Godfrey Sama (Muhimbili University of Health and Allied Sciences, Tanzania), Patricia Shinondo (University Teaching Hospital, Lusaka, Zambia), Marcus Sim (Stepping Hill Hospital, UK), Hannah Thompson (King's College Hospital, UK), Harmony Kaur Ubhi (King's College Hospital, UK), Agota Vaitkiene (Faculty of Medicine, Vilnius University, Lithuania), Dominique Vervoort (Harvard Medical School, USA), Isabelle Williams (Cambridge University, UK), Mina A. Yacoub (Cairo University, Egypt), Aayenah Yunus (King's College London, UK).

Lead Investigators: Muhammad Bin Amjad, Muhammad Amjad Chaudhary, Muhammad Adnan Khan Khattak (Children's Hospital, PIMS, Islamabad, Pakistan), Marlene Anaya Dominguez (Children's Hospital Manuel A.Villarroel Cochabamba, Bolivia), Samiul Hasan, Sabbir Karim, Ashrarur Rahman Mitul (Dhaka Shishu (Children) Hospital, Bangladesh), Paolo Bragagnini, Segundo Rite (Hospital Universitario Miguel Servent,

Zaragoza), Hana Arbab, Lubna Samad, Aqil Soomro (Indus Hospital, Pakistan), Niveshni Maistry (John Radcliffe Hospital, UK), Raed Nael Al-Taher, Ibrahim Rabi Nour, Osama Abdul Kareem Sarhan (Jordan University Hospital, Jordan), Muhammad Arshad, Taimur Qureshi, Hina Yousuf (Liaquat National Hospital, Pakistan), Candy SC Choo, Doris Mae Dimatatac, Shireen Anne Nah (KK Women's and Children's Hospital, Singapore), Vijay Anand Ismavel, Ann Miriam, Shajin T (Makunda Christian Leprosy and General Hospital, India), Monica Ivanov, Andreea Madalina Serban (Marie Curie Hospital in Bucharest, Romania), Bruno Martinez-Leo (Moctezuma Children's Hospital, Mexico), Eva Blazquez-Gomez, Luis Garcia-Aparicio, Martí Iriondo, Jordi Prat, Xavier Tarrado (Hospital Sant Joan de Deu, Spain), Lars Hagander, Emma Svensson (Skane University Hospital's Pediatric Care Hospital, Lund, Sweden), Alhassan Abdul-Mumin, Dominic Bagbio, Sheila Owusu, Stephen Tabiri (Tamale Teaching Hospital, Ghana), Dayang Anita Abdul Aziz (UKM Medical Centre, Kuala Lumpur, Malaysia).

Regional/Continent Leads:
ASEAN Countries: Dayang Anita Abdul Aziz (Hospital Canselor Tuanku Muhriz, Malaysia). *Australasia:* Sebastian King (Royal Children's Hospital, Australia). COSECSA Region, Africa: Milliard Derbew (Addis Ababe University, Ethiopia). *Europe:* Alexis Arnaud (Hospital Anne De Bretagne, France). *Middle-East:* Mahmoud Elfiky (Cairo University, Egypt). *North Africa:* Ahmed Negida (Zagazig University, Egypt). *South America:* Marcia Abrunhosa Matias (Hospital Federal de Bonsucesso, Brazil), Maricarmen Olivos Perez (San Juan de Dios Hospital). *West Africa:* Alliance Niyukuri (Kibuye Hope Hospital, Burundi).

Country Leads:
Afghanistan: Mohammad Rafi Fazli (Ghalib University Hospital, Herat). *Algeria:* Nadia Hamidi (Centre Hospitalo-Universitaire Hassan AEK), Souhern Touabti (Hopital Mere-Enfant). *Angola:* Rossana Francisco Chipalavela (Pionerio Zeca do Lubango). *Argentina:* Pablo Lobos (Hospital Italiano de Buenos Aires). *Australia:* Brendan Jones (Gold Coast University Hospital), Damir Ljuhar (Monash Children's Hospital), Sebastian King (Royal Children's Hospital). *Austria:* Georg Singer (Medical University of Graz). *Bangladesh:* Samiul Hasan (Dhaka Shishu (Children) Hospital). *Belgium:* Annelien Cordonnier (Katholieke Universiteit Leuven). *Bolivia:* Lorena Jauregui (Mario Ortiz Children's Hospital). *Bosnia:* Zlatan Zvizdic (Clinical Centre University of Sarajevo). *Brazil:* Marcia Abrunhosa Matias, Camila Girardi Fachin (Federal University of Parana). *Brunei Darussalam:* Janice Wong (RIPAS Hospital). *Burundi:* Alliance Niyukuri (Kibuye Hope Hospital). *Canada:* Etienne St-Louis (Montreal Children's Hospital). *Chile:* Maricarmen Olivos Perez (San Juan de Dios Hospital). *China:* Qiang Shu (Children's Hospital, Zhejiang University School of Medicine). *Colombia:* Cataline Correa (Hospital Militar Central). *Czech Republic:* Lucie Pos (Motol University Hospital, Charles university 2nd Medical Faculty). *Dominican Republic:* Elvyn Alcantara, Erick Feliz (Hospital Infantil). *Ecuador:* Luis Enrique Zea-Salazar (Omni Hospital). *Egypt:* Mahmoud Elfiky (Cairo University). *Ethiopia:* Milliard Derbew (Addis Ababe University). *France:* Alexis Arnaud (Hospital Anne De Bretagne), Matthiew Peycelon (CHU APHP-Robert Debre). *Gabon:* Nzanzu Kipata Anatole (Bongolo Hospital). *Germany:* Judith Lindert (University Hospital Schleswig Holstein, Luebeck). *Ghana:* Stephen Tabiri (Tamale Teaching Hospital). *India:* Dhruva Ghosh (Christian Medical College and Hospital). *Indonesia:* Cathline Freya Adhiwidjaja (Rumah Sakit Anak dan Bunda Harapan Kita Jakarta). *Iran:* Ahmad Khaleghnejad Tabari, Saran Lotfollahzadeh (Pediatric Surgery Research Center, RICH, Mofid Children's Hospital, SBMU). *Iraq:* Haidar Mohammad Muhssein (Kufa University). *Italy:* Noemi Pasqua, Fabrizio Vatta (San Matteo Hospital). *Kenya:* Hetal Gohil, David Kihiko (University of Nairobi and Kenyatta National Hospital). *Jordan:* Ibrahim Nour (Jordan University Hospital). *Libya:* Suad Ahmed Almada, Muhammed Elhadi, Safa abdal Elrals (Tripoli Children's Hospital). *Lithuania:* Gilvydas Verkauskas (Faculty of Medicine, Vilnius University). *Macedonia:* Toni Risteski (University Clinic for Pediatric Surgery Skopje). *Malaysia:* Dayang Anita Abdul Aziz (Hospital Canselor Tuanku Muhriz). *Mexico:* Alejandro Peharrieta Daher (Hospital Juarez de Mexico). *New Zealand:* James Hamill (Starship Children's Hospital). *Nigeria:* Taiwo Lawal (University College Hospital, Ibadan). *Palestine – Gaza*: Nathan Novotny (Al-Makassad Hospital), Maisara Al-Rayyes, Osaid H. Alser (University of Oxford, UK). *Palestine – West bank*: Nathan Novotny (Al-Makassad Hospital), Sadi Akuhalaf (Al Quds University), Ahmad G. Hammour (Palestinian Medical Complex). *Pakistan:* Muhammad Amjad Chaudhry (Children's Hospital and Shaheed Zulfiqar Ali Bhutto Medical University, Islamabad), Muhammad Arshad (Liaquat National Hospital, Aga Khan University Hospital). *Papua New Guinea:* Benjamin Yapo (Mount Hagen General Hospital), Jack Mulu (Port Morseby General Hospital). *Peru:* Lily Saldana (Instituto Nacional de Salud del Nino). *Philippines:* Beda Espineda (Philippine Children's Medical Center). *Poland:* Krystian Toczewski (Wroclaw Medical University). *Rwanda:* Isaac Ndayishimiye, Eugene Tuyishime (University Teaching Hospital of Kigali). *Saudi Arabia:* Enaam Raboe (King Fahd Armed Forces Hospital). *Scotland:* Gregor Walker (NHS Greater Glasgow and Clyde), Philip Hammond (Royal Hospital for Sick Children, Edinburgh). *Serbia:* Ivona Djordjevic (Clinical Centre for Paediatric Surgery and Orthopaedics). *Singapore:* Shireen Anne Nah (KK Women's and Children's Hospital). *South Africa:* Milind Chitnis (Frere Hospital), Gabriella Hyman (University

of Witwatersrand). *South Korea:* Sanghoon Lee, Joonhyuk Son (Samsung Medical Centre). *Spain:* Luis Garcia-Aparicio (Hospital Sant Joan de Déu). *Sweden:* Muaad Hussien (Karolinska Institutet Hospital), Lars Hagander, Emma Svensson (Skane University Hospital's Paediatric Care Hospital). *Sudan:* Enas Musa Ismail (Khartoum Teaching Hospital), Sawazen Malik (Soba University Hospital). *Syria:* Ahmad Alhamid (Aleppo University Hospital). *Tanzania:* Godfrey Sama (Muhimbili University of Health and Allied Sciences). *Thailand:* Ampaipan Boonthai (Faculty of Medicine, Ramathibodi Hospital). *Tunisia:* Nesrine Ben Hadj Dahman (Hedi Chaker Hospital). *Turkey:* Emrah Aydin (Koc University). *United Kingdom:* Nigel J Hall (University of Southampton), Naomi Wright (King's College London). *Uruguay:* Fabiola Ruth Castedo Camacho, Helena Sobrero (Centro Hospitalario Pereira Rossell). *United States of America:* Marilyn Butler (Oregon Health and Science University). *Uzbekistan:* Aliev Makhmudjan Muslimovich (Republican Specialized Scientific Practical Medical Center of Pediatrics, Tashkent). *Zambia*: Bruce Bvulani, Patricia Shinondo (University Teaching Hospital, Lusaka). *Zimbabwe*: Andile Maqhaw Dube (Mpilo Hospital).

Local Investigators:

Low-income countries

Afghanistan: Sayed Aman Haldari, Abdul Baqi Monaware, Qais Muraveji, Ajaml Sherzad, Muhammad Yousuf Murzaie, Ahmad Zia Samadi (Ataturk National Children Hospital).

Burundi: Butoyi Jean Marie Vianney (Hospital Mutoyi, Gietga); Sonia Inamuco, Alliance Niyukuri, Jesh Thiessen, Ntakarutimana Venant (Kibuye Hope Hospital, Hope Africa University); Mbonicura Jean Claude, Niyonkuru Jeremie (Teaching Hospital University of Kamenge).

Ethiopia: Milliard Derbew, Samuel Negash, Amezene Tadesse (Addis Ababe University).

Gambia: Abdoulie Bah, Kajali Camamra, Armandou Correa, A. Gai, John N. Jabang, Musa Jaiteh, Cherno S. Jallow, Charles A. Roberts, Babucarr Sowe (Edward Francis Small Teaching Hospital).

Rwanda: Yves Castar Nezerwa, Ainhoa Costas-Chavarri, Jean De Dieu Muragijimana, Georges Gasana, Joseph Lule, Albert Ndata, Turatsinze Simeon, Ian Shyaka Gashugi (Rwanda Military Hospital); Innocent Itangishaka, Emmanuel Kayibanda, Emery Manirambona, Jean Paul Mvukiyehe, Isaac Ndayishimiye, Kwizera Jean Raymond, Eugene Tuyishime (University Teaching Hospital of Kigali).

Tanzania: Joseph Elisante, Mubashir Jusabani, Sengua Koipapi, Jay Lodhia, Emmanuel Manjira, David Msuya, Sakina Rashid, Meghna Solanki, Shahnoor Syed, Murad Tarmohamed (Kilimanjaro Christian Medical Centre).

Middle-income countries

Algeria: Touabti Souhem, Nabti Sara, Brahimi Sihem, Benmanseur Saousen (Hôpital Mère - Enfant EHS El Eulma, Setif); Iaiche Achour Toufik, Bouguermouh Dania, Alouani Habiba, Baghdadi Nour El Islam Mounira (Le Nefissa Hamoud University Hospital).

Angola: Rossana Francisco Chipalavela, Liliana Aragão, Victor Gonçalves (Pediatric hospital David Bernardino).

Argentina: Diego Martin Aranda, Gabriel Comba, Marianella Depetrini, Julio Lapalma, Lucio Palazzi, Liliana Pardo, Valeria Pelussi, Veronica Sofficci, Justo Vaquila (Children's Hospital of Santa Fe); Daniel Emilio Gonzalez, Humberto Scherl (Dr Humberto Notti Pediatric Hospital of Mendoza); Marianela Bernaus, Andrea Damiani, Bonavia Horacio, Karen Liljesthrom, Carlos Mac, Daniel Putruele, Cesar Jauri (Hospital de Niños Victor J. Vilela, Rosario, Santa Fe); Marcelo Mauricio Lino Urquizo, Pablo Lobos, Pedro Mercado, Maria Florencia Varela (Hospital Italiano de Buenos Aires, Buenos Aires); Carlos Alberto, Paloma Caseb, Maria Delia Charras, Fabio Cornelli, Maria Belen Dallegre, Victor Hugo Defago, Marcos Federico Leyba, Enrique Romero Manteola, Carlos Mieres, Marcelo Molina, Adriana Morales, Celeste Carolina Patiño Gonzalez, Pablo Ravetta, Nahuel Ignacio Rivarola, Maria Tatiana Szklarz (Hospital Maternoneonatal, Córdoba); Lucia Beatriz Arnoletto, Otilia Eva Blain, Mauro Nicolas Bravo, Carla Sofia Contreras, Luciana Martina Herrera Pesara, Victor Hugo Defago, Jose Gilardi, Enrique Romero Manteola, Carlos Mieres, Maria Eugenia Moreno, Nair Rojas, Nancy Sanchez, Carlos Ariel Sferco, Soledad Simon (Hospital Materno Provincial. Córdoba); José Calderón, Elsa Arancibia Gutiérrez, Enrique Huespe, Gabriela Losa (Hospital Público Materno Infantil, Salta); Valentina Baistrocchi, Marcelo Galdeano, Pablo Medard, Yanina Silva (Hospital Rawson, San Juan); Lorna Andreussi,

Romina Avile, Ezequiel Bianchin, Rodrigo Cepeda, Alejandrina Cripovich, Maria Gabriela Puig, Carina Herrera, Susana Iracelay, Debora Montoya, Dolores Marcos, Nelly Palacios, Belen Serezo (Maternidad Martin, Rosario, Santa Fe); Ines Sueiras (Fundación Dr. J.R. Villavicencio); Oscar Di Siervi, Maria del Carmen Junes, Carolina Millán, Luzia Toselli (Fundacion Hospitalaria).

Bangladesh: Fatema Sayeed (Dhaka Medical College Hospital); Abdullah-Al-Mamun, Tamanna Ferdousi, Samiul Hasan, Umama Huq, Sabbir Karim, Khalid Mahmud, Refoyez Mahmud, Ashrarur Rahman Mitul, Sadia Sultana (Dhaka Shishu (Children) Hospital).

Belarus: Alexander Svirsky (Republican scientific practical center of pediatric surgery, Minsk).

Bolivia: Marlene Anaya Dominguez, Mariana Sadagurschi (Children's Hospital Manuel A.Villarroel Cochabamba); Lorena Jáuregui, Amalia Negrete, Denisse Sempertegui (Mario Ortiz Children's Hospital Suarez, Santa Cruz); Aracelly Terán (Women's Hospital Percy Boland Rodríguez Santa Cruz).

Bosnia and Herzegovina: Elna Biber, Nejra Dendusic, Ajla Hamidovic, Dzan Horozic, Lamija Hukic, Azra Karamustafic, Kenan Karavdic, Emina Letic, Amira Mesic, Emir Milisic, Amila Muhic, Nusret Popovic, Adnan Sabic, Adna Saracevic, Anesa Sehic, Nejra Selak, Emir Sokolovic, Nedim Vanis, Sabina Terzic, Asmir Jonuzi, Nada Zvizdic, Zlatan Zvizdic (Clinic of Paediatric Surgery, Clinical Centre University of Sarajevo, Sarajevo).

Brazil: Amanda Ginani Antunes, Fernando Antonio Bersani Amado, Giovana Camargo de Almeida, Leila Grisa Telles, Luiz Roberto Farion de Aguiar; Elisângela Mattos e Silva (Associação Hospitalar de Prot Infancia Dr. Raul Carneiro); Miguel Angelo Agulham, Karin Becker, Cristiano Bischoff, André Ivan Bradley dos Santos Dias, Camila Girardi Fachin, Elis Novochadlo Klüppel (Federal University of Parana); Adriano Luis Gomes, Flavio Augusto Menin, Danielle Paula de Oliveira, Danielle Lopes Teixeira Ferdinando (Fundação Faculdade Regional de Medicina S J Rio Preto); Fabricio Zottis Barcelos, Natalia Baseggio, Nicole Knorr Brenner, Rafael Trindade Deyl, Carolina Dure, Iuri Nunes Kist, Rafael Bueno Mazzuca, Sarah Bueno Motter, Yna Ramos, Cristine Suzana Trein (Hospital da Criança Santo Antônio - Santa Casa de Porto Alegre); Kamila de Deus Passos Leles, Érika Alves Dutra da Silva, Antonio Cirpriano Gurgel do Amaral Junior, Marília Silva Ferreira dos Santos, Mariana Furtado, Thayná Lopes de Almeida, Susy Oliveira de Andrade, Lais Sartori Giovanoni, Horácio Tamada (Hospital de Base Dr. Ary Pinheiro, Porto Velho); Leticia Feldens, Luciano Ferraz Schopf, José Carlos Soares de Fraga, Felipe Colombo de Holanda, Paola Maria Brolin Santis Isolan, Julia Loyola Ferreira, Carla Luisa Bruxel (Hospital de Clínicas de Porto Alegre); Josiane Bernartt Zanellato (Hospital do Rocio); Patricia Viana Guimarães Flores (Hospital Federal de Bonsucesso); Karina Ilheu da Silva, Karine Furtado Meyer, Cristina Reuter, Bianca Ribas, Francis Tanise Casado (Hospital Santo Antônio Blumenau Santa Catarina); Flavia de Azevedo Belesa, Acimar Gonçalves da Cunha Júnior, Beatriz Souza Barros (Hospital Materno Infantil de Brasília – HMIB); Arthur Almeida Aguiar, Carolina Gonçalves Borges, Rodrigo Melo Gallindo (Instituto de Medicina Intregral Professor Fernando Figueira); Maria Lucia da Silva Augusto, Darli Fernandes de Oliveira, Rachel Fernandes de Souza, Bianca M. R. Martins, Fernanda Ribeiro de Fernandez y Alcázar, Stella Sabbatini, Carla Silva dos Santos, Mariana de Souza Santos Ferreira, Ana Beatriz Souza Machado, Juliana Werneck Raposo (Instituto Fernandes Figueira, Rio de Janeiro); Geraldo Magela Nogueira Marques (Irmandade da Santa Casa de Misericórdia de Santos); Ana Lúcia Granja Scarabel Nogueira Carrasco, Eliane Mitiko Moriya, Patricia Sayuri Matuda Adachi (SPDM – Associação Paulista para o Desenvolvimento da Medicina); Simone de Campos Vieira Abib, Suely Dornellas do Nascimento, Ruth Guinsburg, Edson Khodor Cury, Daniela Testoni, Mila Torii Correa Leite (Universidade Federal de São Paulo – UNIFESP/EPM).

China: Liuming Huang, Mengnan Yu (Bayi Children's Hospital, The Seventh Medical Center of PLA General Hospital); Zhibao Lv, Jinjing Lu (Children's Hospital of Shanghai); Shungen Huang, Lulu Chen (Children's hospital of Soochow university); Duote Cai, Rui Chen, Zhigang Gao, Yijiang Han, Ting Huang, Liang Liang, Yang Liu, Wenjuan Luo, Qiang Shu, Jinhu Wang, Peng Wang, Xiaoxia Zhao (The Children's Hospital, Zhejiang University School of Medicine, Binjiang District, Hangzhou); Yong Feng, Chonggao Zhou (Hunan Children's Hospital, Changsha City, Hunan Province); Qiang Bai (Kunming Children's Hospital); Weibing Tang, Hua Xie, Jethishka Motee (Nanjing Children Hospital); Gang Wen, Jianming Zhu (Ningbo Women Children's Hospital); Xiaodong Guo, Junjie Chen, Donglai Hu (Jinhua municipal central hospital); Xiaozhou Bao, Haijing Li, Zhongrong Li, Junying Lv, Guowei Wu, Libin Zhu (The Second Affiliated Hospital & Yuying Children's Hospital of Wenzhou Medical University); Yu Zuo Bai, Wang Dajia (Shengjing Hospital of China Medical Unversity).

Colombia: Jerhy Andrade, Catalina Correa, Juliana Mancera, Luis Carlos Rinco (Hospital Militar Central, Bogota); Daniela Castaño Avila, Nathalia Silva Beltrán, Edgar Alzate Gallego, Juanita Gomez, Ghordana

Osorio Fory, Martha Jaramillo, Angelica Maria Forero Ladino, Otto Morales, Beatriz Sanchez, Laura Torres, (Fundacion Valle del Lili – Cali); Sergio Castañeda Espinosa, Lina Maria Pardo, Osbaldo Prieto Vargas (Materno Infantil Salucoop, Villavicencio); Nestor Julien Tinoco Guzmin (Sociedad de Cirugia de Bogota Hospital de San Jose, Bogota).

Dominican Republic: Elvyn Alcántara, Erick Féliz, Eliana Toral (Hospital Infantil Dr. Robert Reid Cabral, Santa Domingo).

Ecuador: Enrique Landivar Cino, Gabriela Yulissa Fajardo Ponce, Vicente Salinas (General North Hospital IESS de los Ceibos); Freud Aucatoma Cáceres, Daniel Hinostroza, Santiago Valencia (Hospital de Especialidades Carlos Andrade Marín, Quito); Miguel Astudillo, Elena Bucaram, Elena Chiriboga, Virginia Garcia, Marisol Guayelema, Sandra Lara, Monica Marmol, Adriana Mena, Guillermo Muñoz, Ericka Murillo, Karla Salazar, Janina Sanchez, Julian Silva, Junior Velaña, Ivan Verduga, Leonardo Verduga, Carolina Vergara, Luis Enrique Zea-Salazar (Omni Hospital, Guayaquil).

Egypt: Dina Sobhy Abdelhady, Mohamed Abouheba, Karim Osamy Ali, Alaa Mobarak Attia, Ahmed Aboelela, Omar Ossama Elsiraffy, Omar Sameh Emam, Eyad Hassan Falah, Eman Mohamed Farag, Omar Moustafa Fawzy, Khaled Abd Elrahman Ghaly, Islam Abdelmonem Ghorab, Mohamed Abada Hassan, Eman Abouzeid Abouzeid Ibrahim, Tarek Mohamed Kamel, Moustafa A. Laymouna, Mansour Mkayed Mohammed, Rawan Nemer, Ahmed Mohamed Oshiba, Hayssam Rashwan, Nourhan Akram Soliman, Mostafa Zain (Alexandria University Children's Hospital); Menan Ahmed Elsadek, Dalia Gad, Hanan Mahmoud Mohammed, Doaa Samy (Al Zahra Hospital, Cairo); Mahmoud Abdelfattah Ahmed, Gena Mohamed Hamed Elassall, Shrouk Mahmoud Elghazaly, Tarek Mohamed Essa, Mariam Albatoul Nageh Fouly, Mohammad Nageh Foly, Mohamed Omar Herdan, AbdelShakour Ibrahim, Azhar Arabi Mohammed, Ahmed Mokhtar Mahmoud, Alshaimaa M. Saad, Mahmoud M. Saad, Mohammed Hamada Takrouney (Assiut University Hospital, Assiut); Ahmed Adawy, Sherif Abdelmaksoud, Mohammed El-Ghazaly, Ahmed Elhattab, Mohamed Elewaily, Mohamed Abd Elmenam, Adham Elsaied, Mohamed Elsherbiny, Mohamed Elzohiri, Islam Mansour, Ahmed Rezk, Kareem Sadek, Mirna Sadek, Amr Saleh, Amr Shalaby, Hesham Sheir, Tamer Wafa, Basma Waseem (Mansoura University Children Hospital); Hisham Almohamady, Mohamed Amad, Mohamed Arafa, Helmy Badr, Mohamed Fayez Fouda, Ahmed Hassan Nofal (Tanta University Hospital, Tanta).

Gabon: Dieudonné Lemfuka, Solomon Machemedze, Elysé Nkunzimana, Anatole Nzanzu, Jennifer O'Connor, Zachary O'Connor (Bongolo Hospital, Lebamba).

Ghana: William Appeadu-Mensah, Frank Owusu-Sekyere (Korle-Bu Teaching Hospital, Accra); Francis A. Abantanga, Alhassan Abdul-Mumin, Priscilla Alhassan, Theophilus Teddy Kojo Anyomih, Dominic Bagbio, Sheba MP Kunfah, Sheila Owusu, Stephen Tabiri (Tamale Teaching Hospital).

India: Dhruva Ghosh, Vishal Michael (Christian Medical college Ludhiana, Punjab); Vijay Kumar, P Santosh Prabhu, Sundeep PT, Ankit Raj (Kasturba Medical College (Kasturba Hospital) Manipal); Vijay Anand Ismavel, Roshine Mary Koshy, Ann Miriam, Shajin T (Makunda Christian Leprosy and General Hospital, Bazaricherra, Karimganj District).

Indonesia: Cathline Freya Adhiwidjaja (Rumah Sakit Anak dan Bunda Harapan Kita Jakarta).

Iran: Saran Lotfollahzadeh, Fatemeh Pajouhandeh, Ahmad Khaleghnejad Tabari, Armin Vosoughi (Mofid Children's Hospital, Tehran).

Iraq: Ali Farooq Al-Mayoof, Muhamed Jassim Fadhle, Ali Egab Joda (Child's Central Teaching Hospital, Baghdad/ Mustansiriyah Medical College); Hayder Nadhim Obaid AlGabri, Haidar Mohammad Muhssein, (Kufa University, College Of Medicine).

Jordan: Layana Al-Halbouni, Sayel H. Alzraikat, Justin Z. Amarin, Waleed H. Ghanem, Sara Al Hussein, Omar Ghazi Hussein, Bashar M. Matour, Amir M. I. Murad, Rand Y. Omari, Abullah Qudah, Ali Shoubaki, Haya H. Suradi, Ahmad H. Yanis, Rajai O. Zurikat (Al-Basheer Hospital - Ministry of Health, Qasabet Amman); Husam Aldean Abuhayyeh, Mohanad Mutasem Alebbini, Raed Nael Al-Taher, Ziad A. Bataineh, Thekraiat M. Al Quran, Faris J. Abu Za'nouneh (King Abdallah University Hospital - Jordan University of Science and Technology, Faculty of Medicine); Sultan S. Abdelhamid, Ahmad Hasan Aliwisat, Hashem M. Al-Momani, Nijmeh Nasser Alsaadi, Raed Nael Al-Taher, Marzouq Amarin, Esraa Arabiat, Isam Bsisu, Mohammad S.

Jabaiti, Osama Abdul Kareem Sarhan, Raghad M. Murshidi, Ibrahim Rabi Nour, Hibah Qutishat, Yasmeen Z. Qwaider, Louay Y. Zaghlol (Jordan University Hospital, Amman).

Kenya: Syeda Ra'ana Hussain, Stanley Mugambi Roseline Ochieng (Aga Khan University Hospital, Nairobi); Corazone Deya, Polycarp Omendo Liyenzero, Kevin Ochieng (Joromongi Oginga Odinga Teaching & Referral Hospital, Kisumu); Nirav Chauhan, Soita Wycliffe Chitiavi, Kuria David, Hetal Gohil, Janan Hania Malik, Felister Mose, Robert Mugo, Timothy Mwai, James Ndungu, Swaleh Shahbal, Francisa Syovata (University of Nairobi and Kenyatta National Hospital, Nairobi).

Libya: Aya Albozidi, Wesal Omar F. Saied Aljadidi, Kamila Almabrouk, Nouriyah Ali Alwaggaa, Fatma Bibas, Mala Elgammudi, Yasmine Ali Elhajjaji, Ebtesam Othman Abdulsalam Elkhazmi, Manal Ben Enbaya Mohammed Essamilghi, Sumayyah Ghayth, Ala Khaled, Wegden Ibrahim almabrouk khalel, Tesneem Layas Randa Alamen M. Sharif, Enas Soula, Sara Abobaker Tantush, Ahmed Tarek, Hazem Ahmed, Dafer Abdulhakim S. Zreeg (Tripoli Medical Centre); Abdulsalam Abuajaila, Ma'aly Ahuhlaiga, Mabrouka Alfoghy, Fakhruddin Almuzghi, Hasan Alosta, Fatima Mousa, Saddam Ali Rmali (Misrata Medical Center, Misurata); Abubakar Abdelmalik, Hajir Aljabo, Ahmed Altaweel, Mohammad Annajjar, Lina Ali Arrmalli, Asma Aljenofi, Abdelaziz Deyab, Weam Drah, Abdallah Elayeb, Fathi Elzowawi, Yousef Krayem (Assafwa International Hospital, Misurata); Hana Milad Bazozi , Mohamed Tumi (Alaml Specialised Hospital, Misurata); Abdullahn Abdousalam Elkaloush, Ali Alnaer, Mohammed Meftah Alglaib, Habib Mansour Murtadi, Sara Trainba, Aisha Shaklawoon, Hisham Swessi (Dar Alhekma Hospital); Ali Alnaeri, Sondas Ali Gargum, Hamassat Mustufa, Hawa Ahmed Shalluf, Aya Essam Shnishah, Sara Ali Tarniba (Asalam, Misurata); Hajer Ali Shokri (Asalam Hospital); Ahmed Elhadi, Taher L. Sarkaz, Osama Tababa (Ghout El-Shaal Specialized Hospital).

Macedonia: Aleksandra Gavrilovska-Brzanov, Laze Jovcheski, Marjan Kamilovski, Vesna Cvetanovska Naunova, Toni Risteski (University Clinic for pediatric surgery Skopje).

Malaysia: Dayang Anita Abdul Aziz, Ainal Huda Abu Bakar, Shareena Ishak, Zarina Abdul Latiff, Felicia Lim, Marjmin Osman, Siti Farhan Moh Pauzi, Rufinah Teo, Azrina SK Zaman (Hospital Canselor Tuanku Muhriz, UKM Medical Centre); Tammy Teoh Han Qi (Penang General Hospital); Mohd Fitri Shukri bin Mohamed Adanan, Mohd Yusof bin Abdullah, Zulfitri bin Md Hassan, Wang Junyi, Abhirrami Lechmiannandan, Mohd Yusran Bin Othman, Cheah Hui Shan, Hafatin Fairos bt Tamaddun, Zakaria bin Zahari (Hospital Kuala Lumpur); Mohd Razin bin Hassan, Tarmizi Mohd Nor, Wan Ruzaimie Noor (Hospital Raja Perempuan Zainab II, Kota Bharu, Kelantan); Noor Fa'izatul Rahil Ambok Dalek, Hidayah Hayati binti Hashim, Ahmad Zulhisyam bin Zarwawi (Hospital Sultanah Nur Zahirah, Kuala Terengganu, Terengganu); Ahmad Shafiee bin Bujarimin, Jessmine Anntinea a/p Anthony Dass, Hemasutha a/p Kannessan, Najua binti Ramli, V Muthualhagi M Vellusamy, Quah Soong Yuen (Hospital Sultanah Aminah, Johor Bharu, Johor); Siti Nur Aien binti Hamid Hamzah, Nur Atiqah binti Mohd Hanifah, Keily Wong Yue Jyun, Hazlina Mohd Khalid, Nur Atifah binti Mohd Naim, Rahilah binti Abd Razak (Hospital Wanita dan Kanak-Kanak, Likas, Sabah).

México: Eduardo Bracho Blanchet, Daniel Ibarra, Antonio Calderon Moore, Roberto Dávila Perez, Emilio Fernandez Portilla, Raúl Villegas Silva, Cristian R. Zalles Vidal (Hospital Infantil de México Federico Gomez); Bruno Martinez-Leo, Cesar Carrasco-Ortega (Moctezuma Children's Hospital, México City); Monica Noguez Castillo, Doriela Herappe Mellado (Hospital de Especialidades del niño y mujer de Querétaro); Luis Fernando Gonzalez Cortez, Rafael Santana Ortiz, Jamie Orozco Perez, Guillermo Yanowsky Reyes (Antiguo Hospital Civil de Guadalajara "Fray Antonio Alcalde"); Moises Quiles Corona, Jorge Román Corona-Rivera, Izabel Maryalexandra Rios Flores, Lucina Bobadilla Morales, Christian Peña Padilla, Alfredo Corona Rivera, Cristian Irela Aranda Sánchez, Juan Jose Cardenas Ruiz Velasco(Nuevo Hospital Civil de Guadalajara Dr. Juan I. Menchaca, Guadalajara); Gabriela Ambriz-Gonzalez, Nestor Martinez Hernandez Magro, Francisco Javier Leon Frutos, Jose de Jesus Cardenas Baron, Alejandro González Ojeda, Yesica Yarza (Pediatric Hospital, Western Medical Center, Mexican Institute of Social Security); Pastor Aguirre-Lopez, Juan Domingo Porras, Rafael Valerio-Vazquez (Hospital del Niño Poblano, San Andres Cholula); Lorenzo Juvencio Caamal, Jose Manuel Diaz Gomez, Humberto Garcia Martinez, David Bulnes Mendizabal, Arturo Marin Montalvo, Vicente Sánchez Paredes, Pablo Sanchez Valladares (Child Hospital Dr. Rodolfo Nieto Padrón, Villahermosa Tabasco).

Nigeria: Mohammad Aminu Mohammad, Nwachukwu Calistus, Anyanwu Lofty-John Chukwuemeka, Abdullahi Lawalbarau (Amino Kano Teaching Hospital); Efeturi Agelebe, Emmanuel Akinlabi Ajao, Adebayo Gbenga Tanimola (Bowen University Teaching Hospital); Moruf Adekunle Abdulsalam, Titiloye Hannah

Agboola, Olalekan Temitope Ajai, Omolara Moronkeji Faboya, Roland Iheanyichukwu Osuoji, Bolarinwa Bolanle Temilade, Omolara Williams (Lagos State University Teaching Hospital); Adesoji Ademuyiwa, Felix Alakaloko, Christopher Bode, Olumide Abiodun Elebute, George Ihediwa, Oluwaseun Ladipo-Ajayi, Kayode Olayade, Justina Seyi-Olajide (Lagos University Teaching Hospital); Nurudeen Abdulraheem, Rilwan Adegboyega, Opeoluwa Adesanya, Olalekan S. Ajiboye, Anuoluwapo Aremo, Florence Dedeke, Moses Olanrewaju (Federal Medical Centre, Abeaokuta); Auwal M Abubakar, Ahmed Bamanga, Cyril Cletus, Lateef Oyebanji, Adewale Oyinloye, Ibrahim Shehu (Federal Medical Centre, Yola); Sani Adamu, Abubakar M. Ballah, David C. Nwosu, Michael Felix, Iliya Jalo, Kalakwa A. Mohammed, Aliu Rasaki, Yahya S.Alkali, Yusuf T. Sambo, Faruk Suleiman(Federal Teaching Hospital, Gombe); Emmanuel Ameh, Eze Chukwuemeka Ejinkeonye, Matthias Igoche, Amsa B. Mairami, Osagie O Olabisi, Maryrose Osazuwa, Adekunle T. Otuneye, Morayo Monsurat Salawu, Mariya Mukhtar Yola (National Hospital, Abuja); Efeturi Agelebe, Emmanuel Akinlabi Ajao, Adebayo Gbenga Tanimola (Bowen University Teaching Hospital); Ugwunne Chuka A., Victor Modekwe, Philip Mari Mshelbwala, Osuigwe Andrew N., Olabisi Osagie, Ekwunife Hyginus O., Ugwu Jideofor O., Samson Olori, Ezidiegwu Ugochukwu S. (Nnamdi Azikiwe University Teaching Hospital); Nwokoro Collins, Ogundele Ibukunolu, Amo Shonubi (Olabisi Onabanjo University Teaching Hospital); Ifeanyichukwu Kelvin Egbuchulem, Felix O. Kumolalo, Taiwo Lawal, Olakayode Ogundoyin, Dare I. Olulana, Ikechukwu Ulasi (University College Hospital, Ibadan); Samson Olori, Olabisi Osagie, Philip Mari Mshelbwala (University of Abuja Teaching Hospital); Ekpemo, Christopher Chim Amah, Isaac Chukwu, Sebastian Okwuchukwu Ekenze, Emmanuel Ifeanyi Nwangwu, Elochukwu Perpetua Nwankwo, Nene Elsie Obianyo, Ezomike Uchechukwu Obiora (University of Nigeria Teaching Hospital).

Pakistan: Muhammad Arshad, Sohail Dogar, Ayesha Saleem (Aga Khan University Hospital, Karachi); Nadeem Akhter, Hassan Ali, Ali Raza Chaudary, Muhammad Amjad Chaudhry, Mudassir Fayaz Gondal, Muhammad Bin Amjad, Muhammad Umer Jamal, Safina Karim, Muhammad Adnan Khan Khattak, Uzma Mumtaz, Muhammad Umar Nisar, Ghuri Shankar Pandit, Rafee Raza (Children's Hospital, PIMS, and Shaheed Zulfiqar Ali Bhutto Medical University, Islamabad); Hana Arbab, Lubna Samad, Aqil Soomro (Indus Hospital, Karachi); Muhammad Kashif Bashir, Fatima Naumeri, Zarqa Rani, Muhammad Sharif (King Edward Medical University, Lahore); Muhammad Arshad, Taimur Qureshi, Hina Yousuf (Liaquat National Hospital); Naveed Haider, Soban Hameed, Imran Hashim, Muhammad Bilal Mirza, Wajeeh ur Rehman, Muhammad Saleem, Nabila Talat (The Children’s Hospital & The institute of Child Health, Lahore).

Palestine, Gaza: Hala M. Al-Attar, Adham Ashraf AbuAttaya, Mohammed A. M. Aldirawi, Sharif S. Alijla, Haya Abdulnasser Ali Alshaikhkhalil, Sulaiman T. Alzerei, Yara shareef Ashour, Ahmed S Darwish, Yasmin Mohammed Khalil Abu Jamie, Mohamed Anwer El Tallaa, Bisan D. M. Wishah, Nidal Wishah (Al-Aqsa Hospital, Gaza Strip); Walaa Almadhoun, Ashraf Ayman Al-Salhi, Soha Marwan Salem Alwadia, Dina Ayman Ashour, Nagham Mohammed Al Dammagh, Abdalkarim Yhya Hammato, Doaa Khalil Hasanain, Mustafa abu jayyab, Samy Rafat Ramadan Naim, Ismail Nassar, Anas Qawwash, Jamal Mohammed Salim, Ahmad Ashraf Shaheen, Eman Abu Shiha (Al-Shifa Hospital, Gaza Strip); Nasrallah Ashraf Al Massry, Abdulwhhab Ayman Abu Alamrain, Khaled Alnahhal, Mohammed Alser, Samar H. Al-Shwaikh, Hisham Dalloul, Aya Mahmoud Elmzyyen, Omar Adly ElShaer, Jehad Fares, Amir Talat Sheda Ghabayen, Najlaa Abu Jamie, Najlaa Abu Jamie, Walaa Lubbad, Ayoub A. Nemer, Aya Azmi Shehda Salha, Nureddin Shaheen (European Gaza Hospital, Gaza Strip); Sharif Alijla (Islamic University of Gaza, Gaza).

Palestine, West Bank: Fawzi Abu-Nejmah, Firas Alqarajeh, Tareq Z. Alzughayyar, Hadeel Awad, Jomana Madieh, Osaid Shaher Shabana (Ahli Hospital, Hebron); Raghad Mohammad Abdu Alkareem, Tasneem Fathi Hasan Almusleh, Yazid Yousef Mahmoud Atatri, Wisam Baker, Yasmeen Z. Qwaider, Adly Halabi, Raghad Abdullateef Lahlooh, Mahmoud Fuad Sbaih, Abdulraheem Adnan Abdulraheem Tahyneh (Jenin Government Hospital, Jenin); Abrar Ghassan Balousha, Mohammad Omar Mohammad Abdelhafez, Yara Ajrami, Hasan Subhi Hasan Abu Al-saleem, Ainaa Ata Mohammad Alzamari, Basheer Ba’baa’, Muath A. M. Baniowda, Ammar Darwish, Abu Farha, Ahmad G. Hammouri, Majd Yousef Mohammed Hassan, Nathan Novotny, Abrar Shaheen Sehwiel, Mohammed Shehada, Mervat Sufian, Bashar Yaghi (Palestinian Medical Complex, Ramallah); Rami Anwar Misk, Safa’ Jamal Abatli, Ahmad Abueideh, Ali Abdelhay Abumunshar, Hiba Al-Tammam, Firas Anaya, Hasan Arafat, Muath Abdelrahem Fuad Assi, Abd Al-Naser Bany Odi, Doha Mustafa Saleh Beshtawi, Dania Jaber, Yara Imad Omar KayedLara Zahi Adel Khatatba, Asef Belal Qadomi, Fadwa Sharabati (Rafidia Surgery Hospital, Nablus); Ihda Adawi, Samer Adawi, Mohammad Omar Ibrahim Alqor, Asmahan Mohammad Suliman alzeer, Ahmad Samih Arar, Osama Majed Darras, Annan Ebeido, Ihsan Ghazzawi, ma'alem sameer hajajreh, Sharehan Joubeh, AbdelRazzaq Abu Mayaleh, Mahmoud M. Qabaja, Moradallah Asad Fahmi Qunaibi, Yasmeen Ahmad Samarah, Mutassem Sharabati, Dua Hasan Yaghi (Red Crescent Society, Hebron).

Papua New Guinea: Jack Mulu (Port Moresby General Hospital).

Peru: Francisco Lapouble-Ramirez, Genaro Llap-Unchón, Florangel Patricia Delgado Malaga (Cayetano Heredia National Hospital, Lima); María Rosa Ortiz Argomedo, Victor Casquero, Sthefanny Vega Centeno (Guillermo Almenara National Hospital, Lima); Cathy Alanguia, Loreley Raquel Cardenas Alva, Nancy Rossana Mendoza Leon, Maria Susana Loo Neyra, Cintya Maria de Jesus Torres Picón, Natalia Huaytalla Quiroz (Hospital Dos de Mayo, Lima Flor de Maria Diaz Castañeda); Waldo Berrocal Anaya, Patricia Chavez Galvez, Prince Pamela Aguilar Gargurevich, Carmen Guisse, Erika Ramos Paredes (Hospital Nacional Alberto Sabogal Sologuren); Carlos Segura Calle, Danny Dominguez, Jenny Arauco (Hospital Nacional Docente Madre Niño San Bartolomé); Luis Ormeño Calderón, Cesar Diaz Leon, Joan Elizabeth Gutierrez Maldonado, Ximena Guilardi Silva, Miriam Daniela Fernandez Wilson (Hospital Nacional Edgardo Rebagliati Martins); Marlene Diaz Echegaray, Isidora Garcia, Fernando Trigoso Mori, Veronica Raquel Cañapataña Sahuanay, Lily Saldaña (Instituto Nacional de Salud del Niño); Karina Hernández Córdova, Faye Aguilar Aguilar, Lenny Flores Carbajal, Carlos Mendoza Chiroque, Raul Ramirez De La Cruz, Gladys Johana Sulca Cruzado, Patricia Paredes Espinoza, Natalia Tovar Gutierrez, Jose Luis Apaza Leon, Angel Samanez Obeso, Juan Jose Mendoza Oviedo, Luz Mery Brito Quevedo, Edgar Fernando Delgado Quinteros, Jennifer Sotelo Sanchez, Carolina Paz Soldan (Instituto Nacional de Salud de Nino de San Borja); Porfirio Rivera Altamirano, Julio Rivera Alvarez, Rocio Anicama Elias, Juan Jose Salinas Barreto, Juan Pedro Vasquez Matos, Cesar Torres Miranda, Andrea Gutarra Palomino, Jesmarina Ledesma Peraza, Fernando Ayque Rosas, Jackelyne Alvarado Zelada (Instituto Nacional Materno Perinatal); Segundo Gamboa Kcomt, Luis Ortego Sotelo, Araceli Villalba Villalba (Pediatric Emergencies Hospital, Lima).

Philippines: Beda Espineda, Johann de Guzman, Raisa Yu (Philippine Children's Medical Center, Quezon City).

Romania: Calin Calancea, Bogdan Gavrila, Aurel Mironescu, Liviu Muntean, Adrian Papa, Roxana Verdeata, Lucian Vida (Children Clinical Hospital of Brasov, Brasov/University of Brasov); Vlad Coșoreanu, Sebastian Ionescu, Monica Ivanov, Andreea Madalina Serban (Marie Curie Hospital in Bucharest).

Serbia: Ivona Djordjevic, Ana Kostic, Maja Raicevic, Andjelka Slavkovic, Dragoljub Zivanovic (Clinical Centre for Paediatric Surgery and Orthopaedics, Clinical Center Niš); Marija Lukac, Milan Slavkovic, Miona Stojanovic, Djordje Toplicic (University Children's Hospital in Belgrade).

South Africa: Sanele Madziba, Hansraj Mangray, Shamaman Harilal (Greys Hospital, Pietermaritzburg); Cecilia Rengura, David Tasker, Thozama Siyotula, Marion Arnold, Dirk von Delft, Uzair Jooma, Omar Khamag, Akhona Mbonisweni, Elmarie vd Merwe (Red Cross War Memorial Children's Hospital, Cape Town); Corné de Vos, Delphine Nkuliza, Daniel Sidler, Guigui Sikwete (Tygerberg Children's Hospital).

Sri Lanka: Benedict Paul Bright, Tharanga Gamage, Naveen Wijekoon (Lady Ridgeway hospital for children, Colombo).

Sudan: Alaa Abdulrahman, Ahmad Elian Abu Ajwa, Enas Musa Ismail, Sawazen Malik, Ola Ahmed Abdulmjeed Mohammed, Mohammed Salah, Mahmoud Saleh (Khartoum University Hospital).

Syria: Ahmad Alhamid, Ahmadfateh Assi, Mohammad Mohannad Batal, Vivian Faks, Mohammed Morjan, Ahmad Mouakeh, Mohamad Bassel Mouti, Ahmad Sankari Tarabishi (Aleppo University Hospital, Aleppo); Aos Alhamid, Ziad Aljarad, Mohammed Morjan (AlShahbaa Hospital, Aleppo city).

Thailand: Jiraporn Khorana (Faculty of Medicine, Chiang Mai University); Sirima Liukitithara. Anan Sriniworn (Hatyai hospital); Ratiyaporn Phannua, Patchareeporn Tanming, Kanokrat Thaiwatcharamas (Faculty of Medicine, Khon Kaen University); Wannisa Poocharoen (Queen Sirikit National Institute of Child Health (QSNICH));Ampaipan Boonthai (Faculty of Medicine, Ramathibodi Hospital, Bangkok); Monawat Ngerncham (Faculty of Medicine, Siriraj Hospital);Wasun Nuntasunti (Sisaket Hospital).

Tunisia: Roua Abdelmoumen, Nahla Kchich, Lassaad Sahnoun (Fattouma Bourguiba Hospital, Monastir)

Turkey: Müge Çelik, İlknur Banlı Cesur, Hatice Kaya, Gökmen Kurt, Mustafa Kurthan Mert, Zerrin Özçelik (Adana State Training Hospital); Seçil Erçin, Egemen Eroğlu (Amerikan Hospital, Istanbul); Fatih Akova, Mehmet Pasaoğlu (Biruni University Hospital, Istanbul); M.Eren Akan, Merve Altın, Merve Aykut, Ali

Canözer, Melisa Erdem, Ebru Ergenekon, Ramazan Karabulut, Elif Eren, Elif Keleş, Teymursha Muradi, Kıvanç Şeref, Kaan Sönmez, Zafer TürkyılmazCanan Türkyılmaz, Ali Yalcinkaya, Aslı Öztürk Yeniay, (Gazi University School of Medicine Yakup Aslan, Ahmet Beşir, Hatice Sonay Yalçın Cömert, Şükran Geze, Mustafa İmamoğlu, Şebnem Kader, Mehmet Mutlu, Bahanur Çekiç Haluk Sarıhan (Karadeniz Technical University, Trabzon); Emrah Aydin, Elif Demirtas, Egemen Eroğlu, Tugba Gursoy, Mehmet Ali Ozen, Alkim Yildirim (Koç University, Istanbul); Nazile Erturk, Nilay Hakan, Suleyman Cuneyt Karakus, Alev Suzen (Muğla Education and Research Hospital).

Uzbekistan: Shukurali Eshkabilov (Republican Perinatal Center); Avazjon Dekhkonboev, Aliev Makhmudjan Muslimovich, Rustam Z. Yuldashev (Republican Specialized Scientific Practical Medical Center of Pediatrics, Tashkent).

Zambia: Bruce Bvulani, Chisengo Kapihya, Azad Patel, Patricia Shinondo (University Teaching Hospital, Lusaka).

Zimbabwe: Nyasha Dirani, Andile Maqhawe Dube, Gloria Mhuruyengwe, Mandlenkosi Ncube, Nyasha Ngoromani, Sodumisa Ngwenya, Privilege Sibanda (National University of Science and Technology, Mpilo Hospital).

High-income countries

Australia: Kiera Roberts, Gordon Thomas, Rasika Naidoo, Soundappan Sannappa Venkatraman (Children's Hospital at Westmead); Brendan Jones (Gold Coast University Hospital); Nicholas Ensor, Damir Ljuhar, Ramesh Nataraja, Mithila Sivasubramaniam (Monash Children's Hospital); Matthew Jones, Sebastian King, Sharman Tan Tanny, Warwick Teague (The Royal Children's Hospital).

Austria: Georg Singer, Holger Till (Medical University of Graz).

Belgium: Martine Dassonville, Manon Pigeolet (Queen Fabiola University Children's Hospital).

Brunei Darussalam: Zahidah Adlynee Haji Ahmad, Anas Shikha, Win Sabai Phyu Win, Janice Wong (RIPAS Hospital).

Canada: Eileen Duggan, Sherif Emil, Elena Guadagno, Jean-Martin Laberge, Dan Poenaru, Pramod Puligandla, Nadia Safa, Kenneth Shaw, Etienne St-Louis, Hussein Wissanji (Montreal Children's Hospital); Ann Aspirot, Yousra-Imane Benaskeur, Louise Caouette-Laberge, Shahrzad Joharifard, Léamarie Meloche-Dumas, Nelson Piché, Dickens St-Vil (Sainte-Justine Children's Hospital).

Chile: Maria Consuelo Puentes, Carolina Mendez Benavente, Maricarmen Olivos Perez, Paola Osses Leal (San Juan de Dios Hospital).

Czech Republic: Ladislav Planka, Jan Škvařil (Fakultni Nemocnice BRNO); Radek Štichhauer, Antonín Šafus (University Hospital Hradec Kralove); Klára Berková, Lucie Pos, Tereza Racková, Michal Rygl, Barbora Trojanová (University Hospital Motol).

England: Felicity Arthur, Paul Losty (Alder Hey Children's Hospital); Marcia Abrunhosa Matias, Afnan Alzubair, Suren Arul, Oliver Gee, Ingo Jester, Tony Lander, Benjamin Martin, Max Pachl, Michael Singh, Giampiero Soccorso (Birmingham Children's Hospital); Kate Burns, Hannah Rhodes, Rebecca Roberts, Robin Garrett-Cox (Bristol Royal Hospital for Children) Alireza Keshtgar, Dorothy Kufeji, Simon McCluney, Caroline Pardy (Evelina Children's Hospital, Guy's & St.Thomas' NHS Trust); Hannah Cornwall, Kat Ford, Kokila Lakhoo, Niveshni Maistry, Krithi Ravi (John Radcliffe Hospital); Niyi Ade-Ajayi, Alex MacDonald, Shahad Sabti (King's College Hospital); Fraser Cameron, Amy Hughes Thomas, Merina Kurian, Jayaram Sivaraj, Jason Langley (Nottingham Children's Hospital); Thomas Middleton, Jonathan Sutcliffe (Leeds General Infirmary); Nigel J Hall, Arun Kelay (Southampton General Hospital); Kate Bradshaw, Ceri Jones, Dean Rex, Mark C Thomas (St. George's Hospital).

France: Elodie Haraux (CHU Amiens); Xavier Delforge (CHU Amiens-Picardie); Virginie Fouquet-Languillat, Florent Guerin, Géraldine Hery (CHU APHP-Bicêtre); Thomas Blanc, Aline Broch,Jules Kohaut (CHU APHP-Necker); Liza Ali, Arnaud Bonnard, Matthieu Peycelon (CHU APHP-Robert Debré); Thomas Delefortrie, Luke Harper (CHU Bordeaux); Quentin Ballouhey, Laurent Fourcade, Céline Grosos (CHU Limoges); Maria

Giovanna Grella, Guillaume Levard, Benoit Parmentier (CHU Poitiers); Alexis Arnaud, Camille Duchesnes, Coralie Defert, Samia Laraqui Hossini, Caroline Le Gall (CHU Rennes); Lucie Grynberg, Mariette Renaux Petel (CHU Rouen); Aurelien Scalabre (CHU St Etienne); Olivier Abbo, Mélodie Juricic, Sofia Mouttalib (CHU Toulouse).

Germany: Udo Rolle, Andrea Schmedding (Frankfurt University Hospital); Peter Goebel, Ludwig Patzer, Anke Rissmann (Hospital St. Barbara Elisabeth Halle); Alexandra Antunez-Mora, Bernd Tillig, Sylvester von Bismarck, Alexandra Wilke (Kinderchirurgie Vivantes Neukölln); Christian Knorr, Patricia Reis Barbosa, Domitille Stark (Krankenhaus Barmherzige Brüder Regensburg); Anke Rissmann (Medical Faculty Otto-von-Guericke University Magdeburg); Judith Lindert (University Hospital Schleswig Holstein, Luebeck).

Italy: Luigi Avolio, Gian Battista Parigi, Marco Brunero, Silvia Cavaiuolo, Piero Giovanni Romano, Marinella Guazzotti, Francesco Manni, Matilde Molinelli, Noemi Pasqua, Alessandro Raffaele, Cristiana Riboni, Fabrizio Vatta (Fondazione IRCCS Policlinico San Matteo, Pavia).

Hungary: Laszlo Juhasz, Anna Rieth (Albert Szent-Györgyi Clinical Centre).

Lithuania: Rūta Dagilytė, Pranas Gurskas, Arunas Liubsys, Arunas Strumila, Gilvydas Verkauskas (Children‘s Hospital, Affiliate of Vilnius University Hospital SK); Vidmantas Barauskas, Mindaugas Berzanskis, Dalius Malcius, Agne Mikneviciute, Asta Vinskaite (Lithuanian University of Health Sciences).

New Zealand: Spencer Beasley, Lucy Goddard (Christchurch Hospital); Timothy Hall, James Hamill, Liam Vierboom (Starship Children's Hospital); Stephen Adams, Jitoko Cama, Sridharan Jayaratnam, Askar Kukkady, Udaya Samarakkody, Marilyn Wong (Waikato Hospital); Mark Stringer, Naveen Weeratunga (Wellington Hospital).

Poland: Piotr Kalicińsk, Grzegorz Kowalewsk, Anna Roszkiewicz, Agata Trypens (Children’s Memorial Health Institute, Warsaw); Stefan Anzelewicz, Piotr Czauderna, Hanna Garnier, Marta Osowicka, Dariusz Wyrzykowski (Medical University of Gdańsk, Gdańsk); Tomasz Koszutski, Szymon Tobor, Agnieszka Wolny (Medical University of Silesia, Katowice); Andrzej Grabowski, Wojciech Korlacki, Michal Pasierbek (Department of Children’s Developmental Defects Surgery and Traumatology Medical University of Silesia, Zabrze); Aneta Piotrowska, Przemysław Wolak (Wladyslaw Buszkowski Children's Hospital, Kielce); Sylwester Gerus, Dariusz Patkowski, Krystian Toczewski (Wroclaw Medical University).

Qatar: Mansour Ali, Amer Alsaied, David Sigalet (Sidra Medecine, Doha).

Saudi Arabia: Reem Abdelbaqi, Khalid Alattas, Ameen Alsaggaf, Ahmed Atta, Mohamed Fayez, Alaa Ghallab, Yar Kano, Yazeed Owiwi, Enaam Raboe, Hanin Shalaby, Omar Sindi, Asaad Saleh Radwan, Ali Zeinelabdeen, Mazen Zidan (King Fahd Armed Forces Hospital); Mohammad Almosaibli, Abdullah Alshehri, Tariq Altokhais, Fahad Alturki (King Saud University College of Medicine).

Scotland: Dasha Krisanova, Gregor Walker (Royal Hospital for Children, Glasgow); Wisam Abbas, Philip Hammond (Royal Hospital for Sick Children).

Singapore: Candy SC Choo, Doris Mae Dimatatac, Shireen Anne Nah (KK Women’s and Children’s Hospital).

South Korea: In ji Hwang, Ju Yeon Lee, Eung Song Song (Cheonnam National University Hospital); Sanghoon Lee, Joonhyuk Son (Samsung Medical Center); Hyun-Young Kim, Hee-Beom Yang, Joong Kee Youn (Seoul National University Children's Hospital); Jae Hee Chung, Seok Hyeon Cho (Seoul St. Mary's Hospital, The Catholic University of Korea).

Spain: Isabel Casal Beloy, Miriam García (Complejo Hospitalario Universitario de A Coruña (CHUAC)); Verónica Alonso, Isabel Carrillo, Oscar Gomez, Alberto Sanchez, Maria Elena Molina Vazquez (Hospital Clinico Universitario de Valladolid); Paolo Bragagnini, Mercedes Ruiz de Temiño Bravo, Alexander Siles Hinojosa, Segundo Rite (Hospital Universitario Miguel Servet); Detlef Oliu San Miguel (Maternal Hospital Infantil De Badajoz); Jenny Arboleda, Eva Blazquez-Gomez, Luis Garcia-Aparicio, Martí Iriondo, Jordi Prat, Xavier Tarrado (Hospital Sant Joan de Déu).

Sweden: Carmen Mesas Burgos, Muaad Hussien, Tomas Wester (Astrid Lindgrens Children's Hospital); Kate Abrahamsson, Michaela Dellenmark Blom, Vladimir Gatzinsky (Queen Silvia Children's Hospital); Helena

Arnadóttir, Christina Granéli, Emma Grottling, Lars Hagander, Erik Omling, Martin Salö, Emma Svensson (Skåne University Hospital's Pediatric Care Hospital).

Uruguay: Daniel Borbonet, Fabiola Ruth Castedo Camacho, Gaston Acuña, Helena Sobrero, Vinicio Jimenez Morejon, Paul Puglia, (Centro Hospitalario Pereira Rossell).

United States of America: Fizan Abdullah, Monica Langer, Jonathan Vacek (Ann & Robert H. Lurie Children's Hospital of Chicago); Jonathan Chan (Beaumont Hospital in Detroit); Pavan Brahmamdam, Karen Sherer, Alan Tom (Beaumont Childrens Hospital in Royal Oak); Reto Baertschiger, Laura Herrera, Mary Leech (Children's Hospital at Dartmouth); Afua Amoabin, Alex Bowder, Terry Derks, Brooke Pinar, Sabina M Siddiqui (Children's Hospital of Wisconsin); Lauren Camp, Jeremy Chang, Kathryn Fowler, Ankush Gosain, Erica Hodgman, Bailey D. Lyttle, Lydia McColl Makepeace, Sara Mansfield, Maria Mora, Regan Williams (Le Bonheur Children's Hospital Memphis); Hira Ahmad, Marc Levitt (Nationwide Children's Hospital); Julie Khlevner, William Middlesworth, Kelly Naranjo (New York Presbyterian Morgan Stanley Children's Hospital); Marilyn Butler, Aaron Cunningham, Brandy Gonzales, Sanjay Krishnaswami (Oregon Health and Science University); Jodie Greenberg, Katrine Løfberg, Kate Davenport (Phoenix Children's Hospital); Sarah Fox, Samir Gadepalli, April Hamilton, Stephanie Johnson, Mercedes Pilkington (University of Michigan, Ann Arbor); Jenna Kylene Davis, Nicole Lin, Juan Sola, Yang Yaom, Stephen S. Burks (University of Miami); Murad Almasri, John Coon, Marianelly Fernandez Ferrer, Joann Gonzalez, Medhavi Honhar, Sergio lerma, Sunil Jain, Varun Modi (University of Texas Medical Branch); Chukwubinyelum Amaechi, Alana Beres, Luke J. Dosselman, Jillian Jacobson, Mark N. Pernik (UT Southwestern Medical Center, Children's Medical Center of Dallas); Maija Cheung, Griffin Coghill, Nensi Ruzgar, Sarah Ullrich (Yale New Haven Hospital).

Supplementary Methods 1: Sample size calculation

A sample size calculation was undertaken using Stata/IC 15·0 based on Bonferroni correction for multiple testing, assuming 80% power and an overall type 1 error of 5% (Methods Table 1). This was calculated for the primary outcome of mortality in low- and middle-income countries (LMICs) compared to high-income countries (HICs) and also low, middle and high-income countries (LM&HICs) separately. Mortality estimations utilised in the calculation were based on pooled data from published studies of these conditions in LM&HICs respectively at the time of protocol development as referenced in the first column.

Methods Table 1: Estimated mortality and sample sizes for low, middle and high-income countries and the mean number of cases per month per hospital globally

Condition	Mortality LIC (%, n)	Mortality MIC (%, n)	Mortality LMIC combined (%, n)	Mortality HIC (%, n)	Sample size for LIC	Sample size for MIC	Sample size for HIC	Sample size for LMIC vs HIC (per group)	Mean no. cases/ month/ hospital (L,M&HIC combined)
Oesophageal atresia [1-18]	79·5% (62/78)	41·8% (623/1488)	43·7% (685/1566)	2·7% (6/221)	34	34	23	21	1·02
Congenital diaphragmatic hernia* [19-27]	-	47·4% (130/274)	47·4% (130/274)	20·4% (201/982)	-	-	-	63	0·54
Intestinal atresia [28-38]	42·9% (42/98)	40·0% (97/241)	41·0% (139/339)	2·9% (12/407)	6014	6014	25	24	0·63
Gastroschisis [1,39-54]	83·1% (211/254)	42·6% (205/481)	56·6% (416/735)	3·7% (28/748)	29	29	24	15	0·85
Exomphalos [1,55-66]	25·5% (41/161)	31·9% (132/414)	30·1% (173/575)	12·7% (40/316)	1040	1040	196	115	0·63
Anorectal malformation [1,39,17,67-76]	26·3% (26/99)	17·5% (243/1391)	18·1% (269/1490)	3% (14/462)	460	460	90	85	1·34
Hirschsprung's Disease [77-80]	19·1% (33/173)	16·8% (55/328)	17·6% (88/501)	2·3% (43/1897)	5802	5802	85	79	2·21

*Representative data on the mortality from congenital diaphragmatic hernia in LICs is not currently available. HIC: High-income countries. IQR: Interquartile range. LMIC: Low- and middle-income countries. LIC: Low-income countries. MIC: Middle-income countries.

Based on the patient numbers included in the previously undertaken PaedSurg Africa study, which utilised a similar study design, the estimated sample sizes to detect a significant difference in mortality between LMICs and HICs in this study are achievable. During the PaedSurg Africa study, data was collected by 220 local investigators across 76 hospitals in 23-countries in sub-Saharan Africa (SSA) for the same study duration (7-months) and included 188 patients with anorectal malformation and 111 with gastroschisis. Since this study is global rather than limited to SSA we predicted that the patient numbers would exceed this.

Based on the limited data available from LMICs, it did not appear to be feasible to detect significant differences in mortality between LICs and MICs for intestinal atresia, exomphalos, anorectal malformation and Hirschsprung's disease; congenital diaphragmatic hernia was unknown since there was no reliable data from LICs. Hence, analysis was planned between HICs and LMICs unless sufficient data was collected to detect significant differences in mortality between LM&HICs, separately.

Estimated study population

The mean number of cases presenting to a hospital per month for each study condition was estimated from published studies across all income settings; most institutions caring for patients with these conditions receive 1-2 news cases per month (Methods Table 1). Hence, each participating hospital was expected to have approximately 7-14 cases to include in the study per month.

We aimed to include a minimum of 365 months of data; 183 months from LMICs and 183 months from HICs. This was to ensure enough cases of exomphalos to determine a significant difference between LMICs and HICs; fewer months of data were required to determine significant differences in mortality for the other study conditions. This translated to data collection by 365 hospitals for 1-month each or data collection by 52 hospitals for 7-months each or a variant in between (i.e 100 hospitals for 3-4 months each). An up-to-date total of patient numbers was included on the study website (www.globalpaedsurg.com) so that local investigators could work together towards this target.

References

1. Farmer D, Sitkin N, Lofberg K, Donkor P, Ozgediz D. Surgical Interventions for Congenital Anomalies. In: Debas HT, Donkor P, Gawande A, Jamison DT, Kruk ME, Mock CN, eds. Essential Surgery: Disease Control Priorities, Third Edition (Volume 1). Washington (DC); 2015.
2. Roberts K, Karpelowsky J, Fitzgerald DA, Soundappan SS. Outcomes of oesophageal atresia and tracheo-oesophageal fistula repair. *J Paediatr Child Health* 2016; **52**(7): 694-8.
3. Yang YF, Dong R, Zheng C, et al. Outcomes of thoracoscopy versus thoracotomy for esophageal atresia with tracheoesophageal fistula repair: A PRISMA-compliant systematic review and meta-analysis. *Medicine (Baltimore)* 2016; **95**(30): e4428.
4. Agarwala S, Bhatnagar V, Bajpai M, Gupta DK, Mitra DK. Factors contributing to poor results of treatment of esophageal atresia in developing countries. *Pediatr Surg Int* 1996; **11**(5-6): 312-5.
5. Fall M, Mbaye PA, Horace HJ, et al. Oesophageal atresia: Diagnosis and prognosis in Dakar, Senegal. *Afr J Paediatr Surg* 2015; **12**(3): 187-90.
6. Nwosu JN, Onyekwulu FA. Oesophageal atresia and tracheooesophageal fistula: a 12 years experience in a developing nation. *Niger J Med* 2013; **22**(4): 295-8.
7. Osei-Nketiah S, Hesse AA, Appeadu-Mensah W, Glover-Addy H, Etwire VK, Sarpong P. Management of oesophageal atresia in a developing country: Is primary repair forbidden? *Afr J Paediatr Surg* 2016; **13**(3): 114-9.
8. Adebo OA. Oesophageal atresia and tracheo-oesophageal fistula: review of a 10-year personal experience. *West Afr J Med* 1990; **9**(3): 164-9.
9. Anwar ul H, Ubaidullah, Akhter N, et al. Factors affecting survival in patients with oesophageal atresia and tracheo-oesophageal fistula. *J Ayub Med Coll Abbottabad* 2009; **21**(4): 129-33.
10. Randriamizao HMR, Rakotondrainibe A, Rahanitriniaina NMP, Rajaonera AT, Andriamanarivo ML. [Intraoperative management of esophageal atresia: small steps that cannot be ignored in Madagascar]. *Pan Afr Med J* 2017; **27**: 9.
11. Singh A, Bajpai M, Bhatnagar V, Agarwala S, Srinivas M, Sharma N. Effect of number of associated anomalies on outcome in oesophageal atresia with or without tracheoesophageal fistula patient. *Afr J Paediatr Surg* 2013; **10**(4): 320-2.
12. Zhang Z, Huang Y, Su P, Wang D, Wang L. Experience in treating congenital esophageal atresia in China. *J Pediatr Surg* 2010; **45**(10): 2009-14.
13. Niramis R, Tangkhabuanbut P, Anuntkosol M, Buranakitjaroen V, Tongsin A, Mahatharadol V. Clinical outcomes of esophageal atresia: comparison between the Waterston and the Spitz classifications. *Ann Acad Med Singapore* 2013; **42**(6): 297-300.
14. Narasimman S, Nallusamy M, Hassan S. Review of oesophageal atresia and tracheoesophageal fistula in hospital sultanah bahiyah, alor star. Malaysia from january 2000 to december 2009. *Med J Malaysia* 2013; **68**(1): 48-51.
15. Bouguermouh D, Salem A. Esophageal atresia: a critical review of management at a single center in Algeria. *Dis Esophagus* 2015; **28**(3): 205-10.
16. Upadhyaya VD, Gangopadhyaya AN, Gupta DK, et al. Prognosis of congenital tracheoesophageal fistula with esophageal atresia on the basis of gap length. *Pediatr Surg Int* 2007; **23**(8): 767-71.
17. Tefera E, Teka T, Derbew M. Neonatal gastrointestinal surgical emergencies: a 5- year review in a teaching hospital Addis Ababa, Ethiopia. *Ethiop Med J* 2007; **45**(3): 251-6.
18. Sharma AK, Shukla AK, Prabhakar G, Sarin YK, Sharma CS. Esophageal atresia: tragedies and triumphs over two decades in a developing country. *Int Surg* 1993; **78**(4): 311-4.
19. Long AM, Bunch KJ, Knight M, Kurinczuk JJ, Losty PD, Baps C. Early population-based outcomes of infants born with congenital diaphragmatic hernia. *Arch Dis Child Fetal Neonatal Ed* 2018.
20. Logan JW, Rice HE, Goldberg RN, Cotten CM. Congenital diaphragmatic hernia: a systematic review and summary of best-evidence practice strategies. *J Perinatol* 2007; **27**(9): 535-49.
21. Emam SM, Kamel KH. Influence of pulmonary hypertension on outcome of Egyptian patients with congenital diaphragmatic hernia: an experience in low-resource settings. *J Egypt Soc Parasitol* 2012; **42**(2): 405-16.
22. Numanoglu A, Morrison C, Rode H. Prediction of outcome in congenital diaphragmatic hernia. *Pediatr Surg Int* 1998; **13**(8): 564-8.
23. Ozdogan T, Durakbasa C, Mutus M, Iscen M. Congenital diaphragmatic hernia: a 4-year experience in a single centre. *Afr J Paediatr Surg* 2010; **7**(2): 105-6.
24. Garcia AM, Machicado S, Gracia G, Zarante IM. Risk factors for congenital diaphragmatic hernia in the Bogota birth defects surveillance and follow-up program, Colombia. *Pediatr Surg Int* 2016; **32**(3): 227-34.
25. Rohana J, Boo NY, Thambidorai CR. Early outcome of congenital diaphragmatic hernia in a Malaysian tertiary centre. *Singapore Med J* 2008; **49**(2): 142-4.
26. Dehdashtian M, Bashirnejad S, Malekian A, Aramesh MR, Aletayeb MH. Seasonality, Epidemiology and Outcome of Congenital Diaphragmatic Hernia in South West of Iran. *J Neonatal Surg* 2017; **6**(2): 28.
27. Bhat YR, Kumar V, Rao A. Congenital diaphragmatic hernia in a developing country. *Singapore Med J* 2008; **49**(9): 715-8.
28. Burjonrappa S, Crete E, Bouchard S. Comparative outcomes in intestinal atresia: a clinical outcome and pathophysiology analysis. *Pediatr Surg Int* 2011; **27**(4): 437-42.
29. Gupta S, Gupta R, Ghosh S, et al. Intestinal Atresia: Experience at a Busy Center of North-West India. *J Neonatal Surg* 2016; **5**(4): 51.
30. Dalla Vecchia LK, Grosfeld JL, West KW, Rescorla FJ, Scherer LR, Engum SA. Intestinal atresia and stenosis: a 25-year experience with 277 cases. *Arch Surg* 1998; **133**(5): 490-6; discussion 6-7.
31. Chirdan LB, Uba AF, Pam SD. Intestinal atresia: management problems in a developing country. *Pediatr Surg Int* 2004; **20**(11-12): 834-7.
32. Cairo S, Kakembo N, Kisa P, et al. Disparity in access and outcomes for emergency neonatal surgery: intestinal atresia in Kampala, Uganda. *Pediatr Surg Int* 2017; **33**(8): 907-15.
33. Cox SG, Numanoglu A, Millar AJ, Rode H. Colonic atresia: spectrum of presentation and pitfalls in management. A review of 14 cases. *Pediatr Surg Int* 2005; **21**(10): 813-8.
34. Khan N, Bakht S, Zaheer N. A Minor Innovation in Constructing a Small Bowel Stoma in Neonates with Small Bowel Atresia to Reduce the Morbidity. *J Neonatal Surg* 2016; **5**(4): 45.
35. Ameh EA, Nmadu PT. Intestinal atresia and stenosis: a retrospective analysis of presentation, morbidity and mortality in Zaria, Nigeria. *West Afr J Med* 2000; **19**(1): 39-42.
36. Barrack SM, Kyambi JM, Ndungu J, Wachira N, Anangwe G, Safwat S. Intestinal atresia and stenosis as seen and treated at Kenyatta National Hospital, Nairobi. *East Afr Med J* 1993; **70**(9): 558-64.
37. Ekwunife OH, Oguejiofor IC, Modekwe VI, Osuigwe AN. Jejuno-ileal atresia: a 2-year preliminary study on presentation and outcome. *Niger J Clin Pract* 2012; **15**(3): 354-7.
38. Krishna A, Murali MV, Ahuja S, Kaur N. Factors influencing survival in esophageal atresia. *Indian Pediatr* 1994; **31**(1): 80-3.

39. PaedSurg Africa Research Collaboration. Paediatric Surgery across Sub-Saharan Africa: A Multi-Centre Prospective Cohort Study. https://clinicaltrials.gov/ct2/show/NCT03185637 (Accessed 31st May 2018).
40. Bradnock T, Marven S, Owen A, et al. Gastroschisis: one year outomes from national cohort study. *BMJ* 2011; **343**(d6749).
41. Askarpour S, Ostadian N, Javaherizadeh H, Chabi S. Omphalocele, gastroschisis: epidemiology, survival, and mortality in Imam Khomeini hospital, Ahvaz-Iran. *Pol Przegl Chir* 2012; **84**(2): 82-5.
42. Sekabira J, Hadley GP. Gastroschisis: a third world perspective. *Pediatr Surg Int* 2009; **25**(4): 327-9.
43. Wesonga AS, Fitzgerald TN, Kabuye R, et al. Gastroschisis in Uganda: Opportunities for improved survival. *J Pediatr Surg* 2016; **51**(11): 1772-7.
44. Apfeld JC, Wren SM, Macheka N, et al. Infant, maternal, and geographic factors influencing gastroschisis related mortality in Zimbabwe. *Surgery* 2015; **158**(6): 1475-80.
45. Allotey J, Davenport M, Njere I, et al. Benefit of preformed silos in the management of gastroschisis. *Pediatr Surg Int* 2007; **23**: 1065-9.
46. Ameh EA, Chirdan LB. Ruptured exomphalos and gastroschisis: a retrospective analysis of morbidity and mortality in Nigerian children. *Pediatr Surg Int* 2000; **16**(1-2): 23-5.
47. Weil BR, Leys CM, Rescorla FJ. The jury is still out: changes in gastroschisis management over the last decade are associated with both benefits and shortcomings. *J Pediatr Surg* 2012; **47**(1): 119-24.
48. Lansdale N, Hill R, Gull-Zamir S, et al. Staged reduction of gastroschisis using preformed silos: practicalities and problems. *J Pediatr Surg* 2009; **44**(11): 2126-9.
49. Ford K, Poenaru D, Moulot O, et al. Gastroschisis: Bellwether for neonatal surgery capacity in low resource settings? *J Pediatr Surg* 2016; **51**(8): 1262-7.
50. Du L, Pan WH, Cai W, Wang J, Wu YM, Shi CR. Delivery room surgery: an applicable therapeutic strategy for gastroschisis in developing countries. *World J Pediatr* 2014; **10**(1): 69-73.
51. Erdogan D, Azili MN, Cavusoglu YH, et al. 11-year experience with gastroschisis: factors affecting mortality and morbidity. *Iran J Pediatr* 2012; **22**(3): 339-43.
52. Manson J, Ameh E, Canvassar N, et al. Gastroschisis: a multi-centre comparison of management and outcome. *Afr J Paediatr Surg* 2012; **9**(1): 17-21.
53. Saranrittichai S. Gastroschisis: delivery and immediate repair in the operating room. *J Med Assoc Thai* 2008; **91**(5): 686-92.
54. Abdur-Rahman LO, Abdulrasheed NA, Adeniran JO. Challenges and outcomes of management of anterior abdominal wall defects in a Nigerian tertiary hospital. *Afr J Paediatr Surg* 2011; **8**(2): 159-63.
55. Sakonidou S, Ali K, Farmer I, Hickey A, Greenough A. Mortality and short-term morbidity in infants with exomphalos. *Pediatr Int* 2018.
56. Hsu CC, Lin SP, Chen CH, et al. Omphalocele and gastroschisis in Taiwan. *Eur J Pediatr* 2002; **161**(10): 552-5.
57. Conner P, Vejde JH, Burgos CM. Accuracy and impact of prenatal diagnosis in infants with omphalocele. *Pediatr Surg Int* 2018.
58. Kouame BD, Dick RK, Ouattara O, et al. [Therapeutic approaches for omphalocele in developing countries: experience of Central University Hospital of Yopougon, Abidjan, Cote d'Ivoire]. *Bull Soc Pathol Exot* 2003; **96**(4): 302-5.
59. Osifo OD, Ovueni ME, Evbuomwan I. Omphalocele management using goal-oriented classification in African centre with limited resources. *J Trop Pediatr* 2011; **57**(4): 286-8.
60. Kong JY, Yeo KT, Abdel-Latif ME, et al. Outcomes of infants with abdominal wall defects over 18years. *J Pediatr Surg* 2016; **51**(10): 1644-9.
61. Groves R, Sunderajan L, Khan AR, Parikh D, Brain J, Samuel M. Congenital anomalies are commonly associated with exomphalos minor. *J Pediatr Surg* 2006; **41**(2): 358-61.
62. Na Q, Liu C, Cui H, Zhang Z, Yin S, Li Q. Immediate repair compared with delayed repair of congenital omphalocele: short-term neonatal outcomes in China. *J Int Med Res* 2011; **39**(6): 2344-51.
63. Tarca E, Aprodu S. Past and present in omphalocele treatment in Romania. *Chirurgia (Bucur)* 2014; **109**(4): 507-13.
64. Kante L, Togo A, Diakite I, et al. [Omphalocele in general and pediatric surgery in Gabriel Toure]. *Mali Med* 2010; **25**(3): 23-6.
65. Ngom G, Fall I, Sankale AA, et al. [Evaluation of the management of omphalocele at Dakar]. *Dakar Med* 2004; **49**(3): 203-6.
66. Sabetay C, Plesea E, Ferschin A, Sabetay E, Stoica A, Singer I. [Follow-up evaluation of omphalocele treatment in children. The experience of the department of Pediatric Surgery and Orthopedics No.1 University Hospital Craiova]. *Chirurgia (Bucur)* 2001; **96**(2): 177-85.
67. Rintala RJ, Pakarinen MP. Imperforate anus: long- and short-term outcome. *Semin Pediatr Surg* 2008; **17**(2): 79-89.
68. Haider N, Fisher R. Mortality and morbidity associated with late diagnosis of anorectal malformations in children. *Surgeon* 2007; **5**(6): 327-30.
69. Ekenze SO, Ibeziako SN, Ezomike UO. Trends in neonatal intestinal obstruction in a developing country, 1996-2005. *World J Surg* 2007; **31**(12): 2405-9; discussion 10-1.
70. Chirdan LB, Uba FA, Ameh EA, Mshelbwala PM. Colostomy for high anorectal malformation: an evaluation of morbidity and mortality in a developing country. *Pediatr Surg Int* 2008; **24**(4): 407-10.
71. Chowdhary SK, Chalapathi G, Narasimhan KL, et al. An audit of neonatal colostomy for high anorectal malformation: the developing world perspective. *Pediatr Surg Int* 2004; **20**(2): 111-3.
72. Govender S, Wiersma R. Delayed diagnosis of anorectal malformations (ARM): causes and consequences in a resource-constrained environment. *Pediatr Surg Int* 2016; **32**(4): 369-75.
73. Ameh EA, Chirdan LB. Neonatal intestinal obstruction in Zaria, Nigeria. *East Afr Med J* 2000; **77**(9): 510-3.
74. Mirza B, Ijaz L, Saleem M, Sharif M, Sheikh A. Anorectal malformations in neonates. *Afr J Paediatr Surg* 2011; **8**(2): 151-4.
75. Lukong CS, Ameh EA, Mshelbwala PM, et al. Management of anorectal malformation: Changing trend over two decades in Zaria, Nigeria. *Afr J Paediatr Surg* 2011; **8**(1): 19-22.
76. Archibong AE, Idika IM. Results of treatment in children with anorectal malformations in Calabar, Nigeria. *S Afr J Surg* 2004; **42**(3): 88-90.
77. Bradnock TJ, Knight M, Kenny S, Nair M, Walker GM, British Association of Paediatric Surgeons Congenital Anomalies Surveillance S. Hirschsprung's disease in the UK and Ireland: incidence and anomalies. *Arch Dis Child* 2017; **102**(8): 722-7.
78. Liem NT, Hau BD. One-stage operation for Hirschsprung's disease: experience with 192 cases. *Asian J Surg* 2008; **31**(4): 216-9.
79. Pini Prato A, Rossi V, Avanzini S, Mattioli G, Disma N, Jasonni V. Hirschsprung's disease: what about mortality? *Pediatr Surg Int* 2011; **27**(5): 473-8.
80. Taguchi T, Obata S, Ieiri S. Current status of Hirschsprung's disease: based on a nationwide survey of Japan. *Pediatr Surg Int* 2017; **33**(4): 497-504.

Supplementary Table 1: Characteristics, perioperative care, surgical interventions, and outcomes for patients with oesophageal atresia

Variable	Total (n=560)	HIC (n=141)	MIC (n=412)	LIC (n=7)	P value
**Patient Characteristics:**					
Median gestational age at birth (IQR), weeks	37 (4)	37 (4)	37 (2)	38 (2)	0·621
Median age at presentation (IQR), hours	19 (46)	5 (20)	24 (68)	144 (200)	**<0·001**
Sex:					
Male	314 (56·1%)	91 (64·5%)	223 (54·1%)	0 (0·0%)	**<0·001**
Female	242 (43·2%)	50 (35·5%)	186 (45·1%)	6 (85·7%)	-
Ambiguous	4 (0·7%)	0 (0·0%)	3 (0·7%)	1 (14·3%)	-
Median weight at presentation (IQR), kg	2·5 (0·9)	2·5 (1.0)	2·5 (0·8)	2·2 (1·2)	0·778
Does the patient have another anomaly in addition to the study condition?					
Yes: Cardiovascular	268 (47·9%)	70 (49·6%)	195 (47·3%)	3 (42·9%)	0·862
Yes: Respiratory	44 (7·9%)	11 (7·8%)	32 (7·8%)	1 (14·3%)	0·817
Yes: Gastrointestinal	72 (12·9%)	22 (15·6%)	50 (12·1%)	0 (0·0%)	0·337
Yes: Neurological	28 (5·0%)	13 (9·2%)	15 (3·6%)	0 (0·0%)	**0·027**
Yes: Genito-urinary	70 (12·5%)	28 (19·9%)	42 (10·2%)	0 (0·0%)	**0·007**
Yes: Musculoskeletal	62 (11·1%)	24 (17·0%)	38 (9·2%)	0 (0·0%)	**0·025**
Yes: Down syndrome	9 (1·6%)	3 (2·1%)	6 (1·5%)	0 (0·0%)	0·813
Yes: Beckwith Wiedemann syndrome	0 (0·0%)	0 (0·0%)	0 (0·0%)	0 (0·0%)	-
Yes: Cystic fibrosis	1 (0·2%)	0 (0·0%)	1 (0·2%)	0 (0·0%)	0·835
Yes: Chromosomal	20 (3·6%)	10 (7·1%)	10 (2·4%)	0 (0·0%)	**0·032**
Yes: Other	40 (7·1%)	14 (9·9%)	26 (6·3%)	0 (0·0%)	0·270
No	198 (35·4%)	45 (31·9%)	149 (36·2%)	4 (57·1%)	0·316
Median distance from patient's home to hospital (IQR), km*	30 (95)	17 (92)	31 (106)	92 (165)	**0·026**
Type of delivery:					
Vaginal (spontaneous)	222 (39·6%)	53 (37·6%)	163 (39·6%)	6 (85·7%)	**0·002**
Vaginal (induced)	32 (5·7%)	15 (10·6%)	17 (4·1%)	0 (0·0%)	-
Caesarean section (elective)	145 (25·9%)	22 (15·6%)	123 (29·9%)	0 (0·0%)	-
Caesarean section (urgent/non-elective)	157 (28·0%)	51 (36·2%)	105 (25·5%)	1 (14·3%)	-
Unknown	3 (0·5%)	0 (0·0%)	3 (0·7%)	0 (0·0%)	-
Missing	1 (0·2%)	0 (0·0%)	1 (0·2%)	0 (0·0%)	-
Was the patient septic on arrival to your hospital?					
Yes	124 (22·1%)	10 (7·1%)	110 (26·7%)	4 (57·1%)	**<0·001**
No	436 (77·9%)	131 (92·9%)	302 (73·3%)	3 (42·9%)	-
Was the patient hypovolaemic on arrival to your hospital?					
Yes	77 (13·8%)	12 (8·5%)	64 (15·5%)	1 (14·3%)	0·112
No	483 (86·3%)	129 (91·5%)	348 (84·5%)	6 (85·7%)	-
Was the patient hypothermic on arrival to your hospital?					
Yes	69 (12·3%)	5 (3·5%)	60 (14·6%)	4 (57·1%)	**<0·001**
No	490 (87·5%)	136 (96·5%)	351 (85·2%)	3 (42·9%)	-
Missing	1 (0·2%)	0 (0·0%)	1 (0·2%)	0 (0·0%)	-
American Society of Anaesthesiologists (ASA) Score at the time of primary intervention:					
1. Healthy person	55 (9·8%)	11 (7·8%)	44 (10·7%)	0 (0·0%)	**<0·001**
2. Mild systemic disease	168 (30·0%)	32 (22·7%)	136 (33·0%)	0 (0·0%)	-
3. Severe systemic disease	166 (29·6%)	58 (41·1%)	105 (25·5%)	3 (42·9%)	-
4. Severe systemic disease that is a constant threat to life	100 (17·9%)	32 (22·7%)	66 (16·0%)	2 (28·6%)	-
5. A moribund patient who is not expected to survive without the operation	31 (5·5%)	2 (1·4%)	29 (7·0%)	0 (0·0%)	-
Not applicable - no intervention	37 (6·6%)	4 (2·8%)	31 (7·5%)	2 (28·6%)	-
Missing	3 (0·5%)	2 (1·4%)	1 (0·2%)	0 (0·0%)	-
What study condition does the patient have?					
Oesophageal atresia	560 (100·0%)	141 (100·0%)	412 (100·0%)	7 (100·0%)	-
Congenital diaphragmatic hernia	1 (0·2%)	1 (0·7%)	0 (0·0%)	0 (0·0%)	0·226
Intestinal atresia	18 (3·2%)	7 (5·0%)	11 (2·7%)	0 (0·0%)	0·365
Gastroschisis	0 (0·0%)	0 (0·0%)	0 (0·0%)	0 (0·0%)	-
Exomphalos/Omphalocele	3 (0·5%)	1 (0·7%)	2 (0·5%)	0 (0·0%)	0·934
Anorectal malformation	53 (9·5%)	10 (7·1%)	42 (10·2%)	1 (14·3%)	**0·504**
Hirschsprung's Disease	0 (0·0%)	0 (0·0%)	0 (0·0%)	0 (0·0%)	-
Type of OA +/- TOF (Gross classification)					
Without a fistula	47 (8·4%)	14 (9·9%)	33 (8·0%)	0 (0·0%)	0·147
Proximal TOF, distal OA	10 (1·8%)	4 (2·8%)	5 (1·2%)	1 (14·3%)	-
Distal TOF with proximal OA	476 (85·0%)	114 (80·9%)	356 (86·4%)	6 (85·7%)	-
Proximal and distal TOF	8 (1·4%)	4 (2·8%)	4 (1·0%	0 (0·0%)	-
H-type TOF without OA	19 (3·4%)	5 (3·6%)	14 (3·4%)	0 (0·0%)	-
Long or short gap?					
Long	111 (19·8%)	26 (18·4%)	85 (20·6%)	0 (0·0%)	**<0·001**
Short	375 (67·0%)	99 (70·2%)	275 (66·8%)	1 (14·3%)	-
Unknown	74 (13·2%)	16 (11·4%)	52 (12·6%)	6 (85·7%)	-
Pneumonia at presentation?					
Yes: diagnosed clinically	100 (17·9%)	3 (2·1%)	91 (22·1%)	6 (85·7%)	**<0·001**
Yes: diagnosed radiologically	86 (15·4%)	3 (2·1%)	83 (20·1%)	0 (0·0%)	-
Yes: other means of diagnosis	0 (0·0%)	0 (0·0%)	0 (0·0%)	0 (0·0%)	-
No: patient born in the study centre	123 (22·0%)	47 (33·3%)	76 (18·4%)	0 (0·0%)	-
No: patient born outside the study centre but no evidence of pneumonia on arrival	251 (44·8%)	88 (62·4%)	162 (39·3%)	1 (14·3%)	-

Did the patient have tracheomalacia?					
Yes: diagnosed clinically	34 (6·1%)	11 (7·8%)	23 (5·6%)	0 (0·0%)	0·505
Yes: diagnosed on bronchoscopy	38 (6·8%)	21 (14·9%)	17 (4·3%)	0 (0·0%)	**<0·001**
Yes: diagnosed on CT	0 (0·0%)	0 (0·0%)	0 (0·0%)	0 (0·0%)	-
Yes: diagnosed on bronchogram	2 (0·4%)	1 (0·7%)	1 (1·0%)	0 (0·0%)	0·716
Yes: other method of diagnosis	0 (0·0%)	0 (0·0%)	0 (0·0%)	0 (0·0%)	-
No	486 (87·0%)	108 (76·6%)	371 (90·0%)	7 (100·0%)	**<0·001**
**Care prior to presentation at the paediatric surgery centre:**					
Antenatal ultrasound undertaken?					
Yes: study condition diagnosed	65 (11·6%)	20 (14·2%)	45 (10·9%)	0 (0·0%)	**<0·001**
Yes: problem identified but study condition not diagnosed	126 (22·5%)	52 (36·9%)	73 (17·7%)	1 (14·3%)	-
Yes: no problem identified	289 (51·6%)	58 (41·1%)	226 (54·9%)	5 (71·4%)	-
No	79 (14·1%)	11 (7·8%)	67 (16·3%)	1 (14·3%)	-
Missing	1 (0·2%)	0 (0·0%)	1 (0·2%)	0 (0·0%)	-
Median gestational age of study condition diagnosis if diagnosis was antenatal (IQR), weeks	29 (8)	29·5 (11)	29 (6)	-	0·293
Mode of transport to hospital:					
Ambulance	314 (56·1%)	85 (60·3%)	225 (54·6%)	4 (57·1%)	**<0·001**
Other transport provided by the health service	39 (7·0%)	10 (7·1%)	28 (6·8%)	1 (14·3%)	-
Patient's own transport	73 (13·0%)	2 (1·4%)	69 (16·8%)	2 (28·6%)	-
Born within the hospital	133 (23·8%)	44 (31·2%)	89 (21·6%)	0 (0·0%)	-
Missing	1 (0·2%)	0 (0·0%)	1 (0·2%)	0 (0·0%)	-
If outborn, where did the patient present from?					
Home	17 (4·0%)	1 (1·0%)	15 (4·7%)	1 (14·3%)	**0·019**
Community Clinic/General Practice	66 (15·5%)	5 (5·2%)	59 (18·3%)	2 (28·6%)	-
District Hospital	338 (79·3%)	90 (92·8%)	244 (75·8%)	4 (57·1%)	-
From another country	1 (0·2%)	0 (0·0%)	1 (0·3%)	0 (0·0%)	-
From a different speciality within the hospital	1 (0·2%)	1 (1·0%)	0 (0·0%)	0 (0·0%)	-
Unknown	3 (0·7%)	0 (0·0%)	3 (0·9%)	0 (0·0%)	-
**Perioperative care at the paediatric surgery centre:**					
If septic, were appropriate antibiotics administered?					
Yes within 1 hour of arrival	100 (80·6%)	7 (70·0%)	91 (82·7%)	2 (50·0%)	0·407
Yes within the first day of arrival	23 (18·5%)	3 (30·0%)	18 (16·4%)	2 (50·0%)	-
No	1 (0·8%)	0 (0·0%)	1 (0·9%)	0 (0·0%)	-
If hypovolaemic, was an intravenous fluid bolus given?					
Yes within 1 hour of arrival	56 (72·7%)	5 (41·7%)	51 (79·7%)	0 (0·0%)	**<0·001**
Yes within the first day of arrival	19 (24·7%)	7 (58·3%)	12 (18·8%)	0 (0·0%)	-
No	2 (2·6%)	0 (0·0%)	1 (1·6%)	1 (100·0%)	-
If hypovolaemic, how much intravenous fluid was given?					
10 - 20mls/kg	57 (76·0%)	8 (66·7%)	49 (77·8%)	0 (0·0%)	0·409
Above 20mls/kg	18 (24·0%)	4 (33·3%)	14 (22·2%)	0 (0·0%)	-
If hypothermic, was the patient warmed on arrival to your hospital to within a normal temperature range?					
Yes	64 (92·8%)	5 (100·0%)	55 (91·7%)	4 (100·0%)	0·667
No	5 (7·2%)	0 (0·0%)	5 (8·3%)	0 (0·0%)	-
Did the patient receive central venous access?					
Yes: umbilical catheter	74 (13·2%)	30 (21·3%)	44 (10·7%)	0 (0·0%)	**0·003**
Yes: peripherally inserted central catheter (PICC)	179 (32·0%)	60 (42·6%)	119 (28·9%)	0 (0·0%)	**0·002**
Yes: percutaneously inserted central line with ultrasound guidance	92 (16·4%)	42 (29·8%)	50 (12·1%)	0 (0·0%)	**<0·001**
Yes: surgically placed central line (open insertion)	60 (10·7%)	2 (1·4%)	58 (14·1%)	0 (0·0%)	**<0·001**
No	195 (34·8%)	23 (16·3%)	165 (40·1%)	7 (100%)	**<0·001**
Median total duration of antibiotics following primary intervention (IQR), days	7 (12)	5 (6)	10 (11)	0 (3)	**<0·001**
Did the patient receive a blood transfusion?					
Yes: not cross-matched.	11 (2·0%)	3 (2·1%)	8 (1·9%)	0 (0·0%)	**<0·001**
Yes: cross-matched.	246 (43·9%)	36 (25·5%)	208 (50·5%)	2 (28·6%)	-
No: not required.	295 (52·7%)	100 (70·9%)	190 (46·1%)	5 (71·4%)	-
No: it was required but not available.	7 (1·3%)	1 (0·7%)	6 (1·5%)	0 (0·0%)	-
Missing	1 (0·2%)	1 (0·7%)	0 (0·0%)	0 (0·0%)	-
Did the patient require ventilation?					
Yes and it was given	475 (84·8%)	137 (97·2%)	338 (82·0%)	0 (0·0%)	**<0·001**
Yes, but it was not available	14 (2·5%)	0 (0·0%)	10 (2·4%)	4 (57·1%)	-
No	71 (12·7%)	4 (2·8 %)	64 (15·5 %)	3 (42·9%)	-
Median time patient remained on ventilation if given (IQR), days	5 (6)	5 (5)	5 (7)	-	0·578
Median time to first enteral feed (post-primary intervention) (IQR), days	6 (6)	5 (5)	6 (6)	10 (0)	0·060
Median time to full enteral feeds (post-primary intervention) (IQR), days	12 (11)	12 (12)	12 (11)	-	0·977
Median time to first oral feed post-operatively (IQR), days	7 (6)	8 (5)	7 (6)	-	0·654
Median time to full oral feeds (IQR), days	14 (12)	15 (21)	14 (10)	-	**0·031**
Did the patient require parenteral nutrition?					
Yes and it was given	398 (71·1%)	122 (86·5%)	276 (67·0%)	0 (0·0%)	**<0·001**
Yes and it was sometimes available, but less than required	14 (2·5%)	0 (0·0%)	14 (3·4%)	0 (0·0%)	-
Yes, but it was not available	23 (4·1%)	0 (0·0%)	19 (4·6%)	4 (57·1%)	-
No	125 (22·3%)	19 (13·5%)	103 (25·0%)	3 (42·9%)	-
Median time patient received parenteral nutrition if received (IQR), days	12 (10)	12 (13)	12 (9)	-	0·468
If the patient had a primary oesophageal anastomosis, was a post-operative oesophagogram undertaken?					
Yes	272 (71·2%)	93 (84·5%)	179 (65·8%)	0 (0·0%)	**<0·001**
No	110 (28·8%)	17 (15·5%)	93 (34·2%)	0 (0·0%)	-
If yes, routine or clinically indicated?					

Routine	234 (86·0%)	85 (91·4%)	149 (83·2%)	0 (0·0%)	0·066
Clinically indicated	38 (14·0%)	8 (8·6%)	30 (16·8%)	0 (0·0%)	-
Median number of days until post-operative oesophagogram undertaken, if undertaken (IQR)	7 (3)	7 (3)	7 (2)	-	0·335
Result of post-operative oesophagogram:					
Leak	52 (19·2%)	14 (15·1%)	38 (21·3%)	0 (0·0%)	0·212
No leak	219 (80·8%)	79 (84·9%)	140 (78·7%)	0 (0·0%)	-
For patients diagnosed with a leak radiologically, was it associated with clinical symptoms?					
Yes	35 (68·6%)	11 (78·6%)	24 (64·9%)	0 (0·0%)	0·346
No	16 (31·4%)	3 (21·4%)	13 (35·1%)	0 (0·0%)	-
**Surgical intervention:**					
Primary intervention:					
Oesophageal anastomosis	385 (68·8%)	110 (78·0%)	275 (68·6%)	0 (0·0%)	**<0·001**
TOF ligation	341 (60·9%)	106 (75·2%)	235 (57·0%)	0 (0·0%)	**<0·001**
Gastrostomy	108 (19·3%)	21 (14·9%)	84 (20·4%)	3 (42·9%)	0·102
Palliative care	50 (8·9%)	4 (2·8%)	42 (10·2%)	4 (57·1%)	**<0·001**
Oesophagostomy	42 (7·5%)	3 (2·1%)	39 (9·5%)	0 (0·0%)	**0·013**
Ligation of the distal oesophagus	16 (2·9%)	0 (0·0%)	16 (3·9%)	0 (0·0%)	0·052
Foker technique	4 (0·7%)	1 (1·0%)	3 (1·0%)	0 (0·0%)	0·975
Fundoplication	0 (0·0%)	0 (0·0%)	0 (0·0%)	0 (0·0%)	-
Gastro-oesophageal disconnection	0 (0·0%)	0 (0·0%)	0 (0·0%)	0 (0·0%)	-
Other	10 (1·8%)	3 (2·1%)	7 (7·7%)	0 (0·0%)	0·887
Median time from arrival at your hospital to primary intervention (IQR), hours	35 (54)	23 (19)	48 (68)	96 (96)	**<0·001**
Surgical approach?					
Thoracotomy muscle cutting	212 (40·4%)	37 (27·0%)	175 (45·6%)	0 (0·0%)	**<0·001**
Thoracotomy muscle splitting	147 (28·0%)	48 (35·0%)	98 (25·5%)	1 (25·0%)	-
Thoracoscopy	92 (17·5%)	39 (28·5%)	53 (13·8%)	0 (0·0%)	-
Laparotomy	29 (5·5%)	6 (4·4%)	21 (5·5%)	2 (50·0%)	-
Limited local incision	14 (2·7%)	2 (1·5%)	12 (3·1%)	0 (0·0%)	-
Laparoscopy	3 (0·6%)	1 (0·7%)	2 (0·5%)	0 (0·0%)	-
Cervical approach	2 (0·4%)	1 (0·7%)	1 (0·3%)	0 (0·0%)	-
Not applicable/no intervention	20 (3·8%)	1 (0·7%)	18 (4·7%)	1 (25·0%)	-
Other	1 (0·2%)	0 (0·0%)	1 (0·3%)	0 (0·0%)	-
Unknown	5 (1·0%)	2 (1·5%)	3 (0·8%)	0 (0·0%)	-
If thoracoscopic/ laparoscopic, was the operation converted to open?					
Yes	10 (10·6%)	5 (12·5%)	5 (9·3%)	0 (0·0%)	0·614
No	84 (89·4%)	35 (87·5%)	49 (90·7%)	0 (0·0%)	-
What type of anaesthesia was used for the primary intervention?					
General anaesthesia with endotracheal tube	506 (90·4%)	136 (96·5%)	368 (89·3%)	2 (28·6%)	**<0·001**
Ketamine anaesthesia	2 (0·4%)	0 (0·0%)	1 (0·2%)	1 (14·3%)	-
General anaesthesia with laryngeal airway	1 (0·2%)	0 (0·0%)	1 (0·2%)	0 (0·0%)	-
Local anaesthesia only	1 (0·2%)	0 (0·0%)	1 (0·2%)	0 (0·0%)	-
No anaesthesia, just analgesia	1 (0·2%)	1 (0·7%)	0 (0·0%)	0 (0·0%)	-
Spinal/caudal anaesthesia	0 (0·0%)	0 (0·0%)	0 (0·0%)	0 (0·0%)	-
No anaesthesia, no analgesia	0 (0·0%)	0 (0·0%)	0 (0·0%)	0 (0·0%)	-
Not applicable: no surgery or primary intervention undertaken.	49 (8·8%)	4 (2·8%)	41 (10·0%)	4 (57·1%)	-
Who undertook the anaesthetic for the primary intervention?					
Anaesthetic doctor	506 (90·4%)	136 (96·5%)	368 (89·3%)	2 (28·6%)	**<0·001**
Surgeon	2 (0·4%)	0 (0·0%)	2 (0·5%)	0 (0·0%)	-
Anaesthetic nurse	1 (0·2%)	0 (0·0%)	1 (0·2%)	0 (0·0%)	-
Medical officer	1 (0·2%)	0 (0·0%)	0 (0·0%)	1 (14·3%)	-
Other healthcare professional	0 (0·0%)	0 (0·0%)	0 (0·0%)	0 (0·0%)	-
No anaesthetic undertaken	50 (8·9%)	5 (3·6%)	41 (10·0%)	4 (57·1%)	-
Who undertook the primary intervention?					
Paediatric surgeon (or junior with paediatric surgeon assisting/in the room)	508 (90·7%)	134 (95·0%)	372 (90·3%)	2 (28·6%)	**<0·001**
General surgeon (or junior with general surgeon assisting/in the room)	4 (0·7%)	3 (2·1%)	1 (0·2%)	0 (0·0%)	-
Trainee surgeon (without a paediatric or general surgeon assisting or in the room)	1 (0·2%)	0 (0·0%)	0 (0·0%)	1 (14·3%)	-
Junior doctor, medical officer or other (without a paediatric or general surgeon assisting/in the room)	0 (0·0%)	0 (0·0%)	0 (0·0%)	0 (0·0%)	-
Not applicable - no surgery or primary intervention undertaken.	47 (8·4%)	4 (2·8%)	39 (9·5%)	4 (57·1%)	-
Was a Surgical Safety Checklist used at the time of primary intervention?					
Yes	423 (75·5%)	131 (92·9%)	290 (70·4%)	2 (28·6%)	**<0·001**
No: but it was available	49 (8·8%)	4 (2·8%)	43 (10·4%)	2 (28·6%)	-
No: it was not available	41 (7·3%)	2 (1·4%)	39 (9·5%)	0 (0·0%)	-
Not applicable: a conservative primary intervention was undertaken	5 (0·9%)	0 (0·0%)	5 (1·2%)	0 (0·0%)	-
Not applicable: no surgery or primary intervention undertaken	42 (7·5%)	4 (2·8%)	35 (8·5%)	3 (42·9%)	-
For patients not receiving a primary oesophageal anastomosis, at what age is definitive surgery planned? Median months (IQR)	3 (6)	3 (4)	3 (6)	-	0·935
For patients not receiving a primary oesophageal anastomosis, what is the future planned procedure?					
Primary oesophageal anastomosis if possible	66 (11·8%)	17 (12·1%)	48 (11·7%)	1 (14·3%)	0·971
Gap assessment	30 (5·4%)	6 (4·3%)	24 (5·8%)	0 (0·0%)	0·634
Colonic interposition	20 (3·6%)	1 (0·7%)	19 (4·6%)	0 (0·0%)	0·086
Gastric pull-up	18 (3·2%)	1 (0·7%)	17 (4·1%)	0 (0·0%)	0·124
H fistula - no further intervention planned	3 (0·5%)	2 (1·4%)	1 (0·2%)	0 (0·0%)	0·251
Ligation of TOF	2 (0·4%)	2 (1·4%)	0 (0·0%)	0 (0·0%)	0·051
Jejunal interposition	0 (0·0%)	0 (0·0%)	0 (0·0%)	0 (0·0%)	-
Other	4 (0·7%)	1 (0·7%)	2 (0·5%)	1 (14·3%)	**<0·001**

Not applicable, primary anastomosis undertaken	253 (45·2%)	88 (62·4%)	164 (39·8%)	1 (14·3%)	**<0·001**
Not applicable, patient died	5 (0·9%)	0 (0·0%)	5 (1·2%)	0 (0·0%)	0·404
Unknown	46 (8·2%)	5 (3·5%)	40 (9·7%)	1 (14·3%)	0·060
If the patient had tracheomalacia, was an intervention undertaken?					
Yes: aortopexy	0 (0·0%)	0 (0·0%)	0 (0·0%)	0 (0·0%)	0·614
Yes: tracheostomy	3 (4·2%)	2 (6·3%)	1 (2·6%)	0 (0·0%)	-
Yes: tracheal stent	0 (0·0%)	0 (0·0%)	0 (0·0%)	0 (0·0%)	-
Yes: supportive management (oxygen +/- ventilation) only	17 (23·9%)	7 (21·9%)	10 (25·6%)	0 (0·0%)	-
Yes: other treatment	2 (2·8%)	1 (3·1%)	1 (2·6%)	0 (0·0%)	-
No	49 (69·0%)	22 (68·8%)	27 (69·2%)	0 (0·0%)	-
**Outcomes:**					
Did the patient survive to discharge (or 30-days if still an in-patient 30-days following primary intervention)?					
Yes	423 (75·5%)	131 (92·9%)	291 (70·6%)	1 (14·3%)	**<0·001**
No	137 (24·5%)	10 (7·1%)	121 (29·4%)	6 (85·7%)	
If the patient was discharged prior, were they still alive at 30-days following primary intervention?					
Yes	385 (91·0%)	125 (95·4%)	260 (89·3%)	0 (0·0%)	**<0·001**
No	2 (0·5%)	0 (0·0%)	2 (0·7%)	0 (0·0%)	-
Not followed-up after discharge	19 (4·5%)	0 (0·0%)	19 (6·5%)	0 (0·0%)	-
Followed-up, but not until 30-days post primary intervention	17 (4·0%)	6 (4·6%)	10 (3·4%)	1 (100·0%)	-
Cause of mortality:					
Sepsis	44 (31·7%)	1 (10·0%)	42 (34·1%)	1 (16·7%)	0·069
Respiratory failure	38 (27·3%)	3 (30·0%)	33 (26·8%)	2 (33·3%)	-
Cardiac failure	21 (15·1%)	3 (30·0%)	17 (13·8%)	1 (16·7%)	-
Aspiration pneumonia	19 (13·7%)	0 (0·0%)	18 (14·6%)	1 (16·7%)	-
Haemorrhage	5 (3·6%)	1 (10·0%)	4 (3·3%)	0 (0·0%)	-
Syndrome incompatible with life	3 (2·2%)	2 (20·0%)	1 (0·8%)	0 (0·0%)	-
Recurrent tracheo-oesophageal fistula	1 (0·7%)	0 (0·0%)	1 (0·8%)	0 (0·0%)	-
Anastomotic leak	1 (0·7%)	0 (0·0%)	1 (0·8%)	0 (0·0%)	-
Other	7 (5·0%)	0 (0·0%)	6 (4·9%)	1 (16·7%)	-
Median duration of hospital stays, (IQR) days	18 (18)	23 (17)	18 (17)	6 (17)	**0·001**
Did the patient have a surgical site infection?					
Yes	63 (11·3%)	10 (7·1%)	53 (12·9%)	0 (0·0%)	**<0·001**
No	443 (79·1%)	128 (90·8%)	312 (75·7%)	3 (42·9%)	-
Not applicable, no surgical wound	54 (9·6%)	3 (2·1%)	47 (11·4%)	4 (57·1%)	-
Did the patient have a full thickness wound dehiscence?					
Yes	7 (1·3%)	1 (0·7%)	6 (1·5%)	0 (0·0%)	**<0·001**
No	497 (88·8%)	137 (97·2%)	357 (86·7%)	3 (42·9%)	-
Not applicable, no surgical wound	56 (10·0%)	3 (2·1%)	49 (11·9%)	4 (57·1%)	-
Did the patient require a further unplanned intervention?					
Yes – percutaneous	19 (3·4%)	9 (6·4%)	10 (2·4%)	0 (0·0%)	**<0·001**
Yes – surgical intervention	52 (9·3%)	14 (9·9%)	38 (9·2%)	0 (0·0%)	-
No	443 (79·1%)	115 (81·6%)	325 (78·9%)	3 (42·9%)	-
Not applicable – no primary intervention undertaken	46 (8·2%)	3 (2·1%)	39 (9·5%)	4 (57·1%)	-
If a central line was inserted, did the patient acquire central line sepsis?					
Yes, diagnosed clinically	12 (3·3%)	1 (0·8%)	11 (4·4%)	0 (0·0%)	0·170
Yes, confirmed on microbiology	24 (6·5%)	9 (7·6%)	15 (6·0%)	0 (0·0%)	-
No	332 (90·2%)	109 (91·6%)	223 (89·6%)	0 (0·0%)	-
Did the patient have a condition specific complication within 30-days of primary intervention?					
Pneumonia	117 (20·9%)	12 (8·5%)	104 (25·2%)	1 (14·3%)	**<0·001**
Anastomotic leak	63 (11·3%)	13 (9·2%)	49 (11·9%)	1 (14·3%)	0·665
Pneumothorax	57 (10·2%)	20 (14·2%)	37 (9·0%)	0 (0·0%)	0·141
Mediastinitis	37 (6·6%)	8 (5·7%)	29 (7·04%)	0 (0·0%)	0·664
Anastomotic stricture	27 (4·8%)	13 (9·2%)	14 (3·4%)	0 (0·0%)	**0·017**
Recurrent TOF	10 (1·8%)	3 (2·1%)	7 (1·7%)	0 (0·0%)	0·887
Chylothorax	6 (1·1%)	2 (1·4%)	4 (1·0%)	0 (0·0%)	0·871
Haemothorax	3 (0·5%)	1 (1·0%)	2 (0·5%)	0 (0·0%)	0·934
NEC	3 (0·5%)	0 (0·0%)	3 (0·7%)	0 (0·0%)	0·582
Left vocal cord paralysis/recurrent laryngeal nerve palsy	2 (0·4%)	2 (1·4%)	0 (0·0%)	0 (0·0%)	0·051
Ligation of a bronchus	1 (0·2%)	0 (0·0%)	1 (0·2%)	0 (0·0%)	0·835
Paralysis of the diaphragm	1 (0·2%)	0 (0·0%)	1 (0·2%)	0 (0·0%)	0·835
Other	18 (3·2%)	5 (3·6%)	13 (3·2%)	0 (0·0%)	0·866
N/A, no intervention	4 (0·7%)	1 (0·7%)	2 (0·5%)	1 (14·3%)	**<0·001**
None	285 (50·9%)	83 (58·9%)	200 (48·5%)	2 (28·6%)	0·053
Was the patient followed up at 30-days post primary surgery or intervention to a assess for complications?					
Yes: reviewed in person	231 (54·7%)	65 (50·0%)	166 (57·0%)	0 (0·0%)	**0·012**
Yes: via telephone consultation	31 (7·3%)	5 (3·8%)	26 (8·9%)	0 (0·0%)	-
Yes: via other means	15 (3·6%)	2 (1·5%)	13 (4·5%)	0 (0·0%)	-
Yes: still an in-patient at 30-days	90 (21·3%)	39 (30·0%)	51 (17·5%)	0 (0·0%)	-
No: data is based on in-patient observations only	27 (6·4%)	13 (10·0%)	13 (4·5%)	1 (100·0%)	-
No: follow-up was done, but prior to 30-days	28 (6·7%)	6 (4·6%)	22 (7·6%)	0 (0·0%)	-
If the patient had a complication, when was it diagnosed?					
During the primary admission	186 (33·4%)	45 (31·9%)	141 (34·4%)	0 (0·0%)	**<0·001**
As an emergency re-attender	18 (3·2%)	8 (5·7%)	10 (2·4%)	0 (0·0%)	-
At routine follow-up as an out-patient	15 (2·7%)	2 (1·4%)	13 (3·2%)	0 (0·0%)	-
Not applicable, no complications	338 (60·7%)	86 (61·0%)	246 (60·0%)	6 (100·0%)	-

*Patients born in hospital = 0. HIC: High-income countries. IQR: Interquartile range. LIC: Low-income countries. MIC: Middle-income countries. NEC: Necrotising enterocolitis. OA: Oesophageal atresia. TOF: Trachea-oesophageal fistula.

Supplementary Table 2: Characteristics, perioperative care, surgical interventions, and outcomes for patients with congenital diaphragmatic hernia (CDH)

Variable	Total (n=448)	HIC (n=148)	LMIC* (n=300)	P value
**Patient Characteristics:**				
Median gestational age at birth (IQR), weeks	38 (2)	38 (2)	38 (2)	0·534
Median age at presentation (IQR), hours	7 (96)	0 (24)	20 (168)	**<0·001**
Sex:				
Male	262 (58·5%)	83 (56·1%)	179 (59·7%)	0·470
Female	186 (41·5%)	65 (43·9%)	121 (40·3%)	
Median weight at presentation (IQR), kg	3·1 (1·0)	3·2 (1·0)	3·0 (0·9)	0·493
Does the patient have another anomaly in addition to the study condition?				
Yes: Cardiovascular	179 (40·0%)	55 (37·2%)	124 (41·3%)	0·397
Yes: Respiratory	70 (15·6%)	23 (15·5%)	47 (15·7%)	0·972
Yes: Gastrointestinal	24 (5·4%)	12 (8·1%)	12 (4·0%)	0·069
Yes: Neurological	17 (3·8%)	12 (8·1%)	5 (1·7%)	**0·001**
Yes: Genito-urinary	16 (3·6%)	10 (6·8%)	6 (2·0%)	**0·011**
Yes: Musculoskeletal	16 (3·6%)	9 (6·1%)	7 (2·3%)	**0·044**
Yes: Down syndrome	3 (0·7%)	0 (0·0%)	3 (1·0%)	0·222
Yes: Beckwith Wiedemann syndrome	0 (0·0%)	0 (0·0%)	0 (0·0%)	-
Yes: Cystic fibrosis	0 (0·0%)	0 (0·0%)	0 (0·0%)	-
Yes: Chromosomal	9 (2·0%)	4 (2·7%)	5 (1·7%)	0·462
Yes: Other	22 (4·9%)	9 (6·1%)	13 (4·3%)	0·421
No	209 (46·7%)	69 (46·6%)	140 (46·7%)	0·993
Median distance from patient's home to hospital (IQR), km†	13 (89)	6 (56)	15 (108)	**0·003**
Type of delivery:				
Vaginal (spontaneous)	190 (42·4%)	59 (39·9%)	131 (43·7%)	**<0·001**
Vaginal (induced)	33 (7·4%)	21 (14·2%)	12 (4·0%)	-
Caesarean section (elective)	123 (27·5%)	30 (20·3%)	93 (31·0%)	-
Caesarean section (urgent/non-elective)	92 (20·5%)	29 (19·6%)	63 (21·0%)	-
Unknown	9 (2·0%)	8 (5·4%)	1 (0·3%)	-
Missing	1 (0·2%)	1 (0·7%)	0 (0·0%)	-
Was the patient septic on arrival to your hospital?				
Yes	74 (16·5%)	5 (3·4%)	69 (23·0%)	**<0·001**
No	372 (83·0%)	142 (95·9%)	230 (76·7%)	-
Missing	2 (0·4%)	1 (0·7%)	1 (0·3%)	-
Was the patient hypovolaemic on arrival to your hospital?				
Yes	63 (14·1%)	15 (10·1%)	48 (16·0%)	0·092
No	384 (85·7%)	132 (89·2%)	252 (84·0%)	-
Missing	1 (0·2%)	1 (0·7%)	0 (0·0%)	-
Was the patient hypothermic on arrival to your hospital?				
Yes	49 (10·9%)	3 (2·0%)	46 (15·3%)	**<0·001**
No	398 (88·8%)	144 (97·3%)	254 (84·7%)	-
Missing	1 (0·2%)	1 (0·7%)	0 (0·0%)	-
American Society of Anaesthesiologists (ASA) Score at the time of primary intervention:				
1. Healthy person	38 (8·5%)	2 (1·4%)	36 (12·0%)	**<0·001**
2. Mild systemic disease	76 (17·0%)	17 (11·5%)	59 (19·7%)	-
3. Severe systemic disease	148 (33·0%)	63 (42·6%)	85 (28·3%)	-
4. Severe systemic disease that is a constant threat to life	96 (21·4%)	47 (31·8%)	49 (16·3%)	-
5. A moribund patient who is not expected to survive without the operation	23 (5·1%)	7 (4·7%)	16 (5·3%)	-
Not applicable - no intervention	66 (14·7%)	11 (7·4%)	55 (18·3%)	-
Missing	1 (0·2%)	1 (0·7%)	0 (0·0%)	-
What study condition does the patient have?				
Oesophageal atresia	1(0·2%)	1(0·7%)	0(0·0%)	0·154
Congenital diaphragmatic hernia	448 (100%)	148 (100%)	300 (100%)	-
Intestinal atresia	0 (0·0%)	0(0·0%)	0(0·0%)	-
Gastroschisis	0 (0·0%)	0(0·0%)	0(0·0%)	-
Exomphalos/Omphalocele	1(0·2%)	1(0·7%)	0(0·0%)	0·154
Anorectal malformation	1(0·2%)	1(0·7%)	0(0·0%)	0·154
Hirschsprung's Disease	1(0·2%)	0(0·0%)	1(0·3%)	0·482
Type of CDH				
Left posteriolateral (Bochdalek)	316 (70·5%)	100 (67·6%)	216 (72·0%)	0·190
Right posteriolateral (Bochdalek)	69 (15·4%)	30 (20·3%)	39 (13·0%)	-
Bilateral posteriolateral (Bochdalek)	7 (1·6%)	1 (0·7%)	6 (2·0%)	-
Central	21 (4·7%)	5 (3·4%)	16 (5·3%)	-
Anterior (Morgagni)	21 (4·7%)	9 (6·1%)	12 (4·0%)	-
Other	2 (0·4%)	0 (0·0%)	2 (0·7%)	-
Hiatal hernia	4 (0·9%)	2 (1·4%)	2 (0·7%)	-
Eventration	2 (0·4%)	1 (0·7%)	1 (0·3%)	-

Unknown	6 (1·3%)	0 (0·0%)	6 (2·0%)	-
Type of Bochdalek CDH (CDH Study Group Classification)				
A	41 (10·7%)	15 (11·6%)	26 (10·3%)	**0·012**
B	168 (44·0%)	57 (44·2%)	111 (43·9%)	-
C	87 (22·8%)	36 (27·9%)	51 (20·2%)	-
D	29 (7·6%)	12 (9·3%)	17 (6·7%)	-
Other (specify)	1 (0·3%)	1 (0·8%)	0 (0·0%)	-
Unknown	56 (14·7%)	8 (6·2%)	48 (19·0%)	-
If bilateral, what was the type of Bochdalek hernia on the left (CDH Study Group)				
A	0 (0·0%)	0 (0·0%)	0 (0·0%)	0·230
B	0 (0·0%)	0 (0·0%)	0 (0·0%)	-
C	2 (28·6%)	1 (100·0%)	1 (16·7%)	-
D	1 (14·3%)	0 (0·0%)	1 (16·7%)	-
Unknown	4 (57·1%)	0 (0·0%)	4 (66·7%)	-
If bilateral, what was the type of Bochdalek hernia on the right (CDH Study Group)				
A	1 (14·3%)	0 (0·0%)	1 (16·7%)	0·072
B	1 (14·3%)	0 (0·0%)	1 (16·7%)	-
C	1 (14·3%)	1 (100·0%)	0 (0·0%)	-
D	0 (0·0%)	0 (0·0%)	0 (0·0%)	-
Unknown	4 (57·1%)	0 (0·0%)	4 (66·7%)	-
Liver position?				
Chest	124 (27·7%)	57 (38·5%)	67 (22·3%)	**<0·001**
Abdomen	284 (63·4%)	83 (56·1%)	201 (67·0%)	-
Unknown	40 (8·9%)	8 (5·4%)	32 (10·7%)	-
Did the patient have pulmonary hypertension (at any stage)?				
Yes: diagnosed clinically	57 (12·7%)	13 (8·8%)	44 (14·7%)	**<0·001**
Yes: diagnosis confirmed on echocardiography	202 (45·1%)	70 (47·3%)	132 (44·0%)	-
No	152 (33·9%)	61 (41·2%)	91 (30·3%)	-
Unknown	36 (8·0%)	3 (2·0%)	33 (11·0%)	-
Missing	1 (0·2%)	1 (0·7%)	0 (0·0%)	-
**Care prior to presentation at the paediatric surgery centre:**				
Antenatal ultrasound undertaken?				
Yes: study condition diagnosed	183 (40·8%)	88 (59·5%)	95 (31·7%)	**<0·001**
Yes: problem identified but study condition not diagnosed	28 (6·3%)	8 (5·4%)	20 (6·7%)	-
Yes: no problem identified	191 (42·6%)	42 (28·4%)	149 (49·7%)	-
No	44 (9·8%)	8 (5·4%)	36 (12·0%)	-
Missing	2 (0·4%)	2 (1·4%)	0 (0·0%)	-
Median gestational age of study condition diagnosis if diagnosis was antenatal (IQR), weeks	26 (13)	24 (12)	27 (11)	0·129
Mode of transport to hospital:				
Ambulance	169 (37·7%)	43 (29·1%)	126 (42·0%)	**<0·001**
Other transport provided by the health service	17 (3·8%)	8 (5·4%)	9 (3·0%)	-
Patient's own transport	96 (21·4%)	14 (9·5%)	82 (27·3%)	-
Born within the hospital	165 (36·8%)	82 (55·4%)	83 (27·7%)	-
Missing	1 (0·2%)	1 (0·7%)	0 (0·0%)	-
If outborn, where did the patient present from?				
Home	58 (20·7%)	12 (18·8%)	46 (21·3%)	0·580
Community Clinic/General Practice	39 (13·9%)	7 (10·9%)	32 (14·8%)	-
District Hospital	180 (64·3%)	45 (70·3%)	135 (62·5%)	-
Unknown	3 (1·1%)	0 (0·0%)	3 (1·4%)	-
**Perioperative care at the paediatric surgery centre:**				
If septic, were appropriate antibiotics administered?				
Yes within 1 hour of arrival	63 (85·1%)	5 (100·0%)	58 (84·1%)	0·330
Yes within the first day of arrival	11 (14·9%)	0 (0·0%)	11 (15·9%)	-
No	0 (0·0%)	0 (0·0%)	0 (0·0%)	-
If hypovolaemic, was an intravenous fluid bolus given?				
Yes within 1 hour of arrival	48 (76·2%)	7 (50·0%)	41 (85·4%)	**0·009**
Yes within the first day of arrival	13 (20·6%)	6 (42·9%)	7 (14·6%)	-
No	1 (1·6%)	1 (7·1%)	0 (0·0%)	-
Missing	1 (1·6%)	1	0 (0·0%)	-
If hypovolaemic, how much intravenous fluid was given?				
10 - 20mls/kg	43 (70·5%)	7 (53·8%)	36 (75·0%)	0·110
Above 20mls/kg	17 (27·9%)	6 (46·2%)	11 (22·9%)	-
Missing	1 (1·6%)	0 (0·0%)	1 (2·1%)	-
If hypothermic, was the patient warmed on arrival to your hospital to within a normal temperature range?				
Yes	45 (91·8%)	2 (66·7%)	43 (93·5%)	0·100
No	4 (8·2%)	1 (33·3%)	3 (6·5%)	
Did the patient receive central venous access?				
Yes: umbilical catheter	155 (34·6%)	74 (50·0%)	81 (27·0%)	**<0·001**
Yes: peripherally inserted central catheter (PICC)	139 (31·0%)	81 (54·7%)	58 (19·3%)	**<0·001**
Yes: percutaneously inserted central line with ultrasound guidance	77 (17·2%)	42 (28·4%)	35 (11·7%)	**<0·001**
Yes: surgically placed central line (open insertion)	29 (6·5%)	4 (2·7%)	25 (8·3%)	**0·023**

No	136 (30·4%)	12 (8·1%)	124 (41·3%)	**<0·001**
Median total duration of antibiotics following primary intervention (IQR), days	5 (9)	3 (5)	6 (10)	<0·001
Did the patient receive a blood transfusion?				
Yes: not cross-matched	10 (2·2%)	2 (1·4%)	8 (2·7%)	0·600
Yes: cross-matched.	175 (39·1%)	54 (36·5%)	121 (40·3%)	-
No: not required.	253 (56·5%)	87 (58·8%)	166 (55·3%)	-
No: it was required but not available.	9 (2·0%)	4 (2·7%)	5 (1·7%)	-
Missing	1 (0·2%)	1 (0·7%)	0 (0·0%)	-
Did the patient require ventilation?				
Yes and it was given	387 (86·4%)	138 (93·2%)	249 (83·0%)	**0·010**
Yes, but it was not available	3 (0·7%)	0 (0·0%)	3 (1·0%)	-
No	58 (12·9%)	10 (6·8%)	48 (16·0%)	-
Median time patient remained on ventilation if given (IQR), days	6 (11)	8 (11)	4 (8)	**<0·001**
Median time to first enteral feed (post-primary intervention) (IQR), days	4 (4)	4 (4)	4 (4)	0·923
Median time to full enteral feeds (post-primary intervention) (IQR), days	9 (11)	11 (14)	8 (10)	**<0·001**
Did the patient require parenteral nutrition?				
Yes and it was given	286 (63·8%)	127 (85·8%)	159 (53·0%)	**<0·001**
Yes and it was sometimes available, but less than required	13 (2·9%)	0 (0·0%)	13 (4·3%)	-
Yes, but it was not available	3 (0·7%)	0 (0·0%)	3 (1·0%)	-
No	146 (32·6%)	21 (14·2%)	125 (41·7%)	-
Median time patient received parenteral nutrition if received (IQR), days	10 (11)	13 (13)	8 (8)	**<0·001**
**Surgical intervention:**				
Primary intervention:				
Primary repair (non-absorbable sutures)	254 (56·7%)	73 (49·3%)	181 (60·3%)	**<0·001**
Palliation	68 (15·2%)	12 (8·1%)	56 (18·7%)	-
Patch repair	66 (14·7%)	43 (29·1%)	23 (7·7%)	-
Primary repair (absorbable sutures)	43 (9·6%)	15 (10·1%)	28 (9·3%)	-
Discharged with planned elective repair	8 (1·8%)	4 (2·7%)	4 (1·3%)	-
Other	3 (0·7%)	0 (0·0%)	3 (1·0%)	-
Missing	6 (1·3%)	1 (0·7%)	5 (1·7%)	-
If patch repair, material used:				
Permacol	2 (3·0%)	0 (0·0%)	2 (8·7%)	**<0·001**
PTFE	29 (43·9%)	23 (53·5%)	6 (26·1%)	-
Mesh plug	10 (15·2%)	3 (7·0%)	7 (30·4%)	-
Muscle flap	1 (1·5%)	1 (2·3%)	0 (0·0%)	-
Gortex	14 (21·2%)	14 (32·6%)	0 (0·0%)	-
Prolene	4 (6·1%)	0 (0·0%)	4 (17·4%)	-
Other	5 (7·6%)	1 (2·3%)	4 (17·4%)	-
Unknown	1 (1·5%)	1 (2·3%)	0 (0·0%)	-
Other procedures undertaken at the same time:				
Chest drain insertion	104 (23·2%)	24 (16·2%)	80 (26·7%)	**0·014**
Abdominal wall patch	16 (3·6%)	9 (6·1%)	7 (2·3%)	**0·044**
Fundoplication	14 (3·1%)	1 (0·7%)	13 (4·3%)	**0·036**
Correction of malrotation	26 (5·8%)	13 (8·8%)	13 (4·3%)	0·058
Appendicectomy	29 (6·5%)	13 (8·8%)	16 (5·3%)	0·163
Abdominal silo application (difficult closure)	6 (1·3%)	6 (4·1%)	0 (0·0%)	**0·000**
Gastrostomy insertion	1 (0·2%)	1 (0·7%)	0 (0·0%)	0·154
Central line insertion	2 (0·4%)	0 (0·0%)	2 (0·7%)	0·319
Resection of Meckle's Diverticulum	2 (0·4%)	2 (1·4%)	0 (0·0%)	**0·044**
Other (specify)	11 (2·5%)	3 (2·0%)	8 (2·7%)	0·681
None	178 (39·7%)	66 (44·6%)	112 (37·3%)	0·140
Surgical approach				
Laparotomy	266 (73·3%)	92 (70·2%)	174 (75·0%)	0·230
Laparoscopy	18 (5·0%)	4 (3·1%)	14 (6·0%)	
Thoracotomy	23 (6·3%)	10 (7·6%)	13 (5·6%)	
Thoracoscopy	52 (14·3%)	23 (17·6%)	29 (12·5%)	
Other (please specify)	1 (0·3%)	1 (0·8%)	0 (0·0%)	
Missing	3 (0·8%)	1 (0·8%)	2 (0·9%)	
Conversion to open				
Yes	9 (12·9%)	3 (11·1%)	6 (14·0%)	0·730
No	61 (87·1%)	24 (88·9%)	37 (86·0%)	
Median time from arrival at your hospital to primary intervention (IQR), hours	54 (96)	65 (72)	48 (96)	0·912
What type of anaesthesia was used for the primary intervention?				
General anaesthesia with endotracheal tube	364 (81·3%)	131 (88·5%)	233 (77·7%)	**0·005**
General anaesthesia with laryngeal airway	2 (0·4%)	0 (0·0%)	2 (0·7%)	-
Ketamine anaesthesia	1 (0·2%)	1 (0·7%)	0 (0·0%)	-
Spinal/caudal anaesthesia	0 (0·0%)	0 (0·0%)	0 (0·0%)	-
Local anaesthesia only	1 (0·2%)	1 (0·7%)	0 (0·0%)	-
No anaesthesia, just analgesia	1 (0·2%)	1 (0·7%)	0 (0·0%)	-
No anaesthesia, no analgesia	0 (0·0%)	0 (0·0%)	0 (0·0%)	-

Not applicable: no surgery or primary intervention undertaken.	79 (17·6%)	14 (9·5%)	65 (21·7%)	-
Who undertook the anaesthetic for the primary intervention?				
Anaesthetic doctor	367 (81·9%)	132 (89·2%)	235 (78·3%)	**0·004**
Anaesthetic nurse	0 (0·0%)	0 (0·0%)	0 (0·0%)	-
Medical officer	0 (0·0%)	0 (0·0%)	0 (0·0%)	-
Surgeon	0 (0·0%)	0 (0·0%)	0 (0·0%)	-
Other healthcare professional	1 (0·2%)	1 (0·7%)	0 (0·0%)	-
No anaesthetic undertaken	80 (17·9%)	15 (10·1%)	65 (21·7%)	-
Who undertook the primary intervention?				
Paediatric surgeon (or junior with paediatric surgeon assisting/in the room)	368 (82·1%)	133 (89·9%)	235 (78·3%)	**0·002**
General surgeon (or junior with general surgeon assisting/in the room)	1 (0·2%)	1 (0·7%)	0 (0·0%)	-
Junior doctor, medical officer or other (without a paediatric or general surgeon assisting/in the room)	0 (0·0%)	0 (0·0%)	0 (0·0%)	-
Trainee surgeon (without a paediatric or general surgeon assisting or in the room)	0 (0·0%)	0 (0·0%)	0 (0·0%)	-
Not applicable - no surgery or primary intervention undertaken.	79 (17·6%)	14 (9·5%)	65 (21·7%)	-
Was a Surgical Safety Checklist used at the time of primary intervention?				
Yes	304 (67·9%)	124 (83·8%)	180 (60·0%)	**<0·001**
No: but it was available	33 (7·4%)	6 (4·1%)	27 (9·0%)	-
No: it was not available	30 (6·7%)	1 (0·7%)	29 (9·7%)	-
Not applicable: a conservative primary intervention was undertaken	3 (0·7%)	1 (0·7%)	2 (0·7%)	-
Not applicable: no surgery or primary intervention undertaken	77 (17·2%)	15 (10·1%)	62 (20·7%)	-
Missing	1 (0·2%)	1 (0·7%)	0 (0·0%)	-
Was foetal tracheal occlusion (FETO) undertaken?				
Yes	6 (1·3%)	5 (3·4%)	1 (0·3%)	**0·008**
No	442 (98·7%)	143 (96·6%)	299 (99·7%)	-
If yes, at what gestational age was it inserted?	29 (2)	29 (2)	-	-
If yes, at was gestational age was it removed?	34 (2)	34 (2)	-	-
If the patient had pulmonary hypertension, what treatment was given?				
Nitric oxide	97 (37·5%)	64 (77·1%)	33 (18·8%)	**<0·001**
Prostacyclin	28 (10·8%)	18 (21·7%)	10 (5·7%)	**<0·001**
Alprostadil	13 (5·0%)	6 (7·2%)	7 (4·0%)	0·260
Milrinone	66 (25·5%)	23 (27·7%)	43 (24·4%)	0·570
Sildenafil	64 (24·7%)	17 (20·5%)	47 (26·7%)	0·280
Furosemide	2 (0·8%)	1 (1·2%)	1 (0·6%)	0·580
Other inotropes (dopamine, dobutamine, adrenaline, noradrenaline and others)	16 (6·2%)	5 (6·0%)	11 (6·3%)	0·940
None: not required	57 (22·0%)	12 (14·5%)	45 (25·6%)	**0·044**
None: required but not available	16 (6·2%)	0 (0·0%)	16 (9·1%)	**0·005**
Did the patient receive extracorporeal membrane oxygenation (ECMO)?				
Yes	28 (6·3%)	22 (14·9%)	6 (2·0%)	**<0·001**
No	420 (93·8%)	126 (85·1%)	294 (98·0%)	-
If yes, for how long (IQR), days	7 (7)	8 (7)	6 (2)	0·879
**Outcomes:**				
Did the patient survive to discharge (or 30-days if still an in-patient 30-days following primary intervention)?				
Yes	312 (69·6%)	127 (85·8%)	185 (61·7%)	**<0·001**
No	136 (30·4%)	21 (14·2%)	115 (38·3%)	-
If the patient was discharged prior, were they still alive at 30-days following primary intervention?				
Yes	280 (90·6%)	111 (89·5%)	169 (91·4%)	0·270
No	1 (0·3%)	0 (0·0%)	1 (0·5%)	-
Not followed-up after discharge	11 (3·6%)	3 (2·4%)	8 (4·3%)	-
Followed-up, but not until 30-days post primary intervention	17 (5·5%)	10 (8·1%)	7 (3·8%)	-
Cause of mortality:				
Respiratory failure	83 (60·6%)	10 (47·6%)	73 (62·9%)	**<0·001**
Cardiac failure	27 (19·7%)	4 (19·0%)	23 (19·8%)	-
Sepsis	16 (11·7%)	0 (0·0%)	16 (13·8%)	-
Haemorrhage	6 (4·4%)	3 (14·3%)	3 (2·6%)	-
Other	3 (2·2%)	2 (9·5%)	1 (0·9%)	-
Recurrent tracheo-oesophageal fistula	1 (0·7%)	1 (4·8%)	0 (0·0%)	-
Syndrome incompatible with life	1 (0·7%)	1 (4·8%)	0 (0·0%)	-
Median duration of hospital stay, days	13 (17)	21(19)	10 (14)	**<0·001**
Did the patient have a surgical site infection?				
Yes	25 (5·6%)	12 (8·1%)	13 (4·3%)	**0·002**
No	346 (77·2%)	123 (83·1%)	223 (74·3%)	-
Not applicable, no surgical wound	77 (17·2%)	13 (8·8%)	64 (21·3%)	-
Did the patient have a full thickness wound dehiscence?				
Yes	2 (0·4%)	1 (0·7%)	1 (0·3%)	**0·005**
No	366 (81·7%)	133 (89·9%)	233 (77·7%)	-
Not applicable, no surgical wound	80 (17·9%)	14 (9·5%)	66 (22·0%)	-
Did the patient require a further unplanned intervention?				
Yes – percutaneous	11 (2·5%)	6 (4·1%)	5 (1·7%)	**<0·001**
Yes – surgical intervention	28 (6·3%)	16 (10·8%)	12 (4·0%)	-
No	335 (74·8%)	113 (76·4%)	222 (74·0%)	-
Not applicable – no primary intervention undertaken	74 (16·5%)	13 (8·8%)	61 (20·3%)	-
If central line access required, did the patient acquire central line sepsis?				

Yes, diagnosed clinically	9 (2·9%)	3 (2·2%)	6 (3·4%)	0·700
Yes, confirmed on microbiology	16 (5·1%)	6 (4·3%)	10 (5·6%)	-
No	290 (92·1%)	129 (93·5%)	161 (91·0%)	-
Condition specific complication within 30-days of primary surgery?				
Air leak	33 (7·4%)	10 (6·8%)	23 (7·7%)	0·729
Chylothorax	14 (3·1%)	7 (4·7%)	7 (2·3%)	0·170
Adhesional obstruction	6 (1·3%)	2 (1·4%)	4 (1·3%)	0·988
Pleural effusion	3 (0·7%)	1 (0·7%)	2 (0·7%)	0·991
Recurrence	2 (0·4%)	2 (1·4%)	0 (0·0%)	**0·044**
Haemothorax	2 (0·4%)	1(0·7%)	1 (0·3%)	0·609
Pneumonia	2 (0·4%)	0 (0·0%)	2 (0·7%)	0·319
Phrenic nerve palsy	1 (0·2%)	0 (0·0%)	1 (0·3%)	0·482
Other	18 (4·0%)	5 (3·4%)	13 (4·3%)	-0·628
None	280 (62·5%)	99 (66·9%)	181 (60·3%)	0·177
Was the patient followed up at 30-days post primary surgery or intervention to assess for complications?				
Yes: reviewed in person	176 (56·4%)	70 (55·1%)	106 (57·3%)	**<0·001**
Yes: via telephone consultation	35 (11·2%)	4 (3·1%)	31 (16·8%)	-
Yes: via other means	6 (1·9%)	0 (0·0%)	6 (3·2%)	-
Yes: still an in-patient at 30-days	56 (17·9%)	34 (26·8%)	22 (11·9%)	-
No: data is based on in-patient observations only	21 (6·7%)	15 (11·8%)	6 (3·2%)	-
No: follow-up was done, but prior to 30-days	18 (5·8%)	4 (3·1%)	14 (7·6%)	-
If the patient had a complication, when was it diagnosed?				
During the primary admission	127 (28·3%)	42 (28·4%)	85 (28·3%)	0·760
As an emergency re-attender	6 (1·3%)	1 (0·7%)	5 (1·7%)	-
At routine follow-up as an out-patient	5 (1·1%)	1 (0·7%)	4 (1·3%)	-
Not applicable, no complications	309 (69·0%)	104 (70·3%)	205 (68·3%)	-
Missing	1 (0·2%)	0 (0·0%)	1 (0·3%)	-

*Only 1 patient was from a LIC and hence patients from MIC and LICs were combined in this table. †patients born in hospital = 0. Percentages have been rounded to 1 decimal place and may not total 100·0%. CDH: Congenital diaphragmatic hernia. HIC: High-income countries. IQR: Interquartile range. LMIC: Low- and middle-income countries.

Supplementary Table 3: Characteristics, perioperative care, surgical interventions, and outcomes for patients with intestinal atresia

Variable	Total (n=681)	HIC (n=152)	MIC (n=509)	LIC (n=20)	P value
**Patient Characteristics:**					
Median gestational age at birth (IQR), weeks	37 (3)	37 (3)	37 (3)	36 (2)	0·262
Median age at presentation (IQR), hours	24 (72)	0 (25)	36 (94)	96 (92)	**<0·001**
Sex:					
Male	336 (49·3%)	73 (48·0%)	256 (50·3%)	7 (35·0%)	0·610
Female	343 (50·4%)	79 (52·0%)	251 (49·3%)	13 (65·0%)	-
Ambiguous	2 (0·3%)	0 (0·0%)	2 (0·4%)	0 (0·0%)	-
Median weight at presentation (IQR), kg	2·5 (1·0)	2·7 (1·1)	2·4 (1·0)	2·2 (0·7)	0·124
Does the patient have another anomaly in addition to the study condition?					
Yes: Cardiovascular	151 (22·2%)	49 (32·2%)	98 (19·3%)	4 (20·0%)	**0·003**
Yes: Respiratory	20 (2·9%)	7 (4·6%)	13 (2·6%)	0 (0·0%)	0·309
Yes: Gastrointestinal	81 (11·9%)	24 (15·8%)	56 (11·0%)	1 (5·0%)	0·174
Yes: Neurological	23 (3·4%)	7 (4·6%)	16 (3·1%)	0 (0·0%)	0·476
Yes: Genito-urinary	34 (5·0%)	10 (6·6%)	24 (4·7%)	0 (0·0%)	0·379
Yes: Musculoskeletal	18 (2·6%)	6 (3·9%)	12 (2·4%)	0 (0·0%)	0·425
Yes: Down syndrome	65 (9·5%)	17 (11·2%)	48 (9·4%)	0 (0·0%)	0·274
Yes: Beckwith Wiedemann syndrome	0 (0·0%)	0 (0·0%)	0 (0·0%)	0 (0·0%)	-
Yes: Cystic fibrosis	5 (0·7%)	2 (1·3%)	2 (0·4%)	1 (5·0%)	**0·039**
Yes: Chromosomal	14 (2·1%)	5 (3·3%)	9 (1·8%)	0 (0·0%)	0·411
Yes: Other	36 (5·3%)	7 (4·6%)	29 (5·7%)	0 (0·0%)	0·489
No	402 (59·0%)	80 (52·6%)	307 (60·3%)	15 (75·0%)	**0·081**
Median distance from patient's home to hospital (IQR), km*	19 (96)	6 (46)	25 (108)	28 (109)	**<0·001**
Type of delivery:					
Vaginal (spontaneous)	333 (48·9%)	68 (44·7%)	248 (48·7%)	17 (85·0%)	**<0·001**
Vaginal (induced)	20 (2·9%)	12 (7·9%)	8 (1·6%)	0 (0·0%)	-
Caesarean section (elective)	145 (21·3%)	24 (15·8%)	120 (23·6%)	1 (5·0%)	-
Caesarean section (urgent/non-elective)	181 (26·6%)	48 (31·6%)	131 (25·7%)	2 (10·0%)	-
Unknown	2 (0·3%)	0 (0·0%)	2 (0·4%)	0 (0·0%)	-
Was the patient septic on arrival to your hospital?					
Yes	141 (20·7%)	3 (2·0%)	127 (25·0%)	11 (55·0%)	**<0·001**
No	540 (79·3%)	149 (98·0%)	382 (75·0%)	9 (45·0%)	-
Was the patient hypovolaemic on arrival to your hospital?					
Yes	142 (20·9%)	12 (7·9%)	124 (24·4%)	6 (30·0%)	**<0·001**
No	539 (79·1%)	140 (92·1%)	385 (75·6%)	14 (70·0%)	-
Was the patient hypothermic on arrival to your hospital?					
Yes	74 (10·9%)	9 (5·9%)	62 (12·2%)	3 (15·0%)	**0·077**
No	606 (89·0%)	143 (94·1%)	446 (87·6%)	17 (85·0%)	-
Missing	1 (0·1%)	0 (0·0%)	1 (0·2%)	0 (0·0%)	-
American Society of Anaesthesiologists (ASA) Score at the time of primary intervention:					
1. Healthy person	99 (14·5%)	19 (12·5%)	76 (14·9%)	4 (20·0%)	**0·009**
2. Mild systemic disease	220 (32·3%)	47 (30·9%)	163 (32·0%)	10 (50·0%)	-
3. Severe systemic disease	239 (35·1%)	59 (38·8%)	177 (34·8%)	3 (15·0%)	-
4. Severe systemic disease that is a constant threat to life	57 (8·4%)	20 (13·2%)	36 (7·1%)	1 (5·0%)	-
5. A moribund patient who is not expected to survive without the operation	40 (5·9%)	2 (1·3%)	38 (7·5%)	0 (0·0%)	-
Not applicable - no intervention	24 (3·5%)	3 (2·0%)	19 (3·7%)	2 (10·0%)	-
Missing	2 (0·3%)	2 (1·3%)	0 (0·0%)	0 (0·0%)	-
What study condition does the patient have?					
Oesophageal atresia	18 (2·6%)	7 (4·6%)	11 (2·2%)	0 (0·0%)	0·194
Congenital diaphragmatic hernia	0 (0·0%)	0 (0·0%)	0 (0·0%)	0 (0·0%)	-
Intestinal atresia	681 (100·0%)	152 (100·0%)	509 (100·0%)	20 (100·0%)	-
Gastroschisis	17 (2·5%)	8 (5·3%)	9 (1·8%)	0 (0·0%)	**0·041**
Exomphalos/Omphalocele	8 (1·2%)	3 (2·0%)	5 (1·0%)	0 (0·0%)	0·539
Anorectal malformation	12 (1·8%)	3 (2·0%)	9 (1·8%)	0 (0·0%)	0·819
Hirschsprung's Disease	3 (0·4%)	2 (1·3%)	1 (0·2%)	0 (0·0%)	0·180
Type of intestinal atresia?					
Duodenal (DA)	279 (41·0%)	83 (54·6%)	189 (37·1%)	7 (35·0%)	**<0·001**
Jejuno-ileal (JIA)	369 (54·2%)	57 (37·5%)	300 (58·9%)	12 (60·0%)	-
Colonic (CA)	31 (4·6%)	11 (7·2%)	19 (3·7%)	1 (5·0%)	-
Missing	2 (0·3%)	1 (0·7%)	1 (0·2%)	0 (0·0%)	-
Classification of duodenal or colonic atresia?					
1	162 (52·4%)	38 (40·4%)	119 (57·5%)	5 (62·5%)	**0·070**
2	73 (23·6%)	26 (27·7%)	44 (21·3%)	3 (37·5%)	-
3	69 (22·3%)	29 (30·9%)	40 (19·3%)	0 (0·0%)	-
4	5 (1·6%)	1 (1·1%)	4 (1·9%)	0 (0·0%)	-
Classification of jejuno-ileal atresia					

1	77 (20·8%)	9 (15·8%)	64 (21·3%)	4 (33·3%)	**0·014**
2	72 (19·5%)	20 (35·1%)	50 (16·6%)	2 (16·7%)	-
3a	115 (31·1%)	16 (28·1%)	97 (32·2%)	2 (16·7%)	-
3b	45 (12·2%)	5 (8·8%)	36 (12·0%)	4 (33·3%)	-
4	61 (16·5%)	7 (12·3%)	54 (17·9%)	0 (0·0%)	-
**Care prior to presentation at the paediatric surgery centre:**					
Antenatal ultrasound undertaken?					
Yes: study condition diagnosed	194 (28·5%)	77 (50·7%)	117 (23·0%)	0 (0·0%)	**<0·001**
Yes: problem identified but study condition not diagnosed	136 (20·0%)	31 (20·4%)	100 (19·6%)	5 (25·0%)	-
Yes: no problem identified	264 (38·8%)	39 (25·7%)	217 (42·6%)	8 (40·0%)	-
No	85 (12·5%)	4 (2·6%)	74 (14·5%)	7 (35·0%)	-
Missing	2 (0·3%)	1 (0·7%)	1 (0·2%)	0 (0·0%)	-
Median gestational age of study condition diagnosis if diagnosis was antenatal (IQR), weeks	30 (10)	30 (22)	30 (8)	-	0·914
Mode of transport to hospital:					
Ambulance	264 (38·8%)	58 (38·2%)	201 (39·5%)	5 (25·0%)	**<0·001**
Other transport provided by the health service	46 (6·8%)	10 (6·6%)	30 (5·9%)	6 (30·0%)	-
Patient's own transport	155 (22·8%)	3 (2·0%)	143 (28·1%)	9 (45·0%)	-
Born within the hospital	214 (31·4%)	81 (53·3%)	133 (26·1%)	0 (0·0%)	-
Missing	2 (0·3%)	0 (0·0%)	2 (0·4%)	0 (0·0%)	-
If outborn, where did the patient present from?					
Home	56 (12·0%)	1 (1·4%)	53 (14·2%)	2 (10·0%)	**<0·001**
Community Clinic/General Practice	74 (15·9%)	4 (5·6%)	66 (17·6%)	4 (20·0%)	-
District Hospital	328 (70·5%)	63 (88·7%)	253 (67·6%)	12 (60·0%)	-
From another country	1 (0·2%)	1 (1·4%)	0 (0·0%)	0 (0·0%)	-
From a different speciality within the study centre	3 (0·6%)	2 (2·8%)	0 (0·0%)	1 (5·0%)	-
Unknown	3 (0·6%)	0 (0·0%)	2 (0·5%)	1 (5·0%)	-
**Perioperative care at the paediatric surgery centre:**					
If septic, were appropriate antibiotics administered?					
Yes within 1 hour of arrival	106 (75·2%)	3 (100·0%)	96 (75·6%)	7 (63·6%)	0·190
Yes: within the first day of arrival	33 (23·4%)	0 (0·0%)	30 (23·6%)	3 (27·3%)	-
No	2 (1·4%)	0 (0·0%)	1 (0·8%)	1 (9·1%)	-
If hypovolaemic, was an intravenous fluid bolus given?					
Yes within 1 hour of arrival	110 (77·5%)	7 (58·3%)	99 (79·8%)	4 (66·7%)	0·400
Yes: within the first day of arrival	26 (18·3%)	4 (33·3%)	20 (16·1%)	2 (33·3%)	-
No	6 (4·2%)	1 (8·3%)	5 (4·0%)	0 (0·0%)	-
If hypovolaemic, how much intravenous fluid was given?					
10 - 20mls/kg	98 (72·1%)	7 (63·6%)	86 (72·3%)	5 (83·3%)	0·680
Above 20mls/kg	38 (27·9%)	4 (36·4%)	33 (27·7%)	1 (16·7%)	-
If hypothermic, was the patient warmed on arrival to your hospital to within a normal temperature range?					
Yes	69 (93·2%)	7 (77·8%)	59 (95·2%)	3 (100·0%)	0·140
No	5 (6·8%)	2 (22·2%)	3 (4·8%)	0 (0·0%)	-
Did the patient receive central venous access?					
Yes: umbilical catheter	69 (10·1%)	20 (13·2%)	49 (9·6%)	0 (0·0%)	0·140
Yes: peripherally inserted central catheter (PICC)	268 (39·4%)	99 (65·1%)	168 (33·0%)	1 (5·0%)	**<0·001**
Yes: percutaneously inserted central line with ultrasound guidance	106 (15·6%)	40 (26·3%)	66 (13·0%)	0 (0·0%)	**<0·001**
Yes: surgically placed central line (open insertion)	60 (8·8%)	6 (3·9%)	54 (10·6%)	0 (0·0%)	**0·015**
No	234 (34·4%)	13 (8·6%)	202 (39·7%)	19 (95·0%)	**<0·001**
Duration of antibiotics following primary intervention (days), median (IQR)	8 (10)	4 (5)	10 (9)	5 (7)	**<0·001**
Did the patient receive a blood transfusion?					
Yes: not cross-matched	20 (2·9%)	4 (2·6%)	16 (3·1%)	0 (0·0%)	**<0·001**
Yes: cross-matched.	334 (49·0%)	41 (27·0%)	283 (55·6%)	10 (50·0%)	-
No: not required.	322 (47·3%)	106 (69·7%)	206 (40·5%)	10 (50·0%)	-
No: it was required but not available.	5 (0·7%)	1 (0·7%)	4 (0·8%)	0 (0·0%)	**<0·001**
Did the patient require ventilation?					
Yes: and it was given	370 (54·3%)	117 (77·0%)	252 (49·5%)	1 (5·0%)	**<0·001**
Yes, but it was not available	21 (3·1%)	1 (0·7%)	17 (3·3%)	3 (15·0%)	-
No	290 (42·6%)	34 (22·4%)	240 (47·2%)	16 (80·0%)	-
Median time patient remained on ventilation if given (IQR), days	3 (4)	3 (4)	3 (4)	3 (0)	0·952
Median time to first enteral feed (post-primary intervention) (IQR), days	7 (5)	7 (5)	7 (6)	3 (4)	**<0·001**
Median time to full enteral feeds (post-primary intervention) (IQR), days	14 (13)	16 (17)	13 (12)	5 (3)	**<0·001**
Did the patient require parenteral nutrition?					
Yes: and it was given	490 (72·0%)	141 (92·8%)	347 (68·2%)	2 (10·0%)	**<0·001**
Yes: and it was sometimes available, but less than required	37 (5·4%)	0 (0·0%)	37 (7·3%)	0 (0·0%)	-
Yes: but it was not available	48 (7·0%)	0 (0·0%)	37 (7·3%)	11 (55·0%)	-
No	106 (15·6%)	11 (7·2%)	88 (17·3%)	7 (35·0%)	-
Median time patient received parenteral nutrition if received (IQR), days	14 (12)	15 (12)	14 (12)	20 (20)	0·055
**Surgical intervention:**					
Time from arrival to primary intervention in hours, median (IQR)	25 (52)	22 (28)	28 (57)	48 (84)	**<0·001**
Primary intervention for patients with duodenal atresia:					
Duodenoduodenostomy	200 (71·9%)	62 (74·7%)	134 (71·3%)	4 (57·1%)	0·69

Duodenojejunostomy	39 (14·0%)	10 (12·0%)	28 (14·9%)	1 (14·3%)	-
Web excision only	20 (7·2%)	7 (8·4%)	12 (6·4%)	1 (14·3%)	-
Palliation	9 (3·2%)	1 (1·2%)	7 (3·7%)	1 (14·3%)	-
Other	10 (3·6%)	3 (3·6%)	7 (3·7%)	0 (0·0%)	-
Surgical approach for patients with duodenal atresia:					
Laparotomy	224 (87·8%)	59 (74·7%)	159 (93·5%)	6 (100·0%)	**0·002**
Laparoscopy	26 (10·2%)	18 (22·8%)	8 (4·7%)	0 (0·0%)	-
Endoscopy	1 (0·4%)	0 (0·0%)	1 (0·6%)	0 (0·0%)	-
Other	4 (1·6%)	2 (2·5%)	2 (1·2%)	0 (0·0%)	-
Type of anastomosis for patients with duodenal atresia:					
Kimura's diamond shape	162 (68·9%)	49 (68·1%)	112 (70·9%)	1 (20·0%)	0·180
Side-to-side	50 (21·3%)	15 (20·8%)	32 (20·3%)	3 (60·0%)	-
End-to-end	23 (9·8%)	8 (11·1%)	14 (8·9%)	1 (20·0%)	-
Primary intervention for JIA or CA?					
Primary anastomosis	264 (66·0%)	43 (63·2%)	214 (67·1%)	7 (53·8%)	**0·011**
Bowel resection	170 (42·5%)	24 (35·3%)	144 (45·1%)	2 (15·4%)	**0·037**
Divided stoma	50 (12·5%)	15 (22·1%)	34 (10·7%)	1 (7·7%)	0·713
Division of web only	16 (4·0%)	2 (2·9%)	13 (4·1%)	1 (7·7%)	0·250
Santulli stoma	15 (3·8%)	0 (0·0%)	15 (4·7%)	0 (0·0%)	0·096
Loop stoma	14 (3·5%)	2 (2·9%)	11 (3·4%)	1 (7·7%)	0·317
Bishop-Koop stoma	10 (2·5%)	1 (1·5%)	8 (2·5%)	1 (7·7%)	0·175
Palliation	8 (2·0%)	3 (4·4%)	5 (1·6%)	0 (0·0%)	0·272
Other	27 (6·8%)	3 (4·4%)	23 (7·2%)	1 (7·7%)	0·170
Surgical approach for patients with JIA or CA?					
Laparotomy	327 (95·9%)	51 (96·2%)	267 (96·0%)	9 (90·0%)	0·43
Laparoscopy	8 (2·3%)	1 (1·9%)	7 (2·5%)	0 (0·0%)	-
Endoscopy	1 (0·3%)	0 (0·0%)	1 (0·4%)	0 (0·0%)	-
Other	5 (1·5%)	1 (1·9%)	3 (1·1%)	1 (10·0%)	-
Conversion to open procedure for all patients undergoing laparoscopy or endoscopy (DA, JIA or CA)?					
Yes	6 (16·7%)	1 (5·3%)	5 (29·4%)	-	**0·052**
No	30 (83·3%)	18 (94·7%)	12 (70·6%)	-	-
Was the distal bowel flushed to check for patency?					
Yes	442 (83·2%)	78 (62·9%)	351 (89·3%)	13 (92·9%)	**<0·001**
No	89 (16·8%)	46 (37·1%)	42 (10·7%)	1 (7·1%)	-
Median length of bowel excised in patients undergoing a bowel resection, in cm (IQR)	15 (15)	11 (14)	15 (15)	50 (20)	**0·026**
What type of anaesthesia was used for the primary intervention?					
General anaesthesia with endotracheal tube	655 (96·2%)	149 (98·0%)	489 (96·1%)	17 (85·0%)	0·160
General anaesthesia with laryngeal airway	4 (0·6%)	0 (0·0%)	3 (0·6%)	1 (5·0%)	-
Ketamine anaesthesia	0 (0·0%)	0 (0·0%)	0 (0·0%)	0 (0·0%)	-
Spinal/caudal anaesthesia	0 (0·0%)	0 (0·0%)	0 (0·0%)	0 (0·0%)	-
Local anaesthesia only	2 (0·3%)	0 (0·0%)	2 (0·4%)	0 (0·0%)	-
No anaesthesia, just analgesia	1 (0·1%)	0 (0·0%)	1 (0·2%)	0 (0·0%)	-
No anaesthesia, no analgesia	2 (0·3%)	0 (0·0%)	2 (0·4%)	0 (0·0%)	-
Not applicable: no surgery or primary intervention undertaken	17 (2·5%)	3 (2·0%)	12 (2·4%)	2 (10·0%)	-
Who undertook the anaesthetic for the primary intervention?					
Anaesthetic doctor	646 (94·9%)	144 (94·7%)	488 (95·9%)	14 (70·0%)	**<0·001**
Anaesthetic nurse	9 (1·3%)	1 (0·7%)	4 (0·8%)	4 (20·0%)	-
Medical officer	2 (0·3%)	2 (1·3%)	0 (0·0%)	0 (0·0%)	-
Surgeon	2 (0·3%)	0 (0·0%)	2 (0·4%)	0 (0·0%)	-
Other healthcare professional	2 (0·3%)	2 (1·3%)	0 (0·0%)	0 (0·0%)	-
No anaesthetic undertaken	20 (2·9%)	3 (2·0%)	15 (2·9%)	2 (10·0%)	-
Who undertook the primary intervention?					
Paediatric surgeon (or junior with paediatric surgeon assisting/in the room)	654 (96·0%)	149 (98·0%)	489 (96·1%)	16 (80·0%)	**0·007**
General surgeon (or junior with general surgeon assisting/in the room)	6 (0·9%)	1 (0·7%)	4 (0·8%)	1 (5·0%)	-
Junior doctor, medical officer or other (without a paediatric or general surgeon assisting/in the room)	2 (0·3%)	0 (0·0%)	2 (0·4%)	0 (0·0%)	-
Trainee surgeon (without a paediatric or general surgeon assisting or in the room)	3 (0·4%)	0 (0·0%)	2 (0·4%)	1 (5·0%)	-
Not applicable - no surgery or primary intervention undertaken.	16 (2·3%)	2 (1·3%)	12 (2·4%)	2 (10·0%)	-
Was a Surgical Safety Checklist used at the time of primary intervention?					
Yes	530 (77·8%)	144 (94·7%)	378 (74·3%)	8 (40·0%)	**<0·001**
No: but it was available	68 (10·0%)	4 (2·6%)	58 (11·4%)	6 (30·0%)	-
No: it was not available	66 (9·7%)	1 (0·7%)	61 (12·0%)	4 (20·0%)	-
Not applicable: a conservative primary intervention was undertaken	3 (0·4%)	1 (0·7%)	2 (0·4%)	0 (0·0%)	-
Not applicable: no surgery or primary intervention undertaken	14 (2·1%)	2 (1·3%)	10 (2·0%)	2 (10·0%)	-
**Outcomes:**					
Did the patient survive to discharge (or 30-days if still an in-patient 30-days following primary intervention)?					
Yes	555 (81·5%)	147 (96·7%)	400 (78·6%)	8 (40·0%)	**<0·001**
No	126 (18·5%)	5 (3·3%)	109 (21·4%)	12 (60·0%)	-
If the patient was discharged prior, were they still alive at 30-days following primary intervention?					

Yes	508 (92·2%)	141 (97·2%)	362 (91·0%)	5 (62·5%)	**<0·001**
No	2 (0·4%)	0 (0·0%)	2 (0·5%)	0 (0·0%)	-
Not followed-up after discharge	21 (3·8%)	2 (1·4%)	16 (4·0%)	3 (37·5%)	-
Followed-up, but not until 30-days post primary intervention	20 (3·6%)	2 (1·4%)	18 (4·5%)	0 (0·0%)	-
Cause of mortality:					
Sepsis	67 (52·3%)	0 (0·0%)	63 (56·8%)	4 (33·3%)	**0·001**
Respiratory failure	17 (13·3%)	1 (20·0%)	15 (13·5%)	1 (8·3%)	-
Cardiac failure	14 (10·9%)	4 (80·0%)	9 (8·1%)	1 (8·3%)	-
Anastomotic leak	8 (6·3%)	0 (0·0%)	6 (5·4%)	2 (16·7%)	-
Aspiration pneumonia	7 (5·5%)	0 (0·0%)	5 (4·5%)	2 (16·7%)	-
Malnutrition	4 (3·1%)	0 (0·0%)	4 (3·6%)	0 (0·0%)	-
Electrolyte disturbance	4 (3·1%)	0 (0·0%)	3 (2·7%)	1 (8·3%)	-
Haemorrhage	2 (1·6%)	0 (0·0%)	1 (0·9%)	1 (8·3%)	-
Other	5 (3·9%)	0 (0·0%)	5 (4·5%)	0 (0·0%)	-
Median duration of hospital stay in days (IQR)	19 (17)	24 (13)	18 (15)	11 (9)	**<0·001**
Did the patient have a surgical site infection?					
Yes	71 (10·4%)	16 (10·5%)	53 (10·4%)	2 (10·0%)	0·590
No	586 (86·0%)	132 (86·8%)	438 (86·1%)	16 (80·0%)	-
Not applicable, no surgical wound	24 (3·5%)	4 (2·6%)	18 (3·5%)	2 (10·0%)	-
Did the patient have a full thickness wound dehiscence?					
Yes	17 (2·5%)	2 (1·3%)	15 (2·9%)	0 (0·0%)	0·390
No	639 (93·8%)	145 (95·4%)	476 (93·5%)	18 (90·0%)	-
Not applicable, no surgical wound	25 (3·7%)	5 (3·3%)	18 (3·5%)	2 (10·0%)	-
Did the patient require a further unplanned intervention?					
Yes – percutaneous	5 (0·7%)	2 (1·3%)	3 (0·6%)	0 (0·0%)	0·340
Yes – surgical intervention	102 (15·0%)	19 (12·5%)	78 (15·3%)	5 (25·0%)	-
No	552 (81·1%)	127 (83·6%)	412 (80·9%)	13 (65·0%)	-
Not applicable – no primary intervention undertaken	22 (3·2%)	4 (2·6%)	16 (3·1%)	2 (10·0%)	-
If central line access was used, did the patient acquire central line sepsis?					
Yes, diagnosed clinically	24 (5·4%)	6 (4·3%)	18 (5·8%)	0 (0·0%)	0·610
Yes, confirmed on microbiology	30 (6·7%)	13 (9·4%)	17 (5·5%)	0 (0·0%)	-
No	394 (87·9%)	120 (86·3%)	273 (88·6%)	1 (100·0%)	-
Condition specific complications within 30-days of primary intervention:					
Anastomotic leak	57 (8·4%)	0 (0·0%)	52 (10·2%)	5 (25·0%)	**<0·001**
Short-gut	26 (3·8%)	4 (2·6%)	22 (4·3%)	0 (0·0%)	0·421
Adhesive bowel obstruction	23 (3·4%)	3 (2·0%)	20 (3·9%)	0 (0·0%)	0·351
Anastomotic stenosis	19 (2·8%)	4 (2·6%)	13 (2·6%)	2 (10·0%)	0·139
Stoma prolapse	8 (1·2%)	4 (2·6%)	4 (0·8%)	0 (0·0%)	0·159
Difficulty establishing/ tolerating enteral feeds/intestinal dysmotility	7 (1·0%)	1 (0·7%)	6 (1·2%)	0 (0·0%)	0·769
Parastomal skin breakdown	6 (0·9%)	0 (0·0%)	5 (1·0%)	1 (5·0%)	0·071
Stoma retraction	5 (0·7%)	1 (0·7%)	4 (0·8%)	0 (0·0%)	0·915
Pneumonia (aspiration pneumonia or pneumonia)	5 (0·7%)	0 (0·0%)	4 (0·8%)	1 (5·0%)	**0·047**
Missed additional atresia	4 (0·6%)	0 (0·0%)	4 (0·8%)	0 (0·0%)	0·507
Bowel perforation	4 (0·6%)	3 (2·0%)	1 (0·2%)	0 (0·0%)	**0·040**
Electrolyte disturbance	4 (0·6%)	0 (0·0%)	4 (0·8%)	0 (0·0%)	0·507
High output stoma	3 (0·4%)	3 (2·0%)	0 (0·0%)	0 (0·0%)	**0·005**
Other bowel pathology	3 (0·4%)	3 (2·0%)	0 (0·0%)	0 (0·0%)	**0·005**
N/A, No intervention	3 (0·4%)	0 (0·0%)	2 (0·4%)	1 (5·0%)	**0·006**
Bleeding	2 (0·3%)	0 (0·0%)	1 (0·2%)	1 (5·0%)	**<0·001**
Persistent intestinal (duodenal/jejunal) dilatation	1 (0·1%)	0 (0·0%)	1 (0·2%)	0 (0·0%)	0·844
NEC	1 (0·1%)	1 (0·7%)	0 (0·0%)	0 (0·0%)	0·175
Persisting obstruction requiring redo anastomosis	1 (0·1%)	1 (0·7%)	0 (0·0%)	0 (0·0%)	0·175
Parastomal hernia	0 (0·0%)	0 (0·0%)	0 (0·0%)	0 (0·0%)	-
Other	17 (2·5%)	2 (1·3%)	15 (2·9%)	0 (0·0%)	0·405
Was the patient followed up at 30-days post primary surgery or intervention to assess for complications?					
Yes: reviewed in person	308 (55·5%)	81 (55·1%)	225 (56·3%)	2 (25·0%)	**<0·001**
Yes: via telephone consultation	49 (8·8%)	3 (2·0%)	45 (11·3%)	1 (12·5%)	-
Yes: via other means	13 (2·3%)	3 (2·0%)	10 (2·5%)	0 (0·0%)	-
Yes: still an in-patient at 30-days	107 (19·3%)	42 (28·6%)	65 (16·3%)	0 (0·0%)	-
No: data is based on in-patient observations only	44 (7·9%)	12 (8·2%)	30 (7·5%)	2 (25·0%)	-
No: follow-up was done, but prior to 30-days	34 (6·1%)	6 (4·1%)	25 (6·3%)	3 (37·5%)	-
If the patient had a complication, when was it diagnosed?					
During the primary admission	200 (29·4%)	31 (20·4%)	159 (31·2%)	10 (50·0%)	**0·010**
As an emergency re-attender	12 (1·8%)	1 (0·7%)	11 (2·2%)	0 (0·0%)	-
At routine follow-up as an out-patient	12 (1·8%)	1 (0·7%)	10 (2·0%)	1 (5·0%)	-
Not applicable, no complications	453 (66·5%)	119 (78·3%)	325 (63·9%)	9 (45·0%)	-
Missing	4 (0·6%)	0 (0·0%)	4 (0·8%)	0 (0·0%)	-

*Patients born in hospital = 0. Percentages have been rounded to 1 decimal place and may not total 100·0%. HIC: High-income countries. IQR: Interquartile range. LIC: Low-income countries. MIC: Middle-income countries. NEC: Necrotising enterocolitis.

Supplementary Table 4: Characteristics, perioperative care, surgical interventions, and outcomes for patients with gastroschisis

Variable	Total (n=453)	HIC (n=139)	MIC (n=304)	LIC (n=10)	P value
**Patient Characteristics:**					
Median gestational age at birth (IQR), weeks	36 (2)	36 (2)	37 (3)	36 (4)	0·099
Median age at presentation (IQR), hours	0 (10)	0 (0)	2 (20)	12 (12)	**<0·001**
Sex:					
Male	232 (51·2%)	73 (52·4%)	152 (50·0%)	7 (70·0%)	0·430
Female	221 (48·8%)	66 (47·5%)	152 (50·0%)	3 (30·0%)	
Median weight at presentation (IQR), kg	2·3 (0·7)	2·5 (0·7)	2·2 (0·6)	2·2 (1·2)	**<0·001**
Does the patient have another anomaly in addition to the study condition?					
Yes: Cardiovascular	44 (9·7%)	16 (11·5%)	28 (9·2%)	0 (0·0%)	0·433
Yes: Respiratory	12 (2·6%)	2 (1·4%)	10 (3·3%)	0 (0·0%)	0·462
Yes: Gastrointestinal	46 (10·2%)	4 (2·9%)	42 (13·8%)	0 (0·0%)	**0·001**
Yes: Neurological	3 (0·7%)	1 (0·7%)	2 (0·7%)	0 (0·0%)	0·964
Yes: Genito-urinary	13 (2·9%)	2 (1·4%)	11 (3·6%)	0 (0·0%)	0·381
Yes: Musculoskeletal	3 (0·7%)	1 (0·7%)	2 (0·7%)	0 (0·0%)	0·964
Yes: Down syndrome	0 (0·0%)	0 (0·0%)	0 (0·0%)	0 (0·0%)	-
Yes: Beckwith Wiedemann syndrome	0 (0·0%)	0 (0·0%)	0 (0·0%)	0 (0·0%)	-
Yes: Cystic fibrosis	3 (0·7%)	1 (0·7%)	2 (0·7%)	0 (0·0%)	0·964
Yes: Chromosomal	1 (0·2%)	1 (0·7%)	0 (0·0%)	0 (0·0%)	0·322
Yes: Other	14 (3·1%)	4 (2·9%)	10 (3·3%)	0 (0·0%)	0·827
No	340 (75·1%)	112 (80·6%)	218 (71·7%)	10 (100·0%)	**0·025**
Median distance from patient's home to hospital (IQR), km*	2 (58)	0 (13)	10 (91)	52 (94)	**<0·001**
Type of delivery:					
Vaginal (spontaneous)	176 (38·9%)	45 (32·4%)	122 (40·1%)	9 (90·0%)	**<0·001**
Vaginal (induced)	26 (5·7%)	22 (15·8%)	4 (1·3%)	0 (0·0%)	-
Caesarean section (elective)	123 (27·2%)	36 (25·9%)	87 (28·6%)	0 (0·0%)	-
Caesarean section (urgent/non-elective)	128 (28·3%)	36 (25·9%)	91 (29·9%)	1 (10·0%)	-
Was the patient septic on arrival to your hospital?					
Yes	62 (13·7%)	5 (3·6%)	57 (18·8%)	0 (0·0%)	**<0·001**
No	390 (86·1%)	134 (96·4%)	246 (80·9%)	10 (100·0%)	-
Missing	1 (0·2%)	0 (0·0%)	1 (0·3%)	0 (0·0%)	-
Was the patient hypovolaemic on arrival to your hospital?					
Yes	99 (21·9%)	14 (10·1%)	84 (27·6%)	1 (10·0%)	**<0·001**
No	353 (77·9%)	125 (89·9%)	219 (72·0%)	9 (90·0%)	-
Missing	1 (0·2%)	0 (0·0%)	1 (0·3%)	0 (0·0%)	-
Was the patient hypothermic on arrival to your hospital?					
Yes	90 (19·9%)	7 (5·0%)	81 (26·6%)	2 (20·0%)	**<0·001**
No	362 (79·9%)	132 (95·0%)	222 (73·0%)	8 (80·0%)	-
Missing	1 (0·2%)	0 (0·0%)	1 (0·3%)	0 (0·0%)	-
American Society of Anaesthesiologists (ASA) Score at the time of primary intervention:					
1. Healthy person	61 (13·5%)	21 (15·1%)	37 (12·2%)	3 (30·0%)	**<0·001**
2. Mild systemic disease	127 (28·0%)	35 (25·2%)	91 (29·9%)	1 (10·0%)	-
3. Severe systemic disease	172 (38·0%)	65 (46·8%)	106 (34·9%)	1 (10·0%)	-
4. Severe systemic disease that is a constant threat to life	50 (11·0%)	12 (8·6%)	38 (12·5%)	0 (0·0%)	-
5. A moribund patient who is not expected to survive without the operation	13 (2·9%)	1 (0·7%)	12 (3·9%)	0 (0·0%)	-
Not applicable - no intervention	30 (6·6%)	5 (3·6%)	20 (6·6%)	5 (50·0%)	-
What study condition does the patient have?					
Oesophageal atresia	0 (0·0%)	0 (0·0%)	0 (0·0%)	0 (0·0%)	-
Congenital diaphragmatic hernia	0 (0·0%)	0 (0·0%)	0 (0·0%)	0 (0·0%)	-
Intestinal atresia	17 (3·8%)	8 (5·8%)	9 (3·0%)	0 (0·0%)	0·292
Gastroschisis	453 (100·0%)	139 (100·0%)	304 (100·0%)	10 (100·0%)	-
Exomphalos/Omphalocele	0 (0·0%)	0 (0·0%)	0 (0·0%)	0 (0·0%)	-
Anorectal malformation	0 (0·0%)	0 (0·0%)	0 (0·0%)	0 (0·0%)	-
Hirschsprung's Disease	1 (0·2%)	0 (0·0%)	1 (0·3%)	0 (0·0%)	0·782
Type of Gastroschisis?					
Simple	351 (77·5%)	106 (76·3%)	239 (78·6%)	6 (60·0%)	0·351
Complex: associated with atresia	44 (9·7%)	13 (9·4%)	30 (9·9%)	1 (10·0%)	0·985
Complex: associated with necrosis	26 (5·7%)	10 (7·2%)	15 (4·9%)	1 (10·0%)	0·537
Complex: associated with perforation	19 (4·2%)	7 (5·0%)	11 (3·6%)	1 (10·0%)	0·513
Complex: associated with closing gastroschisis	29 (6·4%)	9 (6·5%)	19 (6·3%)	1 (10·0%)	0·892
**Care prior to presentation at the paediatric surgery centre:**					
Antenatal ultrasound undertaken?					
Yes: study condition diagnosed	281 (62·0%)	132 (95·0%)	148 (48·7%)	1 (10·0%)	**<0·001**
Yes: problem identified but study condition not diagnosed	17 (3·8%)	2 (1·4%)	15 (4·9%)	0 (0·0%)	-
Yes: no problem identified	90 (19·9%)	1 (0·7%)	85 (28·0%)	4 (40·0%)	-
No	65 (14·3%)	4 (2·9%)	56 (18·4%)	5 (50·0%)	-

Median gestational age of study condition diagnosis if diagnosis was antenatal (IQR), weeks	22 (16)	19 (12)	26 (13)	-	**<0·001**
Mode of transport to hospital:					
Ambulance	137 (30·2%)	23 (16·5%)	107 (35·2%)	7 (70·0%)	**<0·001**
Other transport provided by the health service	14 (3·1%)	4 (2·9%)	10 (3·3%)	0 (0·0%)	-
Patient's own transport	58 (12·8%)	0 (0·0%)	56 (18·4%)	2 (20·0%)	-
Born within the hospital	244 (53·9%)	112 (80·6%)	131 (43·1%)	1 (10·0%)	-
If out born, where did the patient present from?					
Home	20 (9·6%)	0 (0·0%)	20 (11·6%)	0 (0·0%)	**0·017**
Community Clinic/General Practice	42 (20·1%)	1 (3·7%)	38 (22·0%)	3 (33·3%)	-
District Hospital	143 (68·4%)	25 (92·6%)	112 (64·7%)	6 (66·7%)	-
From a different speciality within the study centre	1 (0·5%)	1 (3·7%)	0 (0·0%)	0 (0·0%)	-
Unknown	2 (1·0%)	0 (0·0%)	2 (1·2%)	0 (0·0%)	-
Missing	1 (0·5%)	0 (0·0%)	1 (0·6%)	0 (0·0%)	-
**Perioperative care at the paediatric surgery centre:**					
If septic, were appropriate antibiotics administered?					
Yes within 1 hour of arrival	48 (77·4%)	4 (80·0%)	44 (77·2%)	0 (0·0%)	0·910
Yes: within the first day of arrival	12 (19·4%)	1 (20·0%)	11 (19·3%)	0 (0·0%)	-
No	2 (3·2%)	0 (0·0%)	2 (3·5%)	0 (0·0%)	-
If hypovolaemic, was an intravenous fluid bolus given?					
Yes within 1 hour of arrival	88 (88·9%)	11 (78·6%)	77 (91·7%)	0 (0·0%)	**0·006**
Yes: within the first day of arrival	11 (11·1%)	3 (21·4%)	7 (8·3%)	1 (100·0%)	-
No	0 (0·0%)	0 (0·0%)	0 (0·0%)	0 (0·0%)	-
If hypovolaemic, how much intravenous fluid was given?					
10 - 20mls/kg	78 (78·8%)	7 (50·0%)	70 (83·3%)	1 (100·0%)	**0·016**
Above 20mls/kg	21 (21·2%)	7 (50·0%)	14 (16·7%)	0 (0·0%)	-
If hypothermic, was the patient warmed on arrival to your hospital to within a normal temperature range?					
Yes	86 (95·6%)	6 (85·7%)	78 (96·3%)	2 (100·0%)	0·410
No	4 (4·4%)	1 (14·3%)	3 (3·7%)	0 (0·0%)	-
Did the patient receive central venous access?					
Yes: umbilical catheter	14 (3·1%)	4 (2·9%)	10 (3·3%)	0 (0·0%)	0·827
Yes: peripherally inserted central catheter (PICC)	231 (51·0%)	101 (72·7%)	129 (42·4%)	1 (10·0%)	**<0·001**
Yes: percutaneously inserted central line with ultrasound guidance	70 (15·5%)	30 (21·6%)	40 (13·2%)	0 (0·0%)	**0·029**
Yes: surgically placed central line (open insertion)	66 (14·6%)	11 (7·9%)	55 (18·1%)	0 (0·0%)	**0·008**
No	107 (23·6%)	4 (2·9%)	94 (30·9%)	9 (90·0%)	**<0·001**
Median total duration of antibiotics following primary intervention (IQR), days	7 (11)	6 (6)	9 (13)	2 (4)	**<0·001**
Did the patient receive a blood transfusion?					
Yes: not cross-matched	3 (0·7%)	0 (0·0%)	3 (1·0%)	0 (0·0%)	**0·001**
Yes: cross-matched.	187 (41·3%)	39 (28·1%)	146 (48·0%)	2 (20·0%)	-
No: not required.	254 (56·1%)	98 (70·5%)	148 (48·7%)	8 (80·0%)	-
No: it was required but not available.	9 (2·0%)	2 (1·4%)	7 (2·3%)	0 (0·0%)	-
Did the patient require ventilation?					
Yes: and it was given	342 (75·5%)	125 (89·9%)	216 (71·1%)	1 (10·0%)	**<0·001**
Yes, but it was not available	29 (6·4%)	0 (0·0%)	28 (9·2%)	1 (10·0%)	-
No	82 (18·1%)	14 (10·1%)	60 (19·7%)	8 (80·0%)	-
Median time patient remained on ventilation if given (IQR), days	4 (7)	4 (6)	5 (7)	1 (0)	**0·013**
Median time to first enteral feed (post-primary intervention) (IQR), days	11 (11)	10 (8)	13 (10)	0 (0)	**<0·001**
Median time to full enteral feeds (post-primary intervention) (IQR), days	22 (15)	27 (13)	21 (18)	30 (0)	**<0·001**
Did the patient require parenteral nutrition?					
Yes: and it was given	351 (77·5%)	138 (99·3%)	212 (69·7%)	1 (10·0%)	**<0·001**
Yes: and it was sometimes available, but less than required	21 (4·6%)	0 (0·0%)	21 (6·9%)	0 (0·0%)	-
Yes: but it was not available	26 (5·7%)	0 (0·0%)	23 (7·6%)	3 (30·0%)	-
No	55 (12·1%)	1 (0·7%)	48 (15·8%)	6 (60·0%)	-
Median time patient received parenteral nutrition if received (IQR), days	21 (16)	24 (13)	20 (18)	30 (0)	**<0·001**
**Surgical intervention:**					
Primary intervention:					
Primary closure in the operating room (OR)	166 (36·6%)	53 (38·1%)	113 (37·2%)	0 (0·0%)	**<0·001**
Staged closure using a preformed silo	108 (23·8%)	41 (29·5%)	64 (21·1%)	3 (30·0%)	-
Staged closure using a surgical silo (including improvised silo)	83 (18·3%)	17 (12·2%)	64 (21·1%)	2 (20·0%)	-
Staged closure using an Alexis Wound Retractor and Protector	38 (8·4%)	6 (4·3%)	32 (10·5%)	0 (0·0%)	-
Primary closure at the cotside (Bianchi technique)	32 (7·1%)	21 (15·1%)	11 (3·6%)	0 (0·0%)	-
Stoma	3 (0·7%)	0 (0·0%)	3 (1·0%)	0 (0·0%)	-
No intervention undertaken	14 (3·1%)	0 (0·0%)	9 (3·0%)	5 (50·0%)	-
Other method	9 (2·0%)	1 (0·7%)	8 (2·6%)	0 (0·0%)	-
Time from presentation to primary intervention in hours, median (IQR)	4 (6)	2 (3)	5 (10)	3 (5)	**<0·001**
Method of defect closure?					
Fascia and skin closed with sutures	277 (63·4%)	89 (64·0%)	187 (63·8%)	1 (20·0%)	**<0·001**
Sutureless closure with skin edges opposed and dressing applied	45 (10·3%)	25 (18·0%)	20 (6·8%)	0 (0·0%)	-
Just skin closed with sutures, fascia left open	36 (8·2%)	6 (4·3%)	30 (10·2%)	0 (0·0%)	-

Dressing applied, defect left open to close by secondary intention (+/- cord flap/ cord coverage of defect)	21 (4·8%)	12 (8·6%)	9 (3·1%)	0 (0·0%)	-
Umbilical cord sutured over the defect, fascia left open	14 (3·2%)	3 (2·2%)	11 (3·8%)	0 (0·0%)	-
Patch/mesh closure	3 (0·7%)	0 (0·0%)	3 (1·0%)	0 (0·0%)	-
Other	36 (8·2%)	2 (1·4%)	30 (10·2%)	4 (80·0%)	-
Patient died before the defect was closed	5 (1·1%)	2 (1·4%)	3 (1·0%)	0 (0·0%)	-
Time from admission to abdominal wall closure in days, median (IQR)	2 (5)	1 (6)	2 (5)	2 (0)	0·873
What type of anaesthesia was used for the primary intervention?					
General anaesthesia with endotracheal tube	361 (79·7%)	121 (87·1%)	236 (77·6%)	4 (40·0%)	**<0·001**
General anaesthesia with laryngeal airway	1 (0·2%)	0 (0·0%)	1 (0·3%)	0 (0·0%)	-
Ketamine anaesthesia	1 (0·2%)	0 (0·0%)	1 (0·3%)	0 (0·0%)	-
Spinal/caudal anaesthesia	1 (0·2%)	0 (0·0%)	1 (0·3%)	0 (0·0%)	-
Local anaesthesia only	9 (2·0%)	0 (0·0%)	9 (3·0%)	0 (0·0%)	-
No anaesthesia, just analgesia	44 (9·7%)	15 (10·8%)	29 (9·5%)	0 (0·0%)	-
No anaesthesia, no analgesia	17 (3·8%)	3 (2·2%)	14 (4·6%)	0 (0·0%)	-
Not applicable: no surgery or primary intervention undertaken.	19 (4·2%)	0 (0·0%)	13 (4·3%)	6 (60·0%)	-
Who undertook the anaesthetic for the primary intervention?					
Anaesthetic doctor	337 (74·4%)	94 (67·6%)	241 (79·3%)	2 (20·0%)	**<0·001**
Anaesthetic nurse	4 (0·9%)	0 (0·0%)	2 (0·7%)	2 (20·0%)	-
Medical officer	21 (4·6%)	20 (14·4%)	1 (0·3%)	0 (0·0%)	-
Surgeon	9 (2·0%)	0 (0·0%)	9 (3·0%)	0 (0·0%)	-
Other healthcare professional	20 (4·4%)	11 (7·9%)	9 (3·0%)	0 (0·0%)	-
No anaesthetic undertaken	62 (13·7%)	14 (10·1%)	42 (13·8%)	6 (60·0%)	-
Who undertook the primary intervention?					
Paediatric surgeon (or junior with paediatric surgeon assisting/in the room)	423 (93·4%)	133 (95·7%)	285 (93·8%)	5 (50·0%)	**<0·001**
General surgeon (or junior with general surgeon assisting/in the room)	1 (0·2%)	0 (0·0%)	1 (0·3%)	0 (0·0%)	-
Junior doctor, medical officer or other (without a paediatric or general surgeon assisting/in the room)	5 (1·1%)	2 (1·4%)	3 (1·0%)	0 (0·0%)	-
Trainee surgeon (without a paediatric or general surgeon assisting or in the room)	11 (2·4%)	4 (2·9%)	7 (2·3%)	0 (0·0%)	-
Not applicable - no surgery or primary intervention undertaken.	13 (2·9%)	0 (0·0%)	8 (2·6%)	5 (50·0%)	-
Was a Surgical Safety Checklist used at the time of primary intervention?					
Yes	304 (67·1%)	111 (79·9%)	191 (62·8%)	2 (20·0%)	**<0·001**
No: but it was available	63 (13·9%)	12 (8·6%)	50 (16·4%)	1 (10·0%)	-
No: it was not available	29 (6·4%)	0 (0·0%)	28 (9·2%)	1 (10·0%)	-
Not applicable: a conservative primary intervention was undertaken	37 (8·2%)	16 (11·5%)	21 (6·9%)	0 (0·0%)	-
Not applicable: no surgery or primary intervention undertaken	20 (4·4%)	0 (0·0%)	14 (4·6%)	6 (60·0%)	-
**Outcomes:**					
Did the patient survive to discharge (or 30-days if still an in-patient 30-days following primary intervention)?					
Yes	345 (76·2%)	137 (98·6%)	207 (68·1%)	1 (10·0%)	**<0·001**
No	108 (23·8%)	2 (1·4%)	97 (31·9%)	9 (90·0%)	-
If the patient was discharged prior, were they still alive at 30-days following primary intervention?					
Yes	306 (89·2%)	122 (90·4%)	184 (88·9%)	0 (0·0%)	**<0·001**
No	1 (0·3%)	0 (0·0%)	1 (0·5%)	0 (0·0%)	-
Not followed-up after discharge	9 (2·6%)	3 (2·2%)	5 (2·4%)	1 (100·0%)	-
Followed-up, but not until 30-days post primary intervention	27 (7·9%)	10 (7·4%)	17 (8·2%)	0 (0·0%)	-
Cause of mortality:					
Sepsis	50 (45·9%)	2 (100·0%)	43 (43·9%)	5 (55·6%)	0·650
Respiratory failure	25 (22·9%)	0 (0·0%)	24 (24·5%)	1 (11·1%)	-
Cardiac failure	15 (13·8%)	0 (0·0%)	15 (15·3%)	0 (0·0%)	-
Electrolyte disturbance	5 (4·6%)	0 (0·0%)	5 (5·1%)	0 (0·0%)	-
Ischaemic bowel	2 (1·8%)	0 (0·0%)	2 (2·0%)	0 (0·0%)	-
Malnutrition	2 (1·8%)	0 (0·0%)	1 (1·0%)	1 (11·1%)	-
Aspiration pneumonia	2 (1·8%)	0 (0·0%)	2 (2·0%)	0 (0·0%)	-
Haemorrhage	1 (0·9%)	0 (0·0%)	1 (1·0%)	0 (0·0%)	-
Anastomotic leak	1 (0·9%)	0 (0·0%)	1 (1·0%)	0 (0·0%)	-
Other	6 (5·5%)	0 (0·0%)	4 (4·1%)	2 (22·2%)	-
Median duration of hospital stays, (IQR) days	22 (18)	29 (8)	20 (24)	6 (6)	**<0·001**
Did the patient have a surgical site infection?					
Yes	51 (11·3%)	19 (13·7%)	30 (9·9%)	2 (20·0%)	**<0·001**
No	368 (81·2%)	115 (82·7%)	250 (82·2%)	3 (30·0%)	-
Not applicable, no surgical wound	34 (7·5%)	5 (3·6%)	24 (7·9%)	5 (50·0%)	-
Did the patient have a full thickness wound dehiscence?					
Yes	21 (4·6%)	3 (2·2%)	18 (5·9%)	0 (0·0%)	**<0·001**
No	399 (88·1%)	130 (93·5%)	265 (87·2%)	4 (40·0%)	-
Not applicable, no surgical wound	33 (7·3%)	6 (4·3%)	21 (6·9%)	6 (60·0%)	-
Did the patient require a further unplanned intervention?					
Yes – percutaneous	5 (1·1%)	3 (2·2%)	2 (0·7%)	0 (0·0%)	**<0·001**
Yes – surgical intervention	58 (12·8%)	19 (13·7%)	38 (12·5%)	1 (10·0%)	-
No	371 (81·9%)	117 (84·2%)	251 (82·6%)	3 (30·0%)	-
Not applicable – no primary intervention undertaken	19 (4·2%)	0 (0·0%)	13 (4·3%)	6 (60·0%)	-

If central line access used, did the patient acquire central line sepsis?					
Yes, diagnosed clinically	14 (4·1%)	6 (4·4%)	8 (3·8%)	0 (0·0%)	0·390
Yes, confirmed on microbiology	49 (14·2%)	13 (9·6%)	36 (17·2%)	0 (0·0%)	-
No	282 (81·7%)	116 (85·9%)	165 (78·9%)	1 (100·0%)	-
Did the neonate have any of these complications within 30-days of primary intervention?					
Abdominal compartment syndrome (ACS)	36 (8·2%)	7 (5·0%)	29 (9·8%)	0 (0·0%)	0·171
Ischemic bowel	26 (5·9%)	8 (5·8%)	17 (5·8%)	1 (20·0%)	0·840
Necrotising enterocolitis	18 (4·1%)	10 (7·2%)	8 (2·7%)	0 (0·0%)	0·060
None of these	371 (84·5%)	121 (87·1%)	246 (83·4%)	4 (80·0%)	**0·001**
If the patient has ACS, was the abdomen re-opened?					
Yes	11 (30·6%)	5 (71·4%)	6 (20·7%)	-	**0·009**
No	25 (69·4%)	2 (28·6%)	23 (79·3%)	-	-
Was the patient followed up at 30-days post primary surgery or intervention to a assess for complications?					
Yes: reviewed in person	179 (51·9%)	56 (40·9%)	123 (59·4%)	0 (0·0%)	**0·005**
Yes: via telephone consultation	14 (4·1%)	7 (5·1%)	7 (3·4%)	0 (0·0%)	-
Yes: via other means	10 (2·9%)	4 (2·9%)	6 (2·9%)	0 (0·0%)	-
Yes: still an in-patient at 30-days	104 (30·1%)	55 (40·1%)	49 (23·7%)	0 (0·0%)	-
No: data is based on in-patient observations only	20 (5·8%)	9 (6·6%)	10 (4·8%)	1 (100·0%)	-
No: follow-up was done, but prior to 30-days	18 (5·2%)	6 (4·4%)	12 (5·8%)	0 (0·0%)	-
If the patient had a complication, when was it diagnosed?					
During the primary admission	164 (36·2%)	41 (29·5%)	120 (39·5%)	3 (30·0%)	**0·007**
As an emergency re-attender	13 (2·9%)	2 (1·4%)	9 (3·0%)	2 (20·0%)	-
At routine follow-up as an out-patient	2 (0·4%)	0 (0·0%)	2 (0·7%)	0 (0·0%)	-
Not applicable, no complications	273 (60·3%)	96 (69·1%)	172 (56·6%)	5 (50·0%)	-
Missing	1 (0·2%)	0 (0·0%)	1 (0·3%)	0 (0·0%)	-

*Patients born in hospital = 0. Percentages have been rounded to 1 decimal place and may not total 100·0%. HIC: High-income countries. IQR: Interquartile range. LIC: Low-income countries. MIC: Middle-income countries.

Supplementary Table 5: Characteristics, perioperative care, surgical interventions, and outcomes for patients with exomphalos/omphalocele

Variable	Total (n=325)	HIC (n=70)	MIC (n=241)	LIC (n=14)	P value
**Patient Characteristics:**					
Median gestational age at birth (IQR), weeks	38 (3)	38 (4)	38 (3)	37 (2)	0·472
Median age at presentation (IQR), hours	3 (23)	0 (2)	6 (24)	13 (40)	**<0·001**
Sex:					
Male	183 (56·3%)	43 (61·4%)	131 (54·4%)	9 (64·3%)	0·780
Female	141 (43·4%)	27 (38·6%)	109 (45·2%)	5 (35·7%)	-
Ambiguous	1 (0·3%)	0 (0·0%)	1 (0·4%)	0 (0·0%)	-
Median weight at presentation (IQR), kg	2·8 (0·9)	2·8 (0·9)	2·7 (0·9)	2·7 (0·7)	0·589
Does the patient have another anomaly in addition to the study condition?					
Yes: Cardiovascular	114 (35·1%)	29 (41·4%)	85 (35·3%)	0 (0·0%)	**0·012**
Yes: Respiratory	16 (4·9%)	8 (11·4%)	8 (3·3%)	0 (0·0%)	**0·015**
Yes: Gastrointestinal	49 (15·1%)	11 (15·7%)	37 (15·4%)	1 (7·1%)	0·696
Yes: Neurological	21 (6·5%)	8 (11·4%)	11 (4·6%)	2 (14·3%)	0·058
Yes: Genito-urinary	52 (16·0%)	12 (17·1%)	38 (15·8%)	2 (14·3%)	0·947
Yes: Musculoskeletal	27 (8·3%)	8 (11·4%)	17 (7·1%)	2 (14·3%)	0·359
Yes: Down syndrome	4 (1·2%)	1 (1·4%)	1 (0·4%)	2 (14·3%)	**<0·001**
Yes: Beckwith Wiedemann syndrome	6 (1·8%)	3 (4·3%)	3 (1·2%)	0 (0·0%)	0·218
Yes: Cystic fibrosis	0 (0·0%)	0 (0·0%)	0 (0·0%)	0 (0·0%)	-
Yes: Chromosomal	11 (3·4%)	5 (7·1%)	5 (2·1%)	1 (7·1%)	0·087
Yes: Other	24 (7·4%)	7 (10·0%)	17 (7·1%)	0 (0·0%)	0·396
No	137 (42·2%)	27 (38·6%)	103 (42·7%)	7 (50·0%)	0·685
Median distance from patient's home to hospital (IQR), km*	13 (68)	0 (17)	16 (77)	56 (111)	**<0·001**
Type of delivery:					
Vaginal (spontaneous)	116 (35·7%)	16 (22·9%)	89 (36·9%)	11 (78·6%)	**0·015**
Vaginal (induced)	12 (3·7%)	4 (5·7%)	7 (2·9%)	1 (7·1%)	-
Caesarean section (elective)	130 (40·0%)	33 (47·1%)	96 (39·8%)	1 (7·1%)	-
Caesarean section (urgent/non-elective)	66 (20·3%)	17 (24·3%)	48 (19·9%)	1 (7·1%)	-
Unknown	1 (0·3%)	0 (0·0%)	1 (0·4%)	0 (0·0%)	-
Was the patient septic on arrival to your hospital?					
Yes	40 (12·3%)	1 (1·4%)	35 (14·5%)	4 (28·6%)	**0·002**
No	285 (87·7%)	69 (98·6%)	206 (85·5%)	10 (71·4%)	-
Was the patient hypovolaemic on arrival to your hospital?					
Yes	29 (8·9%)	5 (7·1%)	22 (9·1%)	2 (14·3%)	**0·002**
No	296 (91·1%)	65 (92·9%)	219 (90·9%)	12 (85·7%)	-
Was the patient hypothermic on arrival to your hospital?					
Yes	32 (9·8%)	4 (5·7%)	25 (10·4%)	3 (21·4%)	**0·002**
No	293 (90·2%)	66 (94·3%)	216 (89·6%)	11 (78·6%)	-
American Society of Anaesthesiologists (ASA) Score at the time of primary intervention:					
1. Healthy person	48 (14·8%)	6 (8·6%)	39 (16·2%)	3 (21·4%)	**<0·001**
2. Mild systemic disease	125 (38·5%)	19 (27·1%)	104 (43·2%)	2 (14·3%)	-
3. Severe systemic disease	72 (22·2%)	24 (34·3%)	48 (19·9%)	0 (0·0%)	-
4. Severe systemic disease that is a constant threat to life	15 (4·6%)	5 (7·1%)	10 (4·1%)	0 (0·0%)	-
5. A moribund patient who is not expected to survive without the operation	8 (2·5%)	1 (1·4%)	7 (2·9%)	0 (0·0%)	-
Not applicable - no intervention	55 (16·9%)	14 (20·0%)	32 (13·3%)	9 (64·3%)	-
Missing	2 (0·6%)	1 (1·4%)	1 (0·4%)	0 (0·0%)	-
What study condition does the patient have?					
Oesophageal atresia	3 (0·9%)	1 (1·4%)	2 (0·8%)	0 (0·0%)	0·840
Congenital diaphragmatic hernia	1 (0·3%)	1 (1·4%)	0 (0·0%)	0 (0·0%)	0·161
Intestinal atresia	8 (2·3%)	3 (4·3%)	5 (2·1%)	0 (0·0%)	-
Gastroschisis	0 (0·0%)	0 (0·0%)	0 (0·0%)	0 (0·0%)	-
Exomphalos/Omphalocele	325 (100·0%)	70 (100·0%)	241 (100·0%)	14 (100·0%)	-
Anorectal malformation	15 (4·6%)	3 (4·3%)	12 (5·0%)	0 (0·0%)	0·681
Hirschsprung's Disease	0 (0·0%)	0 (0·0%)	0 (0·0%)	0 (0·0%)	-
Type of Exomphalos?					
Major	148 (45·5%)	28 (40·0%)	116 (48·1%)	4 (28·6%)	0·190
Minor	175 (53·8%)	42 (60·0%)	123 (51·0%)	10 (71·4%)	-
Missing	2 (0·6%)	0 (0·0%)	2 (0·8%)	0 (0·0%)	-
Hypoglycaemic on arrival?					
Yes	39 (12·0%)	15 (21·4%)	24 (10·0%)	0 (0·0%)	**<0·001**
No	242 (74·5%)	53 (75·7%)	183 (75·9%)	6 (42·9%)	-
Blood glucose not measured	43 (13·2%)	2 (2·9%)	33 (13·7%)	8 (57·1%)	-
Missing	1 (0·3%)	0 (0·0%)	1 (0·4%)	0 (0·0%)	-
Did the patient have a ruptured sac?					
Yes	34 (10·5%)	6 (8·6%)	27 (11·2%)	1 (7·1%)	0·760

No	288 (88·6%)	64 (91·4%)	212 (88·0%)	12 (85·7%)	-
Missing	3 (0·9%)	0 (0·0%)	2 (0·8%)	1 (7·1%)	-
**Care prior to presentation at the paediatric surgery centre:**					
Antenatal ultrasound undertaken?					
Yes: study condition diagnosed	158 (48·6%)	57 (81·4%)	101 (41·9%)	0 (0·0%)	**<0·001**
Yes: problem identified but study condition not diagnosed	24 (7·4%)	8 (11·4%)	16 (6·6%)	0 (0·0%)	-
Yes: no problem identified	95 (29·2%)	4 (5·7%)	85 (35·3%)	6 (42·9%)	-
No	48 (14·8%)	1 (1·4%)	39 (16·2%)	8 (57·1%)	-
Median gestational age of study condition diagnosis if diagnosis was antenatal (IQR), weeks	23 (15)	21 (18)	24 (13)	-	0·999
Mode of transport to hospital:					
Ambulance	118 (36·3%)	20 (28·6%)	89 (36·9%)	9 (64·3%)	**<0·001**
Other transport provided by the health service	17 (5·2%)	6 (8·6%)	10 (4·1%)	1 (7·1%)	-
Patient's own transport	79 (24·3%)	0 (0·0%)	75 (31·1%)	4 (28·6%)	-
Born within the study hospital	111 (34·2%)	44 (62·9%)	67 (27·8%)	0 (0·0%)	-
If out born, where did the patient present from?					
Home	21 (9·8%)	0 (0·0%)	21 (12·1%)	0 (0·0%)	**0·036**
Community Clinic/General Practice	35 (16·4%)	0 (0·0%)	32 (18·4%)	3 (21·4%)	-
District Hospital	155 (72·4%)	26 (100·0%)	118 (67·8%)	11 (78·6%)	-
Unknown	2 (0·9%)	0 (0·0%)	2 (1·1%)	0 (0·0%)	-
Missing	1 (0·5%)	0 (0·0%)	1 (0·6%)	0 (0·0%)	-
**Perioperative care at the paediatric surgery centre:**					
If septic, were appropriate antibiotics administered?					
Yes within 1 hour of arrival	27 (67·5%)	1 (100·0%)	23 (65·7%)	3 (75·0%)	0·73
Yes: within the first day of arrival	13 (32·5%)	0 (0·0%)	12 (34·3%)	1 (25·0%)	-
No	0 (0·0%)	0 (0·0%)	0 (0·0%)	0 (0·0%)	-
If hypovolaemic, was an intravenous fluid bolus given?					
Yes within 1 hour of arrival	19 (65·5%)	2 (40·0%)	16 (72·7%)	1 (50·0%)	0·200
Yes: within the first day of arrival	9 (31·0%)	2 (40·0%)	6 (27·3%)	1 (50·0%)	-
No	1 (3·4%)	1 (20·0%)	0 (0·0%)	0 (0·0%)	-
If hypovolaemic, how much intravenous fluid was given?					
10 - 20mls/kg	22 (78·6%)	2 (50·0%)	18 (81·8%)	2 (100·0%)	0·270
Above 20mls/kg	6 (21·4%)	2 (50·0%)	4 (18·2%)	0 (0·0%)	-
If hypothermic, was the patient warmed on arrival to your hospital to within a normal temperature range?					
Yes	25 (78·1%)	3 (75·0%)	19 (76·0%)	3 (100·0%)	0·630
No	7 (21·9%)	1 (25·0%)	6 (24·0%)	0 (0·0%)	-
Did the patient receive central venous access?					
Yes: umbilical catheter	6 (1·8%)	4 (5·7%)	2 (0·8%)	0 (0·0%)	**0·025**
Yes: peripherally inserted central catheter (PICC)	103 (31·7%)	35 (50·0%)	67 (27·8%)	1 (7·1%)	**<0·001**
Yes: percutaneously inserted central line with ultrasound guidance	24 (7·4%)	13 (18·6%)	11 (4·6%)	0 (0·0%)	**<0·001**
Yes: surgically placed central line (open insertion)	16 (4·9%)	2 (2·9%)	14 (5·8%)	0 (0·0%)	0·413
No	184 (56·6%)	21 (30·0%)	150 (62·2%)	13 (92·9%)	**<0·001**
Median total duration of antibiotics following primary intervention (IQR), days	7 (10)	3 (12)	7 (11)	4 (7)	**0·001**
Did the patient receive a blood transfusion?					
Yes: not cross-matched	4 (1·2%)	2 (2·9%)	2 (0·8%)	0 (0·0%)	0·380
Yes: cross-matched.	83 (25·5%)	17 (24·3%)	65 (27·0%)	1 (7·1%)	-
No: not required.	233 (71·7%)	51 (72·9%)	169 (70·1%)	13 (92·9%)	-
No: it was required but not available.	4 (1·2%)	0 (0·0%)	4 (1·7%)	0 (0·0%)	-
Missing	1 (0·3%)	0 (0·0%)	1 (0·4%)	0 (0·0%)	-
Did the patient require ventilation?					
Yes: and it was given	144 (44·3%)	48 (68·6%)	96 (39·8%)	0 (0·0%)	**<0·001**
Yes, but it was not available	6 (1·8%)	0 (0·0%)	6 (2·5%)	0 (0·0%)	
No	175 (53·8%)	22 (31·4%)	139 (57·7%)	14 (100·0%)	
Median time patient remained on ventilation if given (IQR), days	4 (10)	6 (11)	4 (8)	-	0·389
Median time to first enteral feed (post-primary intervention) (IQR), days	3 (4)	3 (7)	3 (4)	3 (2)	0·821
Median time to full enteral feeds (post-primary intervention) (IQR), days	6 (12)	12 (22)	5 (9)	30 (29)	**0·001**
Did the patient require parenteral nutrition?					
Yes: and it was given	154 (47·4%)	54 (77·1%)	100 (41·5%)	0 (0·0%)	**<0·001**
Yes: and it was sometimes available, but less than required	8 (2·5%)	0 (0·0%)	8 (3·3%)	0 (0·0%)	-
Yes: but it was not available	5 (1·5%)	0 (0·0%)	4 (1·7%)	1 (7·1%)	-
No	158 (48·6%)	16 (22·9%)	129 (53·5%)	13 (92·9%)	-
Median time patient received parenteral nutrition if received (IQR), days	11 (15)	13 (24)	10 (14)	-	0·199
**Surgical intervention:**					
Median time from arrival at your hospital to primary intervention (IQR), hours	11 (23)	12 (21)	10 (27)	10 (58)	0·902
Primary intervention:					
Primary operative closure	164 (50·5%)	41 (58·6%)	119 (49·4%)	4 (28·6%)	0·081
Conservative management	120 (36·9%)	18 (25·7%)	97 (40·2%)	5 (35·7%)	-
Staged closure	32 (9·8%)	11 (15·7%)	21 (8·7%)	0 (0·0%)	-
Missing	9 (2·8%)	0 (0·0%)	4 (1·7%)	5 (35·7%)	-

If conservative management, was a topical treatment applied to the exomphalos sac?					
Yes: silver sulfadiazine	39 (32·5%)	4 (22·2%)	35 (36·1%)	0 (0·0%)	0·310
Yes: betadine	9 (7·5%)	2 (11·1%)	6 (6·2%)	1 (20·0%)	-
Yes: honey	11 (9·2%)	1 (5·6%)	10 (10·3%)	0 (0·0%)	-
Yes: merbromide tannage	2 (1·7%)	0 (0·0%)	2 (2·1%)	0 (0·0%)	-
Yes: other	45 (37·5%)	7 (38·9%)	36 (37·1%)	2 (40·0%)	-
No	14 (11·7%)	4 (22·2%)	8 (8·2%)	2 (40·0%)	-
If staged closure, median time from primary intervention to closure, IQR days	19 (22)	8 (18)	22 (16)	-	**0·051**
What is the plan for future management?					
No further surgery planned	22 (18·3%)	7 (38·9%)	12 (12·4%)	3 (60·0%)	**0·036**
Delayed closure at this hospital	88 (73·3%)	10 (55·6%)	76 (78·4%)	2 (40·0%)	-
Delayed closure at another hospital	2 (1·7%)	0 (0·0%)	2 (2·1%)	0 (0·0%)	-
Patient died during primary admission	8 (6·7%)	1 (5·6%)	7 (7·2%)	0 (0·0%)	-
What type of anaesthesia was used for the primary intervention?					
General anaesthesia with endotracheal tube	200 (61·5%)	47 (67·1%)	149 (61·8%)	4 (28·6%)	**<0·001**
General anaesthesia with laryngeal airway	0 (0·0%)	0 (0·0%)	0 (0·0%)	0 (0·0%)	-
Ketamine anaesthesia	1 (0·3%)	0 (0·0%)	1 (0·4%)	0 (0·0%)	-
Spinal/caudal anaesthesia	0 (0·0%)	0 (0·0%)	0 (0·0%)	0 (0·0%)	-
Local anaesthesia only	2 (0·6%)	0 (0·0%)	2 (0·8%)	0 (0·0%)	-
No anaesthesia, just analgesia	14 (4·3%)	10 (14·3%)	4 (1·7%)	0 (0·0%)	-
No anaesthesia, no analgesia	42 (12·9%)	3 (4·3%)	39 (16·2%)	0 (0·0%)	-
Not applicable: no surgery or primary intervention undertaken.	65 (20·0%)	10 (14·3%)	45 (18·7%)	10 (71·4%)	-
Missing	1 (0·3%)	0 (0·0%)	1 (0·4%)	0 (0·0%)	-
Who undertook the anaesthetic for the primary intervention?					
Anaesthetic doctor	193 (59·4%)	45 (64·3%)	147 (61·0%)	1 (7·1%)	**<0·001**
Anaesthetic nurse	4 (1·2%)	0 (0·0%)	1 (0·4%)	3 (21·4%)	-
Medical officer	1 (0·3%)	1 (1·4%)	0 (0·0%)	0 (0·0%)	-
Surgeon	2 (0·6%)	1 (1·4%)	1 (0·4%)	0 (0·0%)	-
Other healthcare professional	7 (2·2%)	2 (2·9%)	5 (2·1%)	0 (0·0%)	-
No anaesthetic undertaken	117 (36·0%)	21 (30·0%)	86 (35·7%)	10 (71·4%)	-
Missing	1 (0·3%)	0 (0·0%)	1 (0·4%)	0 (0·0%)	-
Who undertook the primary intervention?					
Paediatric surgeon (or junior with paediatric surgeon assisting/in the room)	238 (73·2%)	61 (87·1%)	176 (73·0%)	1 (7·1%)	**<0·001**
General surgeon (or junior with general surgeon assisting/in the room)	2 (0·6%)	0 (0·0%)	0 (0·0%)	2 (14·3%)	-
Junior doctor, medical officer or other (without a paediatric or general surgeon assisting/in the room)	12 (3·7%)	1 (1·4%)	11 (4·6%)	0 (0·0%)	-
Trainee surgeon (without a paediatric or general surgeon assisting or in the room)	8 (2·5%)	0 (0·0%)	7 (2·9%)	1 (7·1%)	-
Not applicable - no surgery or primary intervention undertaken.	64 (19·7%)	8 (11·4%)	46 (19·1%)	10 (71·4%)	-
Missing	1 (0·3%)	0 (0·0%)	1 (0·4%)	0 (0·0%)	-
Was a Surgical Safety Checklist used at the time of primary intervention?					
Yes	171 (52·6%)	48 (68·6%)	120 (49·8%)	3 (21·4%)	**<0·001**
No: but it was available	24 (7·4%)	2 (2·9%)	22 (9·1%)	0 (0·0%)	-
No: it was not available	17 (5·2%)	2 (2·9%)	14 (5·8%)	1 (7·1%)	-
Not applicable: a conservative primary intervention was undertaken	57 (17·5%)	11 (15·7%)	46 (19·1%)	0 (0·0%)	-
Not applicable: no surgery or primary intervention undertaken	55 (16·9%)	7 (10·0%)	38 (15·8%)	10 (71·4%)	-
Missing	1 (0·3%)	0 (0·0%)	1 (0·4%)	0 (0·0%)	-
**Outcomes:**					
Did the patient survive to discharge (or 30-days if still an in-patient 30-days following primary intervention)?					
Yes	260 (80·0%)	58 (82·9%)	192 (79·7%)	10 (71·4%)	**0·600**
No	65 (20·0%)	12 (17·1%)	49 (20·3%)	4 (28·6%)	
If the patient was discharged prior, were they still alive at 30-days following primary intervention?					
Yes	231 (89·2%)	53 (91·4%)	176 (92·1%)	2 (20·0%)	**<0·001**
No	0 (0·0%)	0 (0·0%)	0 (0·0%)	0 (0·0%)	-
Not followed-up after discharge	17 (6·6%)	2 (3·4%)	7 (3·7%)	8 (80·0%)	-
Followed-up, but not until 30-days post primary intervention	11 (4·2%)	3 (5·2%)	8 (4·2%)	0 (0·0%)	-
Cause of mortality:					
Sepsis	21 (32·3%)	0 (0·0%)	20 (40·8%)	1 (25·0%)	0·069
Cardiac failure	15 (23·1%)	3 (25·0%)	10 (20·4%)	2 (50·0%)	-
Respiratory failure	13 (20·0%)	3 (25·0%)	10 (20·4%)	0 (0·0%)	-
Aspiration pneumonia	4 (6·2%)	0 (0·0%)	4 (8·2%)	0 (0·0%)	-
Haemorrhage	3 (4·6%)	1 (8·3%)	2 (4·1%)	0 (0·0%)	-
Ruptured exomphalos sac	2 (3·1%)	1 (8·3%)	1 (2·0%)	0 (0·0%)	-
Electrolyte disturbance	1 (1·5%)	1 (8·3%)	0 (0·0%)	0 (0·0%)	-
Syndrome incompatible with life	1 (1·5%)	1 (8·3%)	0 (0·0%)	0 (0·0%)	-
Other	4 (6·2%)	2 (16·7%)	1 (2·1%)	1 (25·0%)	-
Missing	1 (1·5%)	0 (0·0%)	1 (2·0%)	0 (0·0%)	-
Median duration of hospital stays, (IQR) days	13 (15·0)	16 (22·0)	12 (15·0)	10 (8·0)	0·059
Did the patient have a surgical site infection?					
Yes	32 (9·8%)	7 (10·0%)	25 (10·4%)	0 (0·0%)	**0·025**
No	191 (58·8%)	50 (71·4%)	135 (56·0%)	6 (42·9%)	-

Not applicable, no surgical wound	101 (31·1%)	13 (18·6%)	80 (33·2%)	8 (57·1%)	-
Missing	1 (0·3%)	0 (0·0%)	1 (0·4%)	0 (0·0%)	-
Did the patient have a full thickness wound dehiscence?					
Yes	11 (3·4%)	2 (2·9%)	9 (3·7%)	0 (0·0%)	**0·034**
No	214 (65·8%)	55 (78·6%)	153 (63·5%)	6 (42·9%)	-
Not applicable, no surgical wound	99 (30·5%)	13 (18·6%)	78 (32·4%)	8 (57·1%)	-
Missing	1 (0·3%)	0 (0·0%)	1 (0·4%)	0 (0·0%)	-
Did the patient require a further unplanned intervention?					
Yes – percutaneous	1 (0·3%)	0 (0·0%)	1 (0·4%)	0 (0·0%)	**<0·001**
Yes – surgical intervention	30 (9·2%)	8 (11·4%)	21 (8·7%)	1 (7·1%)	-
No	243 (74·8%)	56 (80·0%)	183 (75·9%)	4 (28·6%)	-
Not applicable – no primary intervention undertaken	50 (15·4%)	6 (8·6%)	35 (14·5%)	9 (64·3%)	-
Missing	1 (0·3%)	0 (0·0%)	1 (0·4%)	0 (0·0%)	-
If central line access required, did the patient acquire central line sepsis?					
Yes, diagnosed clinically	4 (2·9%)	0 (0·0%)	4 (4·4%)	0 (0·0%)	0·520
Yes, confirmed on microbiology	9 (6·4%)	2 (4·1%)	7 (7·8%)	0 (0·0%)	-
No	127 (90·7%)	47 (95·9%)	79 (87·8%)	1 (100·0%)	-
Was the patient followed up at 30-days post primary surgery or intervention to a assess for complications?					
Yes: reviewed in person	165 (63·5%)	33 (56·9%)	130 (67·7%)	2 (20·0%)	**<0·001**
Yes: via telephone consultation	19 (7·3%)	1 (1·7%)	18 (9·4%)	0 (0·0%)	-
Yes: via other means	2 (0·8%)	1 (1·7%)	1 (0·5%)	0 (0·0%)	-
Yes: still an in-patient at 30-days	31 (11·9%)	13 (22·4%)	18 (9·4%)	0 (0·0%)	-
No: data is based on in-patient observations only	24 (9·2%)	5 (8·6%)	13 (6·8%)	6 (60·0%)	-
No: follow-up was done, but prior to 30-days	19 (7·3%)	5 (8·6%)	12 (6·3%)	2 (20·0%)	-
If the patient had a complication, when was it diagnosed?					
During the primary admission	69 (21·2%)	18 (25·7%)	49 (20·3%)	2 (14·3%)	0·660
As an emergency re-attender	6 (1·8%)	1 (1·4%)	5 (2·1%)	0 (0·0%)	-
At routine follow-up as an out-patient	7 (2·2%)	0 (0·0%)	7 (2·9%)	0 (0·0%)	-
Not applicable, no complications	242 (74·5%)	51 (72·9%)	179 (74·3%)	12 (85·7%)	-
Missing	1 (0·3%)	0 (0·0%)	1 (0·4%)	0 (0·0%)	-

*Patients born in hospital = 0. Percentages have been rounded to 1 decimal place and may not total 100·0%. HIC: High-income countries. IQR: Interquartile range. LIC: Low-income countries. MIC: Middle-income countries.

Supplementary Table 6: Characteristics, perioperative care, surgical interventions, and outcomes for patients with anorectal malformation

Variable	Total (n=991)	HIC (n=178)	MIC (n=788)	LIC (n=25)	P value
**Patient Characteristics:**					
Median gestational age at birth (IQR), weeks	38 (2)	39(3)	38(2)	38(3)	**0·003**
Median age at presentation (IQR), hours	24 (68)	7 (27)	24 (67)	96 (696)	**<0·001**
Sex:					
Male	575 (58·0%)	106 (59·6%)	454 (57·6%)	15 (60·0%)	0·850
Female	398 (40·2%)	71 (39·9%)	317 (40·2%)	10 (40·0%)	-
Ambiguous	17 (1·7%)	1 (0·6%)	16 (2·0%)	0 (0·0%)	-
Unknown	1 (0·1%)	0 (0·0%)	1 (0·1%)	0 (0·0%)	-
Median weight at presentation (IQR), kg	3·0 (1·0)	3·0 (0·9)	3·0 (1·0)	3·2 (1·4)	0·125
Does the patient have another anomaly in addition to the study condition?					
Yes: Cardiovascular	324 (32·7%)	88 (49·4%)	232 (29·4%)	4 (16·0%)	**<0·001**
Yes: Respiratory	25 (2·5%)	6 (3·4%)	19 (2·4%)	0 (0·0%)	-
Yes: Gastrointestinal	93 (9·4%)	15 (8·4%)	78 (9·9%)	0 (0·0%)	-
Yes: Neurological	66 (6·7%)	27 (15·2%)	37 (4·7%)	2 (8·0%)	**<0·001**
Yes: Genito-urinary	191 (19·3%)	56 (31·5%)	133 (16·9%)	2 (8·0%)	**<0·001**
Yes: Musculoskeletal	109 (11·0%)	34 (19·1%)	73 (9·3%)	2 (8·0%)	**<0·001**
Yes: Down syndrome	57 (5·8%)	11 (6·2%)	46 (5·8%)	0 (0·0%)	-
Yes: Beckwith Wiedemann syndrome	1 (0·1%)	0 (0·0%)	1 (0·1%)	0 (0·0%)	-
Yes: Cystic fibrosis	1 (0·1%)	0 (0·0%)	1 (0·1%)	0 (0·0%)	-
Yes: Chromosomal	24 (2·4%)	3 (1·7%)	21 (2·7%)	0 (0·0%)	-
Yes: Other	67 (6·8%)	8 (4·5%)	59 (7·5%)	0 (0·0%)	-
No	441 (44·5%)	58 (32·6%)	365 (46·3%)	18 (72·0%)	**<0·001**
Median distance from patient's home to hospital (IQR), km*	32 (93)	20 (76)	35 (100)	80 (113)	**<0·001**
Type of delivery:					
Vaginal (spontaneous)	520 (52·5%)	92 (51·7%)	410 (52·0%)	18 (72·0%)	**0·003**
Vaginal (induced)	42 (4·2%)	12 (6·7%)	30 (3·8%)	0 (0·0%)	-
Caesarean section (elective)	240 (24·2%)	27 (15·2%)	208 (26·4%)	5 (20·0%)	-
Caesarean section (urgent/non-elective)	177 (17·9%)	44 (24·7%)	132 (16·8%)	1 (4·0%)	-
Unknown	12 (1·2%)	3 (1·7%)	8 (1·0%)	1 (4·0%)	-
Was the patient septic on arrival to your hospital?					
Yes	112 (11·3%)	2 (1·1%)	107 (13·6%)	3 (12·0%)	**<0·001**
No	879 (88·7%)	176 (98·9%)	681 (86·4%)	22 (88·0%)	-
Was the patient hypovolaemic on arrival to your hospital?					
Yes	71 (7·2%)	10 (5·6%)	61 (7·7%)	0 (0·0%)	0·230
No	919 (92·7%)	168 (94·4%)	726 (92·1%)	25 (100·0%)	-
Missing	1 (0·1%)	0 (0·0%)	1 (0·1%)	0 (0·0%)	-
Was the patient hypothermic on arrival to your hospital?					
Yes	60 (6·1%)	5 (2·8%)	53 (6·7%)	2 (8·0%)	0·130
No	930 (93·8%)	173 (97·2%)	734 (93·1%)	23 (92·0%)	-
Missing	1 (0·1%)	0 (0·0%)	1 (0·1%)	0 (0·0%)	-
American Society of Anaesthesiologists (ASA) Score at the time of primary intervention:					
1. Healthy person	276 (27·9%)	43 (24·2%)	222 (28·2%)	11 (44·0%)	0·180
2. Mild systemic disease	357 (36·0%)	73 (41·0%)	277 (35·2%)	7 (28·0%)	-
3. Severe systemic disease	172 (17·4%)	32 (18·0%)	136 (17·3%)	4 (16·0%)	-
4. Severe systemic disease that is a constant threat to life	58 (5·9%)	14 (7·9%)	44 (5·6%)	0 (0·0%)	-
5. A moribund patient who is not expected to survive without the operation	32 (3·2%)	2 (1·1%)	30 (3·8%)	0 (0·0%)	-
Not applicable - no intervention	93 (9·4%)	12 (6·7%)	78 (9·9%)	3 (12·0%)	-
Missing	3 (0·3%)	2 (1·1%)	1 (0·1%)	0 (0·0%)	-
What study condition does the patient have?					
Oesophageal atresia	53 (5·3%)	10 (5·6%)	42 (5·3%)	1 (4·0%)	0·940
Congenital diaphragmatic hernia	1 (0·1%)	1 (0·6%)	0 (0·0%)	0 (0·0%)	0·100
Intestinal atresia	12 (1·2%)	3 (1·7%)	9 (1·1%)	0 (0·0%)	0·710
Gastroschisis	0 (0·0%)	0 (0·0%)	0 (0·0%)	0 (0·0%)	-
Exomphalos/Omphalocele	0 (0·0%)	0 (0·0%)	0 (0·0%)	0 (0·0%)	-
Anorectal malformation	991 (100·0%)	178 (100·0%)	788 (100·0%)	25 (100·0%)	-
Hirschsprung's Disease	0 (0·0%)	0 (0·0%)	0 (0·0%)	0 (0·0%)	-
Type of anorectal malformation (Krickenbeck classification)					
Low ARM: Perineal (cutaneous) fistula	327 (33·0%)	73 (41·0%)	252 (32·0%)	2 (8·0%)	**<0·001**
High ARM: Rectourethral fistula (bulbar)	67 (6·8%)	13 (7·3%)	53 (6·7%)	1 (4·0%)	-
High ARM: Rectourethral fistula (prostatic)	33 (3·3%)	16 (9·0%)	17 (2·2%)	0 (0·0%)	-
High ARM: Rectovesical fistula	18 (1·8%)	5 (2·8%)	12 (1·5%)	1 (4·0%)	-
High ARM: Vestibular fistula	152 (15·3%)	24 (13·5%)	127 (16·1%)	1 (4·0%)	-
High ARM: Cloaca	53 (5·3%)	9 (5·1%)	42 (5·3%)	2 (8·0%)	-
High ARM: No fistula	135 (13·6%)	13 (7·3%)	117 (14·8%)	5 (20·0%)	-
High ARM: Type unknown at present	134 (13·5%)	13 (7·3%)	116 (14·7%)	5 (20·0%)	-

Rare variant: Pouch colon	10 (1·0%)	0 (0·0%)	10 (1·3%)	0 (0·0%)	-
Rare variant: Rectal atresia/ stenosis	12 (1·2%)	4 (2·2%)	7 (0·9%)	1 (4·0%)	-
Rare variant: Rectovaginal fistula	16 (1·6%)	3 (1·7%)	9 (1·1%)	4 (16·0%)	-
Rare variant: H fistula	1 (0·1%)	0 (0·0%)	1 (0·1%)	0 (0·0%)	-
Other	32 (3·2%)	5 (2·8%)	24 (3·0%)	3 (12·0%)	-
Missing	1 (0·1%)	0 (0·0%)	1 (0·1%)	0 (0·0%)	-
Did the neonate have pre-operative bowel perforation?					
Yes	37 (3·7%)	1 (0·6%)	36 (4·6%)	0 (0·0%)	**0·023**
No	951 (96·0%)	177 (99·4%)	749 (95·1%)	25 (100·0%)	-
Missing	3 (0·3%)	0 (0·0%)	3 (0·4%)	0 (0·0%)	-
**Care prior to presentation at the paediatric surgery centre:**					
Antenatal ultrasound undertaken?					
Yes: study condition diagnosed	40 (4·0%)	14 (7·9%)	26 (3·3%)	0 (0·0%)	**<0·001**
Yes: problem identified but study condition not diagnosed	121 (12·2%)	35 (19·7%)	85 (10·8%)	1 (4·0%)	-
Yes: no problem identified	662 (66·8%)	117 (65·7%)	527 (66·9%)	18 (72·0%)	-
No	166 (16·8%)	12 (6·7%)	148 (18·8%)	6 2(4·0%)	-
Missing	2 (0·2%)	0 (0·0%)	2 (0·3%)	0 (0·0%)	-
Median gestational age of study condition antenatal diagnosis (IQR), weeks	28 (12)	27·5 (10)	27·5 (13)	-	0·243
Mode of transport to hospital:					
Ambulance	404 (40·8%)	97 (54·5%)	300 (38·1%)	7 (28·0%)	**<0·001**
Other transport provided by the health service	48 (4·8%)	18 (10·1%)	26 (3·3%)	4 (16·0%)	-
Patient's own transport	383 (38·6%)	24 (13·5%)	346 (43·9%)	13 (52·0%)	-
Born within the hospital	156 (15·7%)	39 (21·9%)	116 (14·7%)	1 (4·0%)	-
If outborn, where did the patient present from?					
Home	173 (20·7%)	11 (7·9%)	159 (23·7%)	3 (12·5%)	**<0·001**
Community Clinic/General Practice	130 (15·6%)	22 (15·8%)	102 (15·2%)	6 (25·0%)	-
District Hospital	519 (62·2%)	106 (76·3%)	398 (59·2%)	15 (62·5%)	-
Unknown	12 (1·4%)	0 (0·0%)	12 (1·8%)	0 (0·0%)	-
Missing	1 (0·1%)	0 (0·0%)	1 (0·1%)	0 (0·0%)	-
**Perioperative care at the paediatric surgery centre:**					
If septic, were appropriate antibiotics administered?					
Yes within 1 hour of arrival	84 (75·0%)	1 (50%)	81 (75·7%)	2 (66·7%)	0·898
Yes within the first day of arrival	26(23·2%)	1 (50%)	24 (4·0%)	1 (33·3%)	-
No	2 (1·8%)	0 (0%)	2 (1·9%)	0 (0·0%)	-
If hypovolaemic, was an intravenous fluid bolus given?					
Yes within 1 hour of arrival	57 (80·3%)	5 (50%)	52 (85·3%)	0 (0·0%)	**0·008**
Yes within the first day of arrival	11 (15·5%)	3 (30%)	8 (13·1%)	0 (0·0%)	-
No	3 (4·3%)	2 (20%)	1 (1·6%)	0 (0·0%)	-
If hypovolaemic, how much intravenous fluid was given?					
10 - 20mls/kg	50 (73·5%)	7 (87·5%)	43 (71·7%)	0 (0·0%)	0·340
Above 20mls/kg	18 (26·5%)	1 (12·5%)	17 (28·3%)	0 (0·0%)	-
If hypothermic, was the patient warmed on arrival to your hospital to within a normal temperature range?					
Yes	54 (90·0%)	5 (100·0%)	47 (88·7%)	2 (100·0%)	0·644
No	6 (10%)	0 (0·0%)	6 (11·3%)	0 (0·0%)	-
Did the patient receive central venous access?					
Yes: umbilical catheter	78 (7·9%)	24 (13·5%)	54 (6·9%)	0 (0·0%)	**0·004**
Yes: peripherally inserted central catheter (PICC)	173 (17·5%)	42 (23·6%)	129 (16·4%)	2 (8·0%)	**0·033**
Yes: percutaneously inserted central line with ultrasound guidance	50 (5·0%)	22 (12·4%)	28 (3·6%)	0 (0·0%)	**<0·001**
Yes: surgically placed central line (open insertion)	26 (2·6%)	0 (0·0%)	26 (3·3%)	0 (0·0%)	**0·032**
No	690 (69·6%)	99 (55·6%)	568 (72·1%)	23 (92·0%)	**<0·001**
Median total duration of antibiotics following primary intervention (IQR), days	5 (6)	3 (4)	6 (5)	5 (5)	**0·001**
Did the patient receive a blood transfusion?					
Yes: not cross-matched	7 (0·7%)	0 (0·0%)	7 (0·9%)	0 (0·0%)	**<0·001**
Yes: cross-matched.	187 (18·9%)	15 (8·4%)	166 (21·1%)	6 (24·0%)	-
No: not required.	783 (79·0%)	162 (91·0%)	604 (76·6%)	17 (68·0%)	-
No: it was required but not available.	12 (1·2%)	1 (0·6%)	9 (1·1%)	2 (8·0%)	-
Missing	2 (0·2%)	0 (0·0%)	2 (0·3%)	0 (0·0%)	-
Did the patient require ventilation?					
Yes and it was given	321 (32·4%)	81 (45·5%)	238 (30·2%)	2 (8·0%)	**<0·001**
Yes, but it was not available	12 (1·2%)	0 (0·0%)	10 (1·3%)	2 (8·0%)	-
No	657 (66·3%)	97 (54·5%)	539 (68·4%)	21 (84·0%)	-
Missing	1 (0·1%)	0 (0·0%)	1 (0·1%)	0 (0·0%)	-
Median time patient remained on ventilation if given (IQR), days	2 (3)	2 (2)	2 (4)	3 (3)	0·980
Median time to first enteral feed (post-primary intervention) (IQR), days	2 (3)	2 (3)	2 (3)	1 (1)	**0·003**
Median time to full enteral feeds (post-primary intervention) (IQR), days	4 (5)	5 (6)	4 (5)	2 (1)	**0·013**
Did the patient require parenteral nutrition?					
Yes and it was given	358 (36·1%)	87 (48·9%)	271 (34·4%)	0 (0·0%)	**<0·001**
Yes and it was sometimes available, but less than required	12 (1·2%)	0 (0·0%)	12 (1·5%)	0 (0·0%)	-
Yes, but it was not available	14 (1·4%)	0 (0·0%)	11 (1·4%)	3 (12·0%)	-
No	605 (61·0%)	91 (51·1%)	492 (62·4%)	22 (88·0%)	-

Missing	2 (0·2%)	0 (0·0%)	2 (0·3%)	0 (0·0%)	-
Median time patient received parenteral nutrition if received (IQR), days	7 (7·0)	6 (8·0)	7 (7·0)	0 (0·0)	0·980
**Surgical intervention:**					
Primary intervention:					
Divided sigmoid colostomy	306 (30·9%)	54 (30·3%)	247 (31·3%)	5 (20·0%)	0·470
Anoplasty/anorectoplasty	223 (22·5%)	37 (20·8%)	180 (22·8%)	6 (24·0%)	0·820
Loop sigmoid colostomy	162 (16·3%)	28 (15·7%)	125 (15·9%)	9 (36·0%)	**0·027**
Fistula dilation and/or washout via fistula (no surgery)	94 (9·5%)	29 (16·3%)	62 (7·9%)	3 (12·0%)	**0·002**
Posterior sagittal anorectoplasty (PSARP)	83 (8·4%)	25 (14·0%)	56 (7·1%)	2 (8·0%)	**0·010**
Palliative care/no intervention	46 (4·6%)	3 (1·7%)	41 (5·2%)	2 (8·0%)	0·095
Loop transverse colostomy	41 (4·1%)	3 (1·7%)	37 (4·7%)	1 (4·0%)	0·19
Other stoma	30 (3·0%)	3 (1·7%)	26 (3·3%)	1 (4·0%)	0·500
Divided transverse colostomy	29 (2·9%)	4 (2·2%)	25 (3·2%)	0 (0·0%)	0·55
Abdominoperineal pull-through	9 (0·9%)	3 (1·7%)	6 (0·8%)	0 (0·0%)	0·450
Laparoscopic-assisted pull-through	1 (0·1%)	0 (0·0%)	1 (0·1%)	0 (0·0%)	0·880
Abdominosacroperineal pull-through	1 (0·1%)	0 (0·0%)	1 (0·1%)	0 (0·0%)	0·880
Other	14 (1·4%)	6 (3·4%)	8 (1·0%)	0 (0·0%)	**0·046**
What is the plan for future management?					
Anoplasty/ pull-through planned at study hospital	535 (54·0%)	94 (52·8%)	430 (54·6%)	11 (44·0%)	0·550
Stoma closure planned at study hospital	169 (17·1%)	33 (18·5%)	133 (16·9%)	3 (12·0%)	0·690
No further operative management	67 (6·8%)	13 (7·3%)	50 (6·3%)	4 (16·0%)	0·160
Anoplasty/ pull-through planned at another hospital	28 (2·8%)	5 (2·8%)	23 (2·9%)	0 (0·0%)	0·690
Other surgical procedure	21 (2·1%)	5 (2·8%)	14 (1·8%)	2 (8·0%)	0·081
Patient died or left against medical advice	18 (1·8%)	2 (1·1%)	16 (2·0%)	0 (0·0%)	0·560
Stoma closure planned at another hospital	9 (0·9%)	3 (1·7%)	6 (0·8%)	0 (0·0%)	0·450
Anal dilatation	4 (0·4%)	1 (0·6%)	2 (0·3%)	1 (4·0%)	**0·014**
If primary anorectal reconstruction was undertaken, was a Peña stimulator or equivalent used to identify the position of the muscle complex intra-operatively?					
Yes	206 (67·3%)	56 (87·5%)	147 (62·8%)	3 (37·5%)	**0·001**
No: equipment was not available	67 (21·9%)	3 (4·7%)	60 (25·6%)	4 (50·0%)	-
No: the equipment was available but not used	33 (10·8%)	5 (7·8%)	27 (11·5%)	1 (12·5%)	-
Median time from arrival at your hospital to primary intervention (IQR), hours	24 (36)	24 (19)	24 (37)	36 (72)	0·094
What type of anaesthesia was used for the primary intervention?					
General anaesthesia with endotracheal tube	826 (83·4%)	152 (85·4%)	658 (83·5%)	16 (64·0%)	**<0·001**
General anaesthesia with laryngeal airway	15 (1·5%)	2 (1·1%)	10 (1·3%)	3 (12·0%)	-
Ketamine anaesthesia	3 (0·3%)	0 (0·0%)	1 (0·1%)	2 (8·0%)	-
Spinal/caudal anaesthesia	18 (1·8%)	0 (0·0%)	18 (2·3%)	0 (0·0%)	-
Local anaesthesia only	10 (1·0%)	0 (0·0%)	10 (1·3%)	0 (0·0%)	-
No anaesthesia, just analgesia	8 (0·8%)	3 (1·7%)	5 (0·6%)	0 (0·0%)	-
No anaesthesia, no analgesia	24 (2·4%)	12 (6·7%)	12 (1·5%)	0 (0·0%)	-
Not applicable: no surgery or primary intervention undertaken.	86 (8·7%)	9 (5·1%)	73 (9·3%)	4 (16·0%)	-
Missing	1 (0·1%)	0 (0·0%)	1 (0·1%)	0 (0·0%)	-
Who undertook the anaesthetic for the primary intervention?					
Anaesthetic doctor	847 (85·5%)	155 (87·1%)	680 (86·3%)	12 (48·0%)	**<0·001**
Anaesthetic nurse	14 (1·4%)	0 (0·0%)	7 (0·9%)	7 (28·0%)	-
Medical officer	3 (0·3%)	0 (0·0%)	1 (0·1%)	2 (8·0%)	-
Surgeon	9 (0·9%)	0 (0·0%)	9 (1·1%)	0 (0·0%)	-
Other healthcare professional	2 (0·2%)	1 (0·6%)	1 (0·1%)	0 (0·0%)	-
No anaesthetic undertaken	114 (11·5%)	22 (12·4%)	88 (11·2%)	4 (16·0%)	-
Missing	2 (0·2%)	0 (0·0%)	2 (0·3%)	0 (0·0%)	-
Who undertook the primary intervention?					
Paediatric surgeon (or junior with paediatric surgeon assisting/in the room)	877 (88·5%)	167 (93·8%)	696 (88·3%)	14 (56·0%)	**<0·001**
General surgeon (or junior with general surgeon assisting/in the room)	13 (1·3%)	0 (0·0%)	10 (1·3%)	3 (12·0%)	-
Junior doctor, medical officer or other (without a paediatric or general surgeon assisting/in the room)	3 (0·3%)	0 (0·0%)	1 (0·1%)	2 (8·0%)	-
Trainee surgeon (without a paediatric or general surgeon assisting or in the room)	16 (1·6%)	0 (0·0%)	13 (1·6%)	3 (12·0%)	-
Not applicable - no surgery or primary intervention undertaken.	80 (8·1%)	10 (5·6%)	67 (8·5%)	3 (12·0%)	-
Missing	2 (0·2%)	1 (0·6%)	1 (0·1%)	0 (0·0%)	-
Was a Surgical Safety Checklist used at the time of primary intervention?					
Yes	702 (70·8%)	153 (86·0%)	540 (68·5%)	9 (36·0%)	**<0·001**
No: but it was available	103 (10·4%)	3 (1·7%)	93 (11·8%)	7 (28·0%)	-
No: it was not available	71 (7·2%)	1 (0·6%)	65 (8·2%)	5 (20·0%)	-
Not applicable: a conservative primary intervention was undertaken	33 (3·3%)	13 (7·3%)	20 (2·5%)	0 (0·0%)	-
Not applicable: no surgery or primary intervention undertaken	81 (8·2%)	8 (4·5%)	69 (8·8%)	4 (16·0%)	-
Missing	1 (0·1%)	0 (0·0%)	1 (0·1%)	0 (0·0%)	-
**Outcomes:**					
Did the patient survive to discharge (or 30-days if still an in-patient 30-days following primary intervention)?					
Yes	888 (89·6%)	175 (98·3%)	693 (87·9%)	20 (80·0%)	**<0·001**
No	103 (10·4%)	3 (1·7%)	95 (12·1%)	5 (20·0%)	-
If the patient was discharged prior, were they still alive at 30-days following primary intervention?					
Yes	797 (89·8%)	156 (89·1%)	626 (90·3%)	15 (75·0%)	**0·001**

No	4 (0·4%)	0 (0·0%)	4 (0·6%)	0 (0·0%)	-
Not followed-up after discharge	40 (4·5%)	5 (2·9%)	30 (4·3%)	5 (25·0%)	-
Followed-up, but not until 30-days post primary intervention	44 (5·0%)	14 (8·0%)	30 (4·3%)	0 (0·0%)	-
Missing	3 (0·3%)	0 (0·0%)	3 (0·4%)	0 (0·0%)	-
Cause of mortality:					
Sepsis	39 (36·4%)	1 (33·3%)	36 (36·4%)	2 (40·0%)	0·980
Cardiac failure	30 (28·0%)	1 (33·3%)	28 (28·3%)	1 (20·0%)	-
Respiratory failure	20 (18·7%)	1 (33·3%)	18 (18·2%)	1 (20·0%)	-
Other	8 (7·5%)	0 (0·0%)	7 (7·1%)	1 (20·0%)	-
Aspiration pneumonia	3 (2·8%)	0 (0·0%)	3 (3·0%)	0 (0·0%)	-
Electrolyte disturbance	2 (1·9%)	0 (0·0%)	2 (2·0%)	0 (0·0%)	-
Haemorrhage	2 (1·9%)	0 (0·0%)	2 (2·0%)	0 (0·0%)	-
Ischaemic bowel	1 (0·9%)	0 (0·0%)	1 (1·0%)	0 (0·0%)	-
Enterocolitis	1 (0·9%)	0 (0·0%)	1 (1·0%)	0 (0·0%)	-
Missing	1 (0·9%)	0 (0·0%)	1 (1·0%)	0 (0·0%)	-
Median duration of hospital stay, days	9 (10)	11 (12)	8 (9)	6 (15)	**<0·001**
Did the patient have a surgical site infection?					
Yes	86 (8·7%)	15 (8·4%)	69 (8·8%)	2 (8·0%)	0·990
No	775 (78·2%)	140 (78·7%)	616 (78·2%)	19 (76·0%)	-
Not applicable, no surgical wound	128 (12·9%)	23 (12·9%)	101 (12·8%)	4 (16·0%)	-
Missing	2 (0·2%)	0 (0·0%)	2 (0·3%)	0 (0·0%)	-
Did the patient have a full thickness wound dehiscence?					
Yes	38 (3·8%)	5 (2·8%)	32 (4·1%)	1 (4·0%)	0·710
No	829 (83·7%)	150 (84·3%)	660 (83·8%)	19 (76·0%)	-
Not applicable, no surgical wound	122 (12·3%)	23 (12·9%)	94 (11·9%)	5 (20·0%)	-
Missing	2 (0·2%)	0 (0·0%)	2 (0·3%)	0 (0·0%)	-
Did the patient require a further unplanned intervention?					
Yes – percutaneous	9 (0·9%)	3 (1·7%)	6 (0·8%)	0 (0·0%)	0·300
Yes – surgical intervention	91 (9·2%)	10 (5·6%)	79 (10·0%)	2 (8·0%)	-
No	805 (81·2%)	152 (85·4%)	634 (80·5%)	19 (76·0%)	-
Not applicable – no primary intervention undertaken	83 (8·4%)	13 (7·3%)	66 (8·4%)	4 (16·0%)	-
Missing	3 (0·3%)	0 (0·0%)	3 (0·4%)	0 (0·0%)	-
If central line access was used, did the patient acquire central line sepsis?					
Yes, diagnosed clinically	7 (2·3%)	0 (0·0%)	7 (3·2%)	0 (0·0%)	0·580
Yes, confirmed on microbiology	9 (3·0%)	2 (2·5%)	7 (3·2%)	0 (0·0%)	-
No	289 (94·8%)	79 (97·5%)	208 (93·7%)	2 (100·0%)	-
Electrolyte disturbance within 30 days of primary intervention					
Yes	84 (9·5%)	19 (10·7%)	62 (7·8%)	3 (13·0%)	**<0·001**
No	751 (85·1%)	131 (73·6%)	606 (85·8%)	14 (60·9%)	-
Not applicable	48 (5·4%)	4 (2·2%)	38 (5·4%)	6 (26·1%)	-
High output stoma (over 20mls/kg/day) within 30 days of primary intervention					
Yes	14 (1·6%)	8 (5·2%)	6 (0·8%)	0 (0·0%)	**<0·001**
No	652 (73·9%)	102 (66·2%)	535 (75·8%)	15 (68·2%)	-
Not applicable	216 (24·5%)	44 (28·6%)	165 (23·4%)	7 (31·8%)	-
Stoma prolapse/ retraction/ herniation within 30 days of primary intervention					
Yes	44 (5·0%)	3 (1·9%)	39 (5·5%)	2 (9·1%)	0·110
No	622 (70·5%)	107 (69·5%)	503 (71·2%)	12 (54·5%)	-
Not applicable	216 (24·5%)	44 (28·6%)	164 (23·2%)	8 (36·4%)	-
Peri-stoma skin breakdown (or perianal if primary reconstructive surgery undertaken without a covering stoma) within 30 days of primary intervention					
Yes	63 (7·1%)	10 (6·5%)	52 (7·4%)	1 (4·3%)	0·590
No	631 (71·5%)	106 (68·8%)	510 (72·3%)	15 (65·2%)	-
Not applicable	188 (21·3%)	38 (24·7%)	143 (20·3%)	7 (30·4%)	-
Anal stenosis (in patients undergoing primary anorectal reconstruction without covering stoma) within 30 days of primary intervention					
Yes	13 (1·5%)	4 (2·6%)	9 (1·3%)	0 (0·0%)	**<0·001**
No	551 (62·4%)	106 (68·8%)	441 (62·5%)	4 (17·4%)	-
Not applicable	319 (36·1%)	44 (28·6%)	256 (36·3%)	19 (82·6%)	-
Was the patient followed up at 30-days post primary surgery or intervention to a assess for complications?					
Yes: reviewed in person	531 (59·8%)	112 (64·0%)	415 (59·9%)	4 (20·0%)	**<0·001**
Yes: via telephone consultation	140 (15·8%)	8 (4·6%)	130 (18·8%)	2 (10·0%)	-
Yes: via other means	32 (3·6%)	2 (1·1%)	29 (4·2%)	1 (5·0%)	-
Yes: still an in-patient at 30-days	42 (4·7%)	16 (9·1%)	24 (3·5%)	2 (10·0%)	-
No: data is based on in-patient observations only	78 (8·8%)	11 (6·3%)	60 (8·7%)	7 (35·0%)	-
No: follow-up was done, but prior to 30-days	65 (7·3%)	26 (14·9%)	35 (5·1%)	4 (20·0%)	-
If the patient had a complication, when was it diagnosed?					
During the primary admission	148 (14·9%)	22 (12·4%)	121 (15·4%)	5 (20·0%)	0·570
As an emergency re-attender	12 (1·2%)	3 (1·7%)	8 (1·0%)	1 (4·0%)	-
At routine follow-up as an out-patient	37 (3·7%)	5 (2·8%)	31 (3·9%)	1 (4·0%)	-
Not applicable, no complications	787 (79·4%)	148 (83·1%)	622 (78·9%)	17 (68·0%)	-
Missing	7 (0·7%)	0 (0·0%)	6 (0·8%)	1 (4·0%)	-

*Patients born in hospital = 0. Percentages have been rounded to 1 decimal place and may not total 100·0%. ARM: Anorectal malfunction. HIC: High-income countries. IQR: Interquartile range. LIC: Low-income countries. MIC: Middle-income countries.

Supplementary Table 7: Characteristics, perioperative care, surgical interventions, and outcomes for patients with Hirschsprung's disease

Variable	Total (n=517)	HIC (n=107)	MIC (n=393)	LIC (n=17)	P value
**Patient Characteristics:**					
Median gestational age at birth (IQR), weeks	38 (2)	39 (2)	38 (2)	39 (4)	**<0·001**
Median age at presentation (IQR), hours	216 (1,740)	100 (466)	336 (2118)	291 (2784)	**<0·001**
Sex:					
Male	399 (77·2%)	81 (75·7%)	309 (78·6%)	9 (52·9%)	**0·044**
Female	118 (22·8%)	26 (24·3%)	84 (21·4%)	8 (47·1%)	
Median weight at presentation (IQR), kg	3·5 (2·1)	3·5 (0·9)	3·5 (2·6)	3·7 (3·8)	0·999
Does the patient have another anomaly in addition to the study condition?					
Yes: Cardiovascular	44 (8·5%)	13 (12·1%)	31 (7·9%)	0 (0·0%)	0·166
Yes: Respiratory	7 (1·4%)	1 (0·9%)	6 (1·5%)	0 (0·0%)	0·794
Yes: Gastrointestinal	19 (3·7%)	7 (6·5%)	12 (3·1%)	0 (0·0%)	0·168
Yes: Neurological	8 (1·5%)	4 (3·7%)	4 (1·0%)	0 (0·0%)	0·113
Yes: Genito-urinary	12 (2·3%)	4 (3·7%)	8 (2·0%)	0 (0·0%)	0·474
Yes: Musculoskeletal	7 (1·4%)	4 (3·7%)	3 (0·8%)	0 (0·0%)	**0·055**
Yes: Down syndrome	23 (4·4%)	9 (8·4%)	14 (3·6%)	0 (0·0%)	**0·065**
Yes: Beckwith Wiedemann syndrome	1 (0·2%)	0 (0·0%)	1 (0·3%)	0 (0·0%)	0·854
Yes: Cystic fibrosis	3 (0·6%)	0 (0·0%)	3 (0·8%)	0 (0·0%)	0·621
Yes: Chromosomal	11 (2·1%)	5 (4·7%)	6 (1·5%)	0 (0·0%)	0·112
Yes: Other	17 (3·3%)	5 (4·7%)	12 (3·1%)	0 (0·0%)	0·524
No	409 (79·1%)	77 (72·0%)	315 (80·2%)	17 (100·0%)	**0·018**
Median distance from patient's home to hospital (IQR), km*	50 (112)	39 (87)	51 (125)	17 (74)	0·193
Type of delivery:					
Vaginal (spontaneous)	267 (51·6%)	56 (52·3%)	202 (51·4%)	9 (52·9%)	**0·002**
Vaginal (induced)	35 (6·8%)	12 (11·2%)	19 (4·8%)	4 (23·5%)	-
Caesarean section (elective)	146 (28·2%)	22 (20·6%)	124 (31·6%)	0 (0·0%)	-
Caesarean section (urgent/non-elective)	57 (11·0%)	14 (13·1%)	41 (10·4%)	2 (11·8%)	-
Unknown	10 (1·9%)	3 (2·8%)	6 (1·5%)	1 (5·9%)	-
Missing	2 (0·4%)	0 (0·0%)	1 (0·3%)	1 (5·9%)	-
Was the patient septic on arrival to your hospital?					
Yes	132 (25·5%)	14 (13·1%)	115 (29·3%)	3 (17·6%)	**0·002**
No	385 (74·5%)	93 (86·9%)	278 (70·7%)	14 (82·4%)	-
Was the patient hypovolaemic on arrival to your hospital?					
Yes	98 (19·0%)	12 (11·2%)	85 (21·6%)	1 (5·9%)	**0·022**
No	418 (80·9%)	95 (88·8%)	308 (78·4%)	15 (88·2%)	-
Missing	1 (0·2%)	0 (0·0%)	0 (0·0%)	1 (5·9%)	-
Was the patient hypothermic on arrival to your hospital?					
Yes	40 (7·7%)	1 (0·9%)	39 (9·9%)	0 (0·0%)	**0·004**
No	476 (92·1%)	106 (99·1%)	354 (90·1%)	16 (94·1%)	-
Missing	1 (0·2%)	0 (0·0%)	0 (0·0%)	1 (5·9%)	-
American Society of Anaesthesiologists (ASA) Score at the time of primary intervention:					
1. Healthy person	113 (21·9%)	15 (14·0%)	90 (22·9%)	8 (47·1%)	**0·007**
2. Mild systemic disease	154 (29·8%)	42 (39·3%)	111 (28·2%)	1 (5·9%)	-
3. Severe systemic disease	122 (23·6%)	32 (29·9%)	87 (22·1%)	3 (17·6%)	-
4. Severe systemic disease that is a constant threat to life	20 (3·9%)	1 (0·9%)	19 (4·8%)	0 (0·0%)	-
5. A moribund patient who is not expected to survive without the operation	10 (1·9%)	2 (1·9%)	8 (2·0%)	0 (0·0%)	-
Not applicable - no intervention	98 (19·0%)	15 (14·0%)	78 (19·8%)	5 (29·4%)	-
What study condition does the patient have?					
Oesophageal atresia	0 (0·0%)	0 (0·0%)	0 (0·0%)	0 (0·0%)	-
Congenital diaphragmatic hernia	1 (0·2%)	0 (0·0%)	1 (0·3%)	0 (0·0%)	0·854
Intestinal atresia	3 (0·6%)	2 (1·9%)	1 (0·3%)	0 (0·0%)	0·142
Gastroschisis	1 (0·2%)	0 (0·0%)	1 (0·3%)	0 (0·0%)	0·854
Exomphalos/Omphalocele	0 (0·0%)	0 (0·0%)	0 (0·0%)	0 (0·0%)	-
Anorectal malformation	0 (0·0%)	0 (0·0%)	0 (0·0%)	0 (0·0%)	-
Hirschsprung's Disease	517 (100·0%)	107 (100·0%)	393 (100·0%)	17 (100·0%)	-
Time to first passage of meconium after birth					
Less than 24 hours	80 (15·5%)	20 (18·7%)	58 (14·8%)	2 (11·8%)	0·280
24-48 hours	148 (28·6%)	27 (25·2%)	118 (30·0%)	3 (17·6%)	-
Over 48 hours	187 (36·2%)	38 (35·5%)	145 (36·9%)	4 (23·5%)	-
Unknown	88 (17·0%)	20 (18·7%)	62 (15·8%)	6 (35·3%)	-
Missing	14 (2·7%)	2 (1·9%)	10 (2·5%)	2 (11·8%)	-
Features at presentation?					
Abdominal distension	460 (89·0%)	96 (89·7%)	350 (89·1%)	14 (82·4%)	0·663
Bilious vomiting	190 (36·8%)	51 (47·7%)	135 (34·4%)	4 (23·5%)	**0·021**
Non-bilious vomiting	103 (19·9%)	22 (20·6%)	79 (20·1%)	2 (11·8%)	0·689
Poor feeding	189 (36·6%)	50 (46·7%)	138 (35·1%)	1 (5·9%)	**0·002**

Suspected enterocolitis	96 (18·6%)	17 (15·9%)	74 (18·8%)	5 (29·4%)	0·397
Perforation	20 (3·9%)	2 (1·9%)	18 (4·6%)	0 (0·0%)	0·306
Other	56 (10·8%)	9 (8·4%)	43 (10·9%)	4 (23·5%)	0·174
Source of diagnosis of Hirschsprung's disease?					
Genetic	1 (0·2%)	1 (0·9%)	0 (0·0%)	0 (0·0%)	0·147
Mucosal biopsy	173 (33·5%)	74 (69·2%)	98 (24·9%)	1 (5·9%)	**<0·001**
Full thickness biopsy	175 (33·8%)	27 (25·2%)	148 (37·7%)	0 (0·0%)	**0·001**
Anorectal manometry	23 (4·4%)	3 (2·8%)	20 (5·1%)	0 (0·0%)	0·396
Barium enema	190 (36·8%)	28 (26·2%)	160 (40·7%)	2 (11·8%)	**0·002**
Not confirmed: suspected only	83 (16·1%)	5 (4·7%)	65 (16·5%)	13 (76·5%)	**<0·001**
Other	7 (1·4%)	1 (0·9%)	5 (1·3%)	1 (5·9%)	0·250
If on biopsy, what was the method of histology staining?					
Hemotoxilin and Eosin (H&E)	281 (80·7%)	62 (61·4%)	218 (88·6%)	1 (100·0%)	**<0·001**
Acetylcholinesterase	71 (20·4%)	53 (52·5%)	18 (7·3%)	0 (0·0%)	**<0·001**
Calretinin	104 (29·9%)	62 (61·4%)	42 (17·1%)	0 (0·0%)	**<0·001**
NADH-tetrazolium	6 (1·7%)	6 (5·9%)	0 (0·0%)	0 (0·0%)	**<0·001**
Other	4 (1·1%)	3 (3·0%)	1 (0·4%)	0 (0·0%)	**0·027**
Length of aganglionosis?					
Rectal	117 (22·6%)	21 (19·6%)	94 (23·9%)	2 (11·8%)	**<0·001**
Sigmoid	179 (34·6%)	35 (32·7%)	143 (36·4%)	1 (5·9%)	-
Descending colon	45 (8·7%)	8 (7·5%)	37 (9·4%)	0 (0·0%)	-
Transverse colon	16 (3·1%)	10 (9·3%)	6 (1·5%)	0 (0·0%)	-
Ascending colon	14 (2·7%)	1 (0·9%)	13 (3·3%)	0 (0·0%)	-
Small bowel	11 (2·1%)	2 (1·9%)	9 (2·3%)	0 (0·0%)	-
Unknown at present	135 (26·1%)	30 (28·0%)	91 (23·2%)	14 (82·4%)	-
**Care prior to presentation at the paediatric surgery centre:**					
Antenatal ultrasound undertaken?					
Yes: study condition diagnosed	5 (1·0%)	1 (0·9%)	4 (1·0%)	0 (0·0%)	**0·046**
Yes: problem identified but study condition not diagnosed	33 (6·4%)	11 (10·3%)	21 (5·3%)	1 (5·9%)	-
Yes: no problem identified	390 (75·4%)	87 (81·3%)	293 (74·6%)	10 (58·8%)	-
No	88 (17·0%)	8 (7·5%)	75 (19·1%)	5 (29·4%)	-
Missing	1 (0·2%)	0 (0·0%)	0 (0·0%)	1 (5·9%)	-
Median gestational age of study condition diagnosis if diagnosis was antenatal (IQR), weeks	28 (2)	28 (0)	27 (2)	-	0·936
Mode of transport to hospital:					
Ambulance	139 (26·9%)	52 (48·6%)	80 (20·4%)	7 (41·2%)	**<0·001**
Other transport provided by the health service	23 (4·4%)	14 (13·1%)	9 (2·3%)	0 (0·0%)	-
Patient's own transport	320 (61·9%)	31 (29·0%)	279 (71·0%)	10 (58·8%)	-
Born within the hospital	34 (6·6%)	10 (9·3%)	24 (6·1%)	0 (0·0%)	-
Missing	1 (0·2%)	0 (0·0%)	1 (0·3%)	0 (0·0%)	-
If outborn, where did the patient present from?					
Home	162 (33·6%)	26 (26·8%)	134 (36·4%)	2 (11·8%)	**0·006**
Community Clinic/General Practice	65 (13·5%)	5 (5·2%)	55 (14·9%)	5 (29·4%)	-
District Hospital	243 (50·4%)	64 (66·0%)	170 (46·2%)	9 (52·9%)	-
From another country	3 (0·6%)	1 (1·0%)	2 (0·5%)	0 (0·0%)	-
Unknown	9 (1·9%)	1 (1·0%)	7 (1·9%)	1 (5·9%)	-
**Perioperative care at the paediatric surgery centre:**					
If septic, were appropriate antibiotics administered?					
Yes within 1 hour of arrival	91 (68·9%)	12 (85·7%)	78 (67·8%)	1 (33·3%)	0·390
Yes: within the first day of arrival	38 (28·8%)	2 (14·3%)	34 (29·6%)	2 (66·7%)	-
No	3 (2·3%)	0 (0·0%)	3 (2·6%)	0 (0·0%)	-
If hypovolaemic, was an intravenous fluid bolus given?					
Yes within 1 hour of arrival	69 (70·4%)	4 (33·3%)	64 (75·3%)	1 (100·0%)	**<0·001**
Yes: within the first day of arrival	23 (23·5%)	3 (25·0%)	20 (23·5%)	0 (0·0%)	-
No	6 (6·1%)	5 (41·7%)	1 (1·2%)	0 (0·0%)	-
If hypovolaemic, how much intravenous fluid was given?					
10 - 20mls/kg	71 (77·2%)	2 (28·6%)	68 (81·0%)	1 (100·0%)	**0·006**
Above 20mls/kg	21 (22·8%)	5 (71·4%)	16 (19·0%)	0 (0·0%)	-
If hypothermic, was the patient warmed on arrival to your hospital to within a normal temperature range?					
Yes	36 (90·0%)	1 (100·0%)	35 (89·7%)	-	0·770
No	3 (7·5%)	0 (0·0%)	3 (7·7%)	-	-
Missing	1 (2·5%)	0 (0·0%)	1 (2·6%)	-	-
Did the patient receive central venous access?					
Yes: umbilical catheter	17 (3·3%)	3 (2·8%)	14 (3·6%)	0 (0·0%)	0·687
Yes: peripherally inserted central catheter (PICC)	81 (15·7%)	40 (37·4%)	40 (10·2%)	1 (5·9%)	**<0·001**
Yes: percutaneously inserted central line with ultrasound guidance	28 (5·4%)	10 (9·3%)	18 (4·6%)	0 (0·0%)	0·094
Yes: surgically placed central line (open insertion)	12 (2·3%)	3 (2·8%)	9 (2·3%)	0 (0·0%)	0·773
No	389 (75·2%)	55 (51·4%)	318 (80·9%)	16 (94·1%)	**<0·001**
Median total duration of antibiotics following primary intervention (IQR), days	6 (7)	3 (5)	7 (6)	3 (6)	**<0·001**
Did the patient receive a blood transfusion?					
Yes: not cross-matched	7 (1·4%)	0 (0·0%)	7 (1·8%)	0 (0·0%)	**<0·001**

Yes: cross-matched.	140 (27·1%)	18 (16·8%)	121 (30·8%)	1 (5·9%)	-
No: not required.	366 (70·8%)	88 (82·2%)	263 (66·9%)	15 (88·2%)	-
No: it was required but not available.	3 (0·6%)	0 (0·0%)	2 (0·5%)	1 (5·9%)	-
Missing	1 (0·2%)	1 (0·9%)	0 (0·0%)	0 (0·0%)	-
Did the patient require ventilation?					
Yes: and it was given	82 (15·9%)	28 (26·2%)	51 (13·0%)	3 (17·6%)	**<0·001**
Yes, but it was not available	2 (0·4%)	0 (0·0%)	1 (0·3%)	1 (5·9%)	-
No	433 (83·8%)	79 (73·8%)	341 (86·8%)	13 (76·5%)	-
Median time patient remained on ventilation if given (IQR), days	2 (3)	3 (4)	2 (3)	1 (1)	0·279
Median time to first enteral feed (post-primary intervention) (IQR), days	3 (3)	3 (4)	3 (3)	1 (0)	**0·023**
Median time to full enteral feeds (post-primary intervention) (IQR), days	4 (5)	5 (7)	4 (4)	3 (16)	**0·046**
Did the patient require parenteral nutrition?					
Yes: and it was given	167 (32·3%)	50 (46·7%)	117 (29·8%)	0 (0·0%)	**<0·001**
Yes: and it was sometimes available, but less than required	38 (7·4%)	0 (0·0%)	38 (9·7%)	0 (0·0%)	-
Yes: but it was not available	8 (1·5%)	0 (0·0%)	7 (1·8%)	1 (5·9%)	-
No	303 (58·6%)	56 (52·3%)	231 (58·8%)	16 (94·1%)	-
Missing	1 (0·2%)	1 (0·9%)	0 (0·0%)	0 (0·0%)	-
Median time patient received parenteral nutrition if received (IQR), days	6 (7·0)	7 (13·0)	5 (6·0)	-	**0·015**
**Surgical intervention:**					
Median time from arrival at your hospital to primary intervention (IQR), hours	45 (115)	29 (164)	48 (115)	16 (37)	0·186
Primary intervention:					
Conservative: regular rectal washouts/ enemas	144 (27·9%)	29 (27·1%)	113 (28·8%)	2 (11·8%)	**0·001**
Primary stoma (with or without pre-operative washouts or enemas prior to planned stoma placement)	142 (27·5%)	32 (29·9%)	105 (26·7%)	5 (29·4%)	-
Primary pull-through (Soave)	62 (12·0%)	13 (12·1%)	49 (12·5%)	0 (0·0%)	-
Failed conservative management followed by a stoma during the same hospital admission.	54 (10·4%)	7 (6·5%)	47 (12·0%)	0 (0·0%)	-
Primary pull-through (Swenson)	24 (4·6%)	9 (8·4%)	15 (3·8%)	0 (0·0%)	-
Conservative: including digital stimulation and laxatives	22 (4·3%)	2 (1·9%)	16 (4·1%)	4 (23·5%)	-
Primary pull-through (Other)	22 (4·3%)	4 (3·7%)	17 (4·3%)	1 (5·9%)	-
Conservative: no treatment	21 (4·1%)	7 (6·5%)	11 (2·8%)	3 (17·6%)	-
Transanal posterior anorectal myectomy	7 (1·4%)	0 (0·0%)	7 (1·8%)	0 (0·0%)	-
Palliative care	2 (0·4%)	0 (0·0%)	2 (0·5%)	0 (0·0%)	-
Primary pull-through (Duhamel)	1 (0·2%)	0 (0·0%)	1 (0·3%)	0 (0·0%)	-
Other	16 (3·1%)	4 (3·7%)	10 (2·5%)	2 (11·8%)	-
Was it laparoscopic assisted?					
Yes	55 (50·5%)	21 (80·8%)	34 (41·5%)	0 (0·0%)	**0·001**
No	54 (49·5%)	5 (19·2%)	48 (58·5%)	1 (100%)	-
If primary pull-through was undertaken, did the patient have a covering stoma?					
Yes	3 (2·8%)	2 (7·7%)	1 (1·2%)	0 (0·0%)	0·210
No	106 (97·2%)	24 (92·3%)	81 (98·8%)	1 (100·0%)	-
What type of anaesthesia was used for the primary intervention?					
General anaesthesia with endotracheal tube	321 (62·1%)	67 (62·6%)	247 (62·8%)	7 (41·2%)	**0·009**
General anaesthesia with laryngeal airway	10 (1·9%)	3 (2·8%)	7 (1·8%)	0 (0·0%)	-
Ketamine anaesthesia	3 (0·6%)	0 (0·0%)	2 (0·5%)	1 (5·9%)	-
Spinal/caudal anaesthesia	0 (0·0%)	0 (0·0%)	0 (0·0%)	0 (0·0%)	-
Local anaesthesia only	0 (0·0%)	0 (0·0%)	0 (0·0%)	0 (0·0%)	-
No anaesthesia, just analgesia	11 (2·1%)	6 (5·6%)	5 (1·3%)	0 (0·0%)	-
No anaesthesia, no analgesia	86 (16·6%)	14 (13·1%)	69 (17·6%)	3 (17·6%)	-
Not applicable: no surgery or primary intervention undertaken.	86 (16·6%)	17 (15·9%)	63 (16·0%)	6 (35·3%)	-
Who undertook the anaesthetic for the primary intervention?					
Anaesthetic doctor	330 (63·8%)	70 (65·4%)	253 (64·4%)	7 (41·2%)	0·130
Anaesthetic nurse	4 (0·8%)	0 (0·0%)	3 (0·8%)	1 (5·9%)	-
Medical officer	1 (0·2%)	0 (0·0%)	1 (0·3%)	0 (0·0%)	-
Surgeon	1 (0·2%)	1 (0·9%)	0 (0·0%)	0 (0·0%)	-
Other healthcare professional	2 (0·4%)	1 (0·9%)	1 (0·3%)	0 (0·0%)	-
No anaesthetic undertaken	179 (34·6%)	35 (32·7%)	135 (34·4%)	9 (52·9%)	-
Who undertook the primary intervention?					
Paediatric surgeon (or junior with paediatric surgeon assisting/in the room)	394 (76·2%)	86 (80·4%)	300 (76·3%)	8 (47·1%)	**0·007**
General surgeon (or junior with general surgeon assisting/in the room)	5 (1·0%)	2 (1·9%)	2 (0·5%)	1 (5·9%)	-
Junior doctor, medical officer or other (without a paediatric or general surgeon assisting/in the room)	37 (7·2%)	4 (3·7%)	32 (8·1%)	1 (5·9%)	-
Trainee surgeon (without a paediatric or general surgeon assisting or in the room)	11 (2·1%)	3 (2·8%)	8 (2·0%)	0 (0·0%)	-
Not applicable - no surgery or primary intervention undertaken.	70 (13·5%)	12 (11·2%)	51 (13·0%)	7 (41·2%)	-
Was a Surgical Safety Checklist used at the time of primary intervention?					
Yes	239 (46·2%)	71 (66·4%)	162 (41·2%)	6 (35·3%)	**<0·001**
No: but it was available	65 (12·6%)	1 (0·9%)	63 (16·0%)	1 (5·9%)	-
No: it was not available	42 (8·1%)	1 (0·9%)	40 (10·2%)	1 (5·9%)	-
Not applicable: a conservative primary intervention was undertaken	102 (19·7%)	24 (22·4%)	75 (19·1%)	3 (17·6%)	-
Not applicable: no surgery or primary intervention undertaken	69 (13·3%)	10 (9·3%)	53 (13·5%)	6 (35·3%)	-
What is the plan for future management?					

No further surgery planned	37 (7·2%)	5 (4·7%)	31 (7·9%)	1 (5·9%)	0·509
Anorectal pull-through at your hospital	299 (57·8%)	67 (62·6%)	224 (57·0%)	8 (47·1%)	0·382
Anorectal pull-through at a different hospital	8 (1·5%)	2 (1·9%)	5 (1·3%)	1 (5·9%)	0·307
Stoma closure	32 (6·2%)	7 (6·5%)	23 (5·9%)	2 (11·8%)	0·604
Other	27 (5·2%)	2 (1·9%)	23 (5·9%)	2 (11·8%)	0·121
Unknown	24 (4·6%)	7 (6·5%)	14 (3·6%)	3 (17·6%)	**0·015**
**Outcomes:**					
Did the patient survive to discharge (or 30-days if still an in-patient 30-days following primary intervention)?					
Yes	487 (94·2%)	105 (98·1%)	367 (93·4%)	15 (88·2%)	0·100
No	30 (5·8%)	2 (1·9%)	26 (6·6%)	2 (11·8%)	-
If the patient was discharged prior, were they still alive at 30-days following primary intervention?					
Yes	444 (91·2%)	100 (95·2%)	331 (90·2%)	13 (86·7%)	0·140
No	1 (0·2%)	0 (0·0%)	1 (0·3%)	0 (0·0%)	-
Not followed-up after discharge	24 (4·9%)	0 (0·0%)	22 (6·0%)	2 (13·3%)	-
Followed-up, but not until 30-days post primary intervention	18 (3·7%)	5 (4·8%)	13 (3·5%)	0 (0·0%)	-
Cause of mortality:					
Sepsis	10 (32·3%)	2 (100·0%)	7 (25·9%)	1 (50·0%)	0·870
Enterocolitis	7 (22·6%)	0 (0·0%)	7 (25·9%)	0 (0·0%)	-
Respiratory failure	4 (12·9%)	0 (0·0%)	4 (14·8%)	0 (0·0%)	-
Electrolyte disturbance	4 (12·9%)	0 (0·0%)	3 (11·1%)	1 (50·0%)	-
Malnutrition	3 (9·7%)	0 (0·0%)	3 (11·1%)	0 (0·0%)	-
Aspiration pneumonia	1 (3·2%)	0 (0·0%)	1 (3·7%)	0 (0·0%)	-
Cardiac failure	1 (3·2%)	0 (0·0%)	1 (3·7%)	0 (0·0%)	-
Ischaemic bowel	1 (3·2%)	0 (0·0%)	1 (3·7%)	0 (0·0%)	-
Median duration of hospital stays, (IQR) days	11 (10)	13 (12)	10 (9)	5 (7)	**0·001**
Did the patient have a surgical site infection?					
Yes	29 (5·6%)	5 (4·7%)	24 (6·1%)	0 (0·0%)	0·840
No	324 (62·7%)	68 (63·6%)	245 (62·3%)	11 (64·7%)	-
Not applicable, no surgical wound	164 (31·7%)	34 (31·8%)	124 (31·6%)	6 (35·3%)	-
Did the patient have a full thickness wound dehiscence?					
Yes	12 (2·3%)	0 (0·0%)	12 (3·1%)	0 (0·0%)	0·340
No	343 (66·3%)	76 (71·0%)	256 (65·1%)	11 (64·7%)	-
Not applicable, no surgical wound	162 (31·3%)	31 (29·0%)	125 (31·8%)	6 (35·3%)	-
Did the patient require a further unplanned intervention?					
Yes – percutaneous	5 (1·0%)	4 (3·7%)	1 (0·3%)	0 (0·0%)	**0·029**
Yes – surgical intervention	64 (12·4%)	13 (12·1%)	50 (12·7%)	1 (5·9%)	-
No	387 (74·9%)	76 (71·0%)	299 (76·1%)	12 (70·6%)	-
Not applicable – no primary intervention undertaken	61 (11·8%)	14 (13·1%)	43 (10·9%)	4 (23·5%)	-
If central line access used, did the patient acquire central line sepsis?					
Yes, diagnosed clinically	3 (2·3%)	0 (0·0%)	3 (3·9%)	0 (0·0%)	0·670
Yes, confirmed on microbiology	6 (4·6%)	3 (5·8%)	3 (3·9%)	0 (0·0%)	-
No	121 (93·1%)	49 (94·2%)	71 (92·2%)	1 (100·0%)	-
Did the patient have any condition specific complications within 30-days of primary intervention?					
Hirschsprung's associated enterocolitis (HAEC)	69 (13·3%)	13 (12·1%)	55 (14·0%)	1 (5·9%)	0·579
Electrolyte disturbance	47 (9·1%)	3 (2·8%)	41 (10·4%)	3 (17·6%)	**0·024**
Peri-stoma skin breakdown (or perianal if primary pull-through was undertaken without a covering stoma)	17 (3·3%)	2 (1·9%)	15 (3·8%)	0 (0·0%)	0·449
High stoma output (over 20mls/kg/day)	15 (2·9%)	6 (5·6%)	9 (2·3%)	0 (0·0%)	0·149
Stoma prolapse/ retraction/ herniation	14 (2·7%)	4 (3·7%)	9 (2·3%)	1 (5·9%)	0·511
Post-operative obstruction	14 (2·7%)	5 (4·7%)	9 (2·3%)	0 (0·0%)	0·316
Anastomotic leak (if primary pull-through was undertaken without a covering stoma)	3 (0·6%)	1 (0·9%)	2 (0·5%)	0 (0·0%)	0·832
Anal stenosis	2 (0·4%)	1 (0·9%)	1 (0·3%)	0 (0·0%)	0·583
Other	118 (22·8%)	8 (7·5%)	102 (26·0%)	8 (47·1%)	**<0·001**
Was the patient followed up at 30-days post primary surgery or intervention to a assess for complications?					
Yes: reviewed in person	307 (63·0%)	69 (65·7%)	234 (63·8%)	4 (26·7%)	**<0·001**
Yes: via telephone consultation	58 (11·9%)	4 (3·8%)	52 (14·2%)	2 (13·3%)	-
Yes: via other means	14 (2·9%)	3 (2·9%)	10 (2·7%)	1 (6·7%)	-
Yes: still an in-patient at 30-days	22 (4·5%)	11 (10·5%)	10 (2·7%)	1 (6·7%)	-
No: data is based on in-patient observations only	51 (10·5%)	10 (9·5%)	35 (9·5%)	6 (40·0%)	-
No: follow-up was done, but prior to 30-days	35 (7·2%)	8 (7·6%)	26 (7·1%)	1 (6·7%)	-
If the patient had a complication, when was it diagnosed?					
During the primary admission	62 (12·0%)	17 (15·9%)	45 (11·5%)	0 (0·0%)	**0·018**
As an emergency re-attender	27 (5·2%)	7 (6·5%)	17 (4·3%)	3 (17·6%)	-
At routine follow-up as an out-patient	25 (4·8%)	1 (0·9%)	24 (6·1%)	0 (0·0%)	-
Not applicable, no complications	400 (77·4%)	82 (76·6%)	305 (77·6%)	13 (76·5%)	-
Missing	3 (0·6%)	0 (0·0%)	2 (0·5%)	1 (5.9%)	-

*Patients born in hospital = 0. Percentages have been rounded to 1 decimal place and may not total 100·0%. HIC: High-income countries. IQR: Interquartile range. LIC: Low-income countries. MIC: Middle-income countries.

Supplementary Table 8: Patient follow-up

Variable	Total n, % (95% CI)	HIC n, % (95% CI)	MIC n, % (95% CI)	LIC n, % (95% CI)	P value*
**If the patient was discharged prior, were they still alive at 30-days post-intervention?†**	**n=2761**	**n=651**	**n=2057**	**n=53**	
Yes	2486, **90·3%** (89·1, 91·3)	606, **93.4%** (91.2, 95.1)	1848, **90.0%** (88.7, 91.2)	32, **60.3%** (46.4, 72.8)	**<0.001**
No	9, **0·3%** (0·2, 0·6)	0, **0.0%**	9, **0.4%** (0.2, 0.8)	0, **0.0%**	
Not followed-up after discharge	135, **4·9%** (4.2, 5.8)	13, **2.0%** (1.2%, 3.4%)	102, **5.0%** (4.1, 6.0)	20, **37.7%** (25.6, 51.7)	
Followed-up, but not until 30-days post primary intervention	124, 4·5% (3.8, 5·3)	30 4.6% (3.2%, 6.5%)	93, 4.5% (3.7, 5.5)	1, 1.9% (0.3, 12.7)	
Missing	7	2	5	0	
**If the patient survived to discharge, were they followed-up to 30-days post-intervention to assess for complications?**	**n=3179**	**n=846**	**n=2277**	**n=56**	
Yes: reviewed in person	1829, **57.8%** (56.0, 59.5)	467, **55.3%** (52.0, 58.7)	1350, **59.6%** (57.5, 61.6)	12, **21.4%** (12.4, 34.4)	**0·001**
Yes: via telephone consultation	341, **10.8%** (9.7, 11.8)	32, **3.8%** (2.5, 5.1)	304, **13.4%** (12.0, 14.8)	5, **8.9%** (3.7, 20.0)	
Yes: via other means	90, **2.8%** (2.3, 3.4)	15, **1.8%** (0.9, 2.7)	73, **3.2%** (2.5, 3.9)	2, **3.6%** (0.9, 13.6)	
Yes: still an in-patient at 30-days	418, **13.2%** (12.1, 14.4)	195, **23.1%** (20.3, 26.0)	220, **9.7%** (8.5, 10.9)	3, **5.4%** (1.7, 15.7)	
No: data is based on in-patient observations only	303, **9,6%** (8.5, 10.6)	74, **8.8%** (6.9, 10.7)	205, **9.0%** (7.9, 10.2)	24, **42.9%** (30.4, 56.3)	
No: follow-up was done, but prior to 30-days	186 **5,9%** (5.1, 6.7)	61, **7.2%** (5.5, 9.0)	115, **5.1%** (4.2, 6.0)	10, **17.9%** (9.8, 30.4)	
Missing	12	2	10	0	
**If the patient had a complication, when was it diagnosed?**	**n=3849**	**n=896**	**n=2860**	**n=93**	
During the primary admission	901, **23·5%** (22·2, 24·9)	203, **22·7%** (20·0, 25·5)	678, **23·8%** (22·3, 25·4)	20, **22·0%** (14·5, 31·8)	0·277
As an emergency re-attender	91, **2·4%** (1·9, 2·9)	22, **2·5%** (1·6, 3·7)	63, **2·2%** (1·7, 2·8)	6, **6·6%** (2·9, 14·1)	
At routine follow-up as an out-patient	101, **2·6%** (2·2, 3·2)	9, **1·0%** (0·5, 1·9)	90, **3·2%** (2·6, 3·9)	2, **2·2%** (0·5, 8·6)	
Not applicable, no complications	2738, **71·5%** (70·0, 72·9)	662, **73·9%** (70·9, 76·7)	2013, **70·8%** (69·1, 72·4)	63, **69·2%** (58·8, 78·0)	
Missing	18	0	16	2	

*Calculated using Chi-squared analysis and Fisher's exact as appropriate. †Includes those who survived to discharge (n=3179) minus those still an in-patient at 30-days (n=418). CI: Confidence interval. HIC: High-income countries. LIC: Low-income countries. MIC: Middle-income countries.

Supplementary Table 9: All-cause in hospital mortality rates for all patients and by condition

		N	Died	%	Lower CI*	Upper CI*	LIC p value†	MIC p value†	LIC, RR (95% CI)	MIC RR (95% CI)	HIC RR (95% CI)
All	LIC	93	37	39.8%	30%	50%	-	-	-	0.51 (0.40 to 0.66)	0.14 (0.10 to 0.20)
	MIC	2860	583	20.4%	19%	22%	<0.001	-	1.95 (1.50 to 2.53)	-	0.27 (0.21 to 0.36)
	HIC	896	50	5.6%	4%	7%	<0.001	<0.001	7.13 (4.94 to 10.30)	3.65 (2.76 to 4.83)	-
Gastroschisis	LIC	10	9	90.0%	87%	93%	-	-	-	0.35 (0.27 to 0.46)	0.02 (0.00 to 0.06)
	MIC	304	97	31.9%	28%	36%	<0.001	-	2.82 (2.17 to 3.67)	-	0.05 (0.01 to 0.18)
	HIC	139	2	1.4%	0%	5%	<0.001	<0.001	62.55 (15.56 to 251.47)	22.18 (5.55 to 88.65)	-
Congenital diaphragmatic hernia	LIC	1	0	0.0%	-	-	-	-	-	-	-
	MIC	299	115	38.5%	34%	43%	-	-	-	-	0.37 (0.24 to 0.56)
	HIC	148	21	14.2%	11%	17%	-	<0.001	-	2.71 (1.78 to 4.13)	-
Oesophageal atresia	LIC	7	6	85.7%	83%	89%	-	-	-	0.34 (0.24 to 0.48)	0.08 (0.04 to 0.16)
	MIC	412	121	29.4%	26%	33%	0.04	-	2.92 (2.08 to 4.09)	-	0.24 (0.13 to 0.45)
	HIC	141	10	7.1%	5%	9%	<0.001	<0.001	12.09 (6.19 to 23.61)	4.14 (2.24 to 7.67)	-
Intestinal atresia	LIC	20	12	60.0%	56%	64%	-	-	-	0.36 (0.24 to 0.53)	0.05 (0.02 to 0.14)
	MIC	509	109	21.4%	18%	24%	<0.001	-	2.80 (1.89 to 4.16)	-	0.15 (0.06 to 0.37)
	HIC	152	5	3.3%	1%	8%	<0.001	<0.001	18.24 (7.17 to 46.38)	6.51 (2.71 to 15.66)	-
Anorectal malformation	LIC	25	5	20.0%	7%	41%	-	-	-	0.60 (0.27 to 1.35)	0.08 (0.02 to 0.33)
	MIC	788	95	12.1%	10%	14%	0.219	-	1.66 (0.74 to 3.72)	-	0.14 (0.04 to 0.44)
	HIC	178	3	1.7%	0%	5%	0.001	<0.001	11.87 (3.02 to 46.64)	7.15 (2.29 to 22.32)	-
Hirschsprung's Disease‡	LIC	17	2	11.8%	1%	36%	-	-	-	0.56 (0.15 to 2.18)	0.16 (0.02 to 1.05)
	MIC	393	26	6.6%	4%	9%	0.326	-	1.78 (0.46 to 6.89)	-	0.28 (0.07 to 1.17)
	HIC	107	2	1.9%	0%	7%	0.09	0.06	6.29 (0.95 to 41.75)	3.54 (0.85 to 14.68)	-
Exomphalos/ Omphalocele‡	LIC	14	4	28.6%	8%	58%	-	-	-	0.71 (0.30 to 1.69)	0.60 (0.23 to 1.59)
	MIC	241	49	20.3%	16%	25%	0.498	-	1.41 (0.59 to 3.34)	-	0.84 (0.48 to 1.49)
	HIC	70	12	17.1%	13%	21%	0.454	0.554	1.67 (0.63 to 4.42)	1.19 (0.67 to 2.10)	-

*Wald confidence interval for a proportion formula used when n>5; Exact binomial confidence intervals used when n≤5. †Chi-squared test for n>5 and Fishers exact test for n≤5. ‡For Hirschsprung's disease there was no significant difference in mortality between LMIC (28/410, 6.8%) and HIC (2/107, 1.9%), p=0.0611. For exomphalos there was no significant difference between LMIC (53/255, 20.8%) and HIC (12/70, 17.1%), p=0.5000. CI: Confidence interval. HIC: High-income countries. LIC: Low-income countries. LMIC: Low- and middle-income countries. MIC: Middle-income countries. N: Total number of patients. RR: Risk ratio.

Supplementary Table 10: Univariable analysis of factors affecting mortality for all patients and by country income status (high-income or low- and middle-income)

	All (N=3849)							HIC (N=896)							LMIC (N=2953)						
	N	Died (%)		RR	95% CI		P-value	N	Died (%)		RR	95% CI		P-value	N	Died (%)		RR	95% CI		P-value
Sex:																					
Male	2231	375	(17%)	base	-	-	-	528	29	(5%)	base	-	-	-	1703	346	(20%)	base	-	-	-
Female	1596	284	(18%)	1·06	0·92	1·22	0·43	367	21	(6%)	1·04	0·60	1·80	0·88	1229	263	(21%)	1·05	0·91	1·21	0·48
Ambiguous	21	10	(48%)	2·83	1·79	4·48	**<0·001**	1*	0*	(0%)	-	-	-	-	20	10	(50%)	2·46	1·57	3·85	**<0·001**
Gestational age at birth:	3846	-	-	0·91	0·89	0·93	**<0·001**	896	-	-	0·81	0·76	0·88	**<0·001**	2913	-	-	0·91	0·89	0·93	**<0·001**
Age at presentation (in hours):	3838	-	-	1·00	1·00	1·00	**0·01**	893	-	-	0·98	0·94	1·02	0·34	2944	-	-	1·00	1·00	1·00	**0·01**
Weight at presentation (kg):	3840	-	-	0·53	0·47	0·59	**<0·001**	894	-	-	0·33	0·23	0·48	**<0·001**	2946	-	-	0·56	0·50	0·63	**<0·001**
Does the patient have another anomaly or another study condition?																					
No	2071	267	(13%)	base	-	-	-	448	8	(2%)	base	-	-	-	1623	259	(16%)	base	-	-	-
Yes	1778	403	(23%)	1·76	1·53	2·02	**<0·001**	448	42	(9%)	5·25	2·49	11·06	**<0·001**	1330	361	(27%)	1·70	0·14	0·18	**<0·001**
Antenatal diagnosis?																					
No: either no ultrasound or ultrasound with no problem identified	2503	419	(17%)	base	-	-	-	387	7	(2%)	base	-	-	-	2116	412	(19%)	base	-	-	-
Yes: study condition diagnosed or problem identify	1338	250	(19%)	1·12	0·97	1·29	0·13	506	43	(8%)	4·70	2·14	10·33	**<0·001**	832	207	(25%)	1·28	1·10	1·48	**0·001**
Distance from the patient's home to the study centre (km):	3844	-	-	1·00	1·00	1·00	**0·03**	896	-	-	0·99	0·99	1·00	0·19	2948	-	-	1·00	1·00	1·00	**0·01**
Born at the study centre?																					
No	2833	497	(18%)	base	-	-	-	504	14	(3%)	base	-	-	-	2329	483	(21%)	base	-	-	-
Yes	1011	173	(17%)	0·98	0·83	1·14	0·76	391	36	(9%)	3·31	1·81	6·06	**<0·001**	620	137	(22%)	1·07	0·90	1·26	0·46
Type of delivery:																					
Vaginal (spontaneous)	1767	333	(19%)	base	-	-	-	373	19	(5%)	base	-	-	-	1394	314	(23%)	base	-	-	-
Vaginal (induced)	194	16	(8%)	0·44	0·27	0·71	**<0·001**	97	2	(2%)	0·41	0·10	1·71	0·22	97	14	(14%)	0·64	0·39	1·05	**0·08**
Caesarean section (elective)	1022	150	(15%)	0·78	0·65	0·93	**0·01**	185	9	(5%)	0·96	0·44	2·07	0·91	837	141	(17%)	0·75	0·63	0·89	**0·001**
Caesarean section (urgent/non-elective)	825	169	(20%)	1·09	0·92	1·28	0·32	226	20	(9%)	1·74	0·95	3·18	0·07	599	149	(25%)	1·10	0·93	1·31	0·25
Was the patient septic on arrival to your hospital?																					
No	3187	428	(13%)	base	-	-	-	857	47	(5%)	base	-	-	-	2330	381	(16%)	base	-	-	-
Yes	659	242	(37%)	2·73	2·39	3·12	**<0·001**	38	3	(8%)	1·44	0·47	4·42	0·52	621	239	(38%)	2·35	2·06	2·69	**<0·001**
Was the patient hypothermic and/or hypovolaemic on arrival to your hospital?																					
No	3112	402	(13%)	base	-	-	-	797	34	(4%)	base	-	-	-	2315	368	(16%)	base	-	-	-
Yes	737	268	(36%)	2·82	2·47	3·21	**<0·001**	99	16	(16%)	3·79	2·17	6·61	**<0·001**	638	252	(39%)	2·48	2·17	2·84	**<0·001**
Did the patient receive an umbilical vein catheter?																					
No	3447	557	(16%)	base	-	-	-	743	27	(4%)	base	-	-	-	2704	530	(20%)	base	-	-	-
Yes	402	113	(28%)	1·74	1·46	2·07	**<0·001**	153	23	(15%)	4·14	2·44	7·02	**<0·001**	249	90	(36%)	1·84	1·54	2·21	**<0·001**
Did the patient receive a peripherally inserted central catheter (PICC)?																					
No	2729	552	(20%)	base	-	-	-	460	30	(7%)	base	-	-	-	2269	522	(23%)	base	-	-	-
Yes	1120	118	(11%)	0·52	0·43	0·63	**<0·001**	436	20	(5%)	0·70	0·41	1·22	0·21	684	98	(14%)	0·62	0·51	0·76	**<0·001**
Did the patient receive a percutaneously inserted direct central line?																					
No	3434	629	(18%)	base	-	-	-	709	40	(6%)	base	-	-	-	2725	589	(22%)	base	-	-	-
Yes	415	41	(10%)	0·54	0·40	0·73	**<0·001**	187	10	(5%)	0·95	0·48	1·86	0·88	228	31	(14%)	0·63	0·45	0·88	**0·01**
Did the patient receive a surgically placed direct central line?																					
No	3595	615	(17%)	base	-	-	-	869	49	(6%)	base	-	-	-	2726	566	(21%)	base	-	-	-
Yes	254	55	(22%)	1·27	0·99	1·62	0·06	27	1	(4%)	0·66	0·09	4·59	0·67	227	54	(24%)	1·15	0·90	1·46	0·28
Time from arrival at study centre to primary intervention (hours)†	3432	-	-	1·00	1·00	1·00	**0·05**	826	-	-	1·00	1·00	1·00	0·65	2606	-	-	1·00	1·00	1·00	**0·02**
American Society of Anesthesiologists (ASA) Score at the time of primary intervention:																					
1 or 2	1873	146	(8%)	base	-	-	-	375	4	(1%)	base	-	-	-	1498	142	(9%)	base	-	-	-
3	1046	183	(17%)	2·24	1·83	2·75	**<0·001**	316‡	9‡	(3%)	2·67	0·83	8·59	0·10	730	174	(24%)	2·51	2·05	3·08	**<0·001**
4 or 5	526	195	(37%)	4·76	3·93	5·76	**<0·001**	137‡	19‡	(14%)	13·00	4·50	37·56	**<0·001**	389	176	(45%)	4·77	3·94	5·78	**<0·001**
N/A: no intervention	395	144	(36%)	4·68	3·82	5·73	**<0·001**	62	18	(29%)	27·22	9·52	77·79	**<0·001**	333	126	(38%)	3·99	3·24	4·92	**<0·001**
What type of anaesthesia was used for the primary intervention?																					
General anaesthesia with endotracheal tube or laryngeal airway	3154	444	(14%)	base	-	-	-	772§	24§	(3%)	base	-	-	-	2382	420	(18%)	base	-	-	-
No general anaesthesia	301	46	(15%)	1·09	0·82	1·44	0·57	69§	6§	(9%)	2·80	1·18	6·61	**0·02**	232	40	(17%)	0·98	0·73	1·31	0·88

N/A: no surgery or primary intervention undertaken.	392	179	(46%)	3·24	2·83	3·72	**<0·001**	55§	20§	(36%)	11·62	6·86	19·68	**<0·001**	337	159	(47%)	2·68	2·32	3·09	**<0·001**
Who undertook the anaesthetic for the primary intervention?																					
Anaesthetic doctor	3115	433	(14%)	base	-	-	-	741	22	(3%)	base	-	-	-	2374	411	(17%)	base	-	-	-
Non-doctor anaesthetist	121	33	(27%)	1·96	1·45	2·66	**<0·001**	43	4	(9%)	3·13	1·13	8·69	**0·03**	78	29	(37%)	2·15	1·59	2·90	**<0·001**
No anaesthetic undertaken	610	202	(33%)	2·38	2·07	2·75	**<0·001**	112	24	(21%)	7·22	4·19	12·43	**<0·001**	498	178	(36%)	2·06	1·78	2·39	**<0·001**
Who undertook the primary intervention?																					
Paediatric surgeon (or junior with paediatric surgeon assisting/ in the room)	3345	475	(14%)	base	-	-	-	825§	32§	(4%)	base	-	-	-	2520	443	(18%)	base	-	-	-
Non-paediatric surgeon	140	20	(14%)	1·01	0·66	1·52	0·98	21§	0§	(0%)	-	-	-	-	199	20	(10%)	0·96	0·64	1·44	0·83
N/A: no surgery or primary intervention undertaken	361	174	(48%)	3·39	2·96	3·89	**<0·001**	49§	18§	(37%)	9·47	5·74	15·62	**<0·001**	312	156	(50%)	2·84	2·47	3·27	**<0·001**
Was a Surgical Safety Checklist used at the time of primary intervention?																					
Yes	2569	275	(11%)	base	-	-	-	747	25	(3%)	base	-	-	-	1822	250	(14%)	base	-	-	-
No	693	210	(30%)	2·83	2·41	3·32	**<0·001**	39	3	(8%)	2·30	0·72	7·29	0·16	654	207	(32%)	2·31	1·96	2·71	**<0·001**
N/A: a conservative primary intervention was undertaken / no surgery undertaken	584	184	(32%)	2·94	2·50	3·47	**<0·001**	109	22	(20%)	6·03	3·53	10·32	**<0·001**	475	162	(34%)	2·49	2·10	2·95	**<0·001**
Total duration of antibiotics following primary intervention (days):†	3802	-	-	0·96	0·94	0·97	**<0·001**	887	-	-	0·96	0·91	1·02	0·19	2915	-	-	0·94	0·93	0·96	**<0·001**
Did the patient receive a blood transfusion?																					
No: not required.	2448	276	(11%)	base	-	-	-	671	19	(3%)	base	-	-	-	1777	257	(14%)	base	-	-	-
Yes: cross-matched or not cross-matched.	1348	377	(28%)	2·48	2·16	2·85	**<0·001**	213	30	(14%)	4·97	2·86	8·66	**<0·001**	1135	347	(31%)	2·11	1·83	2·44	**<0·001**
No: it was required but not available.	47	17	(36%)	3·21	2·16	4·77	**<0·001**	9*	1*	(11%)	3·92	0·59	26·27	0·16	38	16	(42%)	2·91	1·97	4·30	**<0·001**
Did the patient require ventilation?																					
No	1755	179	(10%)	base	-	-	-	258	3	(1%)	base	-	-	-	1497	176	(12%)	base	-	-	-
Yes and it was given	2008	416	(21%)	2·03	1·73	2·39	**<0·001**	637	47	(7%)	6·35	1·99	20·23	**<0·001**	1371	369	(27%)	2·29	1·94	2·70	**<0·001**
Yes, but it was not available	85	75	(88%)	8·65	7·38	10·14	**<0·001**	1*	0*	(0%)	-	-	-	-	84	75	(89%)	7·59	6·49	8·89	**<0·001**
Did the patient require parenteral nutrition?																					
No	1476	278	(19%)	base	-	-	-	212	14	(7%)	base	-	-	-	1264	264	(21%)	base	-	-	-
Yes and it was given	2102	253	(12%)	0·64	0·55	0·75	**<0·001**	683	36	(5%)	0·80	0·44	1·45	0·46	1419	217	(15%)	0·73	0·62	0·86	**<0·001**
Yes and it was sometimes available, but less than required	143	52	(36%)	1·93	1·52	2·46	**<0·001**	0*	0*	-	-	-	-	-	143	52	(36%)	1·74	1·37	2·22	**<0·001**
Yes, but it was not available	125	86	(69%)	3·65	3·12	4·28	**<0·001**	0*	0*	-	-	-	-	-	125	86	(69%)	3·29	2·81	3·86	**<0·001**
Duration of hospital stay (days):**	3541	-	-	0·92	0·91	0·93	**<0·001**	757	-	-	0·89	0·86	0·93	**<0·001**	2784	-	-	0·93	0·91	0·94	**<0·001**
Did the patient have a surgical site infection?																					
No	2942	413	(14%)	base	-	-	-	728	29	(4%)	base	-	-	-	2214	384	(17%)	base	-	-	-
Yes	335	63	(19%)	1·34	1·05	1·70	**0·02**	76	2	(3%)	0·66	0·16	2·72	0·57	259	61	(24%)	1·36	1·07	1·72	**0·01**
N/A: no surgical wound	569	193	(34%)	2·42	2·09	2·79	**<0·001**	92	19	(21%)	5·18	3·03	8·87	**<0·001**	477	174	(36%)	2·10	1·81	2·44	**<0·001**
Did the patient have a full thickness wound dehiscence?																					
No	3178	445	(14%)	base	-	-	-	792§	30§	(4%)	base	-	-	-	2386	415	(17%)	base	-	-	-
Yes	102	24	(24%)	1·68	1·17	2·41	**<0·001**	12§	0§	(0%)	-	-	-	**-**	90	24	(27%)	1·53	1·08	2·18	**0·02**
N/A: no surgical wound	566	200	(35%)	2·52	2·19	2·91	**<0·001**	92§	20§	(22%)	5·74	3·40	9·68	**<0·001**	474	180	(38%)	2·18	1·89	2·52	**<0·001**
Did the patient require a further unplanned intervention?																					
No	3045	400	(13%)	base	-	-	-	728	23	(3%)	base	-	-	-	2317	377	(16%)	base	-	-	-
Yes - percutaneous or surgical intervention	453	98	(22%)	1·65	1·35	2·01	**<0·001**	117	10	(9%)	2·71	1·32	5·54	**0·01**	336	88	(26%)	1·61	1·32	1·97	**<0·001**
N/A: no primary intervention undertaken	347	171	(49%)	3·75	3·26	4·32	**<0·001**	51	17	(33%)	10·55	6·03	18·46	**<0·001**	296	154	(52%)	3·20	2·77	3·69	**<0·001**
Condition																					
Oesophageal atresia	560	137	(24%)	1·51	1·28	1·78	**<0·001**	141	10	(7%)	1·34	0·69	2·61	0·40	419	127	(30%)	1·56	1·32	1·84	**<0·001**
Congenital diaphragmatic hernia	448	136	(30%)	1·93	1·65	2·27	**<0·001**	148	21	(14%)	3·66	2·15	6·24	**<0·001**	300	115	(38%)	2·01	1·71	2·37	**<0·001**
Intestinal atresia	681	126	(19%)	1·08	0·90	1·28	0·40	152	5	(3%)	0·54	0·22	1·35	0·19	529	121	(23%)	1·11	0·93	1·32	0·24
Gastroschisis	453	108	(24%)	1·44	1·20	1·73	**<0·001**	139	2	(1%)	0·22	0·06	0·92	**0·04**	314	106	(34%)	1·73	1·46	2·06	**<0·001**
Exomphalos/ Omphalocele	325	65	(20%)	1·16	0·93	1·47	0·19	70	12	(17%)	3·73	2·04	6·80	**<0·001**	255	53	(21%)	0·99	0·78	1·27	0·93
Anorectal malformation	991	103	(10%)	0·52	0·43	0·64	**<0·001**	178	3	(2%)	0·26	0·08	0·82	**0·02**	813	100	(12%)	0·51	0·42	0·62	**<0·001**
Hirschsprung's Disease	517	30	(6%)	0·30	0·21	0·43	**<0·001**	107	2	(2%)	0·31	0·08	1·25	0·10	410	28	(7%)	0·29	0·20	0·42	**<0·001**
Country income status:																					
HIC	896	50	(6%)	base	-	-	**-**	-	-	-	-	-	-	-	-	-	-	-	-	-	-
MIC	2860	583	(20%)	3·65	2·76	4·83	**<0·001**	-	-	-	-	-	-	-	-	-	-	-	-	-	-
LIC	93	37	(40%)	7·13	4·94	10·30	**<0·001**	-	-	-	-	-	-	-	-	-	-	-	-	-	-

*Category/patients excluded from multivariable analysis due to no or low counts. †Sub-group therefore excluded from multivariable analysis. ‡Categories collapsed for multivariable analysis. §Variable excluded from multivariable analysis due to low or no counts. **Excluded from multivariable analysis due to missing data. CI: Confidence interval. HIC: High-income countries. LIC: Low-income countries. MIC: Middle-income countries. N/A: Not applicable. RR: Risk Ratio.

Supplementary Table 11: Univariable analysis of factors affecting mortality for patients with oesophageal atresia

Generic variables	N (total = 560)	Died, n	Died, %	RR	95% CI		P value
Sex:							
Male	314	75	24%	Reference	-	-	-
Female	242	61	25%	1·06	0·79	1·42	0·719
Ambiguous*	4	1	25%	1·05	0·19	5·79	0·958
Gestational age at birth:	557	-	-	0·91	0·87	0·94	**<0·001**
Age at presentation (in hours):	560	-	-	1·00	1·00	1·00	0·078
Weight at presentation (kg):	558	-	-	0·51	0·41	0·64	**<0·001**
Does the patient have another anomaly or another study condition?							
No	190	26	14%	Reference	-	-	-
Yes	370	111	30%	2·19	1·48	3·24	**<0·001**
Antenatal diagnosis?							
No: either no ultrasound or ultrasound with no problem identified	368	95	26%	Reference	-	-	-
Yes: study condition diagnosed or problem identified	191	42	22%	0·85	0·62	1·17	0·324
Distance from the patients home to the study centre (km):	560	-	-	1·00	1·00	1·00	0·333
Born at the study centre?							
No	426	105	25%	Reference	-	-	-
Yes	133	32	24%	0·98	0·69	1·38	0·891
Type of delivery:							
Vaginal (spontaneous)	222	56	25%	Reference	-	-	-
Vaginal (induced)	32	4	13%	0·50	0·19	1·28	0·145
Caesarean section (elective)	145	32	22%	0·87	0·60	1·28	0·492
Caesarean section (urgent/non-elective)	157	44	28%	1·11	0·79	0·1.56	0·542
Was the patient septic on arrival to your hospital?							
No	436	81	19%	Reference	-	-	-
Yes	124	56	45%	2·43	1·84	3·20	**<0·001**
Was the patient hypothermic and/or hypovolaemic on arrival to your hospital?							
No	449	82	18%	Reference			
Yes	111	55	50%	2·71	2·07	3·56	**<0·001**
Did the patient receive an umbilical vein catheter?							
No	486	122	25%	Reference	-	-	-
Yes	74	15	20%	0·81	0·50	1·30	0·380
Did the patient receive a peripherally inserted central catheter (PICC)?							
No	381	115	30%	Reference	-	-	-
Yes	179	22	12%	0·41	0·27	0·62	**<0·001**
Did the patient receive a percutaneously inserted central line?							
No	468	131	28%	Reference	-	-	**-**
Yes	92	6	7%	0·23	0·10	0·51	**<0·001**
Did the patient receive a surgically placed open central line?							
No	500	125	25%	Reference	-	-	-
Yes	60	12	20%	0·80	0·47	1·36	0·408
Time from arrival at study centre to primary intervention (hours) †	498	-	-	1·00	1·00	1·00	0·752
American Society of Anesthesiologists (ASA) Score at the time of primary intervention:							
1 or 2	223	26	12%	Reference	-	-	-
3	166	34	21%	1·76	1·10	2·81	**0·019**
4 or 5	131	51	39%	3·34	2·19	5·08	**<0·001**
N/A: no intervention‡	37	26	70%	6·03	3·97	9·15	**<0·001**
What type of anaesthesia was used for the primary intervention? §							
General anaesthesia	507	95	19%	Reference	-	-	**-**
No general anaesthesia	4	4	100%	5·34	4·45	6·40	**<0·001**
N/A: no surgery or primary intervention undertaken	49	38	78%	4·14	3·27	5·24	**<0·001**
Who undertook the anaesthetic for the primary intervention? §							
Anaesthetic doctor	506	94	19%	Reference	-	-	-
Non-doctor anaesthetist	4	4	100%	5·38	4·48	6·46	**<0·001**
No anaesthetic undertaken	50	39	78%	4·20	3·32	5·31	**<0·001**
Who undertook the primary intervention?							
Paediatric surgeon (or junior with paediatric surgeon assisting/ in the room)	508	100	20%	Reference	-	-	-
Non-paediatric surgeon	5	1	20%	1·02	0·17	5·93	0·986
N/A: no surgery or primary intervention undertaken ‡	47	36	77%	3·89	3·07	4·93	**<0·001**
Was a Surgical Safety Checklist used at the time of primary intervention?							
Yes	423	61	14%	Reference	-	-	**-**
No	90	43	48%	3·31	2·41	4·55	**<0·001**
N/A: a conservative primary intervention was undertaken / no surgery undertaken‡	47	33	70%	4·87	3·61	6·56	**<0·001**
Total duration of antibiotics following primary intervention (days): †	553	-	-	0·94	0·92	0·97	**<0·001**
Did the patient receive a blood transfusion?							
No: not required	295	51	17%	Reference	-	-	-
Yes: cross-matched OR not cross-matched	257	82	32%	1·85	1·36	2·51	**<0·001**
No: it was required but not available *	7	4	57%	3·31	1·66	6·58	**0·001**
Did the patient require ventilation?							
No	71	22	31%	Reference	-	-	-
Yes and it was given	475	102	22%	0·69	0·47	1·02	0·064
Yes, but it was not available	14	13	93%	3·00	2·05	4·37	**<0·001**
Did the patient require parenteral nutrition?							
No	125	59	47%	Reference	-	-	**-**

Yes and it was given	398	53	13%	0·28	0·21	0·39	**<0·001**
Yes and it was sometimes available, but less than required **	14	8	57%	1·21	0·74	1·98	0·445
Yes, but it was not available **	23	17	74%	1·57	1·15	2·17	**0·004**
Time to first feed (days): †	439	-	-	0·96	0·91	1·01	0·092
Time to full feeds (days): †	419	-	-	0·96	0·88	1·04	0·313
Duration of hospital stay (days): ***	503	-	-	0·91	0·89	0·92	**<0·001**
Did the patient have a surgical site infection?							
No	443	85	19%	Reference	-	-	-
Yes	63	15	24%	1·24	0·77	2·01	0·380
N/A: no surgical wound ‡	54	37	69%	3·57	2·74	4·65	**<0·001**
Did the patient have a full thickness wound dehiscence? §							
No	497	97	20%	Reference	-	-	-
Yes	7	2	29%	1·46	0·45	4·79	0·529
N/A: no surgical wound	56	38	68%	3·48	2·70	4·48	**<0·001**
Did the patient require a further unplanned intervention?							
No	443	89	20%	Reference	-	-	-
Yes - percutaneous or surgical intervention	71	14	20%	0·98	0·59	1·63	0·942
N/A: no primary intervention undertaken ‡	46	34	74%	3·68	2·85	4·74	**<0·001**
Country income status:							
HIC	141	10	7%	Reference	-	-	-
MIC **	412	121	29%	4·14	2·24	7·67	**<0·001**
LIC **	7	6	86%	12·09	6·18	23·62	**<0·001**
**Condition specific variables**							
Type of OA +/- TOF (Gross classification):							
Distal TOF with proximal OA (type C)	476	123	26%	Reference	-	-	-
Other type (types A,B,D or E)	84	14	17%	0·64	0·39	1·07	0·087
Long or short gap?							
Short	375	57	15%	Reference	-	-	-
Long	111	37	33%	2·19	1·54	3·13	**<0·001**
Unknown	74	43	58%	3·82	2·81	5·2	**<0·001**
Pneumonia at presentation?							
No	374	62	17%	reference	-	-	-
Yes	186	75	40%	2·43	1·83	3·24	**<0·001**
Did the patient have tracheomalacia?							
No	487	125	26%	reference	-	-	-
Yes	73	12	16%	0·64	0·37	1·10	0·105
Primary intervention:							
Primary oesophageal anastomosis	385	56	15%	reference	-	-	-
No primary oesophageal anastomosis	125	42	34%	2·31	1·63	3·26	**<0·001**
Palliative care‡	50	39	78%	5·36	4·04	7·12	**<0·001**
Surgical approach: †							
Open surgery: thoracotomy (muscle cutting or splitting), laparotomy, local incision, cervical approach or other	405	87	22%	reference	-	-	-
Minimally invasive approach	95	8	8%	0·39	0·20	0·78	**0·008**
Not applicable/no intervention/unknown	25	16	64%	2·98	2·10	4·22	**<0·001**
Condition specific complications:							
Pneumonia							
No	443	94	21%	reference	-	-	-
Yes	117	43	37%	1·73	1·29	2·33	**<0·001**
Mediastinitis							
No	523	118	23%	reference	-	-	-
Yes	37	19	51%	2·28	1·60	3·24	**<0·001**
Pneumothorax							
No	503	123	25%	reference	-	-	-
Yes	57	14	25%	1·00	0·62	1·62	0·99
Anastomotic leak							
No	497	120	24%	reference	-	-	-
Yes	63	17	27%	1·12	0·72	1·73	0·62
Anastomotic stricture							
No	533	136	26%	reference	-	-	-
Yes	27	1	4%	0·15	0·02	1·00	**0·05**

*Excluded from multivariable analysis due to low counts and inability to combine with another category. †Excluded from multivariable analysis as this variable is a sub-group. ‡N/A groups were not presented on the forest plots. §Excluded from the multivariable analysis due to low counts and inability to collapse categories further. **Category collapsed for the multivariable analysis due to low counts. ***Excluded from multivariable analysis due to missing data. CI: Confidence interval. HIC: High-income countries. LIC: Low-income countries. MIC: Middle-income countries. N/A: Not applicable. OA: Oesophageal Atresia. RR: Risk Ratio. TOF: Tracheo-oesophageal fistula.

Supplementary Table 12: Univariable analysis of factors affecting mortality for patients with congenital diaphragmatic hernia

Generic variables	N (total = 448)	Died, N	Died, %	RR	95% CI		P value
Sex:							
Male	262	80	31%	Reference	-	-	-
Female	186	56	30%	0·99	0·74	1·31	0·9
Gestational age at birth:	437	-	-	0·92	0·89	0·95	**<0·001**
Age at presentation (in hours):	446	-	-	0·99	0·99	1·00	0·09
Weight at presentation (kg):	448	-	-	0·59	0·51	0·68	**<0·001**
Does the patient have another anomaly or another study condition?							
No	202	38	19%	Reference	-	-	-
Yes	246	98	40%	2·12	1·53	2·93	**<0·001**
Antenatal diagnosis?							
No: either no ultrasound or ultrasound with no problem identified	235	59	25%	Reference	-	-	-
Yes: study condition diagnosed or problem identified	211	77	36%	1·45	1·09	1·93	**0·01**
Distance from the patients home to the study centre (km):	448	-	-	0·998	0·996	0·999	**0·01**
Born at the study centre?							
No	282	74	26%	Reference	-	-	**-**
Yes	165	62	38%	1·43	1·08	1·89	**0·01**
Type of delivery:							
Vaginal (spontaneous)	190	51	27%	Reference	-	-	-
Vaginal (induced)	33	6	18%	0·68	0·32	1·45	0·32
Caesarean section (elective)	123	42	34%	1·27	0·91	1·79	0·16
Caesarean section (urgent/non-elective)	92	37	40%	1·5	1·06	2·11	**0·02**
Was the patient septic on arrival to your hospital?							
No	372	97	26%	Reference	-	-	-
Yes	74	39	52%	2·02	1·53	2·66	**<0·001**
Was the patient hypothermic and/or hypovolaemic on arrival to your hospital?							
No	363	94	26%	Reference	-	-	**-**
Yes	85	42	49%	1·91	1·45	2·52	**<0·001**
Did the patient receive an umbilical vein catheter?							
No	293	74	25%	Reference	-	-	**-**
Yes	155	62	40%	1·58	1·20	2·09	**0·001**
Did the patient receive a peripherally inserted central catheter (PICC)?							
No	309	108	35%	Reference	-	-	**-**
Yes	139	28	20%	0·58	0·40	0·83	**0·003**
Did the patient receive a percutaneously inserted direct central line?							
No	371	123	33%	Reference	-	-	**-**
Yes	77	13	17%	0 .51	0·30	0 .85	**0·01**
Did the patient receive a surgically placed direct central line?							
No	419	129	31%	Reference	-	-	-
Yes	29	7	24%	0·78	0 .40	1·52	0·47
Time from arrival at study centre to primary intervention (hours)	364	-	-	0·997	0·994	0·999	**0·03**
American Society of Anesthesiologists (ASA) Score at the time of primary intervention:							
1 or 2	114	9	8%	Reference	-	-	-
3	148	34	23%	2·91	1·45	5·82	**0·003**
4 or 5	119	39	33%	4·15	2·11	8·18	**<0·001**
N/A: no intervention*	66	54	82%	10·36	5·48	19·61	**<0·001**
What type of anaesthesia was used for the primary intervention? †							
General anaesthesia with endotracheal tube or laryngeal airway	366	65	18%	Reference	-	-	-
No general anaesthesia	3	1	33%	1·88	0·37	9·46	0·45
N/A: no surgery or primary intervention undertaken.	79	70	89%	4·99	3·95	**6·30**	**<0·001**
Who undertook the anaesthetic for the primary intervention? †							
Anaesthetic doctor	367	65	18%	Reference	-	**-**	**-**
Non-doctor anaesthetist	1	0	0%	0·00003	3·72e-0	**0·0002**	**<0·001**
No anaesthetic undertaken	80	71	89%	5·01	3·96	**6·33**	**<0·001**
Who undertook the primary intervention? †							
Paediatric surgeon (or junior with paediatric surgeon assisting/ in the room)	368	66	18%	Reference	-	-	-
Non-paediatric surgeon	1	0	0%	0·00003	3·70e-06	0 .0002	**<0·001**
N/A: no surgery or primary intervention undertaken	79	70	89%	4·94	3·91	6·23	**<0·001**
Was a Surgical Safety Checklist used at the time of primary intervention?							
Yes	304	50	16%	Reference	-	-	**-**
No	63	18	29%	1·74	1·09	2·77	**0·02**
N/A: a conservative primary intervention or no surgery undertaken *	80	68	85%	5·17	3·95	6·77	**<0·001**
Total duration of antibiotics following primary intervention (days):	443	-	-	0·92	0·89	0·96	**<0·001**
Did the patient receive a blood transfusion?							
No: not required	253	72	28%	Reference	-	-	-
Yes: cross-matched OR not cross-matched	185	59	32%	1·12	0·84	1·49	0·44
No: it was required but not available ‡	9	5	56%	1·95	1·05	3·62	**0·03**
Did the patient require ventilation?							
No	58	5	9%	Reference	-	-	-
Yes and it was given	387	128	33%	3·84	1·64	8·98	**0·002**

Yes, but it was not available ‡	3	3	100%	11·6	5·01	26·84	**<0·001**
Did the patient require parenteral nutrition?							
No	146	57	39%	Reference	-	-	-
Yes and it was given §	286	73	26%	0·65	0·49	0·87	**0·003**
Yes and it was sometimes available, but less than required §	13	3	23%	0·59	0·21	1·63	0·31
Yes, but it was not available ‡	3	3	100%	2·56	2·09	3·14	**<0·001**
Time to first feed (days): **	315	-	-	1·04	0·99	1·1	0·12
Time to full feeds (days): **	315	-	-	1·07	1·01	1·14	**0·02**
Duration of hospital stay (days): ***	398	-	-	0·89	0·87	0·91	**<0·001**
Did the patient have a surgical site infection?							
No	346	66	19%	Reference	-	-	-
Yes	25	2	8%	0·42	0·11	1·62	0·21
N/A: no surgical wound *	77	68	88%	4·63	3·67	5·84	**<0·001**
Did the patient have a full thickness wound dehiscence? †							
No	366	65	18%	Reference	-	-	-
Yes	2	0	0%	5·95e-06	1·46e-06	0·00002	**<0·001**
N/A: no surgical wound	80	71	89%	0·17	3·95	6·31	**<0·001**
Did the patient require a further unplanned intervention?							
No	335	58	17%	Reference	-	-	-
Yes - percutaneous or surgical intervention	39	13	33%	1·93	1·17	3·18	**0·01**
N/A: no primary intervention undertaken *	74	65	88%	5·07	3·95	6·51	**<0·001**
Country income status:							
HIC	148	21	14%	Reference	-	-	-
MIC §	299	115	38%	2·71	1·78	4·13	**<0·001**
LIC §	1	0	0%	9·60e-06	1·30e-06	0·00007	**<0·001**
**Condition specific variables**							
Type of CDH							
Left posteriolateral (Bochdalek)	316	104	33%	reference	-	-	-
Right posteriolateral (Bochdalek)	69	18	26%	0·79	0·52	1·22	0·29
Other	63	14	22%	0·68	0·41	1·10	0·12
Liver position?							
Abdomen	284	69	24%	reference	-	-	-
Chest	124	38	31%	1·26	0·90	1·76	0·18
Unknown	40	29	73%	2·98	2·25	3·95	**<0·001**
Did the patient have pulmonary hypertension (at any stage)?							
No	152	12	8%	reference	-	-	-
Yes	259	109	42%	5·33	3·04	9·35	**<0·001**
Unknown or missing	36	15	42%	5·28	2·71	10·29	**<0·001**
Primary intervention:							
Primary repair (absorbable sutures)	43	6	20%	reference	-	-	-
Primary repair (non-absorbable sutures)	254	42	17%	1·19	0·54	2·62	0·68
Patch repair	66	16	24%	1·74	0·74	4·09	0·21
Palliation *	68	65	96%	6·85	3·25	14·4	**<0·001**
Surgical approach: **							
Laparotomy or thoracotomy	289	60	21%	reference	-	-	-
Laparoscopy or thoracoscopy	70	3	4%	0·20	0·07	0·64	**0·006**
N/A, no surgical intervention (n=88) or other approach (n=1)	89	73	82%	3·95	3·09	5·05	**<0·001**
Did the patient receive extracorporeal membrane oxygenation (ECMO)?							
No	420	126	30%	reference	-	-	-
Yes	28	10	36%	1·19	0·71	2·00	0·51

*N/A groups were not presented on the forest plots. †Excluded from the multivariable analysis due to low or no counts and inability to collapse categories. ‡Excluded from multivariable analysis due to low counts and inability to combine with another category. §Category collapsed for the multivariable analysis due to low counts. **Excluded from multivariable analysis as this variable is a sub-group. ***Excluded from multivariable analysis due to missing data. CDH: Congenital diaphragmatic hernia. CI: Confidence interval. HIC: High-income countries. LIC: Low-income countries. MIC: Middle-income countries. N/A: Not applicable. OA: Oesophageal Atresia. RR: Risk Ratio. TOF: Tracheo-oesophageal fistula.

Supplementary Table 13: Univariable analysis of factors affecting mortality for patients with intestinal atresia

Generic variables	N (total = 681)	Died, N	Died, %	RR	95% CI		P value
Sex:							
Male	336	59	18%	Reference	-	-	-
Female	343	66	19%	1·09	0 .79	1·50	0·57
Ambiguous *	2	1	50	2·84	0·69	11·61	0·14
Gestational age at birth:	676	-	-	0·95	0·90	0·99	**0·03**
Age at presentation (in hours):	680	-	-	0·99	0·99	1·00	0·15
Weight at presentation (kg):	680	-	-	0·52	0·43	0·63	**<0·001**
Does the patient have another anomaly or another study condition?			-				
No	385	74	19%	Reference	-	-	-
Yes	296	52	18%	0·91	0·66	1·25	0·58
Antenatal diagnosis?							
No: either no ultrasound or ultrasound with no problem identified	349	76	22%	Reference	-	-	-
Yes: study condition diagnosed or problem identified	330	50	15%	0·69	0·50	0·96	**0·02**
Distance from the patients home to the study centre (km):	681	-	-	1·00	0·99	1·00	0·52
Born at the study centre?							
No	465	105	23%	Reference	-	-	-
Yes	214	21	10%	0·43	0·27	0·67	**<0·001**
Type of delivery:							
Vaginal (spontaneous)	333	71	21%	Reference	-	-	-
Vaginal (induced)	20	1	5%	0·23	0·03	1·60	0·13
Caesarean section (elective)	145	29	20%	0·93	0·63	1·37	0·74
Caesarean section (urgent/non-elective)	181	25	14%	0·64	0·42	0·98	**0·04**
Was the patient septic on arrival to your hospital?							
No	540	70	13%	Reference	-	-	-
Yes	141	56	40%	3·06	2·27	4·13	**<0·001**
Was the patient hypothermic and/or hypovolaemic on arrival to your hospital?							
No	509	69	14%	Reference	-	-	-
Yes	172	57	33%	2·44	1·80	3·31	**<0·001**
Did the patient receive an umbilical vein catheter?							
No	612	113	18%	Reference	-	-	-
Yes	69	13	19%	1·02	0·60	1·71	0·93
Did the patient receive a peripherally inserted central catheter (PICC)?							
No	413	103	25%	Reference	-	-	-
Yes	268	23	9%	0·34	0·22	0·52	**<0·001**
Did the patient receive a percutaneously inserted direct central line?							
No	575	120	21%	Reference	-	-	-
Yes	106	6	6%	0·27	0·12	0·59	**0·001**
Did the patient receive a surgically placed direct central line?							
No	621	109	18%	Reference	-	-	-
Yes	60	17	28%	1·61	1·04	2·49	**0·03**
Time from arrival at study centre to primary intervention (hours) †	643		0%	1·001	1·0003	1·0026	**0·008**
American Society of Anesthesiologists (ASA) Score at the time of primary intervention:							
1 or 2	319	39	12%	Reference	-	-	-
3	239	41	17%	1·40	0·93	2·10	0·1
4 or 5	97	30	31%	2·52	1·66	3·84	**<0·001**
N/A: no intervention ‡	24	16	67%	5·45	3·62	8·20	**<0·001**
What type of anaesthesia was used for the primary intervention? §							
General anaesthesia with endotracheal tube or laryngeal airway	659	112	17%	Reference	-	-	-
No general anaesthesia	5	0	0%	4·51e-06	1·84e-06	0·00001	**<0·001**
N/A: no surgery or primary intervention undertaken.	17	14	82%	4·84	3·67	6·39	**<0·001**
Who undertook the anaesthetic for the primary intervention?							
Anaesthetic doctor	646	107	17%	Reference	-	-	-
Non-doctor anaesthetist	15	5	33%	2·01	0·96	4·20	0·06
No anaesthetic undertaken ‡	20	14	70%	4·22	3·02	5·90	**<0·001**
Who undertook the primary intervention?							
Paediatric surgeon (or junior with paediatric surgeon assisting/ in the room)	654	110	17%	Reference	-	-	-
Non-paediatric surgeon	11	3	27%	1·62	0·60	4·32	0·33
N/A: no surgery or primary intervention undertaken ‡	16	13	81%	4·83	3·61	6·46	**<0·001**
Was a Surgical Safety Checklist used at the time of primary intervention?							
Yes	530	59	11%	Reference	-	-	-
No	134	55	41%	3·68	2·69	5·05	**<0·001**
N/A: a conservative primary intervention was undertaken / no surgery undertaken	17	12	71%	6·34	4·29	9·36	**<0·001**
Total duration of antibiotics following primary intervention (days): †	671	-	-	0·97	0·95	1·002	0·07
Did the patient receive a blood transfusion?							
No: not required	322	32	10%	Reference	-	-	-
Yes: cross-matched OR not cross-matched	354	93	26%	2·64	1·82	3·83	**<0·001**
No: it was required but not available *	5	1	20%	2·01	0·33	11·99	0·44
Did the patient require ventilation?							
No	290	60	21%	Reference	-	-	-

Yes and it was given	370	49	13%	0·64	0·45	0·90	**0·01**
Yes, but it was not available	21	17	81%	3·91	2·87	5·31	**<0·001**
Did the patient require parenteral nutrition?							
No	106	30	28%	Reference	-	-	-
Yes and it was given	490	46	9%	0·33	0·22	0·49	**<0·001**
Yes and it was sometimes available, but less than required	37	19	51%	1·81	1·17	2·80	**0·007**
Yes, but it was not available	48	31	65%	2·28	1·57	3·29	**<0·001**
Time to first feed (days): †	575	-	-	0·99	0·94	1·04	0·72
Time to full feeds (days): †	544	-	-	1·00	0·94	1·07	0·86
Duration of hospital stay (days): **	603	-	-	0·91	0·89	0·93	**<0·001**
Did the patient have a surgical site infection?			-				
No	586	94	16%	Reference	-	-	-
Yes	71	17	24%	1·49	0·94	2·35	0·08
N/A: no surgical wound ‡	24	15	63%	3·89	2·71	5·59	**<0·001**
Did the patient have a full thickness wound dehiscence?							
No	639	106	17%	Reference	-	-	-
Yes	17	5	29%	1·77	0·83	3·78	0·13
N/A: no surgical wound ‡	25	15	60%	3·61	2·51	5·20	**<0·001**
Did the patient require a further unplanned intervention?							
No	552	81	15%	Reference	-	-	-
Yes - percutaneous or surgical intervention	107	29	27%	1·84	1·27	2·67	**0·001**
N/A: no primary intervention undertaken ‡	22	16	73%	4·95	3·57	6·86	**<0·001**
Country income status:							
HIC	152	5	3%	Reference	-	-	-
MIC ***	509	109	21%	6·51	2·70	15·67	**<0·001**
LIC ***	20	12	60%	18·24	7·16	46·41	**<0·001**
**Condition specific variables**							
Type of intestinal atresia?							
Duodenal	279	44	16%	reference	-	-	-
Jejuno-ileal	369	77	21%	1·32	0·95	1·85	0·10
Colonic	31	5	16%	1·02	0·44	2·39	0·96
Surgical approach: †							
Laparotomy	550	98	18%	reference	-	-	-
Laparoscopy or endoscopy	36	1	3%	0·16	0·02	1·09	0·06
Was the distal bowel flushed to check for patency?							
No	89	12	14%	reference	-	-	-
Yes	442	72	16%	1·21	0·68	2·13	0·51
N/A ‡	150	42	28%	2·08	1·16	3·73	**0·02**
Condition specific complications within 30-days of primary intervention:							
Anastomotic leak							
No	624	93	15%	reference	-	-	-
Yes	57	33	58%	3·88	2·91	5·19	**<0·001**
Anastomotic stenosis							
No	662	122	18%	reference	-	-	-
Yes	19	4	21%	1·14	0·47	2·77	0·77
Short-gut							
No	655	116	18%	reference	-	-	-
Yes	26	10	39%	2·17	1·30	3·63	**0·003**
Adhesive bowel obstruction							
No	658	123	19%	reference	-	-	-
Yes	23	3	13%	0·70	0·24	2·03	0·51

*Excluded from multivariable analysis due to low counts and inability to combine with another category. †Excluded from multivariable analysis as this variable is a sub-group. ‡N/A groups were not presented on the forest plots. §Excluded from the multivariable analysis due to low or no counts and inability to collapse categories. **Excluded from multivariable analysis due to missing data. ***Category collapsed for the multivariable analysis due to low counts. CI: Confidence interval. HIC: High-income countries. LIC: Low-income countries. MIC: Middle-income countries. N/A: Not applicable. RR: Risk Ratio.

Supplementary Table 14: Univariable analysis of factors affecting mortality for patients with gastroschisis

Generic variables	N (total =453)	Died, N	Died, %	RR	95% CI		P value
Sex:							
Male	232	55	24%	Reference	-	-	-
Female	221	53	24%	1·01	0·72	1·40	0·94
Gestational age at birth:	451	-	-	1·06	0·96	1·17	0·19
Age at presentation (in hours):	453	-	-	0·99	0·99	1·00	0·15
Weight at presentation (kg):	452	-	-	0·55	0·38	0·77	**0·001**
Does the patient have another anomaly or another study condition?							
No	325	77	24%	Reference	-	-	-
Yes	128	31	24%	1·02	0·71	1·47	0·90
Antenatal diagnosis?							
No: either no ultrasound or ultrasound with no problem identified	155	76	49%	Reference	-	-	-
Yes: study condition diagnosed or problem identified	298	32	11%	0·21	0·15	0·31	**<0·001**
Distance from the patients home to the study centre (km):	453	-	-	1·00	0·99	1·00	0·24
Born at the study centre?							
No	209	88	42%	Reference	-	-	-
Yes	244	20	8%	0·19	0·12	0·30	**<0·001**
Type of delivery:							
Vaginal (spontaneous)	176	68	39%	Reference	-	-	-
Vaginal (induced)	26	1	4%	0·09	0·01	0·68	**0·02**
Caesarean section (elective)	123	18	15%	0·37	0·23	0·60	**<0·001**
Caesarean section (urgent/non-elective)	128	21	16%	0·42	0·27	0·65	**<0·001**
Was the patient septic on arrival to your hospital?							
No	390	68	17%	Reference	-	-	-
Yes	62	40	65%	3·70	2·78	4·91	**<0·001**
Was the patient hypothermic and/or hypovolaemic on arrival to your hospital?							
No	324	47	15%	Reference	-	-	-
Yes	129	61	47%	3·25	2·36	4·49	**<0·001**
Did the patient receive an umbilical vein catheter? *							
No	439	103	23%	Reference	-	-	-
Yes	14	5	36%	1·52	0·73	3·13	0·25
Did the patient receive a peripherally inserted central catheter (PICC)?							
No	222	88	40%	Reference	-	-	-
Yes	231	20	9%	0·21	0·13	0·34	**<0·001**
Did the patient receive a percutaneously inserted direct central line?							
No	383	102	27%	Reference	-	-	-
Yes	70	6	9%	0·32	0·14	0·70	**0·005**
Did the patient receive a surgically placed direct central line?							
No	387	96	25%	Reference	-	-	-
Yes	66	12	18%	0·73	0·42	1·25	0·26
Time from arrival at study centre to primary intervention (hours) †	415		0%	1·004	1·0001	1·009	**0·04**
American Society of Anesthesiologists (ASA) Score at the time of primary intervention:							
1 or 2	188	28	15%	Reference	-	-	-
3	172	33	19%	1·28	0·81	2·03	0·28
4 or 5	63	36	57%	3·83	2·56	5·74	**0·002**
N/A: no intervention	30	11	37%	2·46	1·37	4·40	**<0·001**
What type of anaesthesia was used for the primary intervention?							
General anaesthesia with endotracheal tube or laryngeal airway	362	76	21%	Reference	-	-	-
No general anaesthesia	72	19	26%	1·25			0·30
N/A: no surgery or primary intervention undertaken ‡	19	13	68%	3·25			**<0·001**
Who undertook the anaesthetic for the primary intervention?							
Anaesthetic doctor	337	75	22%	Reference	-	-	-
Non-doctor anaesthetist	54	10	19%	0·83	0·45	1·50	0·54
No anaesthetic undertaken ‡	62	23	37%	1·66	1·13	2·44	**0·009**
Who undertook the primary intervention? *							
Paediatric surgeon (or junior with paediatric surgeon assisting/ in the room)	423	90	21%	Reference	-	-	-
Non-paediatric surgeon	17	6	35%	1·65	0·84	3·24	0·13
N/A: no surgery or primary intervention undertaken	13	12	92%	4·33	3·40	5·52	**<0·001**
Was a Surgical Safety Checklist used at the time of primary intervention?							
Yes	304	43	14%	Reference	-	-	-
No	92	47	51%	3·61	2·56	5·08	**<0·001**
N/A: a conservative primary intervention or no surgery undertaken ‡	57	18	32%	2·23	1·39	3·58	**0·001**
Total duration of antibiotics following primary intervention (days): †	447	-	-	0·91	0·88	0·95	**<0·001**
Did the patient receive a blood transfusion?							
No: not required	254	42	17%	Reference	-	-	-
Yes: cross-matched OR not cross-matched	190	62	33%	1·97	1·39	2·78	**<0·001**
No: it was required but not available §	9	4	44%	2·68	1·22	5·87	**0·01**
Did the patient require ventilation?							
No	82	30	36%	Reference	-	-	-
Yes and it was given	342	52	15%	0·41	0·28	0·60	**<0·001**
Yes, but it was not available	29	26	90%	2·45	1·79	3·34	**<0·001**

Did the patient require parenteral nutrition?							
No	55	41	75%	Reference	-	-	-
Yes and it was given	351	31	9%	0·11	0·08	0·17	**<0·001**
Yes and it was sometimes available, but less than required	21	15	71%	0·95	0·70	1·30	0·78
Yes, but it was not available	26	21	81%	1·08	0·84	1·38	0·51
Time to first feed (days): †	326	-	-	1·01	0·95	1·07	0·69
Time to full feeds (days): †	328	-	-	1·03	0·92	1·14	0·56
Duration of hospital stay (days): **	384	-	-	0·89	0·87	0·90	**<0·001**
Did the patient have a surgical site infection?							
No	368	73	20%	Reference	-	-	-
Yes	51	13	25%	1·28	0·76	2·14	0·33
N/A: no surgical wound ‡	34	22	65%	3·26	2·36	4·50	**<0·001**
Did the patient have a full thickness wound dehiscence?							
No	399	79	20%	Reference	-	-	-
Yes	21	6	29%	1·44	0·71	2·92	0·30
N/A: no surgical wound ‡	33	23	70%	3·52	2·60	4·75	**<0·001**
Did the patient require a further unplanned intervention?							
No	371	78	21%	Reference	-	-	-
Yes - percutaneous or surgical intervention	63	15	24%	1·13	0·69	1·83	0·61
N/A: no primary intervention undertaken ‡	19	15	79%	3·75	2·76	5·09	**<0·001**
Country income status:							
HIC	139	2	1%	Reference	-	-	-
MIC ***	304	97	32%	22·17	5·53	88·78	**<0·001**
LIC ***	10	9	90%	62·55	15·53	251·84	**<0·001**
**Condition specific variables**							
Type of Gastroschisis?							
Simple	349	72	21%	reference	-	-	-
Complex	104	36	35%	1·68	1·20	2·35	**0·002**
Primary intervention: *							
Primary closure in the operating room (OR)	166	31	19%	reference	-	-	-
Primary closure at the cotside (Bianchi technique)	32	1	3%	0·17	0·02	1·18	0·07
Staged closure using a preformed silo or Alexis Wound Retractor and Protector	146	29	20%	1·06	0·67	1·68	0·79
Staged closure using a surgical silo (including improvised silo)	83	34	41%	2·19	1·46	3·30	**<0·001**
No intervention undertaken	14	12	86%	4·60	3·13	6·73	**<0·001**
Method of defect closure? *							
Fascia and skin closed with sutures	277	31	11%	reference	-	-	-
Fascia left open, skin or cord sutured over the defect	50	19	38%	3·40	2·09	5·52	**<0·001**
Sutureless closure	66	6	9%	0·81	0·35	1·87	0·63
N/A	50	48	96%	8·58	6·12	12·01	**<0·001**
Did the neonate have any of these complications within 30-days of primary intervention?							
Ischemic bowel							
No	427	90	21%	reference	-	-	-
Yes	26	18	69%	3·28	2·40	4·50	**<0·001**
Abdominal compartment syndrome							
No	417	82	20%	reference	-	-	-
Yes	36	26	72%	3·67	2·77	4·86	**<0·001**
Necrotising enterocolitis							
No	435	104	24%	reference	-	-	-
Yes	18	4	22%	0·93	0·39	2·24	0·87

*Excluded from the multivariable analysis due to low or no counts and inability to collapse categories. †Excluded from multivariable analysis as this variable is a sub-group. ‡N/A groups were not presented on the forest plots. §Excluded from multivariable analysis due to low counts and inability to combine with another category. **Excluded from multivariable analysis due to missing data. ***Category collapsed for the multivariable analysis due to low counts. CI: Confidence interval. HIC: High-income countries. LIC: Low-income countries. MIC: Middle-income countries. N/A: Not applicable. RR: Risk Ratio.

Supplementary Table 15: Univariable analysis of factors affecting mortality for patients with exomphalos/omphalocele

Generic variables	N (total =325)	Died, N	Died, %	RR	95% CI		P value
Sex:							
Male	183	40	22%	Reference	-	-	-
Female	141	24	17%	0·7	0·49	1·22	0·28
Ambiguous *	1	1	100%	4·57	3·47	6·01	**<0·001**
Gestational age at birth:	321	-	-	0·84	0·79	0·88	**<0·001**
Age at presentation (in hours):	324	-	-	0·99	0·99	1·00	0·39
Weight at presentation (kg):	325	-	-	0·42	0·32	0·55	**<0·001**
Does the patient have another anomaly or another study condition?			-				
No	133	12	9%	Reference	-	-	-
Yes	192	53	28%	3·05	1·70	5·50	**<0·001**
Antenatal diagnosis?							
No: either no ultrasound or ultrasound with no problem identified	143	30	21%	Reference	-	-	-
Yes: study condition diagnosed or problem identified	182	35	19%	0·91	0·59	1·41	0·69
Distance from the patients home to the study centre (km):	325		0%	0·99	0·99	1·00	0·31
Born at the study centre?							
No	214	44	21%	Reference	-	-	-
Yes	111	21	19%	0·92	0·57	1·46	0·72
Type of delivery:							
Vaginal (spontaneous)	116	30	26%	Reference	-	-	-
Vaginal (induced)	12	2	17%	0·64	0·17	2·37	0·50
Caesarean section (elective)	130	10	8%	0·29	0·15	0·58	**<0·001**
Caesarean section (urgent/non-elective)	66	22	33%	1·28	0·81	2·04	0·28
Was the patient septic on arrival to your hospital?							
No	285	51	18%	Reference	-	-	-
Yes	40	14	35%	1·95	0·007	1·19	3·19
Was the patient hypothermic and/or hypovolaemic on arrival to your hospital?							
No	277	46	17%	Reference	-	-	-
Yes	48	19	40%	2·38	1·53	3·69	**<0·001**
Did the patient receive an umbilical vein catheter? †							
No	319	64	20%	Reference	-	-	-
Yes	6	1	17%	0·83	0·13	5·05	0·84
Did the patient receive a peripherally inserted central catheter (PICC)?							
No	222	47	21%	Reference	-	-	-
Yes	103	18	17%	0·82	0·44	0·50	1·34
Did the patient receive a percutaneously inserted direct central line?							
No	301	56	19%	Reference	-	-	-
Yes	24	9	38%	2·01	1·14	3·56	**0·01**
Did the patient receive a surgically placed direct central line?							
No	309	62	20%	Reference	-	-	-
Yes	16	3	19%	0·93	0·32	2·65	0·89
Time from arrival at study centre to primary intervention (hours) ‡	272	-	-	0·99	0·99	1·00	0·58
American Society of Anesthesiologists (ASA) Score at the time of primary intervention:							
1 or 2	173	12	7%	Reference	-	-	-
3	72	17	24%	3·40	1·71	6·76	**<0·001**
4 or 5	23	15	65%	9·40	5·04	17·53	**<0·001**
N/A: no intervention §	55	20	36%	5·24	2·73	10·03	**<0·001**
What type of anaesthesia was used for the primary intervention?							
General anaesthesia with endotracheal tube or laryngeal airway	200	29	15%	Reference	-	-	-
No general anaesthesia	59	15	25%	1·75	1·00	3·04	**0·04**
N/A: no surgery or primary intervention undertaken §	65	21	32%	2·22	1·36	3·62	**0·001**
Who undertook the anaesthetic for the primary intervention? †							
Anaesthetic doctor	193	27	14%	Reference	-	-	-
Non-doctor anaesthetist	14	6	43%	3·06	1·52	6·16	**0·002**
No anaesthetic undertaken	117	32	27%	1·95	1·23	3·09	**0·004**
Who undertook the primary intervention? †							
Paediatric surgeon (or junior with paediatric surgeon assisting/ in the room)	238	42	18%	Reference	-	-	-
Non-paediatric surgeon	22	4	18%	1·03	0·40	2·60	0·95
N/A: no surgery or primary intervention undertaken	64	19	30%	1·68	1·05	2·68	**0·02**
Was a surgical safety checklist used at the time of primary intervention?							
Yes	171	22	13%	Reference	-	-	-
No	41	13	31%	2·46	1·35	4·47	**0·003**
N/A: a conservative primary intervention or no surgery undertaken §	112	30	27%	2·08	1·26	3·42	**0·004**
Total duration of antibiotics following primary intervention (days): ‡	320	-		0·98	0·95	1·02	0·49
Did the patient receive a blood transfusion?							
No: not required	233	32	14%	Reference	-	-	-
Yes: cross-matched OR not cross-matched	87	33	38%	2·76	1·81	4·20	**<0·001**
No: it was required but not available *	4	0	0%	4·60e-06	1·64e-06	0·00001	**<0·001**
Did the patient require ventilation?							
No	175	20	11%	Reference	-	-	-
Yes and it was given	144	39	27%	2·36	1·44	3·87	**0·001**

Yes, but it was not available *	6	6	100%	8·75	5·78	13·22	**<0·001**
Did the patient require parenteral nutrition?							
No	158	34	22%	Reference	-	-	-
Yes and it was given	154	27	18%	0·81	0·51	1·28	0·37
Yes and it was sometimes available, but less than required	8	1	13%	0·58	0·09	3·73	0·56
Yes, but it was not available *	5	3	60%	2·78	1·28	6·06	**0·01**
Time to first feed (days): ‡	225	-	-	1·01	0·94	1·09	0·67
Time to full feeds (days): ‡	246	-	-	0·99	0·91	1·07	0·90
Duration of hospital stay (days): **	301	-	-	0·93	0·91	0·96	**<0·001**
Did the patient have a surgical site infection?			-				
No	191	30	16%	Reference	-	-	-
Yes	32	6	19%	1·19	0·53	2·64	0·66
N/A: no surgical wound §	101	29	29%	1·82	1·16	2·86	**0·009**
Did the patient have a full thickness wound dehiscence? †							
No	214	32	15%	Reference	-	-	-
Yes	11	4	36%	2·43	1·04	5·66	**0·03**
N/A: no surgical wound	99	29	29%	1·95	1·25	3·05	**0·003**
Did the patient require a further unplanned intervention?							
No	243	33	14%	Reference	-	-	-
Yes - percutaneous or surgical intervention	31	11	35%	2·61			**0·001**
N/A: no primary intervention undertaken §	50	21	42%	3·09			**<0·001**
Country income status:							
HIC	70	12	17%	Reference	-	-	-
MIC ***	241	49	20%	1·18	0·66	2·10	0·30
LIC ***	14	4	29%	1·66	0·62	4·42	**<0·001**
**Condition specific variables**							
Type of Exomphalos?							
Minor	175	27	22%	reference	-	-	-
Major	148	38	26%	1·66	1·07	2·59	**0·02**
Hypoglycaemic on arrival?							
No	242	38	16%	reference	-	-	-
Yes	39	11	28%	1·80	1·01	3·21	**0·05**
Blood glucose not measured	43	16	37%	2·37	1·46	3·85	**0·001**
Did the patient have a ruptured sac?							
No	288	48	17%	reference	-	-	-
Yes	34	17	50%	3·00	1·96	4·59	**<0·001**
Primary intervention:							
Primary operative closure	164	21	13%	reference	-	-	-
Staged closure	32	9	28%	2·20	1·11	4·35	**0·024**
Conservative management	120	33	28%	2·15	1·31	3·52	**0·002**

*Excluded from multivariable analysis due to low counts and inability to combine with another category. †Excluded from the multivariable analysis due to low or no counts and inability to collapse categories. ‡Excluded from multivariable analysis as this variable is a sub-group.§ N/A groups were not presented on the forest plots. **Excluded from multivariable analysis due to missing data. ***Category collapsed for the multivariable analysis due to low counts. CI: Confidence interval. HIC: High-income countries. LIC: Low-income countries. MIC: Middle-income countries. N/A: Not applicable. RR: Risk Ratio.

Supplementary Table 16: Univariable analysis of factors affecting mortality for patients with anorectal malformation

Generic variables	N (total = 991)	Died, N	Died, %	RR	95% CI		P value
Sex:							
Male	575	60	10%	Reference	-	-	-
Female	398	33	8%	0·79	0·52	1·19	0·26
Ambiguous	17	9	52%	5·07	3·05	8·43	**<0·001**
Gestational age at birth:	977	-	-	0·84	0·80	0·88	**<0·001**
Age at presentation (in hours):	989	-	-	0·99	0·99	0·99	**0·002**
Weight at presentation (kg):	988	-	-	0 .43	0·34	0·56	**<0·001**
Does the patient have another anomaly or another study condition?							
No	430	19	4%	Reference	-	-	-
Yes	561	84	15%	3·38	2·09	5·48	**<0·001**
Antenatal diagnosis?							
No: either no ultrasound or ultrasound with no problem identified	828	74	9%	Reference	-	-	**-**
Yes: study condition diagnosed or problem identified	161	28	17%	1·94	1·30	2·90	**0·001**
Distance from the patients home to the study centre (km):	989		0%	0·99	0 .99	1·00	0·35
Born at the study centre?							
No	835	81	10%	Reference	-	-	-
Yes	156	22	14%	1·45	0·93	2·25	0·09
Type of delivery:							
Vaginal (spontaneous)	520	49	9%	Reference	-	-	-
Vaginal (induced)	42	3	7%	0·75	0·24	2·33	0·62
Caesarean section (elective)	240	22	9%	0·97	0·60	1·57	0·91
Caesarean section (urgent/non-elective)	177	29	16%	1·73	1·13	2·66	**0·01**
Was the patient septic on arrival to your hospital?							
No	879	72	8%	Reference	-	-	**-**
Yes	112	31	28%	3·37	2·32	4·90	**<0·001**
Was the patient hypothermic and/or hypovolaemic on arrival to your hospital?							
No	883	72	8%	Reference	-	-	**-**
Yes	108	31	29%	3·52	2·42	5·10	**<0·001**
Did the patient receive an umbilical vein catheter?							
No	913	88	10%	Reference	-	-	**-**
Yes	78	15	19%	1·99	1·21	3·27	**0·006**
Did the patient receive a peripherally inserted central catheter (PICC)?							
No	818	88	11%	Reference	-	-	-
Yes	173	15	9%	0·80	0·47	1·35	0·41
Did the patient receive a percutaneously inserted direct central line?							
No	941	97	10%	Reference	-	-	-
Yes	50	6	12%	1·16	0·53	2·52	0·70
Did the patient receive a surgically placed direct central line?							
No	965	96	10%	Reference	-	-	-
Yes	26	7	27%	2·70	1·39	5·24	**0·003**
Time from arrival at study centre to primary intervention (hours) *	900	-	-	0·99	0. 99	1·00	0·16
American Society of Anesthesiologists (ASA) Score at the time of primary intervention:							
1 or 2	633	31	5%	Reference	-	-	-
3	172	25	15%	2·96	1·80	4·89	**<0·001**
4 or 5	90	25	28%	5·67	3·51	9·15	**<0·001**
N/A: no intervention †	93	21	23%	4·61	2·76	7·67	**<0·001**
What type of anaesthesia was used for the primary intervention?							
General anaesthesia with endotracheal tube or laryngeal airway	841	69	8%	Reference	-	-	-
No general anaesthesia	63	9	14%	1·74	0·91	3·32	0·092
N/A: no surgery or primary intervention undertaken †	86	24	28%	3·40	2·26	5·11	**<0·001**
Who undertook the anaesthetic for the primary intervention?							
Anaesthetic doctor	847	68	8%	Reference	-	-	**-**
Non-doctor anaesthetist	28	9	32%	4·00	2·23	7·18	**<0·001**
No anaesthetic undertaken †	114	24	21%	2·62	1·71	4·00	**<0·001**
Who undertook the primary intervention?							
Paediatric surgeon (or junior with paediatric surgeon assisting/ in the room)	877	75	9%	Reference	-	-	-
Non-paediatric surgeon	32	3	9%	1·09	0·36	3·29	0·87
N/A: no surgery or primary intervention undertaken †	80	24	30%	3·50	2·35	5·22	**<0·001**
Was a Surgical Safety Checklist used at the time of primary intervention?							
Yes	702	48	7%	Reference	-	-	**-**
No	174	28	16%	2·35	1·52	3·63	**<0·001**
N/A: a conservative primary intervention or no surgery undertaken †	114	26	23%	3·33	2·16	5·15	**<0·001**
Total duration of antibiotics following primary intervention (days): *	982	-	-	0·98	0·94	1·02	0·43
Did the patient receive a blood transfusion?							
No: not required	783	48	6%	Reference	-	-	-
Yes: cross-matched OR not cross-matched	194	52	27%	4·37	3·05	6·26	**<0·001**
No: it was required but not available	12	3	25%	4·07	1·47	11·28	**0·007**
Did the patient require ventilation?							
No	657	24	4%	Reference	-	-	-

Yes and it was given	321	68	21%	5·79	3·71	9·05	**<0·001**
Yes, but it was not available	12	11	92%	25·09	16·35	38·51	**<0·001**
Did the patient require parenteral nutrition?							
No	605	53	9%	Reference	-	-	-
Yes and it was given	358	37	10%	1·17	0·79	1·75	0·41
Yes and it was sometimes available, but less than required	12	4	33%	3·80	1·64	8·82	**0·002**
Yes, but it was not available	14	8	57%	6·52	3·87	10·99	**<0·001**
Time to first feed (days): *	833	-	-	1·07	1·01	1·13	**0·01**
Time to full feeds (days): *	876	-	-	1·01	0·95	1·07	0·63
Duration of hospital stay (days): ‡	960	-	-	0·95	0·91	0·99	**0·02**
Did the patient have a surgical site infection?			-				
No	775	66	9%	Reference	-	-	-
Yes	86	12	14%	1·63	0·92	2·90	0·09
N/A: no surgical wound †	128	24	19%	2·20	1·43	3·37	**<0·001**
Did the patient have a full thickness wound dehiscence?							
No	829	72	9%	Reference	-	-	-
Yes	38	4	11%	1·21	0·46	3·14	0·69
N/A: no surgical wound †	122	26	21%	2·45	1·63	3·68	**<0·001**
Did the patient require a further unplanned intervention?							
No	805	60	7%	Reference	-	-	-
Yes - percutaneous or surgical intervention	100	20	20%	2·68	1·69	4·25	**<0·001**
N/A: no primary intervention undertaken †	83	22	27%	3·55	2·30	5·48	**<0·001**
Country income status:							
HIC	178	3	2%	Reference	-	-	-
MIC §	788	95	12%	7·15	2·29	22·32	
LIC §	25	5	20%	11·86	3·01	46·67	
**Condition specific variables**							
Type of anorectal malformation (Krickenbeck classification)							
Low	327	16	5%	reference	-	-	-
High	592	73	12%	2·52	1·49	4·26	**0·001**
Rare variant or other	71	13	18%	3·74	1·88	7·43	**<0·001**
Did the neonate have pre-operative bowel perforation?							
No	951	89	9%	reference	-	-	-
Yes	37	12	32%	3·47	2·09	5·75	**<0·001**
Primary intervention:							
Fistula dilation and/or washout via fistula, no surgery (yes)	94	5	5%	0·49	0·20	1·17	0·11
Divided sigmoid colostomy (yes)	306	27	9%	0·80	0·52	1·21	0·28
Other colostomy or stoma (yes)	261	40	15%	1·78	1·23	2·57	**0·002**
Anoplasty/anorectoplasty (yes)	223	6	3%	0·21	0·09	0·48	**<0·001**
Anorectal pull-through (yes)	94	1	1%	0·09	0·01	0·66	**0·018**
Palliative care/no intervention (yes) †	46	23	50%	5·91	4·13	8·44	**<0·001**
Electrolyte disturbance							
No	751	41	6%	reference	-	-	-
Yes	84	30	36%	6·54	4·33	9·89	**<0·001**
Not applicable †	156	32	21%	5·76	2·45	5·77	**<0·001**

*Excluded from multivariable analysis as this variable is a sub-group. †N/A groups were not presented on the forest plots. ‡Excluded from multivariable analysis due to missing data. §Category collapsed for the multivariable analysis due to low counts. CI: Confidence interval. HIC: High-income countries. LIC: Low-income countries. MIC: Middle-income countries. N/A: Not applicable. RR: Risk Ratio.

Supplementary Table 17: Univariable analysis of factors affecting mortality for patients with Hirschsprung's disease

Generic variables	N (total = 517)	Died, N	Died, %	RR	95% CI		P value
Sex:							
Male	399	23	6%	Reference	-	-	-
Female	118	7	6%	1·02	0·45	2·34	0·94
Gestational age at birth:	505	-	-	0·91	0·76	1·09	0·34
Age at presentation (in hours):	512	-	-	0·99	0·99	1·00	0·25
Weight at presentation (kg):	516	-	-	0·84	0·65	1·08	0·17
Does the patient have another anomaly or another study condition?							
No	406	21	5%	Reference	-	-	-
Yes	111	9	8%	1·56	0·73	3·32	0·24
Antenatal diagnosis? *							
No: either no ultrasound or ultrasound with no problem identified	478	27	6%	Reference	-	-	-
Yes: study condition diagnosed or problem identified	38	3	8%	1·39	0·44	4·40	0·56
Distance from the patients home to the study centre (km): †	514	-	-	0·99	0·99	1·00	0·49
Born at the study centre?							
No	482	26	5%	Reference	-	-	-
Yes	34	4	12%	2·18	0·80	5·89	0·12
Type of delivery: *							
Vaginal (spontaneous)	267	23	9%	Reference	-	-	-
Vaginal (induced)	35	0	0%	-	-	-	-
Caesarean section (elective)	146	5	3%	0·39	0·15	1·02	0·06
Caesarean section (urgent/non-elective)	57	2	4%	0·40	0·09	1·68	0·21
Was the patient septic on arrival to your hospital? ‡							
No	385	12	3%	Reference	-	-	-
Yes	132	18	14%	4·37	2·16	8·84	**<0·001**
Was the patient hypothermic and/or hypovolaemic on arrival to your hospital?							
No	414	16	4%	Reference	-	-	-
Yes	103	14	14%	3·51	1·77	6·97	**<0·001**
Did the patient receive an umbilical vein catheter? *							
No	500	26	5%	Reference	-	-	-
Yes	17	4	24%	4·52	1·77	11·53	**0·002**
Did the patient receive a peripherally inserted central catheter (PICC)?							
No	436	28	6%	Reference	-	-	-
Yes	81	2	2%	0·38	0·09	1·58	0·18
Did the patient receive a percutaneously inserted direct central line? *							
No	489	27	5%	Reference	-	-	-
Yes	28	3	11%	1·94	0·62	6·01	0·25
Did the patient receive a surgically placed direct central line? *							
No	505	29	6%	Reference	-	-	-
Yes	12	1	8%	1·45	0·21	9·81	0·70
Time from arrival at study centre to primary intervention (hours) §	454	-	-	0·99	0·99	1·00	0·07
American Society of Anesthesiologists (ASA) Score at the time of primary intervention:							
1 or 2	267	9	3%	Reference	-	-	-
3 **	122	8	7%	1·94	0·76	4·92	0·16
4 or 5 **	30	10	33%	9·88	4·36	22·41	**<0·001**
N/A: no intervention ***	98	3	3%	0·90	0·25	3·29	0·88
What type of anaesthesia was used for the primary intervention? *							
General anaesthesia with endotracheal tube or laryngeal airway	331	24	7%	Reference	-	-	-
No general anaesthesia	100	1	1%	0·13	0 .01	1·00	**0·05**
N/A: no surgery or primary intervention undertaken.	86	5	6%	0·80	0·31	2·04	0·64
Who undertook the anaesthetic for the primary intervention? *							
Anaesthetic doctor	330	23	7%	Reference	-	-	-
Non-doctor anaesthetist	8	1	13%	1·79	0·27	11·71	0·54
No anaesthetic undertaken	179	6	3%	0·48	0·19	1·16	0·10
Who undertook the primary intervention? *							
Paediatric surgeon (or junior with paediatric surgeon assisting/ in the room)	394	22	6%	Reference	-	-	-
Non-paediatric surgeon	53	3	6%	1·01	0·31	3·27	0·98
N/A: no surgery or primary intervention undertaken	70	5	7%	1·27	0·50	3·26	0·60
Was a Surgical Safety Checklist used at the time of primary intervention?							
Yes	239	14	6%	Reference	-	-	-
No	107	11	10%	1·75	0·82	3·74	0·14
N/A: a conservative primary intervention or no surgery undertaken ***	171	5	3%	0·49	0·18	1·36	0·17
Total duration of antibiotics following primary intervention (days): §	454	-	-	0·94	0·87	1·01	0·13
Did the patient receive a blood transfusion?							
No: not required	366	9	3%	Reference	-	-	-
Yes: cross-matched OR not cross-matched	147	20	14%	5·53	2·57	11·87	**<0·001**
No: it was required but not available ****	3	1	33%	13·55	2·41	76·24	**0·003**
Did the patient require ventilation?							
No	433	18	4%	Reference	-	-	-
Yes and it was given	82	11	13%	3·22	1·58	6·58	**0·001**
Yes, but it was not available ****	2	1	50%	12·02	2·79	51·75	**0·001**

Did the patient require parenteral nutrition?							
No	303	16	5%	Reference	-	-	-
Yes and it was given	167	7	4%	0·79	0·33	1·89	0·60
Yes and it was sometimes available, but less than required **	38	2	5%	0·99	0·23	4·17	0·99
Yes, but it was not available **	8	5	63%	11·83	5·76	24·28	**<0·001**
Time to first feed (days): §	394	-	-	0·96	0·78	1·19	0·75
Time to full feeds (days): §	457	-	-	1·00	0·91	1·10	0·92
Duration of hospital stay (days): †	492	-	-	0·91	0·84	0·99	**0·04**
Did the patient have a surgical site infection? *							
No	324	19	6%	Reference	-	-	-
Yes	29	6	21%	3·52	1·52	8·14	**0·003**
N/A: no surgical wound	164	5	3%	0·51	0·19	1·36	0·18
Did the patient have a full thickness wound dehiscence? *							
No	343	22	6%	Reference	-	-	-
Yes	12	3	25%	3·89	1·34	11·26	**0·01**
N/A: no surgical wound	162	5	3%	0·48	0·18	1·24	0·13
Did the patient require a further unplanned intervention? *							
No	387	22	6%	Reference	-	-	-
Yes - percutaneous or surgical intervention	69	5	7%	1·27	0·49	3·25	0·61
N/A: no primary intervention undertaken	61	3	5%	0·86	0·26	2·80	0·80
Country income status:							
HIC	107	2	2%	Reference	-	-	-
MIC **	393	26	7%	3·53	0·85	14·69	0·08
LIC **	17	2	12%	6·29	0·94	41·82	**0·05**
**Condition specific variables**							
Time to first passage of meconium after birth: *							
Less than 24 hours	80	2	3%	reference	-	-	-
24-48 hours	148	6	4%	1·62	0·33	7·86	0·55
Over 48 hours	187	19	10%	4·06	0·97	17·06	0·06
Unknown or missing	102	3	3%	1·18	0·20	6·88	0·86
Features at presentation:							
Abdominal distension (yes)	460	24	5%	0·50	0·21	1·16	0·11
Bilious vomiting (yes)	190	11	5%	1·00	0·48	2·05	0·99
Poor feeding (yes)	189	12	6%	1·16	0·57	2·35	0·69
Non-bilious vomiting (yes)	103	7	7%	1·22	0·54	2·77	0·63
Suspected enterocolitis (yes)	96	7	7%	1·33	0·59	3·02	0·49
Perforation (yes) ‡	20	8	40%	9·04	4·60	17·75	**<0·001**
Length of aganglionosis?							
Rectal **	117	4	3%	reference	-	-	-
Sigmoid **	179	5	3%	0·82	0·22	2·98	0·76
Descending/transverse/ascending colon or small bowel **	86	9	11%	3·06	0·97	9·62	0·06
Unknown **	135	12	9%	2·60	0·86	7·85	0·09
Primary intervention: *							
Conservative	187	5	3%	reference	-	-	-
Stoma	196	20	10%	3·82	1·46	9·97	**0·01**
Pull-through	109	0	0%	-	-	-	**-**
Other (transanal posterior anorectal myectomy, palliative care or other)	25	5	20%	7·48	2·33	24·06	**0·001**
Did the patient have any condition specific complications within 30-days of primary intervention:							
Hirschsprung's associated enterocolitis (yes)	69	11	16%	3·76	1·87	7·56	**<0·001**
Electrolyte disturbance (yes)	47	13	28%	7·65	3·96	14·76	**<0·001**
Other (yes) ***	170	13	8%	1·56	0·78	3·14	0·21

*Excluded from the multivariable analysis due to low or no counts and inability to collapse categories. †Excluded from multivariable analysis due to missing data. ‡Excluded due to collinearity. §Excluded from multivariable analysis as this variable is a sub-group. **Category collapsed for the multivariable analysis due to low counts. ***N/A or 'other' groups were not presented on the forest plots. ****Excluded from multivariable analysis due to low counts and inability to combine with another category. CI: Confidence interval. HIC: High-income countries. LIC: Low-income countries. MIC: Middle-income countries. N/A: Not applicable. RR: Risk Ratio.

Supplementary Figure 1: Multivariable analysis of factors affecting mortality in low- and middle-income countries

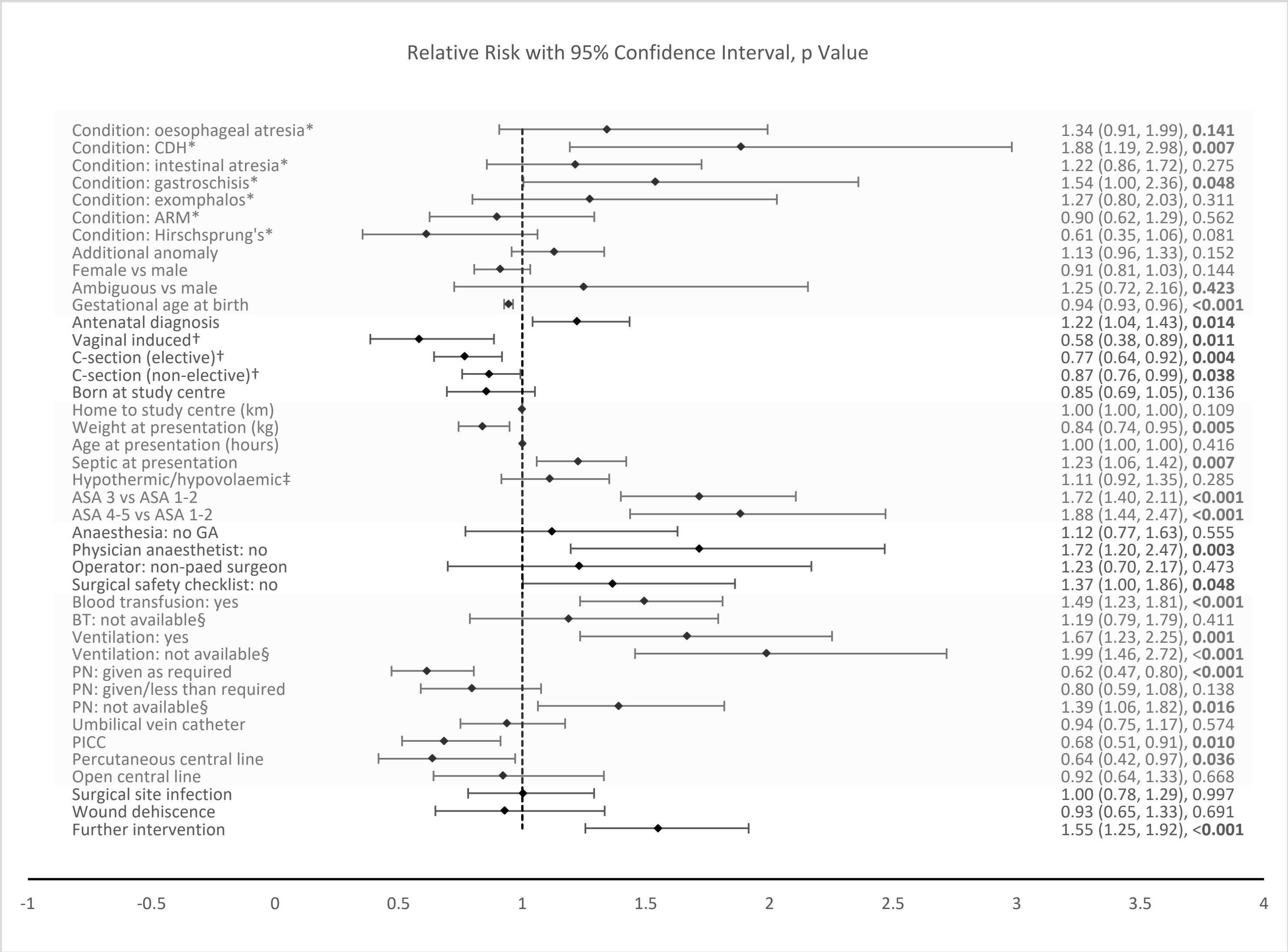

*Vs non-condition (i.e. study patients with oesophageal atresia vs study patients without oesophageal atresia). †Vs spontaneous vaginal delivery. ‡At presentation. §When required. ARM: Anorectal malformation. ASA: American Society of Anesthesiologists score at primary intervention. BT: Blood transfusion. CDH: Congenital diaphragmatic hernia. C-section: Caesarean section. GA: General anaesthetic. PICC: Peripherally inserted central catheter. PN: Parenteral nutrition. Non-paed surgeon: Non-paediatric surgeon. Further intervention: Need for unplanned re-intervention within 30 days of surgery. Additional anomaly includes additional study condition(s) if present. Figure shading demarcates the variables into the following groups, respectively: demographics, antenatal care and birth, distance from home to study hospital and clinical condition at presentation, intra-operative factors, perioperative factors, and secondary outcomes. Of the 2953 study patients from low- and middle-income countries, 2868 were included within this multivariable model (n=85 excluded due to missing data).

Supplementary Figure 2: Multivariable analysis of factors affecting mortality in high-income countries

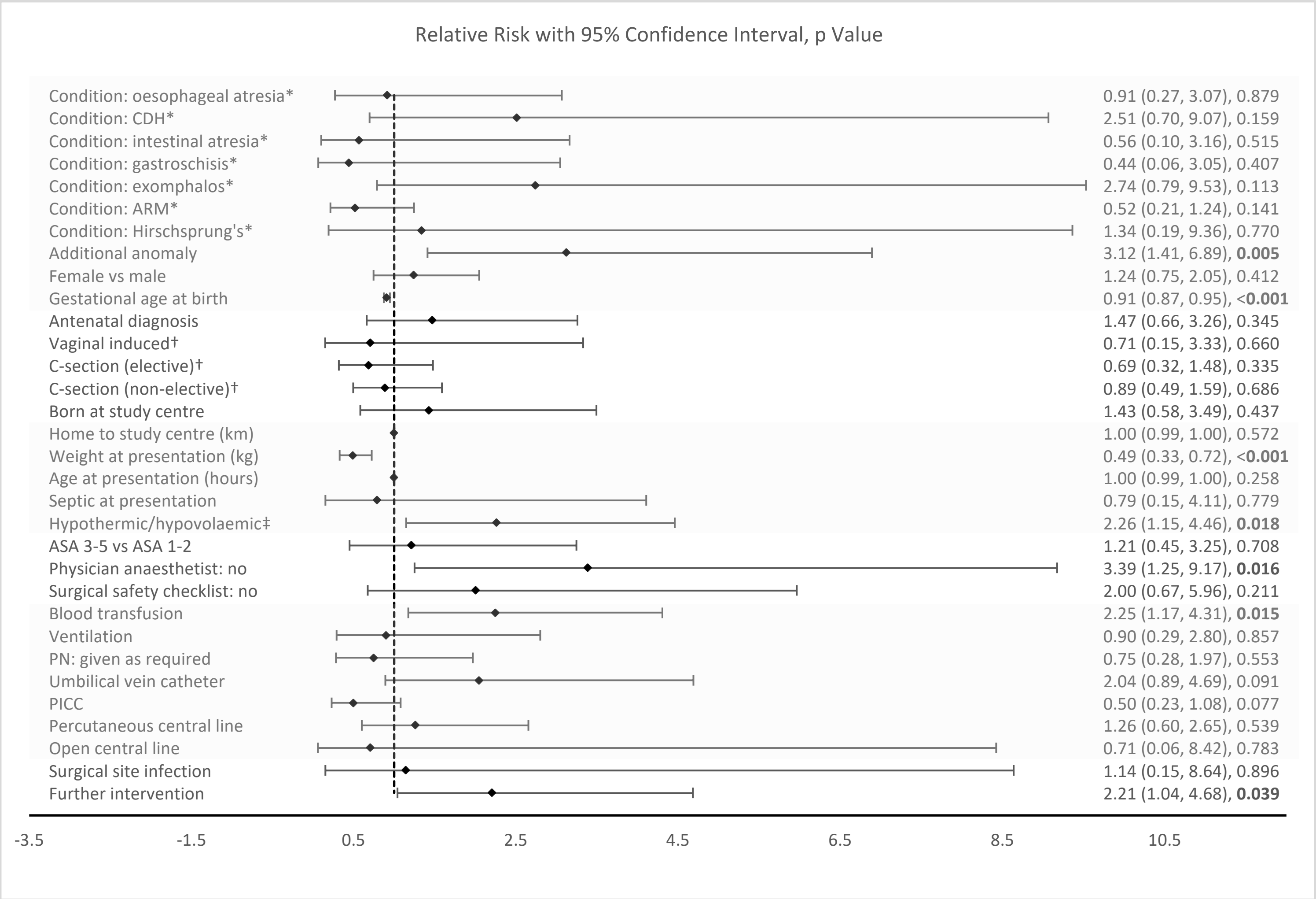

* Vs non-condition (i.e. study patients with oesophageal atresia vs study patients without oesophageal atresia). †Vs spontaneous vaginal delivery. ‡At presentation. ARM: Anorectal malformation. ASA: American Society of Anesthesiologists score at primary intervention. CDH: Congenital diaphragmatic hernia. C-section: Caesarean section. GA: General anaesthetic. PICC: Peripherally inserted central catheter. PN: Parenteral nutrition. Further intervention: Need for unplanned re-intervention within 30 days of surgery. Additional anomaly includes additional study condition(s) if present. Figure shading demarcates the variables into the following groups, respectively: demographics, antenatal care and birth, distance from home to study hospital and clinical condition at presentation, intra-operative factors, perioperative factors, and secondary outcomes. Of the 896 study patients from high-income countries, 857 were included within this multivariable model (n=39 excluded due to missing data).

Supplementary Figure 3: Multivariable analyses of factors affecting mortality for patients with oesophageal atresia

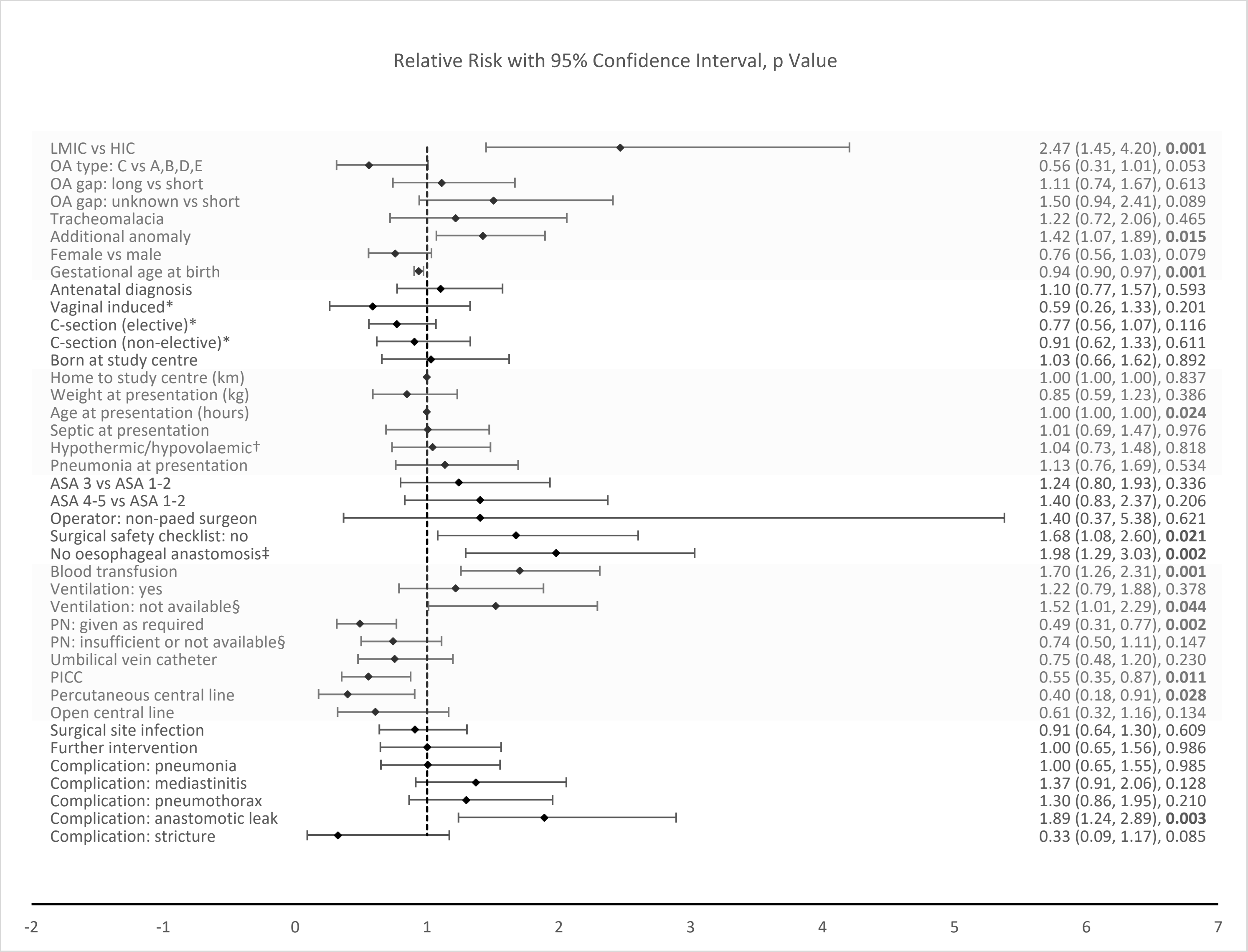

*Vs spontaneous vaginal delivery. †At presentation. ‡Vs primary oesophageal anastomosis. §When required. ASA: American Society of Anesthesiologists score at primary surgical intervention. C-section: Caesarean section. HIC: High-income country. LMIC: Low- or middle-income country. OA: Oesophageal atresia. PICC: Peripherally inserted central catheter. PN: Parenteral nutrition. Further intervention: Need for unplanned re-intervention within 30 days of surgery. Additional anomaly includes additional study condition(s) if present. Figure shading demarcates the variables into the following groups, respectively: demographics, antenatal care and birth, distance from home to study hospital and clinical condition at presentation, intra-operative factors, perioperative factors, and secondary outcomes and condition-specific complications. Of the 560 study patients with oesophageal atresia, 538 were included within this multivariable model (n=22 excluded due to missing data).

Supplementary Figure 4: Multivariable analyses of factors affecting mortality for patients with congenital diaphragmatic hernia

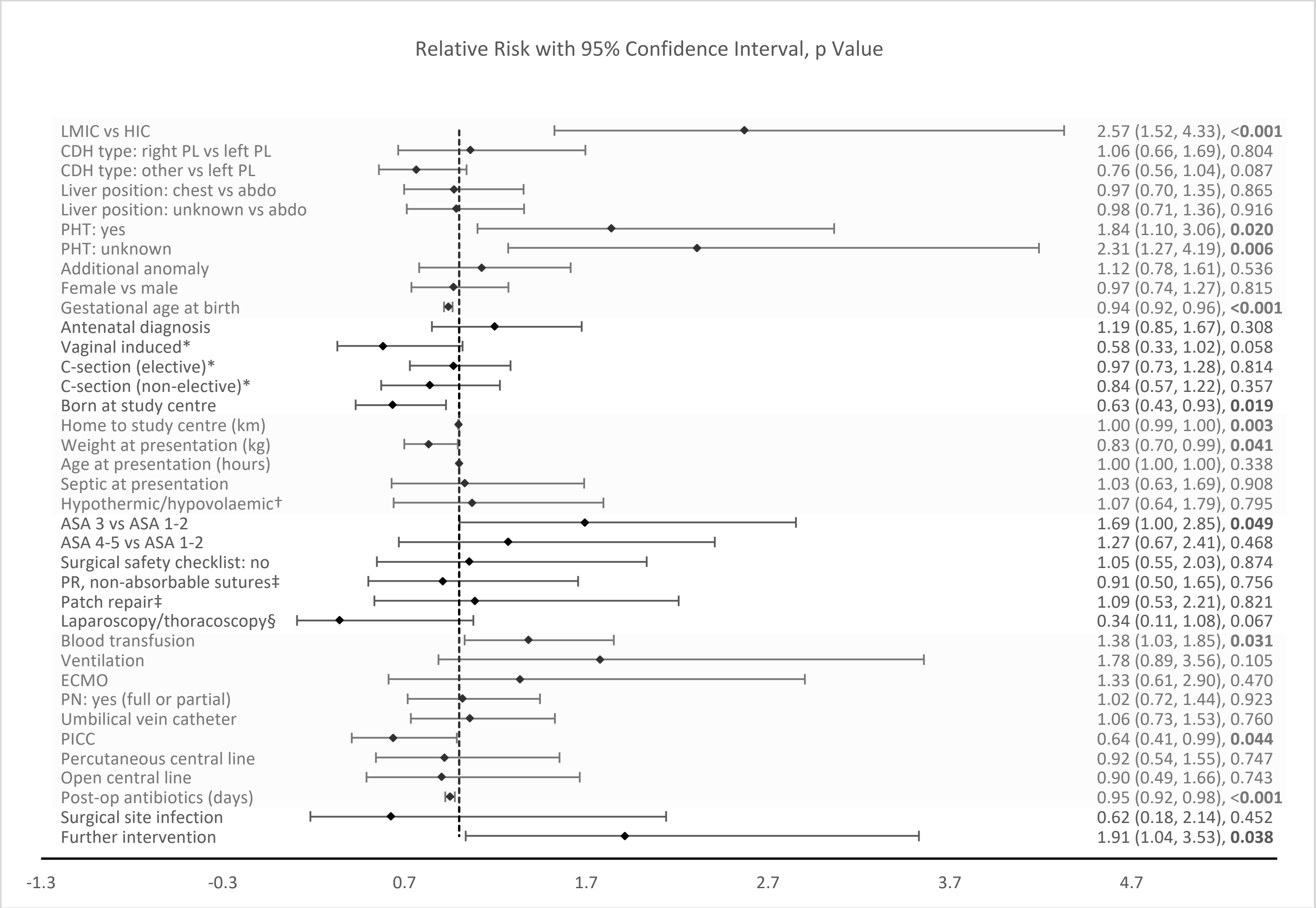

*Vs spontaneous vaginal delivery. †At presentation. ‡Vs primary repair with absorbable sutures. §Vs laparotomy/thoracotomy. ASA: American Society of Anesthesiologists score at primary surgical intervention. CDH: Congenital diaphragmatic hernia. C-section: Caesarean section. ECMO: Extracorporeal membrane oxygenation. HIC: High-income country. LMIC: Low- or middle-income country. PL: Posteriolateral (Bochdalek). PHT: Pulmonary hypertension. PICC: Peripherally inserted central catheter. PN: Parenteral nutrition. PR: Primary repair. Further intervention: Need for unplanned re-intervention within 30 days of surgery. Additional anomaly includes additional study condition(s) if present. Three patients who required ventilation, but it was unavailable all died (not included in multivariable model). Figure shading demarcates the variables into the following groups, respectively: demographics, antenatal care and birth, distance from home to study hospital and clinical condition at presentation, intra-operative factors, perioperative factors, and secondary outcomes. Of the 448 study patients with CDH, 403 were included within this multivariable model (n=45 excluded due to missing data).

Supplementary Figure 5: Multivariable analyses of factors affecting mortality for patients with intestinal atresia

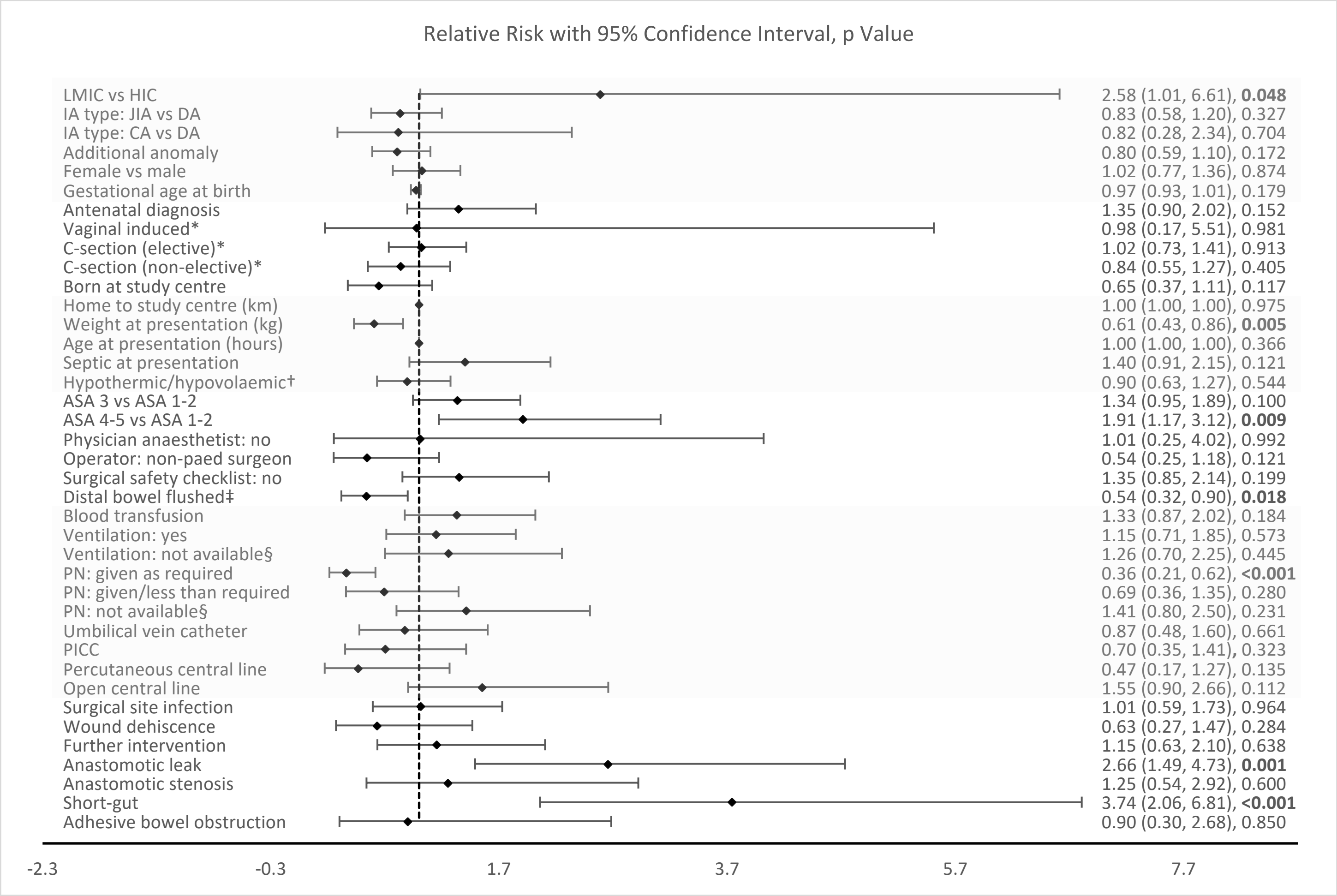

*Vs spontaneous vaginal delivery. †At presentation. ‡Intra-operatively to check for patency. §When required. ASA: American Society of Anesthesiologists score at primary surgical intervention. CA: Colonic atresia. C-section: Caesarean section. DA: Duodenal atresia. HIC: High-income country. IA: Intestinal atresia. JIA: Jejuno-ileal atresia. LMIC: Low- or middle-income country. PICC: Peripherally inserted central catheter. PN: Parenteral nutrition. Further intervention: Need for unplanned re-intervention within 30 days of surgery. Additional anomaly includes additional study condition(s) if present. Figure shading demarcates the variables into the following groups, respectively: demographics, antenatal care and birth, distance from home to study hospital and clinical condition at presentation, intra-operative factors, perioperative factors, and secondary outcomes and condition-specific complications. Of the 681 study patients with intestinal atresia, 659 were included within this multivariable model (n=22 excluded due to missing data).

Supplementary Figure 6: Multivariable analyses of factors affecting mortality for patients with gastroschisis

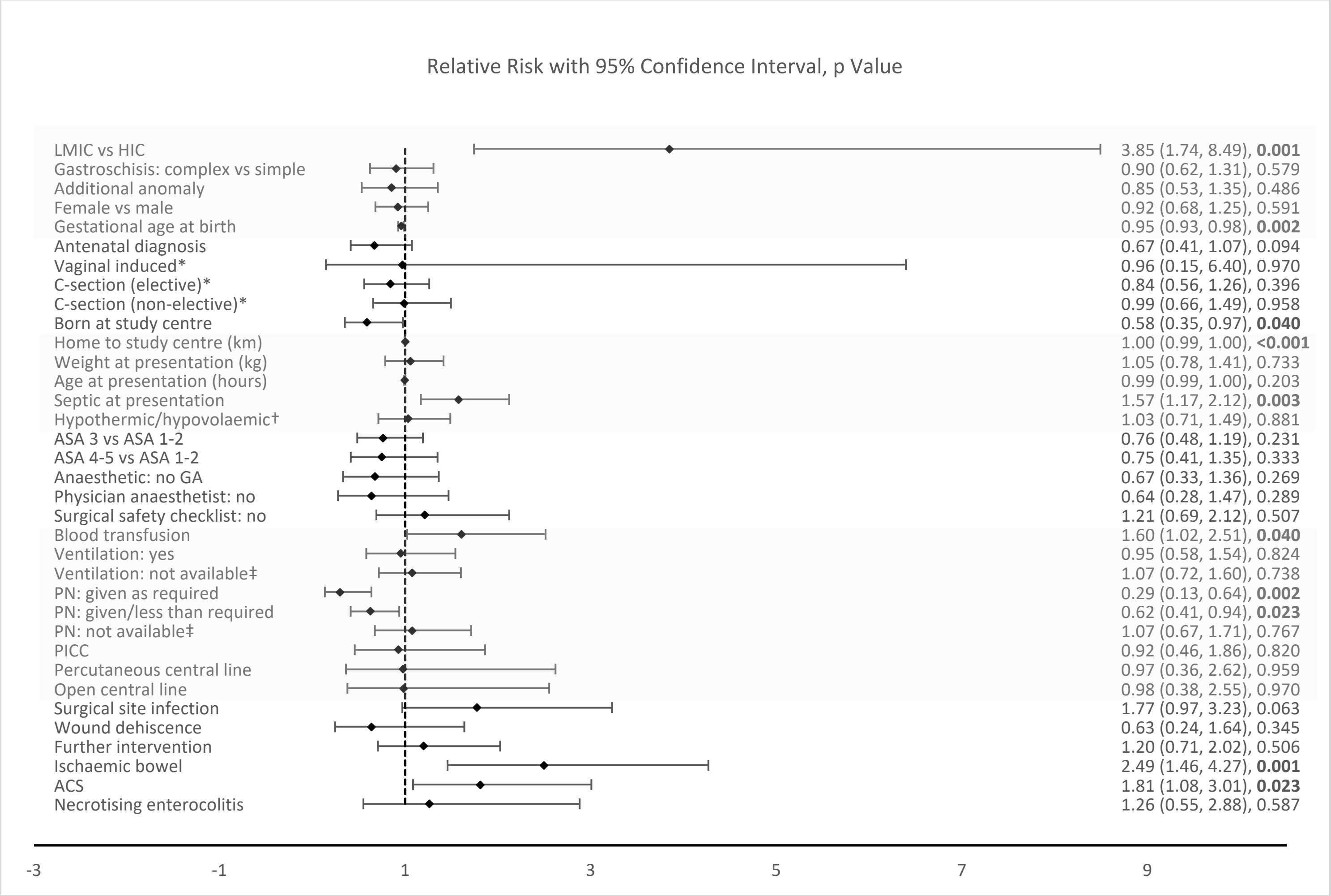

*Vs spontaneous vaginal delivery. †At presentation. ‡When required. ACS: Abdominal compartment syndrome. ASA: American Society of Anesthesiologists score at primary surgical intervention. C-section: Caesarean section. HIC: High-income country. LMIC: Low- or middle-income country. PICC: Peripherally inserted central catheter. PN: Parenteral nutrition. Further intervention: Need for unplanned re-intervention within 30 days of surgery. Additional anomaly includes additional study condition(s) if present. Figure shading demarcates the variables into the following groups, respectively: demographics, antenatal care and birth, distance from home to study hospital and clinical condition at presentation, intra-operative factors, perioperative factors, and secondary outcomes and condition-specific complications. Of the 453 study patients with gastroschisis, 441 were included within this multivariable model (n=12 excluded due to missing data).

Supplementary Figure 7: Multivariable analyses of factors affecting mortality for patients with exomphalos

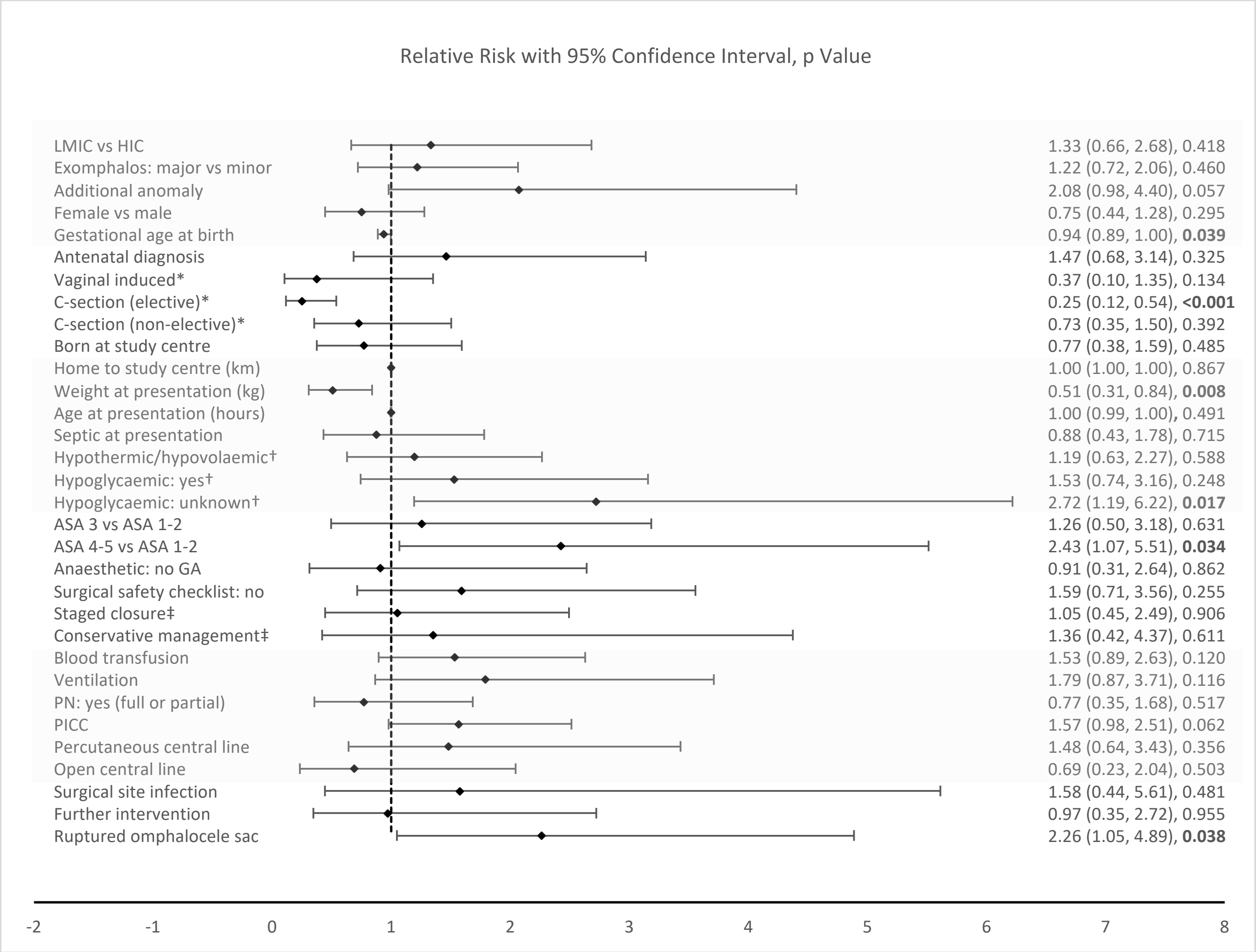

*Vs spontaneous vaginal delivery. †At presentation. ‡Vs primary operative closure. ACS: Abdominal compartment syndrome. ASA: American Society of Anesthesiologists score at primary surgical intervention. C-section: Caesarean section. HIC: High-income country. LMIC: Low- or middle-income country. PICC: Peripherally inserted central catheter. PN: Parenteral nutrition. Further intervention: Need for unplanned re-intervention within 30 days of surgery. Additional anomaly includes additional study condition(s) if present. Figure shading demarcates the variables into the following groups, respectively: demographics, antenatal care and birth, distance from home to study hospital and clinical condition at presentation, intra-operative factors, perioperative factors, and secondary outcomes and condition-specific complications. Of the 325 study patients with exomphalos, 293 were included within this multivariable model (n=32 excluded due to missing data).

Supplementary Figure 8: Multivariable analyses of factors affecting mortality for patients with anorectal malformation

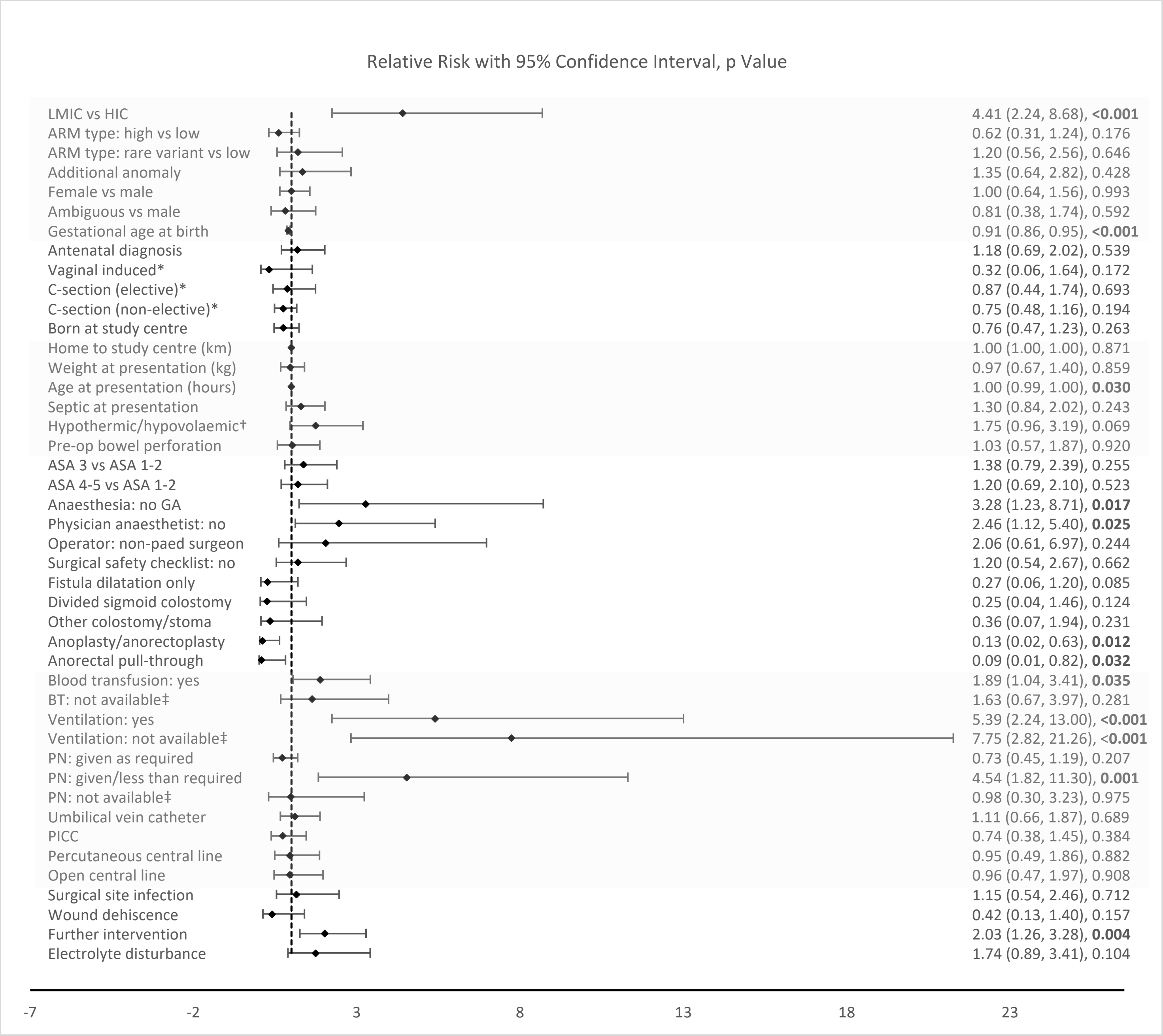

*Vs spontaneous vaginal delivery. †At presentation. ‡When required. ARM: Anorectal malformation. ASA: American Society of Anesthesiologists score at primary surgical intervention. C-section: Caesarean section. HIC: High-income country. LMIC: Low- or middle-income country. PICC: Peripherally inserted central catheter. PN: Parenteral nutrition. Further intervention: Need for unplanned re-intervention within 30 days of surgery. Additional anomaly includes additional study condition(s) if present. Figure shading demarcates the variables into the following groups, respectively: demographics, antenatal care and birth, distance from home to study hospital and clinical condition at presentation, intra-operative factors, perioperative factors, and secondary outcomes and condition-specific complications. Of the 991 study patients with ARM, 952 were included within this multivariable model (n=39 excluded due to missing data).

Supplementary Figure 9: Multivariable analyses of factors affecting mortality for patients with Hirschsprung's Disease

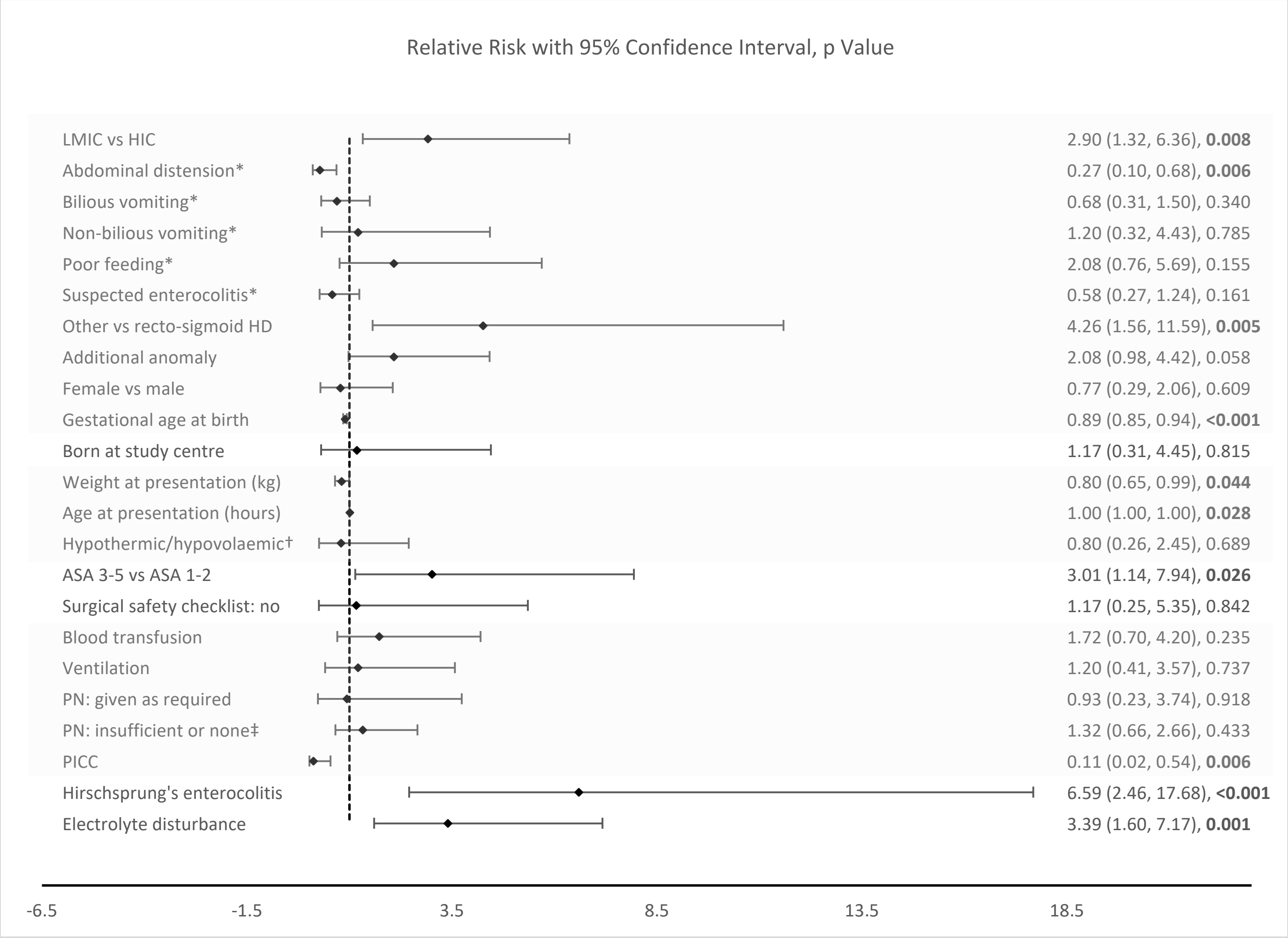

*Symptom at presentation. †At presentation. ‡When required. ASA: American Society of Anesthesiologists score at primary surgical intervention. HD: Hirschsprung's disease. HIC: High-income country. LMIC: Low- or middle-income country. PICC: Peripherally inserted central catheter. PN: Parenteral nutrition. Further intervention: Need for unplanned re-intervention within 30 days of surgery. Additional anomaly includes additional study condition(s) if present. Surgical intervention could not be included in the multivariable model because there were no deaths in the primary pull-through group (0/109). Figure shading demarcates the variables into the following groups, respectively: demographics, birth place, condition at presentation, intra-operative factors, perioperative factors, and condition-specific complications. Of the 517 study patients with Hirschsprung's disease, 494 were included within this multivariable model (n=23 excluded due to missing data).

Supplementary Table 18: Secondary outcomes

Variable	Total (n=3849) n, % (95% CI)	HIC (n=896) n, % (95% CI)	MIC (n=2860) n, % (95% CI)	LIC (n=93) n, % (95% CI)	P value*
**30-day post-intervention mortality:**	681, **17·7%** (16·5, 18·9)	50, **5·6%** (4·3, 7·3)	594, **20·8%** (19·3, 22·3)	37, **39·8%** (30·4, 50·0)	**<0·001**
**Surgical site infection:**					
Yes	335, **10·2%** (9·2, 11·3)	76, **9·5%** (7·6, 11·7)	253, **10·5%** (9·3, 11·8)	6, **9·4%** (4·2, 19·7)	0·407
No	2942, **89·8%** (88·7, 90·8)	728, **90·6%** (88·3, 92·4)	2156, **89·5%** (88·2, 90·7)	58, **90·6%** (80·3, 95·6)	-
Not applicable, no superficial wound	569	92	448	29	-
**Full thickness wound dehiscence:**					
Yes	102, **3·1%** (2·6, 3·8)	12, **1·5%** (0·8, 2·6)	89, **3·7%** (3·0, 4·5)	1, **1·6%** (0·2, 11·1)	**0·003**
No	3178, **96·9%** (96·2, 97·4)	792, **98·5%** (97·4, 99·2)	2325, **96·3%** (95·5, 97·0)	61, **98·4%** (88·9, 99·8)	-
Not applicable, no full thickness wound	566	92	443	31	-
**Further unplanned intervention:**					
Yes - percutaneous intervention	53, **1·5%** (1·2, 2·0)	25, **3·0%** (2·0, 4·3)	28, **1·1%** (0·7, 1·6)	0, **0·0%**	**0·047**
Yes - surgical intervention	400, **11·4%** (10·4, 12·5)	92, **10·9%** (9·0, 13·2)	298, **11·5%** (10·3, 13·0)	10, **15·6%** (8·5, 27·0)	-
No	3045, **87·1%** (85·9, 88·1)	728, **86·2%** (83·7, 88·3)	2263, **87·4%** (86·1, 88·6)	54, **84·4%** (73·0, 91·5)	-
Not applicable, no primary intervention	347	51	267	29	-
**Hospital stay amongst survivors (days), median (IQR):†**	15 (8, 25)	20 (12, 30)	14 (8, 23)	9 (5, 18)	**<0·001**
**Hospital stay amongst non-survivors (days), median (IQR):†**	6 (2, 13)	9 (3, 15)	6 (2, 13)	6 (3, 12)	0·280

*p values represent univariable testing between income country strata. †patients still in hospital at 30-days following admission (n=560) were included as 30. HIC: High-income country. IQR: Interquartile range. LIC: Low-income country. MIC: Middle-income country.

Supplementary Figure 10: Causes of death, % (no. of patients)

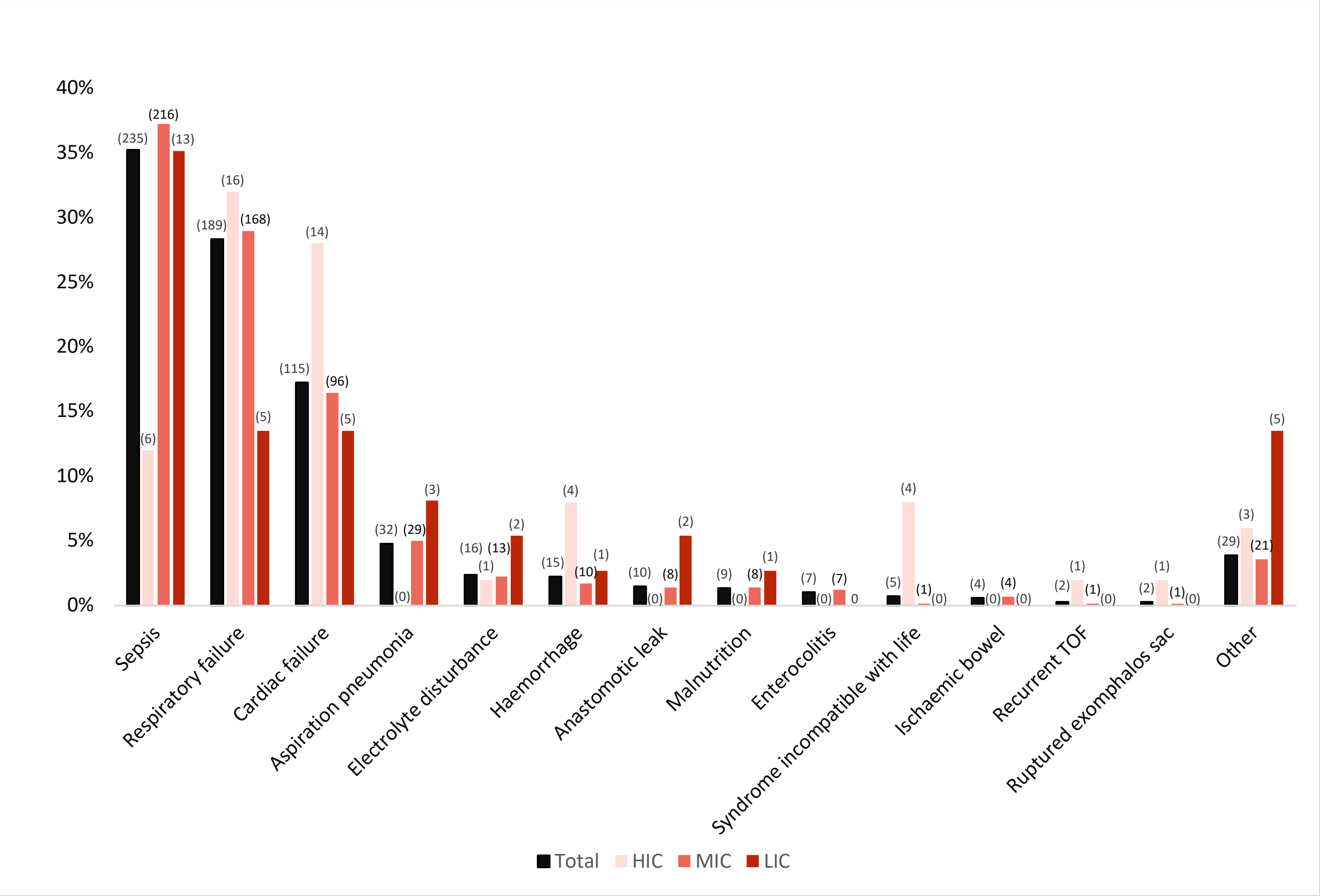

HIC: High-income countries. LIC: Low-income countries. MIC: Middle-income countries. TOF: Tracheo-oesophageal fistula.

Supplementary Table 19: Validation of the patient data
(64 patients with 66 study conditions from 21 hospitals [9 HIC, 11 MIC, 1 LIC] in 17 countries and 5 languages [English, Spanish, Portuguese, German and Lithuanian])

Variable being validated	N	Observed agreement	Expected agreement	Kappa*	SE
**Generic variables for all patients:**					
During which month did the patient present to your hospital?	64	98%	20%	0·98	0·060
Sex	64	100%	52%	1·00	0·125
Did the patient survive to discharge?	64	98%	73%	0·94	0·125
Did the patient require a further unplanned intervention?	64	80%	57%	0·53	0·095
What study condition does this patient have? (choice=Oesophageal atresia)	64	100%	78%	1·00	0·125
What study condition does this patient have? (choice=CDH)	64	100%	78%	1·00	0·125
What study condition does this patient have? (choice=Intestinal atresia)	64	100%	66%	1·00	0·125
What study condition does this patient have? (choice=Gastroschisis)	64	100%	81%	1·00	0·125
What study condition does this patient have? (choice=Exomphalos/ Omphalocele)	64	98%	77%	0·93	0·125
What study condition does this patient have? (choice=Anorectal malformation)	64	100%	66%	1·00	0·125
What study condition does this patient have? (choice=Hirschsprung's Disease)	64	100%	81%	1·00	0·125
**Condition specific variables:**					
**Oesophageal atresia (n=8):**					
Type of OA +/- TOF (Gross classification)	8	100%	78%	1·00	0·354
Long or short gap?	8	63%	48%	0·27	0·210
Primary intervention (choice=TOF ligation)	8	63%	50%	0·25	0·342
Primary intervention (choice=Oesophageal anastomosis)	8	100%	78%	1·00	0·354
Primary intervention (choice=Oesophagostomy)	8	100%	-	-	-
Primary intervention (choice=Gastrostomy)	8	100%	-	-	-
Primary intervention (choice=Ligation of the distal oesophagus)	8	100%	-	-	-
Primary intervention (choice=Gastro-oesophageal disconnection)	8	100%	-	-	-
Primary intervention (choice=Foker technique)	8	100%	-	-	-
Primary intervention (choice=Fundoplication)	8	100%	-	-	-
Other (including primary intervention for other congenital anomaly)	8	100%	-	-	-
Palliative care/no intervention	8	100%	78%	1·00	0·354
Surgical approach?	7	71%	39%	0·53	0·237
**Congenital diaphragmatic hernia (n=8):**					
Type of CDH	8	88%	44%	0·78	0·222
Did the patient receive extracorporeal membrane oxygenation (ECMO)?	8	100%	78%	1·00	0·354
Primary intervention	8	75%	44%	0·56	0·226
Surgical approach	7	100%	55%	1·00	0·284
**Intestinal atresia (n=14):**					
Type of intestinal atresia	14	93%	48%	0·86	0·239
Classification of duodenal atresia or colonic atresia (CA)	8	50%	34%	0·24	0·219
Classification of jejuno-ileal (JIA) atresia	5	100%	36%	1·00	0·326
Primary intervention for duodenal atresia	8	75%	56%	0·43	0·189
Surgical approach for duodenal atresia	7	100%	76%	1·00	0·378
Primary intervention for JIA or CA (choice=Primary anastomosis)	14	86%	65%	0·59	0·244
Primary intervention for JIA or CA (choice=Bowel resection)	14	93%	56%	0·84	0·264
Primary intervention for JIA or CA (choice=Loop stoma)	14	100%	87%	1·00	0·267
Primary intervention for JIA or CA (choice=Divided stoma)	14	100%	87%	1·00	0·267
Primary intervention for JIA or CA (choice=Division of web only)	14	100%	-	-	-
Primary intervention for JIA or CA (choice=Bishop-Koop stoma)	14	100%	-	-	-
Primary intervention for JIA or CA (choice=Santulli stoma)	14	100%	-	-	-
Primary intervention for JIA or CA (choice=Palliation)	14	100%	-	-	-
Primary intervention for JIA or CA (choice=Other)	14	100%	-	-	-
Surgical approach JIA or CA	4	100%	-	-	-
**Gastroschisis (n=7):**					
Type of gastroschisis: (choice=Simple)	7	100%	76%	1·00	0·378
Type of gastroschisis: (choice=Complex: associated with atresia)	7	100%	76%	1·00	0·378
Type of gastroschisis: (choice=Complex: associated with necrosis)	7	100%	-	-	-
Type of gastroschisis: (choice=Complex: associated with perforation)	7	100%	-	-	-
Type of gastroschisis: (choice=Complex: associated with closing gastroschisis)	7	100%	-	-	-
Primary intervention	7	71%	35%	0·56	0·239
Method of defect closure	6	83%	58%	0·60	0·279
On what day following admission was abdominal wall closure achieved?	6	67%	22%	0·57	0·210
**Exomphalos/ omphalocele (n=8):**					
Type of Exomphalos?	8	88%	69%	0·60	0·324
Hypoglycaemic on arrival?	8	88%	67%	0·62	0·248
Primary intervention	8	100%	41%	1·00	0·274
If conservative management, was a topical treatment applied to the exomphalos sac?	3	100%	56%	1·00	0·577
**Anorectal malformation (n=14):**					
Type of anorectal malformation (Krickenbeck classification)	14	64%	16%	0·58	0·100
Did the neonate have pre-operative bowel perforation?	14	100%	87%	1·00	0·267
What was the primary intervention undertaken? (choice=Fistula dilation: no surgery)	14	100%	87%	1·00	0·267
What was the primary intervention undertaken? (choice=Loop sigmoid colostomy)	14	100%	87%	1·00	0·267
What was the primary intervention undertaken? (choice=Divided sigmoid colostomy)	14	71%	46%	0·47	0·227
What was the primary intervention undertaken? (choice=Other stoma)	14	71%	55%	0·36	0·206
What was the primary intervention undertaken? (choice=Anoplasty)	14	100%	87%	1·00	0·267

What was the primary intervention undertaken? (choice=Laparoscopic-assisted pull-through)	14	93%	93%	0·00	0·000
What was the primary intervention undertaken? (choice=Loop transverse colostomy)	14	100%	-	-	-
What was the primary intervention undertaken? (choice=Divided transverse colostomy)	14	100%	-	-	-
What was the primary intervention undertaken? (choice=Posterior sagittal anorectoplasty (PSARP)	14	100%	-	-	-
What was the primary intervention undertaken? (choice=Abdominosacroperineal pull-through)	14	100%	-	-	-
What was the primary intervention undertaken? (choice=Abdominoperineal pull-through)	14	100%	-	-	-
What was the primary intervention undertaken? (choice=Palliative care/no intervention)	14	100%	-	-	-
What was the primary intervention undertaken? (choice=Other)	14	100%	-	-	-
**Hirschsprung's Disease (n=7):**					
Source of diagnosis of Hirschsprung's disease (choice=Mucosal biopsy)	7	100%	51%	1·00	0·378
Source of diagnosis of Hirschsprung's disease (choice=Full thickness biopsy)	7	86%	86%	0·00	0·000
Source of diagnosis of Hirschsprung's disease (choice=Barium enema)	7	86%	53%	0·70	0·360
Source of diagnosis of Hirschsprung's disease (choice=Not confirmed: suspected only)	7	86%	65%	0·59	0·344
Source of diagnosis of Hirschsprung's disease (choice=Genetic)	7	100%	-	-	-
Source of diagnosis of Hirschsprung's disease (choice=Anorectal manometry)	7	100%	-	-	-
Source of diagnosis of Hirschsprung's disease (choice=Other)	7	100%	-	-	-
Primary intervention	7	86%	24%	0·81	0·200
If primary pull-through was undertaken, did the patient have a covering stoma?	3	100%	-	-	-
Was it laparoscopic assisted?	3	100%	-	-	-
**Total: median (IQR)**		**100% (88%, 100%)**	**65% (48%, 78%)**	**0·96 (0·57, 1·00)**	

*Kappa could not be calculated for variables where all data were confined to one category. Interpretation of kappa: <0 no agreement, 0·01-0·2 none to slight, 0·21-0·40 fair, 0·41-0·6 moderate, 0·61-0·8 substantial, 0·81-1·00 almost perfect agreement. Ten hospitals that were randomly selected for validation were unable to provide patient data retrospectively. CDH: Congenital diaphragmatic hernia. HIC: High-income countries. LIC: Low-income countries. MIC: Middle-income countries. OA: Oesophageal atresia. TOF: Tracheo-oesophageal fistula.

Supplementary Table 20: Feedback surveys completed by local investigators at validating hospitals regarding the quality of data collection
(105 surveys from 27 hospitals [9 HIC, 17 MIC, 1 LIC] in 20 countries, completed in 7 languages [English, Spanish, Portuguese, German, Lithuanian, French and Turkish])

Local investigator survey questions and responses	All (n=105) N (%)	HIC (n=26) N (%)	MIC (n=72) N (%)	LIC (n=7) N (%)
**Do you think your team managed to identify all patients eligible for the study during the data collection period?**				
Yes	97 (92%)	24 (92%)	68 (94%)	5 (71%)
No	2 (2%)	0	2 (3%)	0
Unsure	6 (6%)	2 (8%)	2 (3%)	2 (29%)
**If no or unsure, what problems did you experience with identifying patients?**				
Patient died/discharged before a study team member could assess/confirm diagnosis	2	0	2	0
No centralised system to identify eligible patients throughout the hospital	1	1	0	0
Neonatal unit is at a different hospital to the study team	1	1	0	0
Long histology processing time – patients with Hirschsprung's on histology could have been missed	1	0	0	1
Heavy workload	1	0	1	0
**Could any eligible patients have been missed from study inclusion?**				
Yes	6 (6%)	3 (12%)	3 (4%)	0
No	91 (87%)	22 (85%)	65 (90%)	4 (57%)
Unsure	8 (8%)	1 (4%)	4 (6%)	3 (43%)
**If yes or unsure, how might patients have been missed from study inclusion?**				
Patients managed by different services/departments within study hospital	3	2	1	0
Patient died/discharged before a study team member could assess/confirm diagnosis	3	0	3	0
Long histology processing time	2	0	0	2
Inaccurate/missed diagnosis/ not referred to paediatric surgeons	2	0	2	0
No antenatal diagnosis	1	0	1	0
No parental consent	1	0	1	0
Missed during registration	1	0	1	0
**Are there any study conditions that were more likely to have been missed from study inclusion?***				
Oesophageal atresia +/- tracheo-oesophageal fistula	5 (5%)	0	5 (7%)	0
Congenital diaphragmatic hernia	7 (7%)	0	7 (10%)	0
Intestinal atresia	2 (2%)	0	2 (3%)	0
Gastroschisis	0	0	0	0
Omphalocele/ exomphalos	2 (2%)	1 (4%)	1 (1%)	0
Anorectal malformation	3 (3%)	1 (4%)	2 (3%)	0
Hirschsprung's disease	6 (6%)	0	4 (6%)	2 (29%)
None of the above	88 (84%)	25 (96%)	59 (82%)	4 (57%)
**If you selected any of the above conditions, why was this the case?**				
Missed/difficult diagnosis due to poor diagnostic tools, low index of suspicion, management by non-surgical teams without experience with such conditions	5	0	5	0
Patient died/discharged before a study team member reviewed patient/made diagnosis (including those conservatively managed by medical teams)	3	1	2	0
Prolonged histology time for patients with suspected Hirschsprung's disease	2	0	0	2
Patients managed by different services/departments	1	0	1	0
**How did you identify patients to include in the study?***				
Ward patient lists	52 (50%)	12 (46%)	36 (50%)	4 (57%)
Ward round	50 (48%)	8 (31%)	36 (50%)	6 (86%)
Operating room logbook	40 (38%)	5 (19%)	33 (46%)	2 (29%)
Planned operation lists	39 (37%)	9 (35%)	25 (35%)	5 (71%)
Handover	38 (36%)	10 (38%)	23 (32%)	5 (71%)
Personal knowledge of patients	36 (34%)	8 (31%)	25 (35%)	3 (43%)
Word of mouth	18 (17%)	4 (15%)	8 (11%)	6 (86%)
Other	11 (10%)	5 (19%)	6 (8%)	0
**If other, please provide further detail:**				
ICD codes	4	4	0	0
Clinics/ Emergency Room	2	0	2	0
Referrals by paediatricians/other doctors	2	0	2	0
Hospital computer system data	1	0	1	0
Neonatology logbook	1	1	0	0
**When you/ study team members were not present, were you able to identify all the patients to be included in the study on those days?**				
Yes	87 (83%)	22 (85%)	59 (82%)	6 (86%)
No	2 (2%)	0	2 (3%)	0
Unsure	5 (5%)	2 (8%)	3 (4%)	0
Not applicable	11 (11%)	2 (8%)	8 (11%)	1 (14%)
**How did you identify patients to be included in the study on days when you and the other Global PaedSurg local investigators were not present at the hospital?***				
Admission logs/patients register	31 (30%)	9 (35%)	21 (29%)	1 (14%)
Handover	19 (18%)	3 (12%)	16 (22%)	0
Word of mouth	14 (13%)	2 (8%)	7 (10%)	5 (71%)
Ward rounds	7 (7%)	1 (4%)	4 (6%)	2 (29%)
Operating room logbook	7 (7%)	3 (12%)	4 (6%)	0
Billing department/ICD codes	2 (2%)	2 (8%)	0	0
Prenatal diagnosis	1 (1%)	1 (4%)	0	0

Not applicable (one collaborator is always present/substitute was appointed)	44 (4%)	8 (31%)	34 (32%)	2 (29%)
**Do you have any concerns regarding the accuracy of the data collected on the patients included in the study?**				
Yes	0	0	0	0
No	101 (96%)	25 (96%)	69 (96%)	7 (100%)
Unsure	4 (4%)	1 (4%)	3 (4%)	0
**If yes or unsure, what data points might be inaccurate and what were the challenges for collecting this data?**				
Some patients left the hospital before the diagnosis/ investigations were complete	1	0	1	0
Conditions such as ARM with perineal fistula require expert surgical diagnosis which might not be available for the neonatology team managing the patient	1	0	1	0
Human error in data collection	1	1	0	0
No antenatal record cards for antenatal data	1	0	1	0
**Were any of the data points more difficult to collect accurately? If so, which ones and why?**				
None	81	22	56	3
Diagnosis: lack of expert input/ classification not normally used by the study team/ histology time	5	0	2	3
Missing data within patient registers/notes (i.e means of transport to the hospital)	5	-	5	-
Distance from hospital difficult to calculate for patients from rural regions not on the map	4	1	3	-
Patient follow up – difficult to 30-days post intervention	3	2	0	1
Lack of information from referring centres/ information regarding care prior to arrival	3	1	2	-
Specific data from prescriptions such as number of days on parenteral nutrition	2	0	2	0
Gestational age at birth – some parents were unsure	1	0	1	0
Lack of equipment i.e no neonatal blood pressure cuff	1	-	1	-
Time from birth to presentation sometimes difficult to calculate	1	-	1	-
Antenatal care information – not always available	1	-	1	-

*Denominator is the number of completed surveys as more than one answer could be selected. Percentages not calculated for data from free text boxes. Percentages have been rounded and may not total 100. At four validating hospitals a feedback survey was not completed by study collaborators. HIC: High-income countries. ICD: International Classification of Diseases. LIC: Low-income countries. MIC: Middle-income countries.

Supplementary Table 21: Feedback surveys completed by validating local investigators
(31 surveys from 31 hospitals [12 HIC, 18 MIC, 1 LIC] in 20 countries, completed in 6 languages [English, Spanish, Portuguese, German, Lithuanian and Turkish])

**Validator survey questions and responses**	**All (n=31)** N (%)	**HIC (n=12)** N (%)	**MIC (n=18)** N (%)	**LIC (n=1)** N (%)
**Do you think your team managed to identify and include all eligible patients for the study during the data collection period?**				
Yes	29 (93%)	12 (100%)	16 (89%)	1 (100%)
No	1 (3%)	0	1 (6%)	0
Unsure	1 (3%)	0	1 (6%)	0
**If you answered no or unsure, what problems might they have experienced when trying to identify patients?**				
Patients managed by different services/departments within study hospital	1	0	1	0
**Have you managed to identify any additional patients that were eligible for the study, but were not included in the original data collection?**				
Yes	2 (6%)	0	2 (11%)	0
No	29 (94%)	12 (100%)	16 (89%)	1 (100%)
**If yes, through what sources were you able to identify additional patients? Why do you think these patients might have been missed from study inclusion?**				
Hospital admission staff e.g. paediatric and surgical residents triaging patients	1	0	1	0
Admission records on the ward	1	0	1	0
**Are there any study conditions that were more likely to have been missed from study inclusion?***				
Oesophageal atresia	3 (10%)	1 (8%)	2 (11%)	0
Congenital diaphragmatic hernia	3 (10%)	0	3 (17%)	0
Intestinal atresia	3 (10%)	1	2 (11%)	0
Gastroschisis	2 (6%)	0	2 (11%)	0
Omphalocele/Exomphalos	4 (13%)	1 (8%)	3 (17%)	0
Anorectal malformation	6 (19%)	3 (25%)	3 (17%)	0
Hirschsprung's disease	18 (58%)	6 (50%)	11 (61%)	1 (100%)
**If you selected any of the above conditions, why might this have been the case?**				
Late diagnosis due to complex diagnosis/mild presentation/treated as another diagnosis	14	6	8	0
Histopathological delay	4	0	3	1
Patients managed by different services/departments within study hospital	1	0	1	0
Validator forced to select an option due to survey design	9	4	5	0
**What sources did you utilise to check whether all patients had been included in the study?***				
Operating room log book	19 (61%)	7 (58%)	11 (61%)	1 (100%)
Ward patient lists	16 (52%)	4 (33%)	11 (61%)	1 (100%)
Admission records	13 (42%)	3 (25%)	9 (50%)	1 (100%)
Personal knowledge of patients	11 (35%)	3 (25%)	7 (39%)	1 (100%)
Word of mouth/ discussion with colleagues	10 (32%)	5 (42%)	4 (22%)	1 (100%)
Elective operation lists	6 (19%)	4 (33%)	2 (11%)	0
Other	6 (19%)	3 (25%)	3 (17%)	0
**If other, please provide further detail:**				
Electronic medical records or database	5	3	2	0
NICU/PICU admission register & neonatal ward register	1	0	1	0
**If the Global PaedSurg local investigators at your centre were not present at the hospital for one or more of the days during the data collection period, do you think they were able to identify all the patients to be included in the study on those days?**				
Yes	27 (87%)	12 (100%)	14 (78%)	1 (100%)
No	1 (3%)	0	1 (6%)	0
Unsure	3 (10%)	0	3 (17%)	0
**How would they identify patients to be included in the study on days when they were not present at the hospital?**				
Discussion/updates from colleagues	11 (36%)	4 (33%)	7 (39%)	0
Electronic medical records or databases	7 (23%)	6 (50%)	1 (6%)	0
Not applicable: as a collaborator always present	5 (16%)	1 (8%)	4 (22%)	0
Hospital/ward records or operation room logbook	5 (16%)	1 (8%)	4 (22%)	0
Admission records	2 (7%)	0	1 (6%)	1 (100%)
Outpatient clinic	1 (3%)	0	1 (6%)	0
**Do you have any concerns regarding the accuracy of the data collected on the patients included in the study?**				
Yes	2 (7%)	1 (8%)	1 (6%)	0
No	28 (90%)	11 (92%)	16 (89%)	1 (100%)
Unsure	1 (3%)	0	1 (6%)	0
**If yes or unsure, what data points might be inaccurate and what were the challenges for collecting this data?**				
Operative findings - information was missing from the operation reports, with inferences made based on procedure performed	1	1	0	0
Month of the data collection – patients sometimes included in month corresponding to procedure date, not admission date	1	0	1	0
Poor documentation – requiring in-person discussion with responsible clinician in order to clarify certain points	1	0	1	0
**Were any of the data points more difficult to collect accurately?**				
Yes	7 (23%)	4 (33%)	3 (17%)	0
No	24 (77%)	8 (67%)	15 (83%)	1 (100%)

**If so, which ones and why?**				
CVC placement – overcome by reviewing patient data e.g. radiology	1	1	0	0
Operative findings – poor documentation	1	1	0	0
Gastroschisis definitions – may be interpreted differently by different observers	1	1	0	0
Distance to hospital – inferred from most direct route to hospital	1	1	0	0
Type of colostomy for anorectal malformation – overcome by reviewing theatre notes	1	0	1	0
Perianal fistula in ARM – diagnosis requires subspecialty physical examination	1	0	1	0
ASA score (not documented) and high output stoma in anorectal malformation (output not accurately measured)	1	0	1	0
**Were there any data points that you were unable to identify retrospectively during the validation process?**				
Yes	1 (3%)	1 (8%)	0	0
No	30 (97%)	11 (92%)	18 (100%)	1 (100%)
**If yes, what were your challenges? Do you think the Global PaedSurg local investigators at your centre would have been able to collect these data points prospectively during the study?**				
Operative findings – prospectively this might have been easier as the responsible surgeon could be questioned directly	1	1	0	0

*Denominator is the number of completed surveys as more than one answer could be selected. Percentages not calculated for data from free text boxes. Percentages have been rounded and may not total 100. When interpreting the above findings it is important to note that the validating collaborator collected the validation data retrospectively, whereas the study collaborators collected the data for the study prospectively and hence may not have experienced the same problems with collecting data from patient records and hospital documentation. ARM: Anorectal malformation. ASA: American Society of Anesthesiologists. CVC: Central venous catheter. HIC: High-income countries. LIC: Low-income countries. MIC: Middle-income countries. NICU: Neonatal intensive care unit. PICU: Paediatric intensive care unit.

Supplementary Table 22: A comparison of the number of patients in the main study database and the number of eligible patients identified by validating local investigators
(23 hospitals [11 HIC,11 MIC,1 LIC] in 18 countries, 4 languages [English, Spanish, German, Lithuanian])

**Study condition**	**All N**			**HIC N**			**MIC N**			**LIC N**		
	Main database	Validator survey	Difference	Main database	Validator survey	Difference	Main database	Validator survey	Difference	Main database	Validator survey	Difference
Oesophageal atresia	13	9	-4	6	5	-1	7	4	-3	0	0	0
CDH	15	15	0	8	8	0	7	7	0	0	0	0
Intestinal atresia	17	18	+1	6	7	+1	10	10	0	1	1	0
Gastroschisis	13	13	0	5	6	+1	8	7	-1	0	0	0
Exomphalos	9	11	+2	1	1	0	8	10	+2	0	0	0
Anorectal malformation	19	22	+3	5	5	0	14	17	+3	0	0	0
Hirschsprung's disease	8	10	+2	4	3	-1	4	7	+3	0	0	0
**Total (conditions)**	**94**	**98**	**4**	**35**	**35**	**0**	**58**	**62**	**4**	**1**	**1**	**0**
**Total (patients*)**	**92**	**96**	**4**	**34**	**34**	**0**	**57**	**61**	**4**	**1**	**1**	**0**

*Discrepancy between total patients and total conditions is a result of two patients having two co-existing study conditions: 1) intestinal atresia and anorectal malformation; 2) exomphalos and anorectal malformation. Data was not available for eight hospitals. CDH: Congenital diaphragmatic hernia. HIC: High-income countries. LIC: Low-income countries. MIC: Middle-income countries.
